# Acute, subchronic toxicity and genotoxicity studies of JointAlive, a traditional Chinese medicine formulation for knee osteoarthritis

**DOI:** 10.1371/journal.pone.0292937

**Published:** 2023-10-17

**Authors:** Yuanyuan Wang, Li Li, Yanling Mu, Shanglong Wang, Xin Li, Jiancheng Zong, Shengcan Zou, Zimin Liu, Dehai Gao

**Affiliations:** 1 Chenland Nutritionals, Inc., Irvine, California, United States of America; 2 School of Pharmacy and Pharmaceutical Sciences, Shandong First Medical University, Jinan, Shandong, China; 3 Chenland Research Institute, Qingdao City, Shandong Province, China; Zagazig University, EGYPT

## Abstract

**Aim:**

In vivo and in vitro toxicity tests of JointAlive^®^ were studied in animal models to support the safe use of JointAlive^®^ as a drug for knee osteoarthritis treatment.

**Methods:**

The acute toxicity study in Sprague Dawley (SD) rats was conducted at a 20 g/kg bw/day dose of JointAlive^®^. For 13-week subchronic toxicity tests, SD rats were orally dosed daily with 0.5, 1.5 and 5 g/kg bw/day of JointAlive^®^. To assess the potential genotoxicity, Ames test, cellular chromosome aberration and mouse micronucleus test in vivo were carried out.

**Results:**

Based on a lack of notable findings other than histopathology finding of co-incidental prostate inflammation at the high dose, the “No Observed Adverse Effect Level (NOAEL)” of JointAlive^®^ was concluded as 5 g/kg bw/day in males and females. Results also indicated that JointAlive^®^ has no risk of genotoxicity.

**Conclusions:**

General toxicity and genotoxicity studies empirically demonstrated that JointAlive^®^ poses a low risk of potential health risks, providing safety supports for the application of JointAlive^®^ as a potential drug candidate to treat knee osteoarthritis.

## Introduction

Knee osteoarthritis (OA), a common global joint disease, causes pain and even disability in the elderly. From 2000 to 2010, nearly 27 million Americans had symptomatic knee OA, more than half of whom have advanced OA [1–3]. In addition, a longitudinal study of 17,128 Chinese residents aged 45 and over showed an overall prevalence of Symptomatic Knee OA of 8.1% [4]. Symptomatic knee OA is more common in rural areas of China and has significantly increased in the elderly population [5,6] who often experience chronic pain [7,8]. Due to aging and obesity, many people developed knee OA, and physical limitations and mental illness can affect the quality of life of patients to varying degrees [9–13]. Without effective symptomatic treatment, the burden on patients with knee OA will increase. Therefore, interventions are aimed at reducing pain and enhancing mobility, and function to promote the management on OA. Several recent guidelines question the safety and efficacy of standard Western medical treatments for OA [14,15]. For example, the 2013 Guidelines of the American College of Osteopathic Physicians provide "uncertainty" recommendations for acetaminophen and intraarticular corticosteroids. Oral non-steroidal anti-inflammatory drugs (NSAIDs) are only conditionally recommended by the International OA Research Association and the American College of Rheumatology (ACR) [16,17]. However, long-term use of non-selective NSAID has been reported to increase the risk of side effects. Therefore, complementary and integrated therapies are very popular in the treatment of OA [18]. In fact, an increasing number of patients with chronic musculoskeletal pain have been reported to use this therapy which is a component of Traditional Chinese Medicine (TCM) [19,20]. Classical therapies such as herbs used occupies a very important position in Asian culture for thousands of years [21]. TCM has been recognized worldwide as a complementary therapy for the treatment of joints. Currently, the OARSI (OA Research Society International) guidelines include the use of traditional Chinese medicine, such as acupuncture and Tai chi, and have been criticized and advocated for non-surgical treatment of OA [22,23].

*Epimedium brevicornum*, *Dioscorea nipponica* and *Salvia miltiorrhiza* are historical Chinese medicines in relation to OA treatment. JointAlive^®^ is composed of their extracts including icariin, dioscin, tanshinone IIA and other active ingredients. Our previous research has confirmed the effectiveness of JointAlive^®^. In the rat knee OA model, it significantly relieved pain and improved performance, without serious adverse events [24]. The purpose of this study was to assess acute, subchronic toxicity and genotoxicity of JointAlive^®^ in vitro and in vivo to provide a basis for further clinical trials.

## Materials and methods

### Preparation of test substance

JointAlive^®^, a novel patented Chinese medicine formula (US 11331339 B2) was provided by Chenland Nutritionals, Inc., consisting of standardized extracts from *Epimedium brevicornum* leaves, *Dioscorea nipponica* rhizome and *Salvia miltiorrhiza* root and rhizome in the following blending proportion 60%:36%:4% and was extracted with ethanol. In brief, *Epimedium brevicornum* leaves were extracted with 70% ethanol, filtered, and concentrated to be ethanol-free. Then the mixture was purified with macroporous resin and concentrated before drying at 60°C under reduced pressure, crushed, and passed through an 80-mesh sieve, and standardized to 10% icariin extract with dextrin. *Dioscorea nipponica* rhizome was crushed preliminarily and extracted with 80% ethanol, filtered, and concentrated before drying at 60°C under reduced pressure, powdered, and standardized to 6% dioscin extract with dextrin. *Salvia miltiorrhiza* root and rhizome were also crushed preliminarily and extracted with 80% ethanol, filtered, and concentrated. Standing for 12 h, the precipitate was dried, powdered, and standardized to 5% tanshinone IIA extract with dextrin. The final product was produced as a brown powder. The biomarker contents of icariin, diosgenin and tanshinone IIA was not less than 5.5%, 2.0% and 0.2% respectively. To furtherly confirm the chemical components in JointAlive^®^, analyses were performed using LC-MS: icariin, diosgenin acetate, tanshinone IIA, epimedin C, tanshinone I, scutellarein 5,6,7,4’-tetra-methyl ether, 8-prenylquercetin 4’-methyl ether 3-rhamnoside, ferulic acid, 1,2-dihydrotanshinone, diosgenin acetate, cryptotanshinone, delta3,5-deoxytigogenin, and salvirecognone, as previously reported [24].

### Animals and ethics statement

Sprague-Dawley rats (SPF grad) and ICR mice (SPF grad) were purchased from Shanghai Jihui Laboratory Animal Care Co., Ltd (Shanghai, China). The animals were quarantined and acclimatized for a 5–6 d period. During the experiments, the animals were maintained under the conditions: 20–25°C, 30–70% RH, 12 h light/12 h dark cycle, and ≥15 times/h ventilation. Standard animal feeds were purchased from Shanghai Shilin Co., Ltd (Shanghai, China). All animal use was approved by Institutional Animal Care and Use Committee (IACUC) of Suzhou Xishan Zhongke Pharmaceutical Research and Development Co., Ltd, (Approval Nos. IM19030425 for the micronucleus test, IR19031327 for the acute toxicity test and IR19012307 for the subchronic toxicity test). Veterinarians supervised the care and welfare of animals. Animal welfare was conducted as per Guide for the Care and Use of Laboratory Animals (National Research Council, NRC, 2011.).

All the experiments in this study were performed under the (Chinese) Good Laboratory Practice (GLP) conditions, which is a requirement for safety studies to support clinical trials. This study was conducted in compliance with the Quality Management Practice for Non-Clinical Drug Studies (CFDA Order No. 34) implemented by the former China Food and Drug Administration (CFDA) (now the "National Medical Products Administration (NMPA)") on September 1, 2017.

The long-term toxicity test program was formulated, referring to the Technical Guidelines for Toxicity Test of Repeated Drug Administration (CFDA) (now "National Medical Products Administration (NMPA)", May 13, 2014). For the formulation of acute toxicity experiment program, refer to the Technical Guiding Principles for Toxicity Study of Single Drug Administration (China Food and Drug Administration, May 2014). For the study of chromosomal aberrations and bacterial recovery mutations, the Technical Guidelines for Research on Genotoxicity of Drugs issued by China Food and Drug Administration on March 12, 2018 and the International Coordination Committee ICH Tripartite Coordination Guidelines S2 (R1) for technical Requirements for Drug Registration for Human Use were followed.

### Acute oral toxicity study

40 SD rats of 6–7 weeks of age were randomly divided into a control group (pure water, 0 g/kg bw/day) and a treatment group (JointAlive^®^, 20 g/kg bw/day), 10 males and 10 females from each group. Animals were fasted overnight (without restricting water intake) prior to dosing. Test substances were administered by intragastric gavage twice a day in a volume of 20 mL/kg each time for 14 days. Observations were performed before each dosing and close observation were performed within 1 hour after each administration. General appearances and mortality were observed daily. Body weights were measured before treatment and on day 2, 7 and 14 after administration. On day 15, they were subjected to gross necropsy.

### 13-week repeated dose oral toxicity study

120 SD rats of 7–8 weeks of age were randomly divided into 4 groups (10 males and females/treatment group, 5 males and females/recovery group) by body weights, including one control (pure water, 0 g/kg bw/day) and 3 treatment groups at 0.5, 1.5 and 5 g/kg bw/day dosage. Test substances were given orally once a day in a volume of 10 mL/kg for 13 weeks followed by a 28-day recovery period.

#### Clinical observations, body weight, food consumption and ophthalmic examination

Each animal was observed at least twice daily for mortality, and general clinical observations. The detailed clinical signs of each animal were recorded. Rat body weight was measured 1 day prior and weekly after treatment. The changes in individual body weight were calculated. Food consumption was calculated once weekly.

An ophthalmic examination was performed on each animal before administration. At the end of the administration and recovery period, the animals in the control group and the high-dose group were examined with an ophthalmoscope (Vision Technology Co., Ltd, Suzhou, China). Mydriasis was induced by applying Tropicamide eye drops (Jemincare Wuxi Shan He Pharmaceutical Co., Ltd., Wuxi, China) onto the eyes before the examination. The eye examination includes the eyelid, conjunctiva, cornea, sclera, iris, pupil, lens, vitreous body and fundus.

#### Hematology and clinical biochemistry

Rats were anesthetized with Zoletil 50 (75 mg/kg) after fast overnight (without restricting water intake) and blood was collected from the abdominal aorta for hematological and blood chemistry analysis. Blood samples were collected into ethylene diamine tetraacetic acid (EDTA) tubes on the 92^nd^ day and the 29^th^ day of the recovery period, respectively. The serum obtained by centrifugation after coagulation was used for blood chemical examination. Hematological parameters included total red blood cell count (RBC), total leukocyte count (WBC), absolute neutrophil count (NEUT), absolute lymphocyte count (LYMPH), absolute monocyte count (MONO), absolute eosinophil count (EO), absolute basophil count (BASO), relative neutrophil count (NEUT%), relative lymphocyte count (LYMPH%), relative eosinophil count (EO%), relative monocyte count (MONO%), relative basophil count (BAS%), total and differential leucocyte count, hemoglobin (HGB), hematocrit (HCT), mean corpuscular volume (MCV), mean corpuscular hemoglobin concentration (MCHC), mean corpuscular hemoglobin (MCH), platelets (PLT), relative reticulocyte count (RET%), absolute reticulocyte count (RET), fibrinogen (FIB), prothrombin time (PT) and activated partial thromboplastin time (APTT). Serum biochemical parameters included total bilirubin (TBIL), total protein (TP), albumin (ALB), globulin (GLOB), albumin/globulin ratio (A/G), alanine aminotransferase (ALT), aspartate aminotransferase (AST), alkaline phosphatase (ALP), gamma-glutamyl transferase (GGT), creatinine kinase (CK), blood urea nitrogen (UREA), creatinine (CREA), glucose (GLU), triglycerides (TG), cholesterol (CHOL), potassium (K^+^), sodium (Na^+^) and chloride (Cl^-^).

#### Urinalysis

On the 90^th^ day and the 27^th^ day of the recovery period, all rats were placed in metabolic cages to collect fresh urine samples individually. The following parameters were measured: color (COL), turbidity (TURB), pH, nitrite (NIT), glucose (GLU), specific gravity (SG), occult blood (BLD), protein (PRO), bilirubin (BIL), urobilinogen (URO), ketones (KET), and leukocyte (LEU).

#### Necropsy and histopathology

After blood collection and sacrifice on the 92^nd^ day and the 29^th^ day of the recovery period, all animals of the treatment groups and recovery groups were examined grossly for any changes in general appearance, organs in the abdominal, thoracic, and cranial cavities, and other organs. Organ and final body weight ratios were also determined. Tissues and organs were fixed in 10% (v/v) neutral buffered formalin. Testes, epididymis, eyes and optic nerve were fixed in Davidson’s solution, and then embedded in paraffin, sectioned, and stained with hematoxylin and eosin (H&E). Histopathological examinations were performed. The following tissues/organs were collected for histopathology: brain, spinal cord (cervical, thoracic, and lumbar segments), pituitary gland, thyroid, parathyroid glands, heart, aorta, trachea, lungs, salivary glands (submandibular glands), pancreas, esophagus, stomach, duodenum, jejunum, ileum, cecum, colon, rectum, liver, harderian gland, kidneys, bladder, testes, epididymides, prostate, seminal vesicle, ovaries, oviduct, uterus, cervix, vagina, sciatic nerve, skeletal muscle, adrenal glands, spleen, thymus, mesenteric lymph node, submandibular lymph node, bone marrow (sternum), skin, mammary glands, eyes, optic nerve, bone (femur).

### Genotoxicity studies

#### Bacterial reverse mutation assay (Ames test)

The Ames test was carried out by the method of plate with incorporation, and the *Salmonella typhimurium* (TA97a, TA98, TA100, TA102, and TA1535) was purchased from Moltox Molecular Toxicology Inc. (Boone, NC, USA). According to the results of a preliminary toxicity test, the minimum precipitation concentration of 2000 μg/plate was determined to be the maximum concentration for the formal test. Five doses, including 2000, 800, 320, 128, 51.2 μg/plate were tested with and without an exogenous metabolic activation system (S9). The negative control (DMSO) and positive control groups and treated groups were prepared concurrently. Positive control concentrations were selected, including 2-amino fluorene (2-AF, 10 μg/plate) for TA97a, TA98, TA100, TA102 and TA1535 with S9, sodium azide (NaN3, 1.5 μg/plate) for TA100 and TA1535 without S9, Dexon (50 μg/plate) for TA97a, TA98 and TA102 without S9. Triplicate plates for each concentration were prepared. Briefly, aliquots (0.1 mL) of JointAlive^®^ (or negative control or positive control) was added into 2.0 mL of top agar (held at 45°C) along with a 0.1 mL of bacterial suspension, followed by the addition of 0.5 mL of either the S9 mix or 0.2 M phosphate buffer (pH 7.4). The mixture was poured onto a minimal medium agar plate after vortexing and incubated at 37°C for 48 h. Following incubation, the revertant colonies were counted.

#### In vitro mammalian chromosome aberration test

A chromosome aberration test was performed on Chinese Hamster Lung fibroblasts (CHL) cells. Cells were purchased from Council cell bank of Typical Culture Preservation, Chinese Academy of Sciences. To determine the maximum concentration for the test groups, a dose-range finding assay was performed at 500, 200, 120, 32, 12.8 and 5.12 μg/mL. Cells were exposed to the test chemical without metabolic activation (S9) for 3 h or 24 h, all cells died at the highest concentration of 500 μg/mL, about 80% cytotoxicity was observed at 200 μg/mL and less than 50% cytotoxicity at 120 μg/mL. Thus, 150 μg/mL was selected as the highest exposure level and 75, 37.5, 18.75, 9.375 μg/mL were selected as the lower serial concentrations. The experiment was conducted under three conditions, cells were treated with the test substances in duplicate for 3 hours with and without metabolic activation and 24 hours without metabolic activation. Each treatment included negative and positive controls. Cyclophosphamide (CP, 5 μg/mL for 3 h) and ethyl methanesulfonate (EMS, 1000 μg/mL for 3 h and 500 μg/mL for 24 h) were positive controls with and without metabolic activation, respectively. DMSO was the negative control and the solvent. The cells treated for 3 h were further incubated for 21 h after changing to normal media. Cell cultures are treated with 0.1 μg/mL Colcemid for two hours prior to harvesting. After incubation, the cells were collected in 7.5 mM KCl, then fixed with Methanol glacial acetic acid solution (Methanol: glacial acetic acid = 3: 1) and stained with giemsa solution. 300 metaphase cells (Positive control could analyze 100 metaphase cells) were analysed microscopically for the presence of chromatid-type and chromosome-type aberrations.

#### In vivo mammalian (mice) micronucleus test

The results of a preliminary test showed that the mice treated orally with JointAlive^®^ at a dose of 2 and 1 g/kg for 2 days showed no obvious toxicity symptoms. Therefore, the limited dose of 2 g/kg was taken as the highest dose in this experiment, and the middle and low doses were set as 1 g/kg and 0.5 g/kg respectively. In this experiment, animals were divided into five groups (5 males and 5 females for each group) by body weight in a snake-shaped way. Mice were treated (10 mL/kg) with either pure water (control) or JointAlive^®^ of 2, 1, and 0.5 g/kg by oral gavage twice within a 24-hour interval. Cyclophosmide (0.03 g/kg bw) was used as positive control and intraperitoneally injected once. The mice were euthanized by CO_2_ about 24 hours after the final drug administration. Femurs were aseptically removed, then bone marrow cells were collected by perfusion of the femora with phosphate buffer solution (PBS). The cells were smeared on slides, the slides were fixed with methanol and stained with Giemsa. For each animal, at least four thousand polychromatic erythrocytes (PCE) were counted to determine the number of micronucleated polychromatic erythrocytes (MNPCE). To identify any cytotoxicity associated with the test substance, the number of PCE was counted from at least 500 red blood cells in each animal and the ratio of PCE/RBC was calculated.

### Statistical analysis

The experimental data were statistically analyzed using SPSS 19.0 software (SPSS Inc., Chicago, USA). Levene’s test was used to assess the homogeneity of variances. A one-way analysis of variance (ANOVA) was conducted when the variances were homogeneous. Dunnett’s test was performed to assess the significant variance. The histopathological findings were evaluated using Fisher’s exact probability test. Mean ± standard deviation of all values was calculated. A p-value < 0.05 was considered as statistically significant.

## Results

### Acute oral toxicity study

During the experiment, no death was observed in all the groups and no obvious abnormality was found in the control group. In the 20 g/kg bw/day group, loose stool and salivation were observed in all animals on the day of administration, and loose stool was still observed in some animals on the second day after dosing. Perianal filth was still observed in some animals on the fourth day after administration. As shown in Table 1, body weights were measured before administration and on the 2^nd^, 7^th^ and 14^th^ days after administration. The weight of the male animals in the 20 g/kg bw/day group was found to decrease on the 2^nd^ and 7^th^ days, while that of the female animals were found to decrease on the 2^nd^ day and increased on the 14^th^ day, which were statistically different from that of the control group (p<0.05). The reason for the weight loss of rats may be caused by decreased food intake which is associated with the large volume of test material administered. The animals were dissected on the 15^th^ day after drug administration, and no obvious morphological abnormalities were found in the gross necropsy of rats in each group.

**Table 1:** Body weight changes in male and female rats treated with JointAlive^®^ in acute oral toxicity study.

Sex	Dose	Body weights (g)			
		Before dosing	Day 2	Day 7	Day 14
Male	0 g/kg	190.2±4.5	210.8±5.2	263.1±15.2	336.2±17.7
	20 g/kg	188.2±2.9	198.2±6.6*	249.1±13.6*	330.8±19.6
Female	0 g/kg	181.4±2.4	195.6±4.0	219.0±7.5	245.8±9.9
	20 g/kg	179.1±3.9	187.9±5.8*	222.0±6.4	260.6±10.5*

Each value showed the mean ± standard deviation; n = 10 animals/sex group for treatment groups. Significant differences at

*p<0.05 compared with control.

### 13-week repeated oral toxicity study

#### Clinical observations, body weight, food consumption and ophthalmic examination

**Fig 1:**
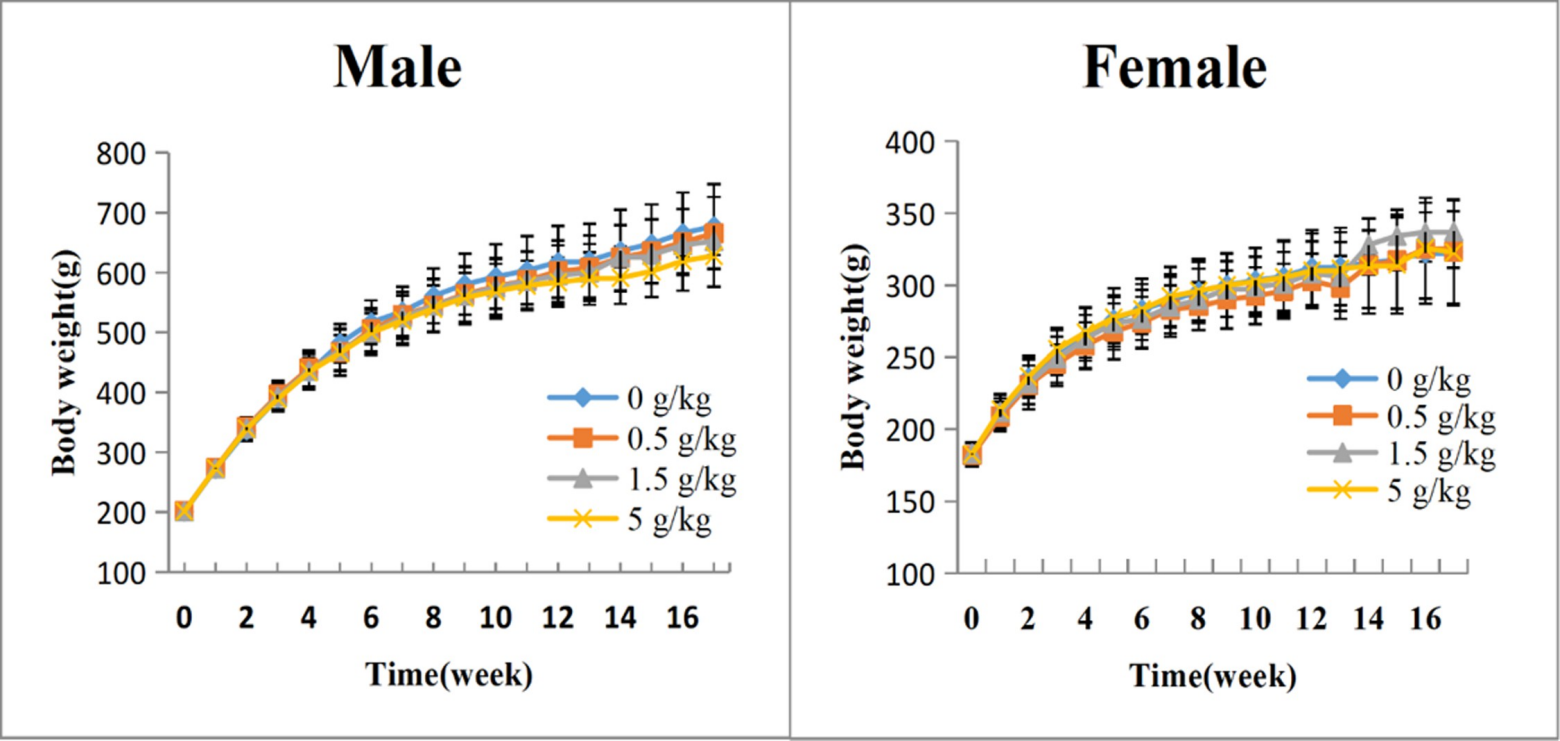
The change of body weight in male and female rats treated with JointAlive^®^ in 13-week subchronic toxicity study. Each value shows the mean ± standard deviation; n = 15 animals/sex group for treatment groups (Week 1–13); n = 5 animals/sex group for recovery groups (Week 14–17).

During the study period, no death was observed in all the groups. The animals in all groups showed no obvious abnormalities. There were no statistically significant differences in body weight between JointAlive^®^-treated groups and control group (Fig 1). In the high-dose group (5 g/kg), a significant decrease in food consumption was observed in males at week 9, week 11 and week 12 and in females at week 9, week 10 and week 11 compared with the control group (Table 2). No significant change was observed in food consumption at other detection time points between JointAlive^®^-treated groups and control group. The results of ophthalmic examination showed that no abnormal findings were obtained in animals throughout the administration period and recovery period.

**Table 2:** Food consumption by male and female rats treated with JointAlive^®^ in 13-week subchronic toxicity study.

Test time	Male				Female			
	0 g/kg	0.5 g/kg	1.5 g/kg	5 g/kg	0 g/kg	0.5 g/kg	1.5 g/kg	5 g/kg
Week 1	27.5±1.1	27.9±1.0	27.9±1.0	26.8±1.2	18.5±1.0	18.4±0.9	18.5±2.0	18.1±1.2
Week 2	32.4±4.3	33.5±3.1	32.3±1.6	32.6±3.1	21.2±1.1	20.7±1.4	20.7±2.4	21.1±1.9
Week 3	33.9±1.0	34.6±1.9	33.9±1.5	34.4±3.0	20.9±1.3	20.6±1.3	21.0±2.6	20.6±1.8
Week 4	29.3±1.5	29.2±1.6	28.4±1.9	29.1±2.6	18.1±0.9	17.7±1.2	18.4±1.3	17.6±1.0
Week 5	36.4±1.9	35.0±1.7	33.6±2.5	34.1±2.7	22.0±0.8	19.4±2.9	21.6±2.1	21.3±1.0
Week 6	35.9±1.9	35.8±2.6	34.3±2.2	34.2±2.4	21.9±1.2	20.8±0.8	21.6±1.8	20.9±1.3
Week 7	35.1±2.0	35.9±2.0	33.1±2.0	31.4±4.0	21.9±1.0	21.0±0.9	21.8±2.0	20.6±0.8
Week 8	33.6±2.2	33.4±2.5	32.6±1.7	30.2±2.3	21.3±0.8	19.8±1.0	21.4±1.3	19.8±1.2
Week 9	33.9±2.1	33.9±2.6	32.1±2.0	29.7±1.8*	20.5±1.4	19.8±1.2	20.6±1.6	17.8±1.3*
Week 10	32.5±2.0	32.7±1.6	30.5±2.6	29.0±2.2	20.5±1.0	19.8±1.0	20.1±0.9	18.4±1.2*
Week 11	32.8±2.1	34.0±2.1	32.4±2.1	28.3±2.4*	21.2±0.8	20.2±0.6	21.5±1.5	18.7±0.8*
Week 12	32.5±2.5	34.0±2.3	30.1±1.5	28.1±1.8*	21.0±1.2	20.2±1.0	20.8±1.9	18.9±1.0
Week 13	32.2±2.8	34.0±2.4	31.2±1.9	29.4±2.2	20.7±1.2	20.1±1.4	20.5±2.0	19.5±1.3
Week 14	31.2±2.1	32.1±3.5	31.0±1.9	30.7±2.0	18.3±2.0	19.7±0.1	19.8±1.5	19.8±0.7
Week 15	33.0±3.2	33.4±4.2	30.6±1.2	31.5±0.1	19.0±0.8	20.7±0.1	20.7±1.5	20.8±1.1
Week 16	34.8±2.8	34.0±3.5	32.1±2.1	31.3±4.5	20.1±1.0	22.3±0.1	20.7±1.8	21.5±1.6
Week 17	34.9±2.8	35.0±3.4	31.7±2.4	31±4.1	19.1±0.8	21.7±0.8	20.8±0.7	20.9±1.8

Each value showed the mean ± standard deviation; n = 15 animals/sex group for treatment groups (Week 1–13); n = 5 animals/sex group for recovery groups (Week 14–17). Significant differences at

*p<0.05 compared with control.

#### Hematology and clinical biochemistry

Hematology parameters are presented in Table 3. At the end of dosing, relative to the control, EO% was significantly increased in the females of middle dose group with statistical difference (p < 0.05) (Table 3). Serum biochemical parameters are shown in Table 4. Males of the high dose group showed an increase in GLU and K^+^, and a decrease in TP, GLOB and Na^+^, as compared with the control group with statistical difference (p < 0.05) (Table 4). In females, the high dose group showed an increase in CHOL, an increase in K^+^ was observed in the middle and high dose groups, with statistical difference (p < 0.05). At the end of recovery period, females of the low dose group showed an increase in GGT, and a decrease in TP and GLOB, compared with the control group with statistical difference (p < 0.05) (Table 4). The changes of the above indexes have no obvious time-dependent or dose-dependent relationship, or the changes are small or incidental, so none of them were considered to have toxicological significance.

**Table 3:** Hematological parameters for rats in 13-week subchronic toxicity study.

Dose (g/kg)	Male								Female							
	Treatment				Recovery				Treatment				Recovery			
	0	0.5	1.5	5	0	0.5	1.5	5	0	0.5	1.5	5	0	0.5	1.5	5
WBC (10^9^/L)	6.64±1.08	6.62±2.37	6.54±1.73	7.33±1.82	6.90±3.48	5.94±1.23	5.38±0.88	5.33±1.46	4.16±1.14	3.77±1.45	3.02±0.71	3.24±1.07	4.42±2.07	4.30±0.40	2.34±0.65	3.18±0.88
NEUT (10^9^/L)	1.06±0.47	1.01±0.29	0.95±0.37	0.99±0.21	1.44±0.98	1.19±0.43	1.11±0.18	1.32±0.56	0.63±0.38	0.40±0.16	0.44±0.15	0.46±0.23	0.48±0.28	0.84±0.22	0.40±0.10	0.66±0.45
LYMPH (10^9^/L)	5.42±1.25	5.49±2.27	5.44±1.83	6.17±1.87	5.27±2.56	4.59±0.94	4.12±0.79	3.87±1.20	3.47±1.01	3.30±1.29	2.51±0.61	2.71±0.88	3.89±2.02	3.35±0.56	1.87±0.53	2.46±0.54
MONO (10^9^/L)	0.07±0.04	0.06±0.03	0.06±0.03	0.06±0.03	0.07±0.08	0.06±0.01	0.05±0.03	0.05±0.02	0.04±0.02	0.03±0.03	0.04±0.02	0.03±0.01	0.02±0.02	0.03±0.03	0.02±0.02	0.03±0.03
EO (10^9^/L)	0.08±0.03	0.07±0.03	0.09±0.04	0.11±0.03	0.12±0.04	0.08±0.02	0.10±0.03	0.09±0.03	0.02±0.01	0.04±0.02	0.04±0.02	0.04±0.03	0.03±0.01	0.08±0.05	0.04±0.01	0.03±0.01
BASO (10^9^/L)	0.00±0.00	0.00±0.00	0.00±0.00	0.00±0.00	0.00±0.00	0.00±0.00	0.00±0.00	0.00±0.00	0.00±0.00	0.00±0.00	0.00±0.00	0.00±0.00	0.00±0.00	0.00±0.00	0.00±0.00	0.00±0.00
NEUT%	16.54±9.09	16.56±5.85	15.47±7.49	14.48±5.98	21.08±9.33	19.90±5.17	20.72±2.52	24.56±6.92	14.98±8.45	10.62±1.27	14.37±3.53	14.05±4.23	11.76±5.59	19.82±5.78	17.30±1.21	19.48±9.07
LYMPH%	81.05±9.49	81.43±6.42	82.15±8.37	83.09±6.29	76.20±9.24	77.56±5.03	76.40±3.45	72.50±6.59	83.62±8.89	87.40±1.17	83.19±3.88	83.89±4.19	86.88±6.04	77.58±7.17	79.96±1.40	78.52±9.73
MONO%	1.14±0.60	0.89±0.48	0.94±0.55	0.90±0.46	0.90±0.46	1.10±0.39	1.02±0.61	0.98±0.31	0.90±0.51	0.87±0.51	1.15±0.66	0.82±0.43	0.46±0.54	0.78±0.90	0.94±0.62	0.88±0.75
EO%	1.27±0.51	1.12±0.80	1.43±0.89	1.53±0.65	1.78±0.33	1.44±0.27	1.86±0.84	1.96±0.97	0.50±0.12	1.11±0.86	1.29±0.60*	1.24±0.60	0.90±0.62	1.82±1.33	1.80±0.41	1.12±0.34
BASO%	0.00±0.00	0.00±0.00	0.01±0.03	0.00±0.00	0.04±0.09	0.00±0.00	0.00±0.00	0.00±0.00	0.00±0.00	0.00±0.00	0.00±0.00	0.00±0.00	0.00±0.00	0.00±0.00	0.00±0.00	0.00±0.00
RBC (10^12^/L)	8.90±0.23	8.91±0.39	8.84±0.18	8.75±0.28	8.73±0.28	9.04±0.45	8.78±0.48	8.56±0.15	7.99±0.30	7.75±0.40	7.92±0.39	7.91±0.38	7.93±0.23	8.06±0.15	7.90±0.29	7.86±0.22
HGB	157.9±5.2	160.4±4.5	159.9±5.5	158.6±6.7	153.0±10.0	159.8±1.3	155.2±7.2	158.2±3.4	149.2±6.3	146.0±7.0	150.0±3.8	151.805.3	143.6±3.7	149.6±4.8	148.2±4.0	148.8±1.9
HCT (%)	44.22±1.56	44.93±0.99	44.81±1.00	44.14±1.79	42.84±2.53	44.58±0.88	43.18±1.84	44.12±1.20	41.63±1.86	40.75±1.76	41.64±1.12	42.46±1.42	40.76±0.95	42.38±1.36	41.78±1.14	41.96±0.78
MCV (fL)	49.70±1.87	50.48±2.14	50.74±1.34	50.45±1.56	49.04±1.35	49.44±2.77	49.22±1.48	51.52±1.36	52.09±1.90	52.62±1.65	52.68±1.85	53.74±1.95	51.46±1.47	52.60±1.14	52.92±1.95	53.46±1.83
MCH (pg)	17.75±0.61	18.03±0.60	18.10±0.51	18.13±0.56	17.48±0.66	17.74±1.01	17.66±0.33	18.50±0.36	18.68±0.66	18.85±0.56	18.97±0.63	19.21±0.44	18.14±0.55	18.56±0.38	18.76±0.65	18.94±0.47
MCHC (g/L)	357.1±5.3	357.1±6.2	356.9±6.7	359.3±3.3	357.0±4.9	358.6±7.5	359.6±4.6	358.8±4.0	358.6±3.8	358.2±4.8	360.3±6.5	357.6±5.4	352.4±2.3	352.8±2.3	354.8±1.3	354.4±4.2
PLT (10^9^/L)	1237±175	1191±138	1100±93	1157±75	1260±108	1232±204	1126±81	12180±62	1087±144	1112±179	1143±92	1089±85	1186±109	1242±1981	1116±79	1024±89
RET%	2.81±0.66	2.96±0.41	2.61±0.51	2.74±0.39	2.93±0.66	3.06±0.51	2.55±0.26	3.00±0.35	2.77±0.42	2.48±0.37	2.81±0.51	2.98±0.72	2.37±0.21	2.38±0.35	2.51±0.37	2.27±0.72
RET (10^9^/L)	250.5±62.5	263.1±33.7	230.0±41.7	239.6±32.4	254.9±52.3	277.±51.8	223.0±11.3	256.8±29.7	221.6±33.3	191.8±25.1	221.2±33.6	234.0±50.2	187.9±13.8	191.3±27.3	197.9±24.9	178.3±57.1
PT (Sec)	16.15±0.52	16.14±0.53	16.40±0.58	16.77±0.71	15.92±1.04	16.08±0.47	16.42±0.57	16.12±0.76	15.72±0.47	16.07±0.82	15.77±0.58	16.12±0.70	16.02±0.45	16.16±0.59	15.52±0.63	15.52±0.41
APTT (Sec)	20.23±2.88	19.37±1.12	20.16±1.27	18.87±1.60	19.38±2.61	19.90±1.65	19.64±0.91	19.62±2.09	18.83±1.40	18.46±1.77	17.43±1.70	18.02±1.72	19.58±1.01	19.04±1.00	18.68±3.03	18.20±2.14
FIB (g/L)	3.25±0.28	3.15±0.39	2.95±0.15	2.95±0.21	3.17±0.44	3.20±0.33	3.01±0.08	3.20±0.48	2.19±0.15	2.26±0.21	2.21±0.20	2.30±0.24	2.25±0.25	2.39±0.24	2.23±0.14	2.17±0.24

Each value showed the mean ± standard deviation. n = 10 animals/sex group for treatment groups; n = 5 animals/sex group for recovery groups. Significant differences at *p < 0.05 compared with control.

**Table 4:** Biochemical analyses of rats in 13-week subchronic toxicity study.

Dose (g/kg)	Male								Female							
	Treatment				Recovery				Treatment				Recovery			
	0	0.5	1.5	5	0	0.5	1.5	5	0	0.5	1.5	5	0	0.5	1.5	5
TBIL (μmol/L)	1.65±0.31	1.56±0.32	1.76±0.20	1.79±0.31	2.42±0.40	2.44±0.30	2.42±0.15	2.36±0.30	2.27±0.43	2.13±0.44	2.19±0.24	2.13±0.39	3.16±0.81	3.18±0.44	3.04±0.47	3.48±0.50
TP (g/L)	68.33±3.92	66.33±4.61	64.64±2.46	63.67±2.17*	67.76±1.47	64.98±3.63	65.16±1.96	65.62±4.34	70.51±2.56	69.77±4.55	71.38±2.26	69.85±5.00	77.40±4.57	69.74±4.29*	71.94±3.97	74.22±2.87
ALB (g/L)	36.48±2.04	35.68±2.25	34.78±0.88	34.68±1.06	35.34±0.88	34.64±1.85	34.46±0.60	35.04±1.65	39.86±1.34	39.32±2.60	40.26±2.00	39.19±2.97	42.60±3.23	38.74±2.67	39.64±2.44	41.82±1.64
GLOB (g/L)	31.85±1.96	30.65±2.58	29.86±1.64	28.99±1.35*	32.42±1.26	30.34±2.12	30.70±1.61	30.58±2.90	30.65±1.69	30.45±2.17	31.12±0.93	30.66±2.13	34.80±1.55	31.00±2.16*	32.30±1.62	32.40±1.48
A/G	1.13±0.05	1.17±0.07	1.19±0.06	1.19±0.06	1.08±0.04	1.16±0.05	1.14±0.05	1.16±0.05	1.30±0.07	1.29±0.07	1.31±0.07	1.28±0.04	1.22±0.08	1.26±0.09	1.22±0.04	1.32±0.04
ALT (U/L)	39.30±5.44	36.00±5.08	36.00±12.60	37.10±13.34	45.40±12.10	37.80±5.45	45.00±18.40	35.80±5.45	43.40±18.62	44.90±30.20	36.60±15.19	26.60±4.60	34.60±6.58	41.80±20.73	68.40±56.63	37.60±7.77
AST (U/L)	120.0±17.6	130.0±31.7	127.6±32.5	130.9±21.9	143.8±33.0	150.0±37.7	133.6±31.1	110.0±19.2	136.3±47.7	134.3±51.5	111.4±30.9	117.2±13.6	109.8±20.1	136.81±36.7	140.4±72.1	86.6±12.3
ALP (U/L)	81.10±14.00	77.00±12.92	84.80±10.96	87.30±26.81	69.20±8.76	88.60±21.72	72.00±10.12	74.20±14.77	37.50±8.61	37.80±6.34	38.60±9.80	35.70±9.86	38.40±7.09	33.80±7.05	39.40±5.77	35.40±7.89
GGT (U/L)	0.50±0.71	0.60±0.52	0.60±0.70	0.90±0.74	0.60±0.89	0.20±0.45	0.80±0.45	0.40±0.89	1.00±0.47	0.80±0.79	1.00±0.67	1.10±0.74	0.00±0.00	1.00±0.00*	0.60±0.55	0.80±0.84
CK (U/L)	429.4±83.6	449.6±148.7	453.4±190.6	443.4±66.8	392.4±125.7	420.2±154.7	357.2±117.6	290.8±132.1	345.7±129.0	363.8±155.0	325.7±138.8	391.0±102.4	358.6±170.6	326.6±114.2	235.0±45.9	165.8±43.5
UREA (mmol/L)	6.18±0.94	6.13±0.71	6.36±0.75	5.85±0.81	7.22±0.90	6.98±0.73	6.80±0.93	6.95±1.06	8.48±2.62	8.86±2.22	8.33±1.19	6.17±0.74	7.10±1.23	8.00±0.38	7.85±1.48	7.69±1.50
CREA (μmol/L)	25.04±2.04	25.25±3.34	26.85±2.77	26.31±3.89	32.40±5.91	29.12±7.84	27.82±3.30	27.32±4.75	36.25±7.37	37.34±6.62	34.75±8.28	33.07±6.52	30.24±4.15	35.62±2.58	32.46±1.07	33.14±5.46
GLU (mmol/L)	8.14±0.81	9.37±1.38	9.16±1.07	9.78±1.33*	9.64±1.74	9.17±1.30	9.85±2.02	10.06±2.81	7.14±0.72	7.25±0.83	7.39±1.19	7.03±0.83	8.13±0.70	7.78±1.19	8.50±0.66	7.93±0.15
TG (mmol/L)	0.92±0.55	0.77±0.41	0.74±0.62	0.54±0.27	0.71±0.10	0.83±0.70	0.60±0.37	0.61±0.39	0.30±0.09	0.29±0.10	0.28±0.13	0.23±0.06	0.36±0.15	0.37±0.26	0.29±0.12	0.28±0.07
CHOL (mmol/L)	1.84±0.49	1.88±0.66	1.54±0.39	1.45±0.25	1.66±0.44	1.67±0.31	1.42±0.16	1.66±0.37	1.28±0.36	1.67±0.34	1.59±0.34	1.85±0.47*	2.04±0.75	1.37±0.56	1.66±0.28	1.58±0.27
K^+^ (mmol/L)	4.74±0.21	4.87±0.23	4.89±0.11	5.01±0.24*	4.74±0.27	4.62±0.30	4.58±0.23	4.60±0.22	3.96±0.30	4.20±0.14	4.31±0.18*	4.40±0.23*	4.14±0.26	4.18±0.16	4.26±0.22	4.28±0.27
Na^+^ (mmol/L)	142.1±1.1	141.8±0.8	141.70±1.25	140.3±1.7*	145.6±0.6	145.6±1.5	146.2±1.3	145.6±1.1	142.10±0.74	141.80±0.79	141.90±1.29	141.20±1.14	144.60±1.14	146.00±1.00	144.60±1.14	145.40±0.55
Cl^-^ (mmol/L)	104.30±1.89	104.30±2.58	104.10±1.60	102.80±2.04	107.00±0.71	107.40±1.82	106.80±0.84	107.80±2.59	107.80±1.62	107.90±0.88	106.40±0.97	106.20±1.75	109.20±1.30	109.60±1.52	109.20±0.45	109.60±0.89

Each value showed the mean ± standard deviation. n = 10 animals/sex group for treatment groups; n = 5 animals/sex group for recovery groups. Significant differences at

*p < 0.05 compared with control.

#### Urinalysis

There was no significant change in any of the urinalysis measurements after 13 weeks, except for SG and pH: Increased SG in male animals and decreased pH in female animals were observed in the middle-dose (1.5 g/kg) group, and increased SG and decreased pH in male and female animals were observed in the high-dose (5 g/kg) group, showing significant differences (p < 0.05) from those in the negative control group, which recovered to normal at the end of recovery (Table 5).

**Table 5:** Effects of JointAlive^®^ on urinalysis of rats in 13-week subchronic toxicity.

Male	Treatment				Recovery			
	0 g/kg	0.5 g/kg	1.5 g/kg	5 g/kg	0 g/kg	0.5 g/kg	1.5 g/kg	5 g/kg
pH	8.5±0.2	8.5±0.0	8.0±0.6	7.7±0.9*	8.1±0.2	8.1±0.2	8.1±0.7	8.0±0.4
SG	1.010±0.004	1.018±0.009	1.023±0.008*	1.028±0.011*	1.015±0.006	1.014±0.006	1.016±0.005	1.012±0.006
Female								
pH	8.5±0.0	8.1±0.4	7.4±0.6*	8.0±0.4*	7.6±0.5	7.8±0.3	8.1±0.7	8.3±0.3
SG	1.011±0.005	1.009±0.005	1.010±0.005	1.028±0.009*	1.008±0.005	1.004±0.001	1.006±0.002	1.007±0.001

Each value showed the mean ± standard deviation. n = 10 animals/sex group for treatment groups; n = 5 animals/sex group for recovery groups. Significant differences at *p < 0.05 compared with control.

#### Organ weights, macroscopic and histopathological observations

**Fig 2:**
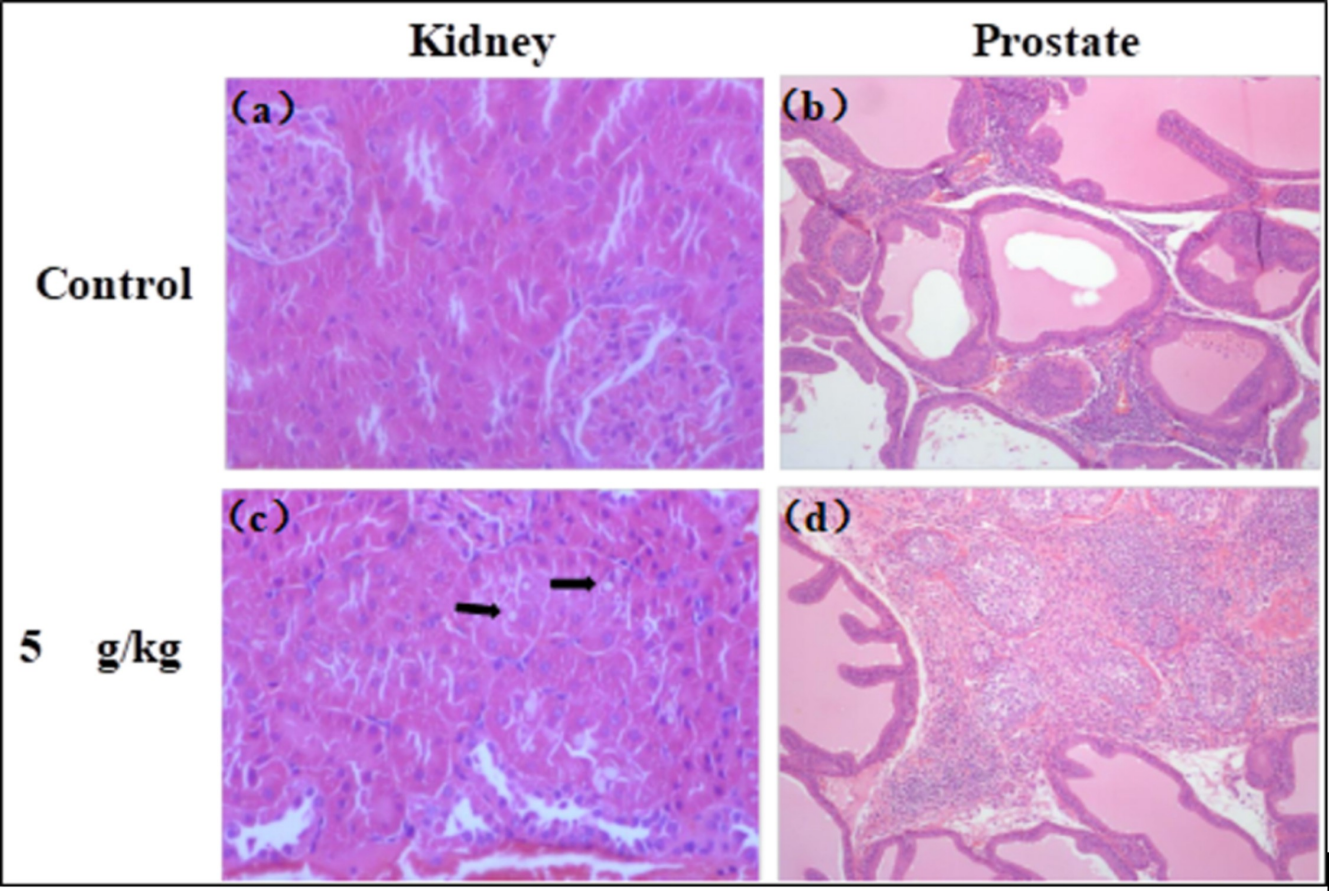
Representative histological findings in JointAlive^®^-treated SD rats. All tissues were stained with H&E. (a) Control group, normal kidney in the field of view, HE stained, ×400. (b) Control group, prostate, infiltration of focal inflammatory cells, ×100. (c) High-dose group, kidney, multifocal hyaline droplet accumulation (black arrows) in renal tubules, ×400. (d) High-dose group, prostate, chronic inflammation, ×100.

Absolute organ weights are listed in Table 6. No significant difference was found in absolute organ weight in either males or females between the JointAlive^®^ treated group and control group. For the weights relative to body weight, males of the high dose group at the end of dosing had a significant increase by 13% in kidney as compared with the control group with a statistically significant difference (p < 0.05) (Table 6). No macroscopic anomalies related to the test substance were observed in any of the treatment groups. Histopathological examination showed multifocal hyaline droplet accumulation in renal tubules in 5/10 males (mild to moderate) at the end of dosing and 1/5 males (mild) at the end of the recovery period in the high-dose group. At the end of administration period, chronic inflammation of prostate was observed in 2/10 males (mild) of the control group, 0/10 males of the low dose group, 2/10 males (mild to moderate) of the middle dose group, and 7/10 males (mild to moderate) of the high dose group (Fig 2). Chronic inflammation of prostate was observed in 2/5 males (minimal and slight) of the control group, 0/5 males of the low and middle dose group, 1/5 males (slight) of the high dose group after the recovery period (S1 Table).

**Table 6:** Effects of JointAlive^®^ on organ weights of rats in 13-week subchronic toxicity study.

Dose (g/kg)	Male								Female							
	Absolute (g)				Relative (% body weight)				Absolute (g)				Relative (% body weight)			
	0	0.5	1.5	5	0	0.5	1.5	5	0	0.5	1.5	5	0	0.5	1.5	5
Brain	2.204±0.138	2.198±0.067	2.185±0.060	2.211±0.076	0.375±0.035	0.385±0.039	0.390±0.041	0.392±0.037	1.990±0.093	2.004±0.126	2.012±0.082	1.988±0.086	0.670±0.074	0.713±0.066	0.710±0.070	0.684±0.056
Heart	1.695±0.159	1.740±0.162	1.618±0.150	1.678±0.143	0.287±0.013	0.303±0.020	0.287±0.019	0.296±0.018	0.993±0.069	0.945±0.086	0.975±0.090	0.955±0.083	0.333±0.020	0.335±0.026	0.342±0.025	0.327±0.017
Liver	16.395±3.081	16.413±3.075	14.903±2.185	16.186±1.401	2.750±0.301	2.831±0.277	2.629±0.161	2.851±0.193	7.794±0.408	7.624±1.113	7.660±0.797	8.310±1.022	2.614±0.171	2.690±0.214	2.682±0.130	2.838±0.220
Spleen	0.911±0.119	0.956±0.116	0.866±0.190	0.887±0.123	0.154±0.011	0.166±0.014	0.154±0.031	0.156±0.019	0.570±0.091	0.527±0.064	0.545±0.045	0.571±0.091	0.191±0.027	0.187±0.022	0.192±0.020	0.195±0.022
Kidney	3.682±0.472	3.696±0.458	3.505±0.462	3.984±0.275	0.620±0.033	0.642±0.057	0.619±0.035	0.702±0.043*	2.014±0.068	1.893±0.177	1.942±0.216	2.107±0.095	0.677±0.050	0.671±0.040	0.681±0.057	0.724±0.048
Thymus	0.263±0.051	0.284±0.051	0.250±0.053	0.293±0.031	0.045±0.011	0.049±0.006	0.045±0.009	0.052±0.007	0.201±0.071	0.206±0.046	0.224±0.046	0.233±0.042	0.067±0.021	0.073±0.015	0.079±0.017	0.080±0.011
Adrenal gl.	0.072±0.008	0.074±0.012	0.074±0.013	0.072±0.010	0.012±0.001	0.013±0.002	0.013±0.002	0.013±0.002	0.085±0.008	0.072±0.010*	0.080±0.006	0.083±0.009	0.029±0.005	0.026±0.003	0.028±0.002	0.028±0.003
Testis	3.643±0.421	3.608±0.319	3.485±0.293	3.504±0.313	0.617±0.063	0.631±0.072	0.620±0.051	0.617±0.050	0.676±0.118	0.569±0.086	0.656±0.119	0.641±0.122	0.226±0.037	0.203±0.036	0.232±0.051	0.222±0.050
Epididymis	1.448±0.209	1.482±0.116	1.451±0.130	1.448±0.090	0.245±0.027	0.259±0.031	0.258±0.023	0.256±0.021	0.094±0.018	0.085±0.018	0.095±0.016	0.095±0.018	0.032±0.008	0.031±0.007	0.033±0.005	0.032±0.006

Each value showed the mean ± standard deviation. n = 10 animals/sex group for treatment groups; n = 5 animals/sex group for recovery groups. Significant differences at *p < 0.05 compared with control.

### Genotoxicity studies

#### Bacterial reverse mutation assay

No cytotoxicity and positive mutagenic responses were observed at all exposure levels of JointAlive^®^ (2000, 800, 320, 128, 51.2 μg/plate) in any of the tester strains (TA97a, TA98, TA100, TA102 and TA1535) with and without S9. The number of revertant colonies in JointAlive^®^ treatment groups were not increased greater than 2-fold those of the control in any strain with and without S9. In contrast, there was large fold increase in the number of revertant colonies in positive control groups compared with the controls. The results of negative and positive control were within the range of historical control values (Table 7).

**Table 7:** Bacterial reverse mutation test on JointAlive^®^ using various Salmonella typhimurium strains.

	Dose (μg/plate)	S9	Colonies/plate				
			TA97a	TA98	TA100	TA102	TA1535
Control	0	+	109.3±3.2	25.3±3.8	131.3±24.6	350.3±19.7	8.7±1.5
JointAlive^®^	51.2	+	136.7±11.9	28.0±2.0	147.0±2.6	310.0±41.2	9.3±5.1
	128	+	133.0±18.2	25.0±5.2	127.3±11.5	319.7±22.8	9.0±3.6
	320	+	118.0±25.5	24.0±4.6	141.7±8.0	342.7±24.1	9.3±2.1
	800	+	124.7±29.4	26.3±3.1	130.0±5.2	323.7±28.9	8.0±2.6
	2000	+	146.0±2.6	25.7±3.8	136.7±22.0	299.3±25.0	8.0±2.0
Positive control	10 (2-AF)	+	1375.7±128.2	1280.3±113.4	1266.7±183.9	1606.7±188.7	187.7±69.0
Control	0	-	120.3±10.7	28.3±4.0	120.7±25.8	309.7±15.8	10.0±1.0
JointAlive^®^	51.2	-	123.0±30.3	28.3±4.0	127.7±3.1	302.3±13.3	7.7±2.9
	128	-	126.3±3.5	31.0±5.0	113.7±7.1	306.7±17.4	10.7±1.5
	320	-	120.3±9.3	25.7±2.1	127.7±30.1	301.3±12.3	8.0±1.7
	800	-	121.3±27.9	19.0±2.0	121.7±15.0	300.3±23.7	7.7±0.6
	2000	-	140.3±10.0	26.0±1.7	126.7±17.4	311.3±17.4	7.7±3.1
Positive control	1.5 (NaN3)	-			1386.0±141.9		377.0±20.0
	50 (Dexon)		1305.3±261.6	1072.0±189.3		1616.7±210.1	

Each value showed the mean ± standard deviation.

#### Chromosome aberration assay

According to the cytotoxicity results, three concentration (150, 75, 37.5 μg/mL) groups were selected to analyze chromosome aberrations. Table 8 shows that compared to the control, there was no statistically significant increase in the number of metaphase cells with chromosome aberration at all doses of JointAlive^®^ for 3 h or 24 h in the absence or presence of S9. In contrast, the positive controls showed significant increase in the number of cells with chromosome aberration, which verified the validity of the experiments (Table 8).

**Table 8:** In vitro chromosome aberration test in CHL cells subjected to JointAlive^®^.

	Dose (μg/mL)	Treatment time (+/-S9)	n	Aberrant metaphases	Total aberrations	Aberration rate (%)
Control	0	3 h (-S9)	300	2	2	0.7
		24 h (-S9)	300	3	3	1
JointAlive^®^	37.5	3 h (-S9)	300	5	5	1.7
		24 h (-S9)	300	5	5	1.7
	75	3 h (-S9)	300	3	3	1
		24 h (-S9)	300	4	4	1.3
	150	3h (-S9)	300	3	3	1
		24 h (-S9)	300	6	6	2
Positive control	1000 (EMS)	3 h (-S9)	100	14	14	14.0*
	500 (EMS)	24 h (-S9)	100	12	12	12.0*
Control	0	3 h (+S9)	300	3	3	1
JointAlive^®^	37.5	3 h (+S9)	300	2	2	0.7
	75	3 h (+S9)	300	2	2	0.7
	150	3 h (+S9)	300	2	2	0.7
Positive control	5 (CP)	3 h (+S9)	100	13	13	13.0*

Significantly different from the control at

* p < 0.05.

#### In vivo mammalian (mice) micronucleus test

No clinical signs of toxicity or death were observed in any of the JointAlive^®^ treatment groups. The effects of JointAlive^®^ on micronucleus frequency were evaluated at 0.5, 1 and 2 g/kg. There were no statistically significant differences in micronucleus frequency between the control group and JointAlive^®^ treated groups (p > 0.05), the MNPCE frequencies at 4000 PCEs/animal were 0.7‰, 1.0‰, 1.2‰ and 1.0‰ respectively. The PCE/RBC ratios are cytotoxicity indices, these ratios did not significantly differ between the control and JointAlive^®^ treated groups and were 63.9%, 67.2%, 66.6% and 71.4%. This indicates that JointAlive^®^ was not cytotoxic to bone marrow following oral exposure. The micronucleus frequency was significantly increased in positive control group compared with negative control group (p < 0.05), which indicated the validity of the experiment (Table 9).

**Table 9:** In vivo micronucleus test for JointAlive^®^ in ICR mice bone marrow cells.

Group	Dose (g/kg)	Male (n = 5)		Female (n = 5)	
		Micronucleus Frequency (‰)	PCE/RBC (%)	Micronucleus Frequency (‰)	PCE/RBC (%)
Control	0	0.5 ± 0.5	67.5 ± 4.3	0.8 ± 0.3	60.2 ± 14.4
JointAlive^®^	0.5	0.7±0.3	69.9±7.1	1.3±0.8	64.6±2.2
	1	1.1±0.4	68.2±7.5	1.3 ± 0.5	65.1±6.2
	2	1.1±0.3	70.8± 6.4	0.9 ± 0.3	72.0±7.0
Positive control	0.03 (CP)	11.1 ± 1.1*	58.2 ± 9.1	10.4 ± 1.6*	67.1 ± 9.0

Each value showed the mean ± standard deviation. Significantly different from the control at

* p < 0.05.

## Discussion

Traditionally, TCMs have been used to prevent or treat various diseases. Today, their use has increased dramatically around the world. However, the toxicity of many herbs has not been widely studied. People tend to think that TCMs are harmless and have no side effects. At the same time, the safety of some commercially available herbal medicines has recently been questioned due to reports of adverse effects associated with their use, such as severe liver toxicity and even death [25–27]. JointAlive^®^, as a TCM formulation, consists of the standardized extracts of *Epimedium brevicornum* leaves (Epimedium), *Discorea nipponica* rhizomes (Chuanlong Yam), and *Salvia miltiorrhiza* roots and rhizomes (Chinese Salvia). The general toxicity and genotoxicity studies introduced above have preliminarily verified its safety to support further safety investigation and clinical studies in the future.

The present study was conducted to assess the acute and long-term oral toxicity of JointAlive^®^ along with genotoxicity. For the single-dose assay, we observed no animal death throughout the experiment. The acute oral approximately lethal dose (ALD) for JointAlive^®^ was ˃ 20 g/kg bw/day in rats.

After 13 weeks repeated administration, all the animals in the study survived to scheduled autopsy. The clinical observation results showed that there were no obvious abnormalities in animals of all the groups. A significant decrease in food consumption in males may be related to the daily administration of large amounts of the herbal formula, which is consistent with the results of another report on matured hop extract [28]. However, there was no significant difference in animal body weight between the control group and the JointAlive^®^ treated groups, so it is considered to have no toxicological significance. Under the conditions of this study, relative to control group, EO% was significantly increased in the females of middle dose group, males of the high dose group showed an increase in GLU and K^+^, and a decrease in TP, GLOB and Na^+^, in females, an increase in CHOL was observed in the high dose group, an increase in K^+^ was observed in the middle and high dose groups. At the end of recovery period, females of the low dose group showed an increase in GGT, and a decrease in TP and GLOB. The changes of the above indexes have no obvious time-dependent or dose-dependent relationship. All the changes were within facility historical control ranges. Overall, it was concluded that these results have no toxicology significance. Histopathological examination found that in the high dose group the accumulation of multifocal hyaline droplets in renal tubules was observed in 5/10 males (mild to moderate) at the end of dosing and 1/5 males (mild) at the end of recovery period. In addition, the increase in weight relative to body weight of kidney in males of the high-dose group at the end of the administration period may be related to these pathological changes, which is similar to Wang’s research [29]. Hyaline droplets are the accumulated low molecular weight protein, alpha2u globulin, in lysosomes. Accumulation of hyaline droplets in renal tubules result from the imbalance between reabsorption and hydrolysis of renal tubules due to increased filtered protein or decreased catabolism. The hyaline droplet finding in male rats other than female rats is due to that Alpha2u globulin is a major urinary protein found in mature male rats while Alpha2u globulin or a very similar protein is present in female rat urine only at very low levels [30]. According to the Jacobsen’s report, the abnormalities in pH and SG in urine may also be responsible for this [31]. However, this is a symptom that can be recovered in the short term. From a safety assessment perspective, it can be concluded that male rat renal tubule tumors arising as a result of a process involving α2u-g accumulation would not contribute to the weight of evidence that a chemical poses a human carcinogenic hazard [32]. Additional, renal effects induced in male rats by chemicals causing α2u-g accumulation are unlikely to occur in humans [33]. Thus, it is generally accepted that humans are not at risk from this male rat specific finding. Chronic inflammation of prostate was observed in 2/10 males (mild) of the control group, 2/10 males (mild to moderate) of the middle dose group, and 7/10 males (mild to moderate) of the high dose group at the end of the administration period. However, the incidence in the high dose group were significantly higher than that in the control group. Chronic inflammation of prostate is a common spontaneous lesion in SD rats [34]. It cannot be inferred that the prostate inflammation relates to JointAlive^®^. The increased incidence was considered co-incidental as no other related findings were seen. The above pathological changes mostly recovered to be normal at the end of the recovery period. So, it can be speculated that the abnormal symptoms of rats in the high-dose group were related to the intake of Epimedium in JointAlive^®^. JointAlive^®^ genotoxicity was first assessed using Ames test and chromosome aberration analysis in this study, and it was shown that there were no statistically significant differences in the number of reproducible colonies or structures or numerical chromosomal aberrations between the JointAlive^®^ treatment groups and the negative control group. The frequency of PCEs with micronuclei (MNPCEs) in bone marrow cells did not increase at 2 g/kg JointAlive^®^.

## Conclusions

In the present study, acute, subchronic toxicity and genotoxicity tests were conducted to assess the safety of JointAlive^®^. The acute toxicity test indicated that approximate lethal dose in rats is > 20 g/kg bw/day. The 13-week subchronic oral toxicity test established the No Observed Adverse Effect Level (NOAEL) of JointAlive^®^ was considered to be 5 g/kg bw/day for both males and females. Genotoxicity tests showed that JointAlive^®^ did not induce any genotoxic effects. These studies showed a good safety profile for JointAlive^®^. This is the first safety assessment report for JointAlive^®^. Clinical research is essential to ensure its safe application.

## Supporting information

S1 Table Histopathological examination of prostate for JointAlive^®^ of male rats at the end of recovery. (DOCX)

S1 File (DOCX)

S2 File (DOCX)
