# Supplementary material for: Acute, subchronic toxicity and genotoxicity studies of JointAlive, a traditional Chinese medicine formulation for knee osteoarthritis
Source: PLoS One. 2023 Oct 17;18(10):e0292937. doi: 10.1371/journal.pone.0292937 (PMC10581469; doi:10.1371/journal.pone.0292937)
Supplement: S1 Table — (DOCX) [file pone.0292937.s001.docx]

**S1 Table. Histopathological examination of prostate for JointAlive^®^ of male rats at the end of recovery.**

| Multifocal chronic  inflammation | Male | | | |
| --- | --- | --- | --- | --- |
|  | 0 g/kg | 0.5 g/kg | 1.5 g/kg | 5 g/kg |
| ± | 1/5 | 0/5 | 0/5 | 0/5 |
| 1+ | 1/5 | 0/5 | 0/5 | 1/5 |

Minimal = ±; slight = 1+.
