# Supplementary material for: Acute, subchronic toxicity and genotoxicity studies of JointAlive, a traditional Chinese medicine formulation for knee osteoarthritis
Source: PLoS One. 2023 Oct 17;18(10):e0292937. doi: 10.1371/journal.pone.0292937 (PMC10581469; doi:10.1371/journal.pone.0292937)
Supplement: S1 File — (DOCX) [file pone.0292937.s002.docx]

**Single-Dose Toxicity Study of JointAlive in Rats**

1. Individual animal data: body weight

| **Body weight (g)** | | | | | | |
| --- | --- | --- | --- | --- | --- | --- |
| **Dose** | **Gender** | **Animal no.** | **D1** | **D2** | **D7** | **D14** |
| 0 g/kg | ♂ | 1001 | 188.1 | 205.7 | 243.7 | 318.1 |
|  |  | 1002 | 184.3 | 206.0 | 244.1 | 316.3 |
|  |  | 1003 | 186.7 | 203.6 | 247.4 | 322.2 |
|  |  | 1004 | 186.3 | 207.6 | 260.0 | 352.1 |
|  |  | 1005 | 187.4 | 210.2 | 258.5 | 344.6 |
|  |  | 1006 | 191.4 | 213.4 | 263.9 | 316.4 |
|  |  | 1007 | 189.9 | 211.2 | 276.5 | 347.3 |
|  |  | 1008 | 194.9 | 214.7 | 276.0 | 345.9 |
|  |  | 1009 | 194.6 | 215.9 | 271.7 | 331.7 |
|  |  | 1010 | 198.3 | 219.8 | 288.7 | 367.1 |
|  | ♀ | 2011 | 180.1 | 197.8 | 222.8 | 245.0 |
|  |  | 2012 | 181.6 | 200.1 | 220.4 | 245.2 |
|  |  | 2013 | 179.6 | 192.0 | 214.9 | 247.7 |
|  |  | 2014 | 176.1 | 191.2 | 209.8 | 232.9 |
|  |  | 2015 | 183.5 | 199.6 | 231.3 | 264.8 |
|  |  | 2016 | 181.7 | 195.7 | 226.0 | 253.8 |
|  |  | 2017 | 181.9 | 193.0 | 213.4 | 233.1 |
|  |  | 2018 | 182.2 | 201.2 | 225.9 | 254.2 |
|  |  | 2019 | 183.5 | 189.6 | 216.4 | 238.7 |
|  |  | 2020 | 184.1 | 195.6 | 209.1 | 242.6 |
| 20 g/kg | ♂ | 1121 | 187.9 | 197.4 | 243.4 | 314.9 |
|  |  | 1122 | 182.5 | 197.9 | 241.3 | 312.3 |
|  |  | 1123 | 190.7 | 197.7 | 240.3 | 321.5 |
|  |  | 1124 | 186.3 | 197.6 | 241.8 | 331.0 |
|  |  | 1125 | 186.2 | 192.1 | 233.7 | 327.1 |
|  |  | 1126 | 186.9 | 191.7 | 245.4 | 325.1 |
|  |  | 1127 | 188.7 | 198.7 | 248.0 | 317.7 |
|  |  | 1128 | 190.3 | 190.5 | 250.8 | 325.9 |
|  |  | 1129 | 190.4 | 210.6 | 274.4 | 368.6 |
|  |  | 1130 | 192.4 | 208.0 | 272.2 | 364.3 |
|  | ♀ | 2131 | 176.5 | 184.3 | 216.5 | 241.6 |
|  |  | 2132 | 177.6 | 177.5 | 211.1 | 256.0 |
|  |  | 2133 | 173.0 | 187.1 | 214.3 | 257.1 |
|  |  | 2134 | 178.7 | 186.6 | 227.8 | 261.4 |
|  |  | 2135 | 177.4 | 194.8 | 224.1 | 262.4 |
|  |  | 2136 | 180.2 | 183.3 | 224.0 | 260.0 |
|  |  | 2137 | 181.6 | 195.3 | 231.2 | 276.5 |
|  |  | 2138 | 175.9 | 184.9 | 227.7 | 273.8 |
|  |  | 2139 | 184.9 | 194.5 | 220.5 | 249.8 |
|  |  | 2140 | 185.2 | 190.2 | 223.1 | 267.8 |

**Bacterial Reverse Mutation Test of JointAlive**

1. Table 1. Summary of test data for treatment with the *in vitro* metabolic activation system (+S9)

| **Concentration group (μg/plate)** | | **TA97a** | **TA98** | **TA100** | **TA102** | **TA1535** |
| --- | --- | --- | --- | --- | --- | --- |
| Vehicle control (DMSO)  (100 μL/plate) | Mean ± SD | 109.3±3.2 | 25.3±3.8 | 131.3±24.6 | 350.3±19.7 | 8.7±1.5 |
|  | Background lawn | Normal | Normal | Normal | Normal | Normal |
|  |  |  |  |  |  |  |
| Test articles  51.2 | Mean ± SD | 136.7±11.9 | 28.0±2.0 | 147.0±2.6 | 310.0±41.2 | 9.3±5.1 |
|  | Ratio | 1.3 | 1.1 | 1.1 | 0.9 | 1.1 |
|  | Background lawn | Normal | Normal | Normal | Normal | Normal |
| Test articles  128 | Mean ± SD | 133.0±18.2 | 25.0±5.2 | 127.3±11.5 | 319.7±22.8 | 9.0±3.6 |
|  | Ratio | 1.2 | 1.0 | 1.0 | 0.9 | 1.0 |
|  | Background lawn | Normal | Normal | Normal | Normal | Normal |
| Test articles  320 | Mean ± SD | 118.0±25.5 | 24.0±4.6 | 141.7±8.0 | 342.7±24.1 | 9.3±2.1 |
|  | Ratio | 1.1 | 0.9 | 1.1 | 1.0 | 1.1 |
|  | Background lawn | Normal | Normal | Normal | Normal | Normal |
| Test articles  800 | Mean ± SD | 124.7±29.4 | 26.3±3.1 | 130.0±5.2 | 323.7±28.9 | 8.0±2.6 |
|  | Ratio | 1.1 | 1.0 | 1.0 | 0.9 | 0.9 |
|  | Background lawn | Normal | Normal | Normal | Normal | Normal |
| Test articles  2000 | Mean ± SD | 146.0±2.6 | 25.7±3.8 | 136.7±22.0 | 299.3±25.0 | 8.0±2.0 |
|  | Ratio | 1.3 | 1.0 | 1.0 | 0.9 | 0.9 |
|  | Background lawn | Normal | Normal | Normal | Normal | Normal |
| Positive control | Mean ± SD | 1375.7±128.2 | 1280.3±113.4 | 1266.7±183.9 | 1606.7±188.7 | 187.7±69.0 |
|  | Ratio | 12.6 | 50.6 | 9.6 | 4.6 | 21.6 |
|  | Background lawn | Normal | Normal | Normal | Normal | Normal |

Note: Mean = the mean revertant colony count (colonies/plate); SD = standard deviation; ratio = mean of test group/mean of vehicle control group

**Table 2. Summary of test data for treatment without the *in vitro* metabolic activation system (−S9)**

| **Concentration group (μg/plate)** | | **TA97a** | **TA98** | **TA100** | **TA102** | **TA1535** |
| --- | --- | --- | --- | --- | --- | --- |
| Vehicle control (DMSO)  (100 μL/plate) | Mean ± SD | 120.3±10.7 | 28.3±4.0 | 120.7±25.8 | 309.7±15.8 | 10.0±1.0 |
|  | Background lawn | Normal | Normal | Normal | Normal | Normal |
|  |  |  |  |  |  |  |
| Test articles  51.2 | Mean ± SD | 123.0±30.3 | 28.3±4.0 | 127.7±3.1 | 302.3±13.3 | 7.7±2.9 |
|  | Ratio | 1.0 | 1.0 | 1.1 | 1.0 | 0.8 |
|  | Background lawn | Normal | Normal | Normal | Normal | Normal |
| Test articles  128 | Mean ± SD | 126.3±3.5 | 31.0±5.0 | 113.7±7.1 | 306.7±17.4 | 10.7±1.5 |
|  | Ratio | 1.0 | 1.1 | 0.9 | 1.0 | 1.1 |
|  | Background lawn | Normal | Normal | Normal | Normal | Normal |
| Test articles  320 | Mean ± SD | 120.3±9.3 | 25.7±2.1 | 127.7±30.1 | 301.3±12.3 | 8.0±1.7 |
|  | Ratio | 1.0 | 0.9 | 1.1 | 1.0 | 0.8 |
|  | Background lawn | Normal | Normal | Normal | Normal | Normal |
| Test articles  800 | Mean ± SD | 121.3±27.9 | 19.0±2.0 | 121.7±15.0 | 300.3±23.7 | 7.7±0.6 |
|  | Ratio | 1.0 | 0.7 | 1.0 | 1.0 | 0.8 |
|  | Background lawn | Normal | Normal | Normal | Normal | Normal |
| Test articles  2000 | Mean ± SD | 140.3±10.0 | 26.0±1.7 | 126.7±17.4 | 311.3±17.4 | 7.7±3.1 |
|  | Ratio | 1.2 | 0.9 | 1.0 | 1.0 | 0.8 |
|  | Background lawn | Normal | Normal | Normal | Normal | Normal |
| Positive control | Mean ± SD | 1305.3±261.6 | 1072.0±189.3 | 1386.0±141.9 | 1616.7±210.1 | 377.0±20.0 |
|  | Ratio | 10.9 | 37.9 | 11.5 | 5.2 | 37.7 |
|  | Background lawn | Normal | Normal | Normal | Normal | Normal |

Note: Mean = the mean revertant colony count (colonies/plate); SD = standard deviation; ratio = mean of test group/mean of vehicle control group

1. Table 3. Individual data for treatment with the *in vitro* metabolic activation system (+S9)

| **Concentration group**  **(μg/plate)** | **Parallel plate** | **Revertant colony count (colonies/plate)** | | | | |
| --- | --- | --- | --- | --- | --- | --- |
|  |  | **TA97a** | **TA98** | **TA100** | **TA102** | **TA1535** |
| Vehicle control (DMSO)  (100 μL/plate) | 1 | 113 | 28 | 103 | 373 | 9 |
|  | 2 | 107 | 27 | 144 | 337 | 7 |
|  | 3 | 108 | 21 | 147 | 341 | 10 |
| Test articles  51.2 | 1 | 133 | 26 | 144 | 319 | 5 |
|  | 2 | 150 | 30 | 148 | 265 | 8 |
|  | 3 | 127 | 28 | 149 | 346 | 15 |
| Test articles  128 | 1 | 144 | 19 | 139 | 307 | 13 |
|  | 2 | 112 | 28 | 116 | 346 | 6 |
|  | 3 | 143 | 28 | 127 | 306 | 8 |
| Test articles  320 | 1 | 146 | 29 | 141 | 359 | 11 |
|  | 2 | 96 | 23 | 134 | 354 | 10 |
|  | 3 | 112 | 20 | 150 | 315 | 7 |
| Test articles  800 | 1 | 91 | 27 | 133 | 357 | 9 |
|  | 2 | 138 | 23 | 133 | 308 | 5 |
|  | 3 | 145 | 29 | 124 | 306 | 10 |
| Test articles  2000 | 1 | 144 | 30 | 162 | 325 | 8 |
|  | 2 | 149 | 24 | 122 | 275 | 10 |
|  | 3 | 145 | 23 | 126 | 298 | 6 |
| Positive control | 1 | 1523 | 1264 | 1464 | 1441 | 109 |
|  | 2 | 1314 | 1176 | 1236 | 1567 | 238 |
|  | 3 | 1290 | 1401 | 1100 | 1812 | 216 |

1. Table 4. Individual data for treatment without the *in vitro* metabolic activation system (−S9)

| **Concentration group**  **(μg/plate)** | **Parallel plate** | **Revertant colony count (colonies/plate)** | | | | |
| --- | --- | --- | --- | --- | --- | --- |
|  |  | **TA97a** | **TA98** | **TA100** | **TA102** | **TA1535** |
| Vehicle control (DMSO)  (100 μL/plate) | 1 | 118 | 24 | 93 | 306 | 9 |
|  | 2 | 132 | 29 | 144 | 327 | 11 |
|  | 3 | 111 | 32 | 125 | 296 | 10 |
| Test articles  51.2 | 1 | 99 | 24 | 127 | 309 | 6 |
|  | 2 | 157 | 29 | 125 | 287 | 6 |
|  | 3 | 113 | 32 | 131 | 311 | 11 |
| Test articles  128 | 1 | 130 | 31 | 120 | 313 | 9 |
|  | 2 | 126 | 26 | 106 | 320 | 11 |
|  | 3 | 123 | 36 | 115 | 287 | 12 |
| Test articles  320 | 1 | 116 | 28 | 156 | 298 | 9 |
|  | 2 | 114 | 24 | 131 | 315 | 9 |
|  | 3 | 131 | 25 | 96 | 291 | 6 |
| Test articles  800 | 1 | 91 | 21 | 123 | 313 | 8 |
|  | 2 | 127 | 19 | 136 | 315 | 7 |
|  | 3 | 146 | 17 | 106 | 273 | 8 |
| Test articles  2000 | 1 | 130 | 25 | 133 | 305 | 5 |
|  | 2 | 141 | 25 | 107 | 331 | 7 |
|  | 3 | 150 | 28 | 140 | 298 | 11 |
| Positive control | 1 | 1065 | 1284 | 1453 | 1440 | 354 |
|  | 2 | 1584 | 1012 | 1482 | 1849 | 390 |
|  | 3 | 1267 | 920 | 1223 | 1561 | 387 |

1. Table 5. Institutional data for historical background controls

| **Strain** | **Vehicle control** | | **Positive control** | |
| --- | --- | --- | --- | --- |
|  | **+S9** | **-S9** | **+S9** | **-S9** |
| TA97a | 73~246 | 78~211 | 234~3267 | 744~7152 |
| TA98 | 19~179 | 15~85 | 318~6328 | 520~4056 |
| TA100 | 75~224 | 59~196 | 188~3705 | 888~4906 |
| TA102 | 157~644 | 162~588 | 472~6399 | 1088~7520 |
| TA1535 | 3~53 | 6~35 | 35~1184 | 133~2912 |

***In Vivo* Micronucleus Test of JointAlive in Mice**

Table 1. Summary of animal toxicity data

| **Parameter** | **Gender** | **Vehicle control** | **Test Article** | | | **Positive control** |
| --- | --- | --- | --- | --- | --- | --- |
|  |  |  | **500 mg/kg** | **1000 mg/kg** | **2000 mg/kg** |  |
| Death (identified cases/total animals in the group) | | | | | | |
|  | Male | 0/5 | 0/5 | 0/5 | 0/7 | 0/5 |
|  | Female | 0/5 | 0/5 | 0/5 | 0/7 | 0/5 |
| Dying (identified cases/total animals in the group) | | | | | | |
|  | Male | 0/5 | 0/5 | 0/5 | 0/7 | 0/5 |
|  | Female | 0/5 | 0/5 | 0/5 | 0/7 | 0/5 |
| Weight gain rate (%) | | | | | | |
|  | Male | 1.5% | 6.2% | 6.4% | 4.8% | 5.9% |
|  | Female | -0.2% | -0.9% | -0.1% | -0.5% | -2.1% |
| Abnormality (identified cases/total animals in the group) | | | | | | |
|  | Male | 0/5 | 0/5 | 0/5 | 0/7 | 0/5 |
|  | Female | 0/5 | 0/5 | 0/5 | 0/7 | 0/5 |

Note: weight gain rate (%) = weight gain/weight on day 1 of administration

Table 2. Summary of microscopic examination data of bone marrow smears (mean ± SD)

| **Group** | **Number of animals** | **Micronucleus formation rate (‰)** | **PCE/RBC (%)** |
| --- | --- | --- | --- |
| Vehicle control | 5M | 0.5 ± 0.5 | 67.5 ± 4.3 |
| Vehicle control | 5F | 0.8 ± 0.3 | 60.2 ± 14.4 |
| Total | 10 (5M/5F) | 0.7 ± 0.4 | 63.9 ± 10.7 |
| Test Article  (low-dose group) | 5M | 0.7 ± 0.3 | 69.9 ± 7.1 |
| Test Article  (low-dose group) | 5F | 1.3 ± 0.8 | 64.6 ± 2.2 |
| Total | 10 (5M/5F) | 1.0 ± 0.6 | 67.2 ± 5.7 |
| Test Article  (moderate-dose group) | 5M | 1.1 ± 0.4 | 68.2 ± 7.5 |
| Test Article  (moderate-dose group) | 5F | 1.3 ± 0.5 | 65.1 ± 6.2 |
| Total | 10 (5M/5F) | 1.2 ± 0.5 | 66.6 ± 6.7 |
| Test Article  (high-dose group) | 5M | 1.1 ± 0.3 | 70.8 ± 6.4 |
| Test Article  (high-dose group) | 5F | 0.9 ± 0.3 | 72.0 ± 7.0 |
| Total | 10 (5M/5F) | 1.0 ± 0.3 | 71.4 ± 6.4 |
| (positive control) | 5M | 11.1 ± 1.1* | 58.2 ± 9.1 |
| (positive control) | 5F | 10.4 ± 1.6* | 67.1 ± 9.0 |
| Total | 10 (5M/5F) | 10.8 ± 1.4* | 62.6 ± 9.8 |

Note: "*" indicates P < 0.05 when compared to the vehicle control group; Mean = mean value; SD = standard deviation; M = male; and F = female

1. Table 3. Individual value of animal body weight

| **Animal no. (male)** | **Animal weight (g)** | | | **Animal no. (female)** | **Animal weight (g)** | | |
| --- | --- | --- | --- | --- | --- | --- | --- |
|  | **Day 1 of administration** | **Day of dissection** | **Weight gain** |  | **Day 1 of administration** | **Day of dissection** | **Weight gain** |
| **Vehicle control** | | | |  |  |  |  |
| 1001 | 30.0 | 30.0 | 0.0 | 2001 | 28.7 | 28.7 | 0.0 |
| 1002 | 28.4 | 28.4 | 0.0 | 2002 | 27.1 | 27.1 | 0.0 |
| 1003 | 28.3 | 30.3 | 2.0 | 2003 | 27.0 | 27.1 | 0.1 |
| 1004 | 27.5 | 28.0 | 0.5 | 2004 | 25.7 | 24.8 | -0.9 |
| 1005 | 27.4 | 27.0 | -0.4 | 2005 | 25.7 | 26.2 | 0.5 |
| ***Mean*±SD** | 28.32±1.04 | 28.74±1.39 | 0.42 ±0.94 | ***Mean*±SD** | 26.84±1.24 | 26.78±1.43 | -0.06±0.51 |
| **Test article (500 mg/kg)** | | | |  |  |  |  |
| 1101 | 30.0 | 31.3 | 1.3 | 2101 | 28.4 | 28.2 | -0.2 |
| 1102 | 28.4 | 29.7 | 1.3 | 2102 | 27.1 | 27.2 | 0.1 |
| 1103 | 28.3 | 30.4 | 2.1 | 2103 | 26.9 | 26.3 | -0.6 |
| 1104 | 27.5 | 29.7 | 2.2 | 2104 | 25.8 | 25.0 | -0.8 |
| 1105 | 27.3 | 29.2 | 1.9 | 2105 | 25.7 | 26.0 | 0.3 |
| ***Mean*±SD** | 28.30±1.07 | 30.06±0.81 | 1.76 ±0.43 | ***Mean*±SD** | 26.78±1.10 | 26.54±1.22 | -0.24±0.46 |
| **Test article (1000 mg/kg)** | | | |  |  |  |  |
| 1201 | 29.8 | 31.9 | 2.1 | 2201 | 28.3 | 27.0 | -1.3 |
| 1202 | 28.5 | 30.1 | 1.6 | 2202 | 27.3 | 28.3 | 1.0 |
| 1203 | 28.3 | 29.8 | 1.5 | 2203 | 26.8 | 27.0 | 0.2 |
| 1204 | 27.7 | 29.6 | 1.9 | 2204 | 25.8 | 25.5 | -0.3 |
| 1205 | 27.1 | 29.0 | 1.9 | 2205 | 25.7 | 26.0 | 0.3 |
| ***Mean*±SD** | 28.28±1.01 | 30.08±1.09 | 1.80 ±0.24 | ***Mean*±SD** | 26.78±1.08 | 26.76±1.08 | -0.02±0.85 |
| **Test article (2000 mg/kg)** | | | |  |  |  |  |
| 1301 | 29.8 | 32.5 | 2.7 | 2301 | 28.3 | 26.7 | -1.6 |
| 1302 | 28.6 | 29.9 | 1.3 | 2302 | 27.5 | 27.4 | -0.1 |
| 1303 | 28.3 | 28.9 | 0.6 | 2303 | 26.6 | 27.2 | 0.6 |
| 1304 | 27.7 | 29.2 | 1.5 | 2304 | 25.8 | 25.6 | -0.2 |
| 1305 | 27.1 | 29.1 | 2.0 | 2305 | 25.6 | 26.0 | 0.4 |
| 1306 | 26.1 | 27.4 | 1.3 | 2306 | 25.0 | 24.8 | -0.2 |
| 1307 | 26.0 | 26.3 | 0.3 | 2307 | 25.0 | 25.1 | 0.1 |
| ***Mean*±SD** | 27.66±1.38 | 29.04±1.96 | 1.39 ±0.81 | ***Mean*±SD** | 26.26±1.27 | 26.11±1.02 | -0.14±0.71 |
| **Positive control (CP, 30 mg/kg)** | | | |  |  |  |  |
| 1401 | 29.6 | 32.3 | 2.7 | 2401 | 28.3 | 27.0 | -1.3 |
| 1402 | 28.6 | 29.6 | 1.0 | 2402 | 27.6 | 26.4 | -1.2 |
| 1403 | 28.3 | 30.5 | 2.2 | 2403 | 26.5 | 26.7 | 0.2 |
| 1404 | 27.9 | 28.9 | 1.0 | 2404 | 25.9 | 26.1 | 0.2 |
| 1405 | 26.9 | 28.3 | 1.4 | 2405 | 25.6 | 24.9 | -0.7 |
| ***Mean*±SD** | 28.26±0.99 | 29.92±1.56 | 1.66 ±0.76 | ***Mean*±SD** | 26.78±1.14 | 26.22±0.81 | -0.56±0.73 |

Note: Mean = mean value; SD = standard deviation; standard deviation of weight gain = weight on the dissection day – weight on day 1 of administration.Table 4. Individual value of animal clinical symptoms

| **Animal**  **no. (male)** | **Animal symptom** | | | | | | **Animal**  **no. (female)** | **Animal symptom** | | | | |
| --- | --- | --- | --- | --- | --- | --- | --- | --- | --- | --- | --- | --- |
|  | **The first**  **dose** | | | **The last dose** | | **Day of**  **dissection** |  | **The first**  **dose** | **The last dose** | | | **Day of**  **dissection** |
|  |  |  |  | **Before administration** | **Postdose** |  |  |  | **Before administration** | **Postdose** | |  |
| **Vehicle control** | | | | | | | | | | | | |
| 1001 | | N | N | | N | N | 2001 | N | N | | N | N |
| 1002 | | N | N | | N | N | 2002 | N | N | | N | N |
| 1003 | | N | N | | N | N | 2003 | N | N | | N | N |
| 1004 | | N | N | | N | N | 2004 | N | N | | N | N |
| 1005 | | N | N | | N | N | 2005 | N | N | | N | N |
| **Test article (500 mg/kg)** | | | | | | | | | | | | |
| 1101 | | N | N | | N | N | 2101 | N | N | | N | N |
| 1102 | | N | N | | N | N | 2102 | N | N | | N | N |
| 1103 | | N | N | | N | N | 2103 | N | N | | N | N |
| 1104 | | N | N | | N | N | 2104 | N | N | | N | N |
| 1105 | | N | N | | N | N | 2105 | N | N | | N | N |
| **Test article (1000 mg/kg)** | | | | | | | | | | | | |
| 1201 | | N | N | | N | N | 2201 | N | N | | N | N |
| 1202 | | N | N | | N | N | 2202 | N | N | | N | N |
| 1203 | | N | N | | N | N | 2203 | N | N | | N | N |
| 1204 | | N | N | | N | N | 2204 | N | N | | N | N |
| 1205 | | N | N | | N | N | 2205 | N | N | | N | N |
| **Test article (2000 mg/kg)** | | | | | | |  |  |  | | |  |
| 1301 | N | | | N | N | N | 2301 | N | N | N | | N |
| 1302 | N | | | N | N | N | 2302 | N | N | N | | N |
| 1303 | N | | | N | N | N | 2303 | N | N | N | | N |
| 1304 | N | | | N | N | N | 2304 | N | N | N | | N |
| 1305 | N | | | N | N | N | 2305 | N | N | N | | N |
| 1306 | N | | | N | N | N | 2306 | N | N | N | | N |
| 1307 | N | | | N | N | N | 2307 | N | N | N | | N |
| **Positive control (CP, 30 mg/kg)** | | | | | | |  |  |  | | |  |
| 1401 | N | | | N | N | N | 2401 | N | N | N | | N |
| 1402 | N | | | N | N | N | 2402 | N | N | N | | N |
| 1403 | N | | | N | N | N | 2403 | N | N | N | | N |
| 1404 | N | | | N | N | N | 2404 | N | N | N | | N |
| 1405 | N | | | N | N | N | 2405 | N | N | N | | N |

Note: N = no abnormalities were observed.

1. Table 5. Individual value of microscopic examination of mouse bone marrow smear

| **Animal no.**  **(male)** | **Micronucleus Formation Rate** | | |  | **Bone Marrow Cytotoxicity** | | |
| --- | --- | --- | --- | --- | --- | --- | --- |
|  | **MN-PCE** | **PCE** | **Ratio (‰)** |  | **PCE** | **NCE** | **Ratio (%)** |
| **Vehicle control** | | | | | | | |
| 1001 | 2 | 4001 | 0.5 |  | 321 | 182 | 63.8 |
| 1002 | 3 | 4000 | 0.8 |  | 318 | 189 | 62.7 |
| 1003 | 1 | 4001 | 0.2 |  | 363 | 139 | 72.3 |
| 1004 | 0 | 4003 | 0.0 |  | 342 | 163 | 67.7 |
| 1005 | 5 | 4002 | 1.2 |  | 376 | 153 | 71.1 |
| **Test article (500 mg/kg)** | | | | | | | |
| 1101 | 2 | 4002 | 0.5 |  | 293 | 207 | 58.6 |
| 1102 | 3 | 4003 | 0.7 |  | 387 | 113 | 77.4 |
| 1103 | 4 | 4003 | 1.0 |  | 487 | 204 | 70.5 |
| 1104 | 1 | 4000 | 0.3 |  | 430 | 153 | 73.8 |
| 1105 | 4 | 4000 | 1.0 |  | 345 | 155 | 69.0 |
| **Test article (1000 mg/kg)** | | | | | | | |
| 1201 | 5 | 4040 | 1.2 |  | 455 | 160 | 74.0 |
| 1202 | 4 | 4005 | 1.0 |  | 344 | 190 | 64.4 |
| 1203 | 7 | 4001 | 1.7 |  | 391 | 110 | 78.0 |
| 1204 | 3 | 4003 | 0.7 |  | 302 | 199 | 60.3 |
| 1205 | 3 | 4000 | 0.8 |  | 321 | 179 | 64.2 |
| **Test article (2000 mg/kg)** | | | | | | | |
| 1301 | 6 | 4006 | 1.5 |  | 300 | 200 | 60.0 |
| 1302 | 5 | 4005 | 1.2 |  | 349 | 151 | 69.8 |
| 1303 | 5 | 4011 | 1.2 |  | 407 | 140 | 74.4 |
| 1304 | 4 | 4002 | 1.0 |  | 426 | 146 | 74.5 |
| 1305 | 3 | 4000 | 0.8 |  | 386 | 126 | 75.4 |
| **Positive control (CP, 30 mg/kg)** | | | | | | | |
| 1401 | 44 | 4027 | 10.9 |  | 262 | 238 | 52.4 |
| 1402 | 41 | 4020 | 10.2 |  | 266 | 234 | 53.2 |
| 1403 | 42 | 4024 | 10.4 |  | 284 | 216 | 56.8 |
| 1404 | 52 | 4032 | 12.9 |  | 271 | 229 | 54.2 |
| 1405 | 45 | 4007 | 11.2 |  | 437 | 152 | 74.2 |

1. Table 6. Individual value of microscopic examination of mouse bone marrow smear (continued)

| **Animal no.**  **(female)** | **Micronucleus Formation Rate** | | |  | **Bone Marrow Cytotoxicity** | | |
| --- | --- | --- | --- | --- | --- | --- | --- |
|  | **MN-PCE** | **PCE** | **Ratio (‰)** |  | **PCE** | **NCE** | **Ratio (%)** |
| **Vehicle control** | | | | | | | |
| 2001 | 4 | 4004 | 1.0 |  | 212 | 385 | 35.5 |
| 2002 | 4 | 4000 | 1.0 |  | 357 | 204 | 63.6 |
| 2003 | 2 | 4001 | 0.5 |  | 347 | 153 | 69.4 |
| 2004 | 4 | 4003 | 1.0 |  | 356 | 145 | 71.1 |
| 2005 | 1 | 4000 | 0.3 |  | 331 | 208 | 61.4 |
| **Test article (500 mg/kg)** | | | | | | | |
| 2101 | 6 | 4016 | 1.5 |  | 338 | 163 | 67.5 |
| 2102 | 10 | 4009 | 2.5 |  | 329 | 186 | 63.9 |
| 2103 | 4 | 4013 | 1.0 |  | 356 | 185 | 65.8 |
| 2104 | 3 | 4002 | 0.7 |  | 309 | 192 | 61.7 |
| 2105 | 3 | 4003 | 0.7 |  | 365 | 204 | 64.1 |
| **Test article (1000 mg/kg)** | | | | | | | |
| 2201 | 3 | 4009 | 0.7 |  | 363 | 137 | 72.6 |
| 2202 | 7 | 4008 | 1.7 |  | 290 | 210 | 58.0 |
| 2203 | 8 | 4016 | 2.0 |  | 353 | 147 | 70.6 |
| 2204 | 4 | 4024 | 1.0 |  | 308 | 193 | 61.5 |
| 2205 | 4 | 4026 | 1.0 |  | 316 | 187 | 62.8 |
| **Test article (2000 mg/kg)** | | | | | | | |
| 2301 | 2 | 4000 | 0.5 |  | 367 | 134 | 73.3 |
| 2302 | 3 | 4000 | 0.8 |  | 430 | 109 | 79.8 |
| 2303 | 5 | 4006 | 1.2 |  | 305 | 195 | 61.0 |
| 2304 | 5 | 4007 | 1.2 |  | 353 | 147 | 70.6 |
| 2305 | 4 | 4005 | 1.0 |  | 401 | 131 | 75.4 |
| **Positive control (CP, 30 mg/kg)** | | | | | | | |
| 2401 | 36 | 4006 | 9.0 |  | 373 | 127 | 74.6 |
| 2402 | 50 | 4002 | 12.5 |  | 366 | 143 | 71.9 |
| 2403 | 40 | 4020 | 10.0 |  | 307 | 193 | 61.4 |
| 2404 | 47 | 4027 | 11.7 |  | 270 | 230 | 54.0 |
| 2405 | 35 | 4000 | 8.8 |  | 379 | 135 | 73.7 |

1. Table 7. Laboratory data of the historical background control

|  | **Negative** | |  | **Positive** | |
| --- | --- | --- | --- | --- | --- |
|  | **Mean ± SD** | **Range** |  | **Mean ± SD** | **Range** |
| Male^①^ | 1.41 ±0.74 | 0.39~3.14 |  | 19.76±10.89 | 7.67~50.14 |
| Female^②^ | 1.51±1.02 | 0.00~4.28 |  | 19.45±10.43 | 7.22~51.00 |
| Total | 1.46±0.89 | 0.00~4.28 |  | 19.61±10.67 | 7.22~51.00 |

Note: (1) The sample size is 49 items, and the data is from all GLP studies conducted by this Test Facility from 2013 to 2017. (2) The sample size is 49 items, and the data is from all GLP studies conducted by this Test Facility from 2013 to 2017.

***In Vitro* Mammalian Cell Chromosomal Aberration Test for JointAlive**

1. Table 1. Dissolution of the test article in cell media

| **Processing condition** | **Concentration (µg/mL)** | **Initial state of administration** | | **State after the completion of administration** | |
| --- | --- | --- | --- | --- | --- |
|  |  | **Precipitation** | **pH** | **Precipitation** | **pH** |
| –S9  3 h | DMSO | P0 | Normal | P0 | Normal |
|  | 9.375 | P0 | Normal | P0 | Normal |
|  | 18.75 | P0 | Normal | P0 | Normal |
|  | 37.5 | P0 | Normal | P0 | Normal |
|  | 75 | P0 | Normal | P0 | Normal |
|  | 150 | P0 | Normal | P0 | Normal |
|  | EMS (1000 μg/mL) | P0 | Normal | P0 | Normal |
| +S9  3 h | DMSO | P0 | Normal | P0 | Normal |
|  | 9.375 | P0 | Normal | P0 | Normal |
|  | 18.75 | P0 | Normal | P0 | Normal |
|  | 37.5 | P0 | Normal | P0 | Normal |
|  | 75 | P0 | Normal | P0 | Normal |
|  | 150 | P0 | Normal | P0 | Normal |
|  | CP (5 μg/mL) | P0 | Normal | P0 | Normal |
| –S9  24 h | DMSO | P0 | Normal | P0 | Normal |
|  | 9.375 | P0 | Normal | P0 | Normal |
|  | 18.75 | P0 | Normal | P0 | Normal |
|  | 37.5 | P0 | Normal | P0 | Normal |
|  | 75 | P0 | Normal | P0 | Normal |
|  | 150 | P0 | Normal | P0 | Normal |
|  | EMS (500 μg/mL) | P0 | Normal | P0 | Normal |

Notes: P0 = no precipitation; P1 = weak precipitation; P2 = strong precipitation. Changes in medium pH: normal (no color change); turn yellow; turn purple.

1. Table 2. Cytotoxicity of the test article

| **Processing condition** | **Concentration (µg/mL)** | **Cell count at harvest**  **(× 10^4^ cells)** | **Cell count increase**  **(× 10^4^ cells)** | **Cell growth inhibition rate (%)** |
| --- | --- | --- | --- | --- |
| Baseline cell count (X_0_) | | 43.3 | NA | NA |
| –S9  3 h | DMSO | 148.1 | 104.8 | 0.0 |
|  | 9.375 | 145.3 | 102.0 | 2.7 |
|  | 18.75 | 162.6 | 119.3 | -13.8 |
|  | 37.5 | 140.0 | 96.7 | 7.7 |
|  | 75 | 119.3 | 76.0 | 27.5 |
|  | 150 | 95.1 | 51.8 | 50.6 |
|  | EMS (1000 μg/mL) | 115.6 | 72.3 | 31.0 |
| +S9  3 h | DMSO | 142.6 | 99.3 | 0.0 |
|  | 9.375 | 170.5 | 127.2 | -28.1 |
|  | 18.75 | 161.0 | 117.7 | -18.5 |
|  | 37.5 | 148.3 | 105.0 | -5.7 |
|  | 75 | 121.6 | 78.3 | 21.1 |
|  | 150 | 92.1 | 48.8 | 50.9 |
|  | CP (5 μg/mL) | 110.0 | 66.7 | 32.8 |
| –S9  24 h | DMSO | 141.0 | 97.7 | 0.0 |
|  | 9.375 | 138.0 | 94.7 | 3.1 |
|  | 18.75 | 147.5 | 104.2 | -6.7 |
|  | 37.5 | 133.8 | 90.5 | 7.4 |
|  | 75 | 109.1 | 65.8 | 32.7 |
|  | 150 | 90.0 | 46.7 | 52.2 |
|  | EMS (500 μg/mL) | 102.6 | 59.3 | 39.3 |

Notes: Total viable cell count at harvest = cell density × volume; baseline cell count (X_0_) = baseline cell density × volume (volumes here are both 2 mL)

Cell count increase = total viable cell count at harvest – baseline cell count (X_0_)

RICC (%) = (cell count increase in the dose group/cell count increase in the vehicle control group) × 100%

Cell growth inhibition rate (%) = 1 – RICC (%), indicating cytotoxicity.

1. Table 3. Summary of chromosomal aberration data

| **Processing condition** | **Concentration**  **(µg/mL)** | **Observed cell count (cells)** | **Total count of cells with aberration (cells)** | **Total count of cells with chromosomal aberration (chromosomes)** | **Chromosomal aberration rate (%)** |
| --- | --- | --- | --- | --- | --- |
| –S9  3 h | DMSO | 300 | 2 | 2 | 0.7 |
|  | 37.5 | 300 | 5 | 5 | 1.7 |
|  | 75 | 300 | 3 | 3 | 1.0 |
|  | 150 | 300 | 3 | 3 | 1.0 |
|  | EMS (1000) | 100 | 14 | 14 | 14.0* |
| +S9  3 h | DMSO | 300 | 3 | 3 | 1.0 |
|  | 37.5 | 300 | 2 | 2 | 0.7 |
|  | 75 | 300 | 2 | 2 | 0.7 |
|  | 150 | 300 | 2 | 2 | 0.7 |
|  | CP (5) | 100 | 13 | 13 | 13.0* |
| –S9  24 h | DMSO | 300 | 3 | 3 | 1.0 |
|  | 37.5 | 300 | 5 | 5 | 1.7 |
|  | 75 | 300 | 4 | 4 | 1.3 |
|  | 150 | 300 | 6 | 6 | 2.0 |
|  | EMS (500) | 100 | 12 | 12 | 12.0* |

Notes: A metaphase cell with multiple types of chromosomal aberrations was counted as 1 cell with chromosomal aberration; * indicates significant difference of the concentration group compared with the control group, P < 0.05.

1. Table 4. Laboratory data of the historical background control

| **Processing condition** | **Rate of cells with chromosomal aberration (%)** | | | **Total sample size***  **(samples)** |
| --- | --- | --- | --- | --- |
|  | Mean | Standard deviation | Range |  |
| Historical background data of vehicle control | | | | |
| –S9 for 3 h | 0.86 | 0.45 | 0.00-2.00 | 77 |
| +S9 for 3 h | 0.73 | 0.50 | 0.00-3.50 | 77 |
| –S9 for 24 h | 0.84 | 0.59 | 0.00-3.50 | 77 |
| Historical background data of positive control | | | | |
| –S9 for 3 h | 9.99 | 1.93 | 6.00-17.00 | 77 |
| +S9 for 3 h | 10.14 | 1.93 | 6.00-15.00 | 77 |
| –S9 for 24 h | 10.48 | 2.20 | 6.00-16.00 | 77 |

Note: * Data sourced from all GLP tests conducted in this TEST FACILITY during 2013–2017

1. Table 5. Detailed data of chromosomal aberrations

| **Processing condition** | **Concentration group (µg/mL)** | **Total count of observed cells** | **Count of polyploid cells** | **Count of endoreduplicated cells** | **Count of structural chromosomal aberrations** | | | | | | | | | | | | | | | | | | **Chromosomal Aberration**  **Total cell count**  **(excluding gaps)** | **Total count of aberrant**  **chromosomes (excluding gaps)** |
| --- | --- | --- | --- | --- | --- | --- | --- | --- | --- | --- | --- | --- | --- | --- | --- | --- | --- | --- | --- | --- | --- | --- | --- | --- |
|  |  |  |  |  | Chromatid-type aberration | | | | | | |  | Chromosome-type aberration | | | | | | | | SD | Gap |  |  |
|  |  |  |  |  | Deletion | |  | Exchange | | | |  | Deletion | |  | Exchange | | | | |  |  |  |  |
|  |  |  |  |  | TB | ID |  | TR | QR | CR | TI |  | SB | DM |  | D | DF | MC | R | RF |  |  |  |  |
| –S9  3 h | DMSO | 300 | 0 | 0 | 0 | 1 |  | 0 | 0 | 0 | 0 |  | 0 | 1 |  | 0 | 0 | 0 | 0 | 0 | 0 | 0 | 2 | 2 |
|  | 37.5 | 300 | 0 | 0 | 0 | 2 |  | 1 | 0 | 0 | 0 |  | 1 | 0 |  | 0 | 0 | 0 | 1 | 0 | 0 | 1 | 5 | 5 |
|  | 75 | 300 | 0 | 0 | 2 | 1 |  | 0 | 0 | 0 | 0 |  | 0 | 0 |  | 0 | 0 | 0 | 0 | 0 | 0 | 0 | 3 | 3 |
|  | 150 | 300 | 0 | 0 | 1 | 0 |  | 0 | 0 | 0 | 0 |  | 0 | 1 |  | 0 | 0 | 0 | 1 | 0 | 0 | 1 | 3 | 3 |
|  | EMS (1000) | 100 | 0 | 0 | 9 | 4 |  | 0 | 0 | 0 | 0 |  | 0 | 1 |  | 0 | 0 | 0 | 0 | 0 | 0 | 2 | 14 | 14 |
| +S9  3 h | DMSO | 300 | 0 | 0 | 2 | 1 |  | 0 | 0 | 0 | 0 |  | 0 | 0 |  | 0 | 0 | 0 | 0 | 0 | 0 | 2 | 3 | 3 |
|  | 37.5 | 300 | 0 | 0 | 1 | 0 |  | 0 | 0 | 0 | 0 |  | 1 | 0 |  | 0 | 0 | 0 | 0 | 0 | 0 | 1 | 2 | 2 |
|  | 75 | 300 | 0 | 0 | 2 | 0 |  | 0 | 0 | 0 | 0 |  | 0 | 0 |  | 0 | 0 | 0 | 0 | 0 | 0 | 1 | 2 | 2 |
|  | 150 | 300 | 0 | 0 | 1 | 1 |  | 0 | 0 | 0 | 0 |  | 0 | 0 |  | 0 | 0 | 0 | 0 | 0 | 0 | 0 | 2 | 2 |
|  | CP  (5) | 100 | 0 | 0 | 4 | 7 |  | 1 | 0 | 0 | 0 |  | 1 | 0 |  | 0 | 0 | 0 | 0 | 0 | 0 | 2 | 13 | 13 |
| –S9  24 h | DMSO | 300 | 0 | 0 | 0 | 2 |  | 1 | 0 | 0 | 0 |  | 0 | 0 |  | 0 | 0 | 0 | 0 | 0 | 0 | 0 | 3 | 3 |
|  | 37.5 | 300 | 0 | 0 | 3 | 2 |  | 0 | 0 | 0 | 0 |  | 0 | 0 |  | 0 | 0 | 0 | 0 | 0 | 0 | 0 | 5 | 5 |
|  | 75 | 300 | 0 | 0 | 2 | 1 |  | 0 | 0 | 0 | 0 |  | 1 | 0 |  | 0 | 0 | 0 | 0 | 0 | 0 | 0 | 4 | 4 |
|  | 150 | 300 | 0 | 0 | 2 | 3 |  | 0 | 0 | 0 | 0 |  | 1 | 0 |  | 0 | 0 | 0 | 0 | 0 | 0 | 0 | 6 | 6 |
|  | EMS (500) | 100 | 0 | 0 | 3 | 5 |  | 0 | 0 | 0 | 0 |  | 1 | 1 |  | 0 | 0 | 0 | 2 | 0 | 0 | 0 | 12 | 12 |

Notes: TB = chromatid breakage; ID = chromatid deletion; TR = triradial; QR = quadriradial; CR = complex rearrangement; TI = chromatid intrachange;

SB = chromosomal breakage; DM = double minute; D = dicentric chromosome; DF = dicentric chromosome and its fragment; MC = multicentric chromosome; R = ring; RF = ring and its fragment; and SD = severely damaged cell. Cell with 2 or more abnormal types was counted as 1 abnormal cell.
