# Supplementary material for: Acute, subchronic toxicity and genotoxicity studies of JointAlive, a traditional Chinese medicine formulation for knee osteoarthritis
Source: PLoS One. 2023 Oct 17;18(10):e0292937. doi: 10.1371/journal.pone.0292937 (PMC10581469; doi:10.1371/journal.pone.0292937)
Supplement: S2 File — (DOCX) [file pone.0292937.s003.docx]

**A 13-Week Repeated-Dose Toxicity Study of JointAlive in Rats**

### Individual animal data: clinical observations

| **Dose** | **Gender** | **Animal no.** | **Clinical observation data** | |
| --- | --- | --- | --- | --- |
| **g/kg** |  |  | **Symptom** | **Duration** |
| 0 | Male | 10001 | No abnormalities were observed | D1~D91 |
|  |  | 10002 | No abnormalities were observed | D1~D91 |
|  |  | 10003 | No abnormalities were observed | D1~D91 |
|  |  | 10004 | No abnormalities were observed | D1~D91 |
|  |  | 10005 | No abnormalities were observed | D1~D91 |
|  |  | 10006 | No abnormalities were observed | D1~D91 |
|  |  | 10007 | No abnormalities were observed | D1~D91 |
|  |  | 10008 | No abnormalities were observed | D1~D91 |
|  |  | 10009 | No abnormalities were observed | D1~D91 |
|  |  | 10010 | No abnormalities were observed | D1~D91 |
|  |  | 10011 | No abnormalities were observed | D1~D91, rD1~rD28 |
|  |  | 10012 | No abnormalities were observed | D1~D91, rD1~rD28 |
|  |  | 10013 | No abnormalities were observed | D1~D91, rD1~rD28 |
|  |  | 10014 | No abnormalities were observed | D1~D91, rD1~rD28 |
|  |  | 10015 | No abnormalities were observed | D1~D91, rD1~rD28 |
|  |  | 10016 | No abnormalities were observed | D1~D28 |
|  |  | 10017 | No abnormalities were observed | D1~D28 |
|  |  | 10018 | No abnormalities were observed | D1~D28 |
|  |  | 10019 | No abnormalities were observed | D1~D28 |
|  |  | 10020 | No abnormalities were observed | D1~D28 |
|  | Female | 20021 | No abnormalities were observed | D1~D91 |
|  |  | 20022 | No abnormalities were observed | D1~D91 |
|  |  | 20023 | No abnormalities were observed | D1~D91 |
|  |  | 20024 | No abnormalities were observed | D1~D91 |
|  |  | 20025 | No abnormalities were observed | D1~D91 |
|  |  | 20026 | No abnormalities were observed | D1~D91 |
|  |  | 20027 | No abnormalities were observed | D1~D91 |
|  |  | 20028 | No abnormalities were observed | D1~D91 |
|  |  | 20029 | No abnormalities were observed | D1~D91 |
|  |  | 20030 | No abnormalities were observed | D1~D91 |
|  |  | 20031 | No abnormalities were observed | D1~D91, rD1~rD28 |
|  |  | 20032 | No abnormalities were observed | D1~D91, rD1~rD28 |
|  |  | 20033 | No abnormalities were observed | D1~D91, rD1~rD28 |
|  |  | 20034 | No abnormalities were observed | D1~D91, rD1~rD28 |
|  |  | 20035 | No abnormalities were observed | D1~D91, rD1~rD28 |
|  |  | 20036 | No abnormalities were observed | D1~D28 |
|  |  | 20037 | No abnormalities were observed | D1~D28 |
|  |  | 20038 | No abnormalities were observed | D1~D28 |
|  |  | 20039 | No abnormalities were observed | D1~D28 |
|  |  | 20040 | No abnormalities were observed | D1~D28 |

### Individual animal data: clinical observations

| **Dose** | **Gender** | **Animal no.** | **Clinical observation data** | |
| --- | --- | --- | --- | --- |
| **g/kg** |  |  | **Symptom** | **Duration** |
| 0.5 | Male | 11041 | No abnormalities were observed | D1~D91 |
|  |  | 11042 | No abnormalities were observed | D1~D91 |
|  |  | 11043 | No abnormalities were observed | D1~D91 |
|  |  | 11044 | No abnormalities were observed | D1~D91 |
|  |  | 11045 | No abnormalities were observed | D1~D91 |
|  |  | 11046 | No abnormalities were observed | D1~D91 |
|  |  | 11047 | No abnormalities were observed | D1~D91 |
|  |  | 11048 | No abnormalities were observed | D1~D91 |
|  |  | 11049 | No abnormalities were observed | D1~D91 |
|  |  | 11050 | No abnormalities were observed | D1~D91 |
|  |  | 11051 | No abnormalities were observed | D1~D91, rD1~rD28 |
|  |  | 11052 | No abnormalities were observed | D1~D91, rD1~rD28 |
|  |  | 11053 | No abnormalities were observed | D1~D91, rD1~rD28 |
|  |  | 11054 | No abnormalities were observed | D1~D91, rD1~rD28 |
|  |  | 11055 | No abnormalities were observed | D1~D91, rD1~rD28 |
|  |  | 11056 | No abnormalities were observed | D1~D28 |
|  |  | 11057 | No abnormalities were observed | D1~D28 |
|  |  | 11058 | No abnormalities were observed | D1~D28 |
|  |  | 11059 | No abnormalities were observed | D1~D28 |
|  |  | 11060 | No abnormalities were observed | D1~D28 |
|  | Female | 21061 | No abnormalities were observed | D1~D91 |
|  |  | 21062 | No abnormalities were observed | D1~D91 |
|  |  | 21063 | No abnormalities were observed | D1~D91 |
|  |  | 21064 | No abnormalities were observed | D1~D91 |
|  |  | 21065 | No abnormalities were observed | D1~D91 |
|  |  | 21066 | No abnormalities were observed | D1~D91 |
|  |  | 21067 | No abnormalities were observed | D1~D91 |
|  |  | 21068 | No abnormalities were observed | D1~D91 |
|  |  | 21069 | No abnormalities were observed | D1~D91 |
|  |  | 21070 | No abnormalities were observed | D1~D91 |
|  |  | 21071 | No abnormalities were observed | D1~D91, rD1~rD28 |
|  |  | 21072 | No abnormalities were observed | D1~D91, rD1~rD28 |
|  |  | 21073 | No abnormalities were observed | D1~D91, rD1~rD28 |
|  |  | 21074 | No abnormalities were observed | D1~D91, rD1~rD28 |
|  |  | 21075 | No abnormalities were observed | D1~D91, rD1~rD28 |
|  |  | 21076 | No abnormalities were observed | D1~D28 |
|  |  | 21077 | No abnormalities were observed | D1~D28 |
|  |  | 21078 | No abnormalities were observed | D1~D28 |
|  |  | 21079 | No abnormalities were observed | D1~D28 |
|  |  | 21080 | No abnormalities were observed | D1~D28 |

### Individual animal data: clinical observations

| **Dose** | **Gender** | **Animal no.** | **Clinical observation data** | |
| --- | --- | --- | --- | --- |
| **g/kg** |  |  | **Symptom** | **Duration** |
| 1.5 | Male | 12081 | No abnormalities were observed | D1~D91 |
|  |  | 12082 | No abnormalities were observed | D1~D91 |
|  |  | 12083 | No abnormalities were observed | D1~D91 |
|  |  | 12084 | No abnormalities were observed | D1~D91 |
|  |  | 12085 | No abnormalities were observed | D1~D91 |
|  |  | 12086 | No abnormalities were observed | D1~D91 |
|  |  | 12087 | No abnormalities were observed | D1~D91 |
|  |  | 12088 | No abnormalities were observed | D1~D91 |
|  |  | 12089 | No abnormalities were observed | D1~D91 |
|  |  | 12090 | No abnormalities were observed | D1~D91 |
|  |  | 12091 | No abnormalities were observed | D1~D91, rD1~rD28 |
|  |  | 12092 | No abnormalities were observed | D1~D91, rD1~rD28 |
|  |  | 12093 | No abnormalities were observed | D1~D91, rD1~rD28 |
|  |  | 12094 | No abnormalities were observed | D1~D91, rD1~rD28 |
|  |  | 12095 | No abnormalities were observed | D1~D91, rD1~rD28 |
|  |  | 12096 | No abnormalities were observed | D1~D28 |
|  |  | 12097 | No abnormalities were observed | D1~D28 |
|  |  | 12098 | No abnormalities were observed | D1~D28 |
|  |  | 12099 | No abnormalities were observed | D1~D28 |
|  |  | 12100 | No abnormalities were observed | D1~D28 |
|  | Female | 22101 | No abnormalities were observed | D1~D91 |
|  |  | 22102 | No abnormalities were observed | D1~D91 |
|  |  | 22103 | No abnormalities were observed | D1~D91 |
|  |  | 22104 | No abnormalities were observed | D1~D91 |
|  |  | 22105 | No abnormalities were observed | D1~D91 |
|  |  | 22106 | No abnormalities were observed | D1~D91 |
|  |  | 22107 | No abnormalities were observed | D1~D91 |
|  |  | 22108 | No abnormalities were observed | D1~D91 |
|  |  | 22109 | No abnormalities were observed | D1~D91 |
|  |  | 22110 | No abnormalities were observed | D1~D91 |
|  |  | 22111 | No abnormalities were observed | D1~D91, rD1~rD28 |
|  |  | 22112 | No abnormalities were observed | D1~D91, rD1~rD28 |
|  |  | 22113 | No abnormalities were observed | D1~D91, rD1~rD28 |
|  |  | 22114 | No abnormalities were observed | D1~D91, rD1~rD28 |
|  |  | 22115 | No abnormalities were observed | D1~D91, rD1~rD28 |
|  |  | 22116 | No abnormalities were observed | D1~D28 |
|  |  | 22117 | No abnormalities were observed | D1~D28 |
|  |  | 22118 | No abnormalities were observed | D1~D28 |
|  |  | 22119 | No abnormalities were observed | D1~D28 |
|  |  | 22120 | No abnormalities were observed | D1~D28 |

### Individual animal data: clinical observations

| **Dose** | **Gender** | **Animal no.** | **Clinical observation data** | |
| --- | --- | --- | --- | --- |
| **g/kg** |  |  | **Symptom** | **Duration** |
| 5 | Male | 13121 | No abnormalities were observed | D1~D91 |
|  |  | 13122 | No abnormalities were observed | D1~D91 |
|  |  | 13123 | No abnormalities were observed | D1~D91 |
|  |  | 13124 | No abnormalities were observed | D1~D91 |
|  |  | 13125 | No abnormalities were observed | D1~D91 |
|  |  | 13126 | No abnormalities were observed | D1~D91 |
|  |  | 13127 | No abnormalities were observed | D1~D91 |
|  |  | 13128 | No abnormalities were observed | D1~D91 |
|  |  | 13129 | No abnormalities were observed | D1~D91 |
|  |  | 13130 | No abnormalities were observed | D1~D91 |
|  |  | 13131 | No abnormalities were observed | D1~D91, rD1~rD28 |
|  |  | 13132 | No abnormalities were observed | D1~D91, rD1~rD28 |
|  |  | 13133 | No abnormalities were observed | D1~D91, rD1~rD28 |
|  |  | 13134 | No abnormalities were observed | D1~D91, rD1~rD28 |
|  |  | 13135 | No abnormalities were observed | D1~D91, rD1~rD28 |
|  |  | 13136 | No abnormalities were observed | D1~D28 |
|  |  | 13137 | No abnormalities were observed | D1~D28 |
|  |  | 13138 | No abnormalities were observed | D1~D28 |
|  |  | 13139 | No abnormalities were observed | D1~D28 |
|  |  | 13140 | No abnormalities were observed | D1~D28 |
|  | Female | 23141 | No abnormalities were observed | D1~D91 |
|  |  | 23142 | No abnormalities were observed | D1~D91 |
|  |  | 23143 | No abnormalities were observed | D1~D91 |
|  |  | 23144 | No abnormalities were observed | D1~D91 |
|  |  | 23145 | No abnormalities were observed | D1~D91 |
|  |  | 23146 | No abnormalities were observed | D1~D91 |
|  |  | 23147 | No abnormalities were observed | D1~D91 |
|  |  | 23148 | No abnormalities were observed | D1~D91 |
|  |  | 23149 | No abnormalities were observed | D1~D91 |
|  |  | 23150 | No abnormalities were observed | D1~D91 |
|  |  | 23151 | No abnormalities were observed | D1~D91, rD1~rD28 |
|  |  | 23152 | No abnormalities were observed | D1~D91, rD1~rD28 |
|  |  | 23153 | No abnormalities were observed | D1~D91, rD1~rD28 |
|  |  | 23154 | No abnormalities were observed | D1~D91, rD1~rD28 |
|  |  | 23155 | No abnormalities were observed | D1~D91, rD1~rD28 |
|  |  | 23156 | No abnormalities were observed | D1~D28 |
|  |  | 23157 | No abnormalities were observed | D1~D28 |
|  |  | 23158 | No abnormalities were observed | D1~D28 |
|  |  | 23159 | No abnormalities were observed | D1~D28 |
|  |  | 23160 | No abnormalities were observed | D1~D28 |

### Individual animal data: body weight

| **Dose** | **Gender** | **Animal no.** | **Body weight (g)** | | | | |
| --- | --- | --- | --- | --- | --- | --- | --- |
| **g/kg** |  |  | **D-1** | **D7** | **D14** | **D21** | **D28** |
| 0 | Male | 10001 | 190.0 | 266.7 | 347.3 | 396.7 | 453.2 |
|  |  | 10002 | 194.6 | 269.1 | 338.4 | 397.3 | 444.8 |
|  |  | 10003 | 190.9 | 260.2 | 315.5 | 350.6 | 386.4 |
|  |  | 10004 | 198.2 | 274.5 | 350.0 | 412.4 | 462.9 |
|  |  | 10005 | 195.2 | 260.0 | 322.3 | 372.6 | 407.9 |
|  |  | 10006 | 199.1 | 277.6 | 352.7 | 404.5 | 454.7 |
|  |  | 10007 | 200.5 | 274.9 | 349.4 | 411.6 | 460.4 |
|  |  | 10008 | 201.7 | 271.7 | 343.0 | 392.4 | 439.6 |
|  |  | 10009 | 203.5 | 275.1 | 334.3 | 388.7 | 433.7 |
|  |  | 10010 | 203.3 | 263.4 | 327.1 | 371.2 | 408.0 |
|  |  | 10011 | 198.2 | 273.0 | 320.0 | 394.9 | 442.5 |
|  |  | 10012 | 203.2 | 271.2 | 329.4 | 408.4 | 457.0 |
|  |  | 10013 | 200.3 | 259.7 | 287.0 | 339.4 | 380.6 |
|  |  | 10014 | 205.5 | 277.6 | 347.6 | 404.1 | 451.6 |
|  |  | 10015 | 204.3 | 279.7 | 360.0 | 413.0 | 461.2 |
|  |  | 10016 | 209.4 | 281.7 | 357.0 | 413.6 | 465.3 |
|  |  | 10017 | 201.0 | 266.5 | 317.4 | 353.6 | 371.4 |
|  |  | 10018 | 205.0 | 275.7 | 333.1 | 374.4 | 404.5 |
|  |  | 10019 | 207.8 | 290.4 | 355.3 | 405.0 | 446.5 |
|  |  | 10020 | 207.2 | 271.5 | 338.6 | 393.5 | 441.1 |
|  | Female | 20021 | 169.6 | 192.1 | 211.1 | 227.3 | 242.3 |
|  |  | 20022 | 175.0 | 214.4 | 241.0 | 259.2 | 279.8 |
|  |  | 20023 | 178.9 | 219.6 | 245.1 | 257.8 | 268.6 |
|  |  | 20024 | 177.6 | 213.0 | 232.0 | 257.5 | 270.5 |
|  |  | 20025 | 176.3 | 204.6 | 226.0 | 247.1 | 256.8 |
|  |  | 20026 | 178.7 | 199.0 | 225.0 | 236.6 | 243.6 |
|  |  | 20027 | 182.2 | 225.0 | 248.3 | 260.1 | 275.1 |
|  |  | 20028 | 180.9 | 196.4 | 219.5 | 228.9 | 235.6 |
|  |  | 20029 | 182.5 | 210.6 | 234.0 | 262.0 | 271.8 |
|  |  | 20030 | 177.8 | 208.4 | 237.2 | 258.6 | 267.5 |
|  |  | 20031 | 181.7 | 201.3 | 236.3 | 250.9 | 265.7 |
|  |  | 20032 | 182.8 | 211.1 | 227.6 | 244.3 | 254.6 |
|  |  | 20033 | 180.1 | 212.5 | 231.4 | 250.3 | 260.6 |
|  |  | 20034 | 192.8 | 221.0 | 262.4 | 275.0 | 294.8 |
|  |  | 20035 | 190.1 | 207.0 | 231.0 | 241.4 | 241.6 |
|  |  | 20036 | 190.2 | 224.4 | 240.4 | 265.0 | 278.6 |
|  |  | 20037 | 191.0 | 230.0 | 259.2 | 255.1 | 275.3 |
|  |  | 20038 | 190.5 | 210.4 | 226.7 | 236.3 | 254.1 |
|  |  | 20039 | 194.6 | 222.1 | 241.0 | 269.4 | 282.1 |
|  |  | 20040 | 191.5 | 206.1 | 242.0 | 239.7 | 250.2 |

### Individual animal data: body weight

| **Dose** | **Gender** | **Animal no.** | **Body weight (g)** | | | | |
| --- | --- | --- | --- | --- | --- | --- | --- |
| **g/kg** |  |  | **D-1** | **D7** | **D14** | **D21** | **D28** |
| 0.5 | Male | 11041 | 196.6 | 253.0 | 330.0 | 373.7 | 409.6 |
|  |  | 11042 | 194.4 | 263.1 | 330.3 | 378.1 | 425.8 |
|  |  | 11043 | 198.0 | 266.9 | 327.0 | 370.9 | 407.5 |
|  |  | 11044 | 199.4 | 267.0 | 323.6 | 367.0 | 401.0 |
|  |  | 11045 | 197.4 | 276.9 | 356.0 | 429.0 | 477.8 |
|  |  | 11046 | 199.6 | 264.0 | 328.6 | 380.0 | 420.7 |
|  |  | 11047 | 202.0 | 274.9 | 341.4 | 401.2 | 441.9 |
|  |  | 11048 | 200.4 | 258.6 | 307.4 | 350.0 | 381.3 |
|  |  | 11049 | 198.8 | 273.0 | 337.6 | 397.4 | 447.6 |
|  |  | 11050 | 201.3 | 271.2 | 346.5 | 406.9 | 466.4 |
|  |  | 11051 | 204.1 | 285.2 | 365.0 | 418.5 | 463.3 |
|  |  | 11052 | 200.3 | 278.1 | 345.8 | 397.9 | 437.9 |
|  |  | 11053 | 202.5 | 270.6 | 336.5 | 388.0 | 424.1 |
|  |  | 11054 | 204.3 | 284.8 | 363.4 | 424.8 | 474.3 |
|  |  | 11055 | 198.9 | 270.3 | 338.0 | 384.9 | 423.2 |
|  |  | 11056 | 210.2 | 283.2 | 355.2 | 412.5 | 456.4 |
|  |  | 11057 | 206.5 | 268.9 | 327.1 | 371.2 | 412.2 |
|  |  | 11058 | 206.6 | 289.9 | 366.9 | 430.9 | 483.6 |
|  |  | 11059 | 206.9 | 290.7 | 367.0 | 430.8 | 487.2 |
|  |  | 11060 | 209.1 | 279.6 | 350.1 | 401.9 | 454.0 |
|  | Female | 21061 | 162.1 | 190.7 | 214.0 | 229.2 | 230.3 |
|  |  | 21062 | 170.9 | 198.2 | 216.6 | 233.4 | 241.3 |
|  |  | 21063 | 175.5 | 188.7 | 204.1 | 212.3 | 223.4 |
|  |  | 21064 | 174.4 | 203.0 | 227.0 | 242.5 | 248.8 |
|  |  | 21065 | 173.8 | 201.9 | 232.7 | 250.3 | 259.0 |
|  |  | 21066 | 175.4 | 199.1 | 217.4 | 237.4 | 250.2 |
|  |  | 21067 | 182.4 | 212.0 | 241.7 | 261.7 | 277.1 |
|  |  | 21068 | 180.8 | 208.9 | 226.9 | 237.8 | 250.1 |
|  |  | 21069 | 183.3 | 210.8 | 234.0 | 241.1 | 266.1 |
|  |  | 21070 | 179.0 | 199.4 | 213.9 | 229.7 | 239.2 |
|  |  | 21071 | 187.6 | 211.0 | 240.0 | 253.0 | 268.5 |
|  |  | 21072 | 185.4 | 212.7 | 220.4 | 246.9 | 265.2 |
|  |  | 21073 | 182.6 | 210.2 | 240.4 | 247.5 | 268.6 |
|  |  | 21074 | 186.3 | 222.4 | 251.7 | 259.6 | 270.4 |
|  |  | 21075 | 191.3 | 210.4 | 235.6 | 249.3 | 256.0 |
|  |  | 21076 | 189.2 | 213.9 | 231.1 | 244.5 | 253.9 |
|  |  | 21077 | 191.8 | 218.2 | 232.7 | 256.3 | 270.9 |
|  |  | 21078 | 191.8 | 224.7 | 256.5 | 267.5 | 284.2 |
|  |  | 21079 | 184.6 | 216.4 | 237.0 | 252.2 | 265.7 |
|  |  | 21080 | 192.5 | 223.2 | 241.6 | 257.9 | 274.9 |

### Individual animal data: body weight

| **Dose** | **Gender** | **Animal no.** | **Body weight (g)** | | | | |
| --- | --- | --- | --- | --- | --- | --- | --- |
| **g/kg** |  |  | **D-1** | **D7** | **D14** | **D21** | **D28** |
| 1.5 | Male | 12081 | 193.6 | 267.2 | 330.6 | 391.9 | 434.2 |
|  |  | 12082 | 196.4 | 267.1 | 329.7 | 387.3 | 422.3 |
|  |  | 12083 | 183.0 | 247.7 | 308.0 | 365.2 | 401.4 |
|  |  | 12084 | 198.0 | 272.6 | 333.7 | 390.5 | 426.7 |
|  |  | 12085 | 198.8 | 271.2 | 342.6 | 401.0 | 443.3 |
|  |  | 12086 | 195.3 | 258.0 | 317.0 | 351.9 | 384.5 |
|  |  | 12087 | 202.7 | 273.5 | 345.5 | 409.6 | 460.4 |
|  |  | 12088 | 199.5 | 256.0 | 300.0 | 336.2 | 366.6 |
|  |  | 12089 | 198.7 | 268.0 | 328.0 | 384.4 | 425.0 |
|  |  | 12090 | 203.2 | 283.1 | 366.0 | 428.6 | 484.3 |
|  |  | 12091 | 206.7 | 278.0 | 350.0 | 397.3 | 449.5 |
|  |  | 12092 | 205.3 | 283.2 | 355.0 | 401.5 | 451.0 |
|  |  | 12093 | 203.2 | 275.0 | 337.0 | 383.9 | 434.2 |
|  |  | 12094 | 203.9 | 276.7 | 344.6 | 393.8 | 435.4 |
|  |  | 12095 | 207.6 | 278.6 | 350.3 | 400.3 | 440.9 |
|  |  | 12096 | 207.6 | 279.6 | 355.0 | 409.2 | 463.8 |
|  |  | 12097 | 206.0 | 278.2 | 333.3 | 375.0 | 410.2 |
|  |  | 12098 | 205.1 | 288.2 | 365.0 | 425.7 | 480.1 |
|  |  | 12099 | 206.4 | 278.6 | 342.0 | 395.4 | 430.2 |
|  |  | 12100 | 207.6 | 281.7 | 369.5 | 428.1 | 475.3 |
|  | Female | 22101 | 168.6 | 193.2 | 207.9 | 220.2 | 232.0 |
|  |  | 22102 | 177.2 | 203.5 | 230.4 | 245.7 | 262.8 |
|  |  | 22103 | 175.8 | 199.6 | 211.7 | 237.7 | 246.1 |
|  |  | 22104 | 173.6 | 195.0 | 204.6 | 219.4 | 228.2 |
|  |  | 22105 | 171.7 | 189.0 | 195.0 | 208.8 | 221.6 |
|  |  | 22106 | 181.9 | 214.2 | 236.5 | 249.3 | 258.3 |
|  |  | 22107 | 181.0 | 210.0 | 220.6 | 246.6 | 254.9 |
|  |  | 22108 | 182.2 | 213.1 | 237.0 | 254.2 | 271.8 |
|  |  | 22109 | 177.1 | 216.7 | 242.6 | 254.7 | 271.2 |
|  |  | 22110 | 180.2 | 215.0 | 232.0 | 245.6 | 259.2 |
|  |  | 22111 | 179.8 | 206.4 | 227.0 | 233.7 | 244.0 |
|  |  | 22112 | 193.1 | 211.5 | 246.0 | 256.1 | 284.0 |
|  |  | 22113 | 188.0 | 207.1 | 232.4 | 254.4 | 267.7 |
|  |  | 22114 | 186.3 | 216.3 | 231.9 | 250.9 | 268.5 |
|  |  | 22115 | 189.6 | 216.2 | 236.7 | 255.6 | 269.1 |
|  |  | 22116 | 191.9 | 215.6 | 228.0 | 257.6 | 261.9 |
|  |  | 22117 | 189.6 | 217.1 | 232.6 | 261.9 | 273.3 |
|  |  | 22118 | 187.7 | 233.2 | 259.1 | 272.3 | 293.5 |
|  |  | 22119 | 193.3 | 238.6 | 264.7 | 293.0 | 306.6 |
|  |  | 22120 | 195.2 | 227.7 | 260.7 | 271.1 | 283.1 |

### Individual animal data: body weight

| **Dose** | **Gender** | **Animal no.** | **Body weight (g)** | | | | |
| --- | --- | --- | --- | --- | --- | --- | --- |
| **g/kg** |  |  | **D-1** | **D7** | **D14** | **D21** | **D28** |
| 5 | Male | 13121 | 190.7 | 257.0 | 314.7 | 377.9 | 418.0 |
|  |  | 13122 | 190.9 | 262.5 | 314.0 | 361.3 | 406.0 |
|  |  | 13123 | 196.2 | 270.0 | 334.6 | 396.7 | 437.6 |
|  |  | 13124 | 200.4 | 268.9 | 336.0 | 392.9 | 444.5 |
|  |  | 13125 | 196.2 | 266.7 | 335.7 | 382.3 | 420.9 |
|  |  | 13126 | 201.6 | 272.4 | 339.6 | 370.3 | 426.8 |
|  |  | 13127 | 196.6 | 271.4 | 341.0 | 363.4 | 414.8 |
|  |  | 13128 | 202.2 | 277.6 | 359.4 | 402.2 | 446.7 |
|  |  | 13129 | 203.6 | 284.6 | 377.7 | 433.2 | 498.8 |
|  |  | 13130 | 201.6 | 268.5 | 335.6 | 392.5 | 425.9 |
|  |  | 13131 | 198.9 | 270.4 | 346.0 | 394.2 | 442.6 |
|  |  | 13132 | 201.2 | 278.2 | 342.5 | 388.0 | 429.7 |
|  |  | 13133 | 203.9 | 275.1 | 336.7 | 394.0 | 426.4 |
|  |  | 13134 | 202.4 | 274.0 | 318.7 | 368.2 | 395.6 |
|  |  | 13135 | 204.9 | 270.0 | 330.5 | 374.7 | 413.6 |
|  |  | 13136 | 205.1 | 286.9 | 356.4 | 404.6 | 476.3 |
|  |  | 13137 | 211.6 | 284.6 | 352.5 | 410.1 | 461.6 |
|  |  | 13138 | 210.5 | 281.2 | 341.2 | 390.7 | 434.0 |
|  |  | 13139 | 205.1 | 276.2 | 336.0 | 384.4 | 425.2 |
|  |  | 13140 | 206.2 | 272.1 | 340.0 | 402.1 | 437.2 |
|  | Female | 23141 | 173.7 | 198.5 | 217.9 | 240.8 | 247.7 |
|  |  | 23142 | 176.0 | 199.7 | 214.1 | 226.3 | 234.5 |
|  |  | 23143 | 173.4 | 197.7 | 218.5 | 242.0 | 251.3 |
|  |  | 23144 | 174.9 | 208.4 | 236.0 | 260.0 | 272.2 |
|  |  | 23145 | 181.1 | 216.1 | 246.7 | 262.7 | 270.7 |
|  |  | 23146 | 172.6 | 206.0 | 225.0 | 236.7 | 246.5 |
|  |  | 23147 | 180.6 | 202.1 | 212.7 | 235.2 | 241.6 |
|  |  | 23148 | 177.9 | 221.6 | 244.6 | 263.3 | 284.6 |
|  |  | 23149 | 177.6 | 208.6 | 255.2 | 272.9 | 290.3 |
|  |  | 23150 | 179.9 | 207.7 | 234.7 | 257.6 | 270.0 |
|  |  | 23151 | 188.6 | 222.4 | 246.7 | 271.7 | 279.7 |
|  |  | 23152 | 182.7 | 221.4 | 246.3 | 267.4 | 278.2 |
|  |  | 23153 | 186.8 | 211.0 | 230.4 | 244.2 | 257.0 |
|  |  | 23154 | 189.9 | 223.5 | 244.7 | 272.9 | 282.3 |
|  |  | 23155 | 182.3 | 206.2 | 219.2 | 238.8 | 245.0 |
|  |  | 23156 | 183.3 | 214.1 | 235.8 | 251.7 | 264.1 |
|  |  | 23157 | 193.1 | 223.6 | 249.7 | 265.9 | 276.0 |
|  |  | 23158 | 193.1 | 220.2 | 248.4 | 263.9 | 278.6 |
|  |  | 23159 | 196.6 | 229.4 | 245.0 | 264.7 | 281.2 |
|  |  | 23160 | 192.7 | 233.4 | 261.1 | 274.6 | 292.8 |

### Individual animal data: body weight

| **Dose** | **Gender** | **Animal no.** | **Body weight (g)** | | | | |
| --- | --- | --- | --- | --- | --- | --- | --- |
| **g/kg** |  |  | **D35** | **D42** | **D49** | **D56** | **D63** |
| 0 | Male | 10001 | 499.2 | 543.7 | 571.0 | 596.9 | 619.4 |
|  |  | 10002 | 494.2 | 530.5 | 552.6 | 582.0 | 595.8 |
|  |  | 10003 | 431.9 | 455.1 | 467.1 | 483.7 | 498.3 |
|  |  | 10004 | 504.6 | 550.4 | 578.5 | 606.4 | 630.8 |
|  |  | 10005 | 450.3 | 484.4 | 501.4 | 523.4 | 532.7 |
|  |  | 10006 | 515.9 | 554.6 | 566.2 | 607.8 | 641.6 |
|  |  | 10007 | 514.6 | 547.7 | 547.7 | 582.3 | 613.2 |
|  |  | 10008 | 482.2 | 510.4 | 520.2 | 549.5 | 574.0 |
|  |  | 10009 | 471.1 | 504.1 | 527.8 | 545.2 | 564.8 |
|  |  | 10010 | 443.8 | 474.7 | 484.4 | 498.0 | 514.7 |
|  |  | 10011 | 492.3 | 525.0 | 545.0 | 569.2 | 586.9 |
|  |  | 10012 | 510.5 | 555.0 | 581.6 | 610.2 | 633.3 |
|  |  | 10013 | 417.7 | 445.4 | 453.7 | 479.2 | 485.6 |
|  |  | 10014 | 492.2 | 531.0 | 554.5 | 584.0 | 609.1 |
|  |  | 10015 | 509.7 | 543.4 | 571.8 | 598.9 | 603.0 |
|  | Female | 20021 | 246.6 | 257.1 | 258.4 | 262.0 | 268.2 |
|  |  | 20022 | 281.7 | 293.8 | 299.3 | 307.1 | 303.1 |
|  |  | 20023 | 283.0 | 290.5 | 306.0 | 307.6 | 316.6 |
|  |  | 20024 | 274.6 | 287.6 | 297.8 | 298.6 | 301.1 |
|  |  | 20025 | 273.1 | 284.6 | 294.3 | 296.6 | 305.4 |
|  |  | 20026 | 272.4 | 272.0 | 273.2 | 283.4 | 298.2 |
|  |  | 20027 | 291.4 | 298.1 | 300.5 | 316.2 | 313.8 |
|  |  | 20028 | 251.8 | 262.1 | 266.5 | 267.6 | 279.3 |
|  |  | 20029 | 271.9 | 285.1 | 291.4 | 292.4 | 289.6 |
|  |  | 20030 | 278.0 | 289.6 | 298.9 | 313.4 | 318.9 |
|  |  | 20031 | 284.4 | 286.2 | 289.8 | 299.3 | 305.4 |
|  |  | 20032 | 274.8 | 278.0 | 278.5 | 283.0 | 289.5 |
|  |  | 20033 | 274.8 | 276.7 | 281.0 | 286.7 | 293.7 |
|  |  | 20034 | 319.2 | 327.0 | 331.3 | 342.9 | 355.3 |
|  |  | 20035 | 265.6 | 260.6 | 271.1 | 279.8 | 265.2 |

### Individual animal data: body weight

| **Dose** | **Gender** | **Animal no.** | **Body weight (g)** | | | | |
| --- | --- | --- | --- | --- | --- | --- | --- |
| **g/kg** |  |  | **D35** | **D42** | **D49** | **D56** | **D63** |
| 0.5 | Male | 11041 | 443.7 | 474.6 | 498.4 | 500.9 | 528.0 |
|  |  | 11042 | 376.4 | 510.0 | 535.5 | 547.9 | 566.8 |
|  |  | 11043 | 456.3 | 491.6 | 510.6 | 528.3 | 547.0 |
|  |  | 11044 | 439.0 | 465.6 | 489.4 | 511.9 | 527.2 |
|  |  | 11045 | 515.3 | 551.5 | 576.0 | 594.5 | 603.8 |
|  |  | 11046 | 461.9 | 488.0 | 516.4 | 534.2 | 555.4 |
|  |  | 11047 | 480.0 | 504.4 | 527.2 | 526.0 | 537.9 |
|  |  | 11048 | 417.0 | 441.5 | 460.7 | 471.5 | 478.2 |
|  |  | 11049 | 484.1 | 517.0 | 544.8 | 564.0 | 586.6 |
|  |  | 11050 | 512.4 | 559.9 | 596.4 | 624.4 | 652.9 |
|  |  | 11051 | 505.8 | 543.0 | 566.1 | 586.1 | 603.4 |
|  |  | 11052 | 473.2 | 497.4 | 516.5 | 528.1 | 550.3 |
|  |  | 11053 | 466.2 | 489.8 | 510.6 | 528.9 | 547.8 |
|  |  | 11054 | 519.4 | 563.6 | 589.2 | 614.6 | 641.2 |
|  |  | 11055 | 455.9 | 476.4 | 491.0 | 512.7 | 534.0 |
|  | Female | 21061 | 244.6 | 256.0 | 254.9 | 270.9 | 258.9 |
|  |  | 21062 | 244.4 | 259.0 | 269.6 | 271.6 | 271.6 |
|  |  | 21063 | 227.2 | 238.0 | 257.8 | 259.2 | 256.8 |
|  |  | 21064 | 266.8 | 277.2 | 281.6 | 281.6 | 285.5 |
|  |  | 21065 | 280.7 | 292.9 | 296.7 | 292.6 | 305.3 |
|  |  | 21066 | 262.5 | 267.4 | 282.0 | 272.9 | 282.1 |
|  |  | 21067 | 292.8 | 301.6 | 308.0 | 306.5 | 314.8 |
|  |  | 21068 | 265.1 | 265.9 | 270.9 | 276.3 | 274.0 |
|  |  | 21069 | 284.9 | 269.5 | 291.7 | 283.4 | 310.8 |
|  |  | 21070 | 250.2 | 253.4 | 262.4 | 262.7 | 270.7 |
|  |  | 21071 | 290.9 | 294.6 | 296.7 | 310.4 | 313.2 |
|  |  | 21072 | 280.8 | 281.1 | 299.6 | 302.2 | 307.5 |
|  |  | 21073 | 278.6 | 287.7 | 297.7 | 303.6 | 306.1 |
|  |  | 21074 | 283.9 | 291.2 | 292.0 | 301.5 | 302.6 |
|  |  | 21075 | 265.5 | 276.3 | 286.7 | 285.9 | 290.4 |

### Individual animal data: body weight

| **Dose** | **Gender** | **Animal no.** | **Body weight (g)** | | | | |
| --- | --- | --- | --- | --- | --- | --- | --- |
| **g/kg** |  |  | **D35** | **D42** | **D49** | **D56** | **D63** |
| 1.5 | Male | 12081 | 481.7 | 516.2 | 551.8 | 567.5 | 589.8 |
|  |  | 12082 | 458.0 | 500.5 | 531.7 | 548.2 | 570.1 |
|  |  | 12083 | 441.1 | 477.0 | 493.6 | 517.5 | 528.6 |
|  |  | 12084 | 470.1 | 502.6 | 535.0 | 551.3 | 564.7 |
|  |  | 12085 | 477.7 | 510.2 | 538.1 | 552.1 | 564.2 |
|  |  | 12086 | 413.4 | 432.5 | 450.0 | 465.1 | 474.2 |
|  |  | 12087 | 501.7 | 534.7 | 555.7 | 586.9 | 605.3 |
|  |  | 12088 | 394.0 | 415.5 | 435.1 | 451.2 | 469.2 |
|  |  | 12089 | 460.0 | 483.1 | 499.6 | 514.2 | 529.7 |
|  |  | 12090 | 534.4 | 570.5 | 605.5 | 629.4 | 654.5 |
|  |  | 12091 | 494.7 | 526.0 | 562.8 | 585.6 | 602.8 |
|  |  | 12092 | 486.0 | 517.5 | 530.8 | 550.1 | 560.2 |
|  |  | 12093 | 472.5 | 496.2 | 523.7 | 545.7 | 558.6 |
|  |  | 12094 | 480.3 | 507.5 | 530.4 | 555.2 | 575.0 |
|  |  | 12095 | 482.5 | 507.1 | 532.4 | 554.5 | 572.0 |
|  | Female | 22101 | 255.3 | 253.7 | 257.0 | 259.7 | 272.6 |
|  |  | 22102 | 285.2 | 287.0 | 283.5 | 294.0 | 297.2 |
|  |  | 22103 | 263.4 | 251.2 | 271.2 | 277.7 | 285.3 |
|  |  | 22104 | 246.6 | 251.4 | 248.6 | 256.8 | 266.6 |
|  |  | 22105 | 229.7 | 231.0 | 245.2 | 246.5 | 256.2 |
|  |  | 22106 | 277.3 | 288.2 | 303.7 | 301.6 | 303.1 |
|  |  | 22107 | 277.8 | 282.0 | 289.0 | 297.2 | 308.7 |
|  |  | 22108 | 286.3 | 292.7 | 299.2 | 309.7 | 306.7 |
|  |  | 22109 | 294.4 | 294.4 | 302.3 | 309.5 | 315.2 |
|  |  | 22110 | 281.9 | 287.5 | 288.4 | 297.6 | 306.7 |
|  |  | 22111 | 263.8 | 270.1 | 277.5 | 275.5 | 285.1 |
|  |  | 22112 | 289.8 | 290.4 | 307.3 | 309.0 | 315.2 |
|  |  | 22113 | 283.0 | 287.8 | 306.2 | 307.9 | 316.0 |
|  |  | 22114 | 283.9 | 288.0 | 299.8 | 310.6 | 317.2 |
|  |  | 22115 | 284.0 | 294.4 | 297.5 | 295.6 | 305.5 |

### Individual animal data: body weight

| **Dose** | **Gender** | **Animal no.** | **Body weight (g)** | | | | |
| --- | --- | --- | --- | --- | --- | --- | --- |
| **g/kg** |  |  | **D35** | **D42** | **D49** | **D56** | **D63** |
| 5 | Male | 13121 | 452.4 | 486.9 | 503.9 | 520.3 | 535.9 |
|  |  | 13122 | 443.8 | 474.9 | 500.4 | 526.1 | 541.2 |
|  |  | 13123 | 466.9 | 492.5 | 523.3 | 550.8 | 556.9 |
|  |  | 13124 | 470.8 | 501.9 | 536.4 | 541.9 | 561.0 |
|  |  | 13125 | 445.6 | 478.6 | 454.7 | 485.0 | 513.1 |
|  |  | 13126 | 471.3 | 493.0 | 525.9 | 548.7 | 564.5 |
|  |  | 13127 | 450.1 | 483.6 | 502.3 | 522.0 | 546.5 |
|  |  | 13128 | 485.7 | 527.1 | 557.0 | 584.6 | 608.0 |
|  |  | 13129 | 548.8 | 590.1 | 618.4 | 630.6 | 657.8 |
|  |  | 13130 | 470.6 | 497.2 | 522.8 | 536.9 | 545.6 |
|  |  | 13131 | 490.9 | 534.0 | 555.2 | 572.6 | 606.4 |
|  |  | 13132 | 465.1 | 506.1 | 534.7 | 552.4 | 564.2 |
|  |  | 13133 | 464.2 | 504.2 | 529.9 | 546.2 | 561.1 |
|  |  | 13134 | 425.3 | 454.1 | 472.0 | 491.6 | 508.0 |
|  |  | 13135 | 435.7 | 471.6 | 461.2 | 487.0 | 504.3 |
|  | Female | 23141 | 256.2 | 257.9 | 274.7 | 277.1 | 284.2 |
|  |  | 23142 | 249.4 | 253.0 | 254.2 | 258.5 | 261.2 |
|  |  | 23143 | 261.0 | 270.2 | 278.0 | 278.5 | 282.7 |
|  |  | 23144 | 282.1 | 296.2 | 306.5 | 312.8 | 312.2 |
|  |  | 23145 | 288.4 | 303.4 | 310.9 | 310.3 | 324.9 |
|  |  | 23146 | 261.6 | 276.4 | 289.5 | 271.4 | 280.3 |
|  |  | 23147 | 256.8 | 257.2 | 266.0 | 298.4 | 309.4 |
|  |  | 23148 | 297.1 | 305.9 | 309.7 | 319.5 | 318.9 |
|  |  | 23149 | 305.4 | 304.9 | 310.0 | 309.7 | 300.8 |
|  |  | 23150 | 288.2 | 294.5 | 304.7 | 303.4 | 299.3 |
|  |  | 23151 | 299.4 | 305.2 | 312.1 | 317.2 | 327.2 |
|  |  | 23152 | 296.7 | 299.1 | 301.2 | 311.7 | 320.2 |
|  |  | 23153 | 266.8 | 266.7 | 270.5 | 275.4 | 276.2 |
|  |  | 23154 | 300.6 | 302.8 | 318.4 | 328.5 | 326.9 |
|  |  | 23155 | 252.9 | 254.6 | 276.2 | 269.1 | 275.3 |

### Individual animal data: body weight

| **Dose** | **Gender** | **Animal no.** | **Body weight (g)** | | | |
| --- | --- | --- | --- | --- | --- | --- |
| **g/kg** |  |  | **D70** | **D77** | **D84** | **D91** |
| 0 | Male | 10001 | 635.7 | 652.9 | 670.8 | 664.2 |
|  |  | 10002 | 612.3 | 624.0 | 631.4 | 633.7 |
|  |  | 10003 | 504.6 | 510.5 | 521.0 | 524.3 |
|  |  | 10004 | 642.9 | 656.6 | 672.2 | 681.4 |
|  |  | 10005 | 542.0 | 535.8 | 548.3 | 531.8 |
|  |  | 10006 | 664.5 | 673.9 | 693.6 | 690.9 |
|  |  | 10007 | 631.8 | 639.3 | 656.1 | 667.5 |
|  |  | 10008 | 586.2 | 600.1 | 626.4 | 621.8 |
|  |  | 10009 | 581.7 | 594.3 | 604.4 | 608.1 |
|  |  | 10010 | 526.4 | 530.8 | 542.3 | 545.2 |
|  |  | 10011 | 601.6 | 609.6 | 621.9 | 624.3 |
|  |  | 10012 | 645.0 | 660.8 | 675.8 | 676.1 |
|  |  | 10013 | 491.6 | 502.2 | 506.8 | 512.3 |
|  |  | 10014 | 620.8 | 635.2 | 657.0 | 664.6 |
|  |  | 10015 | 607.7 | 626.1 | 636.2 | 650.3 |
|  | Female | 20021 | 276.5 | 278.1 | 279.9 | 276.7 |
|  |  | 20022 | 309.3 | 308.7 | 314.2 | 314.2 |
|  |  | 20023 | 318.2 | 331.8 | 343.6 | 346.4 |
|  |  | 20024 | 300.4 | 312.6 | 327.1 | 324.6 |
|  |  | 20025 | 306.6 | 304.6 | 296.1 | 303.9 |
|  |  | 20026 | 292.3 | 286.3 | 301.3 | 299.6 |
|  |  | 20027 | 321.4 | 318.9 | 329.7 | 329.4 |
|  |  | 20028 | 279.4 | 282.6 | 283.5 | 285.6 |
|  |  | 20029 | 297.6 | 296.2 | 300.6 | 293.6 |
|  |  | 20030 | 330.3 | 338.6 | 346.5 | 350.1 |
|  |  | 20031 | 303.5 | 302.0 | 311.6 | 317.6 |
|  |  | 20032 | 290.0 | 291.9 | 299.5 | 301.3 |
|  |  | 20033 | 297.6 | 298.0 | 302.2 | 304.6 |
|  |  | 20034 | 357.1 | 363.0 | 368.9 | 368.5 |
|  |  | 20035 | 270.5 | 277.2 | 281.4 | 280.5 |

### Individual animal data: body weight

| **Dose** | **Gender** | **Animal no.** | **Body weight (g)** | | | |
| --- | --- | --- | --- | --- | --- | --- |
| **g/kg** |  |  | **D70** | **D77** | **D84** | **D91** |
| 0.5 | Male | 11041 | 540.0 | 551.9 | 562.3 | 565.0 |
|  |  | 11042 | 588.1 | 600.9 | 624.9 | 630.7 |
|  |  | 11043 | 553.2 | 563.2 | 570.2 | 578.0 |
|  |  | 11044 | 529.8 | 533.4 | 543.0 | 545.5 |
|  |  | 11045 | 632.4 | 647.2 | 661.8 | 676.0 |
|  |  | 11046 | 578.3 | 590.2 | 597.4 | 599.4 |
|  |  | 11047 | 555.7 | 564.0 | 576.6 | 588.1 |
|  |  | 11048 | 489.2 | 497.4 | 511.4 | 512.4 |
|  |  | 11049 | 588.5 | 606.2 | 629.8 | 640.6 |
|  |  | 11050 | 661.0 | 678.7 | 696.7 | 694.2 |
|  |  | 11051 | 611.5 | 613.7 | 628.0 | 636.7 |
|  |  | 11052 | 561.3 | 570.1 | 587.2 | 578.6 |
|  |  | 11053 | 565.4 | 574.6 | 586.0 | 595.3 |
|  |  | 11054 | 647.2 | 656.4 | 681.9 | 698.6 |
|  |  | 11055 | 547.7 | 561.4 | 569.6 | 577.8 |
|  | Female | 21061 | 262.2 | 261.5 | 278.9 | 266.2 |
|  |  | 21062 | 281.0 | 282.2 | 287.6 | 274.9 |
|  |  | 21063 | 268.6 | 277.3 | 282.8 | 272.6 |
|  |  | 21064 | 289.9 | 291.1 | 290.5 | 292.4 |
|  |  | 21065 | 313.4 | 316.9 | 318.2 | 320.6 |
|  |  | 21066 | 279.9 | 285.7 | 297.3 | 291.3 |
|  |  | 21067 | 326.4 | 335.0 | 341.1 | 336.3 |
|  |  | 21068 | 276.1 | 286.3 | 288.4 | 284.9 |
|  |  | 21069 | 304.7 | 307.7 | 319.3 | 313.3 |
|  |  | 21070 | 264.3 | 273.5 | 274.3 | 270.2 |
|  |  | 21071 | 312.8 | 312.2 | 321.1 | 321.3 |
|  |  | 21072 | 302.7 | 305.5 | 316.5 | 314.4 |
|  |  | 21073 | 306.1 | 301.5 | 311.8 | 303.2 |
|  |  | 21074 | 304.5 | 303.8 | 312.3 | 317.5 |
|  |  | 21075 | 296.8 | 300.3 | 303.2 | 299.0 |

### Individual animal data: body weight

| **Dose** | **Gender** | **Animal no.** | **Body weight (g)** | | | |
| --- | --- | --- | --- | --- | --- | --- |
| **g/kg** |  |  | **D70** | **D77** | **D84** | **D91** |
| 1.5 | Male | 12081 | 599.4 | 606.7 | 614.0 | 624.4 |
|  |  | 12082 | 597.5 | 612.1 | 629.1 | 640.7 |
|  |  | 12083 | 544.5 | 553.6 | 568.7 | 579.2 |
|  |  | 12084 | 575.7 | 585.7 | 594.4 | 593.2 |
|  |  | 12085 | 570.5 | 579.7 | 590.8 | 594.6 |
|  |  | 12086 | 482.4 | 489.9 | 494.5 | 506.6 |
|  |  | 12087 | 619.5 | 628.2 | 650.9 | 637.0 |
|  |  | 12088 | 486.5 | 499.6 | 501.4 | 508.4 |
|  |  | 12089 | 536.7 | 543.3 | 549.6 | 549.0 |
|  |  | 12090 | 667.3 | 678.4 | 673.9 | 677.0 |
|  |  | 12091 | 610.7 | 625.6 | 628.8 | 630.4 |
|  |  | 12092 | 568.9 | 582.1 | 588.7 | 595.4 |
|  |  | 12093 | 580.2 | 597.3 | 615.1 | 620.6 |
|  |  | 12094 | 597.3 | 603.3 | 614.1 | 618.9 |
|  |  | 12095 | 591.3 | 602.2 | 614.8 | 621.4 |
|  | Female | 22101 | 274.3 | 267.2 | 274.3 | 269.9 |
|  |  | 22102 | 295.2 | 291.1 | 297.2 | 286.5 |
|  |  | 22103 | 288.4 | 293.1 | 301.7 | 296.0 |
|  |  | 22104 | 264.0 | 265.5 | 269.5 | 270.8 |
|  |  | 22105 | 256.4 | 259.5 | 269.3 | 263.7 |
|  |  | 22106 | 319.2 | 322.9 | 323.0 | 331.2 |
|  |  | 22107 | 294.8 | 301.5 | 314.2 | 308.0 |
|  |  | 22108 | 307.8 | 313.3 | 312.5 | 306.1 |
|  |  | 22109 | 313.5 | 311.9 | 324.2 | 316.4 |
|  |  | 22110 | 306.0 | 306.5 | 319.2 | 317.5 |
|  |  | 22111 | 294.6 | 298.8 | 305.9 | 311.0 |
|  |  | 22112 | 321.1 | 322.6 | 324.5 | 324.8 |
|  |  | 22113 | 318.4 | 328.4 | 330.9 | 325.1 |
|  |  | 22114 | 314.7 | 321.5 | 338.1 | 341.9 |
|  |  | 22115 | 316.1 | 310.6 | 320.7 | 322.7 |

### Individual animal data: body weight

| **Dose** | **Gender** | **Animal no.** | **Body weight (g)** | | | |
| --- | --- | --- | --- | --- | --- | --- |
| **g/kg** |  |  | **D70** | **D77** | **D84** | **D91** |
| 5 | Male | 13121 | 537.5 | 548.9 | 546.9 | 542.7 |
|  |  | 13122 | 551.4 | 561.7 | 568.5 | 582.3 |
|  |  | 13123 | 590.5 | 607.0 | 599.0 | 601.3 |
|  |  | 13124 | 556.3 | 558.1 | 563.0 | 558.6 |
|  |  | 13125 | 527.8 | 541.1 | 550.5 | 544.8 |
|  |  | 13126 | 576.0 | 587.1 | 584.7 | 599.6 |
|  |  | 13127 | 555.4 | 572.0 | 579.9 | 596.9 |
|  |  | 13128 | 620.7 | 621.8 | 628.2 | 627.5 |
|  |  | 13129 | 685.5 | 673.4 | 685.8 | 702.6 |
|  |  | 13130 | 557.4 | 571.7 | 572.1 | 589.1 |
|  |  | 13131 | 609.2 | 622.5 | 637.2 | 639.9 |
|  |  | 13132 | 564.4 | 578.8 | 578.9 | 584.6 |
|  |  | 13133 | 579.3 | 591.6 | 593.7 | 597.5 |
|  |  | 13134 | 498.7 | 516.0 | 528.5 | 539.2 |
|  |  | 13135 | 519.4 | 526.1 | 538.4 | 543.3 |
|  | Female | 23141 | 281.9 | 288.6 | 293.4 | 291.8 |
|  |  | 23142 | 267.2 | 264.2 | 268.7 | 268.8 |
|  |  | 23143 | 282.9 | 279.8 | 296.1 | 297.7 |
|  |  | 23144 | 320.4 | 331.9 | 343.3 | 344.9 |
|  |  | 23145 | 332.5 | 326.6 | 330.2 | 334.6 |
|  |  | 23146 | 317.5 | 320.0 | 308.6 | 317.2 |
|  |  | 23147 | 287.8 | 278.0 | 284.8 | 284.9 |
|  |  | 23148 | 305.8 | 320.0 | 331.6 | 327.8 |
|  |  | 23149 | 309.7 | 319.5 | 315.1 | 309.8 |
|  |  | 23150 | 306.4 | 309.8 | 313.0 | 314.6 |
|  |  | 23151 | 323.1 | 332.4 | 336.9 | 334.0 |
|  |  | 23152 | 326.0 | 319.6 | 331.7 | 338.1 |
|  |  | 23153 | 276.4 | 273.6 | 282.9 | 279.3 |
|  |  | 23154 | 331.1 | 339.9 | 344.3 | 343.1 |
|  |  | 23155 | 269.7 | 277.6 | 271.3 | 284.6 |

### Individual animal data: body weight

| **Dose** | **Gender** | **Animal no.** | **Body weight (g)** | | | |
| --- | --- | --- | --- | --- | --- | --- |
| **g/kg** |  |  | **rD7** | **rD14** | **rD21** | **rD28** |
| 0 | Male | 10011 | 637.1 | 645.7 | 662.2 | 675.0 |
|  |  | 10012 | 689.5 | 697.7 | 716.3 | 729.0 |
|  |  | 10013 | 519.0 | 536.5 | 550.2 | 558.8 |
|  |  | 10014 | 671.6 | 685.4 | 705.6 | 726.6 |
|  |  | 10015 | 663.2 | 675.6 | 694.6 | 697.1 |
|  | Female | 20031 | 319.3 | 320.9 | 329.2 | 330.5 |
|  |  | 20032 | 300.4 | 301.1 | 307.0 | 305.7 |
|  |  | 20033 | 307.1 | 314.4 | 314.0 | 315.7 |
|  |  | 20034 | 366.0 | 372.7 | 377.4 | 379.8 |
|  |  | 20035 | 283.3 | 280.2 | 283.5 | 284.5 |
| 0.5 | Male | 11051 | 641.2 | 641.8 | 660.1 | 666.4 |
|  |  | 11052 | 586.8 | 597.1 | 613.0 | 623.6 |
|  |  | 11053 | 596.2 | 606.5 | 625.0 | 639.9 |
|  |  | 11054 | 713.2 | 726.2 | 742.6 | 769.4 |
|  |  | 11055 | 586.8 | 604.5 | 612.1 | 627.1 |
|  | Female | 21071 | 318.1 | 324.7 | 325.7 | 328.2 |
|  |  | 21072 | 318.8 | 322.1 | 336.0 | 334.0 |
|  |  | 21073 | 310.1 | 309.4 | 321.6 | 321.8 |
|  |  | 21074 | 317.9 | 316.7 | 329.9 | 328.4 |
|  |  | 21075 | 304.4 | 312.2 | 313.0 | 304.6 |
| 1.5 | Male | 12091 | 640.1 | 625.1 | 665.8 | 677.3 |
|  |  | 12092 | 597.8 | 609.4 | 613.7 | 615.4 |
|  |  | 12093 | 628.4 | 635.9 | 645.0 | 653.6 |
|  |  | 12094 | 622.4 | 636.0 | 648.5 | 651.5 |
|  |  | 12095 | 640.0 | 642.2 | 656.0 | 664.0 |
|  | Female | 22111 | 312.4 | 316.4 | 318.6 | 318.9 |
|  |  | 22112 | 329.9 | 333.9 | 332.9 | 336.6 |
|  |  | 22113 | 330.6 | 346.2 | 344.0 | 340.6 |
|  |  | 22114 | 340.3 | 346.4 | 355.4 | 357.5 |
|  |  | 22115 | 327.2 | 328.5 | 332.9 | 332.7 |
| 5 | Male | 13131 | 654.5 | 659.6 | 685.6 | 693.5 |
|  |  | 13132 | 593.9 | 607.1 | 625.0 | 635.5 |
|  |  | 13133 | 607.9 | 624.9 | 646.3 | 658.5 |
|  |  | 13134 | 551.6 | 558.1 | 573.1 | 574.3 |
|  |  | 13135 | 548.4 | 560.2 | 567.3 | 576.8 |
|  | Female | 23151 | 329.3 | 324.4 | 346.0 | 337.8 |
|  |  | 23152 | 330.8 | 332.0 | 345.5 | 346.6 |
|  |  | 23153 | 278.2 | 284.7 | 293.2 | 289.9 |
|  |  | 23154 | 349.9 | 357.2 | 360.7 | 362.1 |
|  |  | 23155 | 278.2 | 274.5 | 283.0 | 277.8 |

### Food intake of individual animal

| **Gender** | **Dose** | **Animal no.** | **Food intake (g)** | | | |
| --- | --- | --- | --- | --- | --- | --- |
|  | **g/kg** |  | **W1** | **W2** | **W3** | **W4** |
| Male | 0 | 10001-10003 | 26.2 | 31.0 | 34.6 | 27.8 |
|  |  | 10004-10005 | 27.0 | 39.6 | 35.0 | 29.7 |
|  |  | 10006-10008 | 28.4 | 29.2 | 34.8 | 30.3 |
|  |  | 10009-10010 | 26.7 | 30.7 | 31.9 | 26.8 |
|  |  | 10011-10013 | 26.3 | 26.5 | 33.8 | 28.8 |
|  |  | 10014-10015 | 28.4 | 37.3 | 34.2 | 31.3 |
|  |  | 10016-10018 | 29.0 | 30.9 | 33.2 | 28.8 |
|  |  | 10019-10020 | 28.2 | 33.8 | 33.8 | 30.7 |
|  | 0.5 | 11041-11043 | 27.5 | 30.5 | 33.0 | 28.4 |
|  |  | 11044-11045 | 28.3 | 34.7 | 35.7 | 29.1 |
|  |  | 11046-11048 | 26.0 | 31.4 | 31.8 | 26.0 |
|  |  | 11049-11050 | 28.3 | 35.1 | 35.5 | 30.1 |
|  |  | 11051-11053 | 28.2 | 31.3 | 34.1 | 29.1 |
|  |  | 11054-11055 | 27.3 | 33.4 | 33.2 | 28.8 |
|  |  | 11056-11058 | 28.4 | 31.7 | 36.9 | 31.0 |
|  |  | 11059-11060 | 29.3 | 40.1 | 36.8 | 30.9 |
|  | 1.5 | 12081-12083 | 27.6 | 30.6 | 34.3 | 28.3 |
|  |  | 12084-12085 | 27.1 | 31.3 | 31.6 | 26.8 |
|  |  | 12086-12088 | 26.3 | 31.0 | 32.1 | 25.9 |
|  |  | 12089-12090 | 27.6 | 34.0 | 34.6 | 31.1 |
|  |  | 12091-12093 | 27.6 | 31.9 | 34.9 | 29.0 |
|  |  | 12094-12095 | 28.9 | 32.7 | 33.2 | 27.9 |
|  |  | 12096-12098 | 29.4 | 31.6 | 35.7 | 27.3 |
|  |  | 12099-12100 | 28.4 | 35.1 | 35.0 | 31.1 |
|  | 5 | 13121-13123 | 25.4 | 31.7 | 33.9 | 28.2 |
|  |  | 13124-13125 | 25.5 | 31.5 | 31.2 | 28.4 |
|  |  | 13126-13128 | 27.0 | 31.4 | 33.2 | 29.3 |
|  |  | 13129-13130 | 28.6 | 39.9 | 39.9 | 33.0 |
|  |  | 13131-13133 | 27.5 | 31.8 | 35.6 | 29.3 |
|  |  | 13134-13135 | 26.3 | 29.6 | 31.3 | 25.7 |
|  |  | 13136-13138 | 28.4 | 31.8 | 37.5 | 32.5 |
|  |  | 13139-13140 | 26.0 | 32.8 | 32.9 | 26.5 |

### Food intake of individual animal

| **Gender** | **Dose** | **Animal no.** | **Food intake (g)** | | | |
| --- | --- | --- | --- | --- | --- | --- |
|  | **g/kg** |  | **W1** | **W2** | **W3** | **W4** |
| Female | 0 | 20021-20023 | 18.7 | 20.5 | 20.5 | 17.8 |
|  |  | 20024-20025 | 19.3 | 22.2 | 22.2 | 19.2 |
|  |  | 20026-20028 | 19.2 | 20.9 | 20.1 | 18.2 |
|  |  | 20029-20030 | 19.3 | 22.8 | 23.3 | 19.4 |
|  |  | 20031-20033 | 18.1 | 19.2 | 19.7 | 17.0 |
|  |  | 20034-20035 | 17.4 | 21.4 | 20.7 | 18.2 |
|  |  | 20036-20038 | 19.0 | 21.7 | 19.7 | 17.9 |
|  |  | 20039-20040 | 16.6 | 20.7 | 21.2 | 16.9 |
|  | 0.5 | 21061-21063 | 17.4 | 18.1 | 18.3 | 15.7 |
|  |  | 21064-21065 | 17.4 | 20.9 | 20.4 | 16.4 |
|  |  | 21066-21068 | 17.3 | 20.0 | 20.0 | 17.0 |
|  |  | 21069-21070 | 19.2 | 20.6 | 21.0 | 18.3 |
|  |  | 21071-21073 | 18.2 | 20.2 | 20.0 | 17.9 |
|  |  | 21074-21075 | 19.0 | 22.1 | 22.7 | 18.2 |
|  |  | 21076-21078 | 19.2 | 21.3 | 20.3 | 18.3 |
|  |  | 21079-21080 | 19.5 | 22.6 | 22.0 | 19.5 |
|  | 1.5 | 22101-22103 | 17.2 | 18.4 | 19.3 | 17.4 |
|  |  | 22104-22105 | 14.8 | 16.4 | 17.2 | 15.8 |
|  |  | 22106-22108 | 19.0 | 20.8 | 20.8 | 18.0 |
|  |  | 22109-22110 | 20.9 | 23.4 | 22.8 | 19.3 |
|  |  | 22111-22113 | 17.3 | 20.7 | 20.2 | 18.7 |
|  |  | 22114-22115 | 18.5 | 20.7 | 19.1 | 18.8 |
|  |  | 22116-22118 | 19.6 | 21.8 | 22.9 | 19.2 |
|  |  | 22119-22120 | 20.6 | 23.5 | 25.5 | 20.1 |
|  | 5 | 23141-23143 | 16.5 | 18.9 | 18.5 | 16.1 |
|  |  | 23144-23145 | 19.5 | 21.6 | 22.9 | 18.2 |
|  |  | 23146-23148 | 17.3 | 18.6 | 19.9 | 16.7 |
|  |  | 23149-23150 | 18.0 | 22.6 | 18.1 | 18.4 |
|  |  | 23151-23153 | 18.6 | 21.5 | 21.6 | 18.0 |
|  |  | 23154-23155 | 17.2 | 20.2 | 20.3 | 16.6 |
|  |  | 23156-23158 | 17.9 | 21.3 | 21.3 | 17.7 |
|  |  | 23159-23160 | 19.9 | 24.2 | 22.5 | 18.8 |

### Food intake of individual animal

| **Gender** | **Dose** | **Animal no.** | **Food intake (g)** | | | | |
| --- | --- | --- | --- | --- | --- | --- | --- |
|  | **g/kg** |  | **W5** | **W6** | **W7** | **W8** | **W9** |
| Male | 0 | 10001-10003 | 35.7 | 35.1 | 34.7 | 32.4 | 32.5 |
|  |  | 10004-10005 | 36.0 | 37.5 | 36.8 | 34.5 | 34.7 |
|  |  | 10006-10008 | 39.5 | 37.7 | 35.0 | 35.7 | 36.2 |
|  |  | 10009-10010 | 34.2 | 32.5 | 32.0 | 30.2 | 30.8 |
|  |  | 10011-10013 | 35.3 | 35.5 | 34.3 | 32.7 | 33.0 |
|  |  | 10014-10015 | 37.9 | 36.8 | 37.5 | 36.0 | 35.9 |
|  | 0.5 | 11041-11043 | 34.9 | 35.7 | 34.8 | 31.8 | 32.5 |
|  |  | 11044-11045 | 35.6 | 37.0 | 36.9 | 34.0 | 34.1 |
|  |  | 11046-11048 | 31.9 | 31.9 | 33.1 | 30.0 | 30.4 |
|  |  | 11049-11050 | 36.4 | 38.2 | 38.4 | 36.0 | 36.6 |
|  |  | 11051-11053 | 34.9 | 33.7 | 34.8 | 32.4 | 32.6 |
|  |  | 11054-11055 | 36.3 | 38.2 | 37.6 | 36.2 | 37.0 |
|  | 1.5 | 12081-12083 | 34.1 | 35.7 | 33.9 | 32.9 | 32.9 |
|  |  | 12084-12085 | 32.0 | 34.0 | 33.5 | 33.2 | 30.0 |
|  |  | 12086-12088 | 30.1 | 30.6 | 29.3 | 29.4 | 29.5 |
|  |  | 12089-12090 | 37.5 | 37.0 | 35.5 | 34.0 | 34.5 |
|  |  | 12091-12093 | 33.8 | 34.0 | 33.1 | 32.3 | 31.6 |
|  |  | 12094-12095 | 34.1 | 34.7 | 33.1 | 33.8 | 33.8 |
|  | 5 | 13121-13123 | 32.8 | 32.0 | 31.5 | 29.7 | 28.5 |
|  |  | 13124-13125 | 33.2 | 33.9 | 27.5 | 27.6 | 28.6 |
|  |  | 13126-13128 | 34.0 | 34.4 | 34.0 | 32.0 | 31.2 |
|  |  | 13129-13130 | 38.8 | 37.5 | 35.2 | 32.4 | 31.2 |
|  |  | 13131-13133 | 34.9 | 36.0 | 34.7 | 31.9 | 31.4 |
|  |  | 13134-13135 | 30.7 | 31.1 | 25.6 | 27.3 | 27.3 |

### Food intake of individual animal

| **Gender** | **Dose** | **Animal no.** | **Food intake (g)** | | | | |
| --- | --- | --- | --- | --- | --- | --- | --- |
|  | **g/kg** |  | **W5** | **W6** | **W7** | **W8** | **W9** |
| Female | 0 | 20021-20023 | 21.0 | 21.3 | 21.7 | 20.6 | 19.4 |
|  |  | 20024-20025 | 22.2 | 23.1 | 22.9 | 21.6 | 21.9 |
|  |  | 20026-20028 | 22.0 | 21.4 | 21.8 | 21.6 | 20.4 |
|  |  | 20029-20030 | 22.9 | 23.5 | 22.8 | 21.8 | 22.3 |
|  |  | 20031-20033 | 21.1 | 20.4 | 20.0 | 20.0 | 18.8 |
|  |  | 20034-20035 | 22.8 | 21.6 | 22.1 | 22.1 | 20.0 |
|  | 0.5 | 21061-21063 | 18.7 | 19.9 | 19.9 | 18.3 | 18.6 |
|  |  | 21064-21065 | 20.7 | 20.4 | 20.6 | 19.7 | 19.7 |
|  |  | 21066-21068 | 19.8 | 20.2 | 20.2 | 19.3 | 18.4 |
|  |  | 21069-21070 | 21.5 | 20.8 | 21.6 | 19.8 | 21.4 |
|  |  | 21071-21073 | 21.8 | 21.8 | 21.8 | 20.5 | 20.3 |
|  |  | 21074-21075 | 13.9 | 21.7 | 21.9 | 21.1 | 20.5 |
|  | 1.5 | 22101-22103 | 20.6 | 19.5 | 19.8 | 20.0 | 18.7 |
|  |  | 22104-22105 | 19.2 | 19.7 | 18.8 | 19.8 | 18.6 |
|  |  | 22106-22108 | 20.6 | 23.2 | 23.3 | 21.7 | 20.8 |
|  |  | 22109-22110 | 25.1 | 24.0 | 23.6 | 23.4 | 22.5 |
|  |  | 22111-22113 | 22.7 | 21.8 | 22.7 | 21.6 | 21.3 |
|  |  | 22114-22115 | 21.4 | 21.5 | 22.7 | 21.9 | 21.7 |
|  | 5 | 23141-23143 | 19.6 | 19.0 | 19.3 | 17.4 | 15.9 |
|  |  | 23144-23145 | 22.0 | 22.8 | 21.3 | 19.6 | 19.8 |
|  |  | 23146-23148 | 21.0 | 20.9 | 20.4 | 20.0 | 18.3 |
|  |  | 23149-23150 | 22.5 | 21.8 | 20.7 | 20.5 | 18.0 |
|  |  | 23151-23153 | 21.5 | 20.2 | 20.5 | 20.5 | 17.4 |
|  |  | 23154-23155 | 20.9 | 20.5 | 21.6 | 20.6 | 17.5 |

### Food intake of individual animal

| **Gender** | **Dose** | **Animal no.** | **Food intake (g)** | | | |
| --- | --- | --- | --- | --- | --- | --- |
|  | **g/kg** |  | **W10** | **W11** | **W12** | **W13** |
| Male | 0 | 10001-10003 | 31.2 | 31.9 | 31.8 | 30.7 |
|  |  | 10004-10005 | 33.0 | 32.0 | 32.2 | 31.3 |
|  |  | 10006-10008 | 35.1 | 36.0 | 36.4 | 35.6 |
|  |  | 10009-10010 | 29.6 | 30.6 | 29.6 | 30.1 |
|  |  | 10011-10013 | 32.1 | 31.3 | 30.6 | 29.8 |
|  |  | 10014-10015 | 33.9 | 34.7 | 34.2 | 35.9 |
|  | 0.5 | 11041-11043 | 32.8 | 34.0 | 34.0 | 33.4 |
|  |  | 11044-11045 | 33.3 | 36.3 | 35.7 | 35.6 |
|  |  | 11046-11048 | 30.1 | 30.6 | 30.3 | 30.5 |
|  |  | 11049-11050 | 33.4 | 35.6 | 35.7 | 34.9 |
|  |  | 11051-11053 | 31.8 | 32.6 | 32.0 | 32.3 |
|  |  | 11054-11055 | 34.8 | 34.9 | 36.0 | 37.0 |
|  | 1.5 | 12081-12083 | 30.0 | 30.2 | 31.0 | 30.9 |
|  |  | 12084-12085 | 29.2 | 31.8 | 29.1 | 29.3 |
|  |  | 12086-12088 | 26.7 | 30.1 | 27.6 | 28.8 |
|  |  | 12089-12090 | 31.6 | 34.1 | 29.9 | 32.8 |
|  |  | 12091-12093 | 30.8 | 33.0 | 30.8 | 32.0 |
|  |  | 12094-12095 | 34.5 | 35.3 | 31.9 | 33.5 |
|  | 5 | 13121-13123 | 27.1 | 26.2 | 26.0 | 27.7 |
|  |  | 13124-13125 | 27.4 | 26.8 | 26.6 | 27.3 |
|  |  | 13126-13128 | 29.8 | 29.3 | 28.9 | 29.7 |
|  |  | 13129-13130 | 32.0 | 30.0 | 29.5 | 32.5 |
|  |  | 13131-13133 | 30.8 | 31.6 | 30.5 | 31.6 |
|  |  | 13134-13135 | 26.6 | 25.8 | 26.8 | 27.8 |

### Food intake of individual animal

| **Gender** | **Dose** | **Animal no.** | **Food intake (g)** | | | |
| --- | --- | --- | --- | --- | --- | --- |
|  | **g/kg** |  | **W10** | **W11** | **W12** | **W13** |
| Female | 0 | 20021-20023 | 20.0 | 21.4 | 20.2 | 20.1 |
|  |  | 20024-20025 | 20.7 | 21.8 | 22.0 | 21.9 |
|  |  | 20026-20028 | 20.4 | 21.1 | 21.5 | 20.6 |
|  |  | 20029-20030 | 22.0 | 22.0 | 22.6 | 22.4 |
|  |  | 20031-20033 | 19.0 | 19.9 | 19.7 | 19.4 |
|  |  | 20034-20035 | 21.0 | 20.7 | 19.9 | 19.9 |
|  | 0.5 | 21061-21063 | 18.4 | 19.6 | 18.6 | 17.4 |
|  |  | 21064-21065 | 19.4 | 20.0 | 19.5 | 20.5 |
|  |  | 21066-21068 | 19.3 | 20.0 | 20.8 | 20.0 |
|  |  | 21069-21070 | 21.0 | 19.8 | 20.9 | 21.1 |
|  |  | 21071-21073 | 20.2 | 20.5 | 21.3 | 20.5 |
|  |  | 21074-21075 | 20.6 | 21.3 | 20.2 | 21.0 |
|  | 1.5 | 22101-22103 | 19.2 | 19.3 | 18.5 | 17.2 |
|  |  | 22104-22105 | 19.0 | 20.6 | 19.1 | 18.9 |
|  |  | 22106-22108 | 20.2 | 21.4 | 21.1 | 21.3 |
|  |  | 22109-22110 | 20.0 | 22.7 | 23.9 | 21.9 |
|  |  | 22111-22113 | 21.1 | 21.4 | 21.5 | 21.3 |
|  |  | 22114-22115 | 21.0 | 23.4 | 20.9 | 22.5 |
|  | 5 | 23141-23143 | 16.8 | 17.6 | 17.4 | 18.6 |
|  |  | 23144-23145 | 19.3 | 19.8 | 19.7 | 21.2 |
|  |  | 23146-23148 | 18.4 | 18.9 | 18.6 | 19.3 |
|  |  | 23149-23150 | 19.9 | 18.2 | 20.0 | 17.7 |
|  |  | 23151-23153 | 18.9 | 18.2 | 18.1 | 19.2 |
|  |  | 23154-23155 | 17.3 | 19.4 | 19.3 | 20.7 |

### Food intake of individual animal

| **Gender** | **Dose** | **Animal no.** | **Food intake (g)** | | | |
| --- | --- | --- | --- | --- | --- | --- |
|  | **g/kg** |  | **rW1** | **rW2** | **rW3** | **rW4** |
| Male | 0 | 10011-10013 | 29.7 | 30.7 | 32.8 | 32.9 |
|  |  | 10014-10015 | 32.6 | 35.2 | 36.7 | 36.9 |
|  | 0.5 | 11051-11053 | 29.6 | 30.4 | 31.5 | 32.6 |
|  |  | 11054-11055 | 34.6 | 36.4 | 36.4 | 37.4 |
|  | 1.5 | 12091-12093 | 29.6 | 29.7 | 30.6 | 30.0 |
|  |  | 12094-12095 | 32.3 | 31.4 | 33.6 | 33.4 |
|  | 5 | 13131-13133 | 32.1 | 31.5 | 34.4 | 33.9 |
|  |  | 13134-13135 | 29.3 | 31.5 | 28.1 | 28.1 |
| Female | 0 | 20031-20033 | 17.9 | 18.4 | 19.4 | 18.5 |
|  |  | 20034-20035 | 18.6 | 19.6 | 20.8 | 19.6 |
|  | 0.5 | 21071-21073 | 19.7 | 20.8 | 22.3 | 22.2 |
|  |  | 21074-21075 | 19.6 | 20.6 | 22.3 | 21.1 |
|  | 1.5 | 22111-22113 | 18.7 | 19.6 | 19.4 | 20.3 |
|  |  | 22114-22115 | 20.8 | 21.7 | 21.9 | 21.3 |
|  | 5 | 23151-23153 | 19.3 | 20.0 | 20.4 | 19.6 |
|  |  | 23154-23155 | 20.3 | 21.5 | 22.6 | 22.2 |

### Ophthalmology data of individual animal

| **Dose** | **Gender** | **Animal no.** | **Ophthalmological examination result** | | | |
| --- | --- | --- | --- | --- | --- | --- |
| **g/kg** |  |  | **D-2** | **D28** | **D91** | **rD28** |
| 0 | Male | 10001 | Normal |  | Normal |  |
|  |  | 10002 | Normal |  | Normal |  |
|  |  | 10003 | Normal |  | Normal |  |
|  |  | 10004 | Normal |  | Normal |  |
|  |  | 10005 | Normal |  | Normal |  |
|  |  | 10006 | Normal |  | Normal |  |
|  |  | 10007 | Normal |  | Normal |  |
|  |  | 10008 | Normal |  | Normal |  |
|  |  | 10009 | Normal |  | Normal |  |
|  |  | 10010 | Normal |  | Normal |  |
|  |  | 10011 | Normal |  |  | Normal |
|  |  | 10012 | Normal |  |  | Normal |
|  |  | 10013 | Normal |  |  | Normal |
|  |  | 10014 | Normal |  |  | Normal |
|  |  | 10015 | Normal |  |  | Normal |
|  |  | 10016 | Normal | Normal |  |  |
|  |  | 10017 | Normal | Normal |  |  |
|  |  | 10018 | Normal | Normal |  |  |
|  |  | 10019 | Normal | Normal |  |  |
|  |  | 10020 | Normal | Normal |  |  |
|  | Female | 20021 | Normal |  | Normal |  |
|  |  | 20022 | Normal |  | Normal |  |
|  |  | 20023 | Normal |  | Normal |  |
|  |  | 20024 | Normal |  | Normal |  |
|  |  | 20025 | Normal |  | Normal |  |
|  |  | 20026 | Normal |  | Normal |  |
|  |  | 20027 | Normal |  | Normal |  |
|  |  | 20028 | Normal |  | Normal |  |
|  |  | 20029 | Normal |  | Normal |  |
|  |  | 20030 | Normal |  | Normal |  |
|  |  | 20031 | Normal |  |  | Normal |
|  |  | 20032 | Normal |  |  | Normal |
|  |  | 20033 | Normal |  |  | Normal |
|  |  | 20034 | Normal |  |  | Normal |
|  |  | 20035 | Normal |  |  | Normal |
|  |  | 20036 | Normal | Normal |  |  |
|  |  | 20037 | Normal | Normal |  |  |
|  |  | 20038 | Normal | Normal |  |  |
|  |  | 20039 | Normal | Normal |  |  |
|  |  | 20040 | Normal | Normal |  |  |

Note: Examination items included eyelid, conjunctiva, cornea, sclera, iris, pupil, lens, vitreous bodies, and fundus.

### Ophthalmology data of individual animal

| **Dose** | **Gender** | **Animal no.** | **Ophthalmological examination result** |
| --- | --- | --- | --- |
| **g/kg** |  |  | **D-2** |
| 0.5 | Male | 11041 | Normal |
|  |  | 11042 | Normal |
|  |  | 11043 | Normal |
|  |  | 11044 | Normal |
|  |  | 11045 | Normal |
|  |  | 11046 | Normal |
|  |  | 11047 | Normal |
|  |  | 11048 | Normal |
|  |  | 11049 | Normal |
|  |  | 11050 | Normal |
|  |  | 11051 | Normal |
|  |  | 11052 | Normal |
|  |  | 11053 | Normal |
|  |  | 11054 | Normal |
|  |  | 11055 | Normal |
|  |  | 11056 | Normal |
|  |  | 11057 | Normal |
|  |  | 11058 | Normal |
|  |  | 11059 | Normal |
|  |  | 11060 | Normal |
|  | Female | 21061 | Normal |
|  |  | 21062 | Normal |
|  |  | 21063 | Normal |
|  |  | 21064 | Normal |
|  |  | 21065 | Normal |
|  |  | 21066 | Normal |
|  |  | 21067 | Normal |
|  |  | 21068 | Normal |
|  |  | 21069 | Normal |
|  |  | 21070 | Normal |
|  |  | 21071 | Normal |
|  |  | 21072 | Normal |
|  |  | 21073 | Normal |
|  |  | 21074 | Normal |
|  |  | 21075 | Normal |
|  |  | 21076 | Normal |
|  |  | 21077 | Normal |
|  |  | 21078 | Normal |
|  |  | 21079 | Normal |
|  |  | 21080 | Normal |

Note: Examination items included eyelid, conjunctiva, cornea, sclera, iris, pupil, lens, vitreous bodies, and fundus.

### Ophthalmology data of individual animal

| **Dose** | **Gender** | **Animal no.** | **Ophthalmological examination result** |
| --- | --- | --- | --- |
| **g/kg** |  |  | **D-2** |
| 1.5 | Male | 12081 | Normal |
|  |  | 12082 | Normal |
|  |  | 12083 | Normal |
|  |  | 12084 | Normal |
|  |  | 12085 | Normal |
|  |  | 12086 | Normal |
|  |  | 12087 | Normal |
|  |  | 12088 | Normal |
|  |  | 12089 | Normal |
|  |  | 12090 | Normal |
|  |  | 12091 | Normal |
|  |  | 12092 | Normal |
|  |  | 12093 | Normal |
|  |  | 12094 | Normal |
|  |  | 12095 | Normal |
|  |  | 12096 | Normal |
|  |  | 12097 | Normal |
|  |  | 12098 | Normal |
|  |  | 12099 | Normal |
|  |  | 12100 | Normal |
|  | Female | 22101 | Normal |
|  |  | 22102 | Normal |
|  |  | 22103 | Normal |
|  |  | 22104 | Normal |
|  |  | 22105 | Normal |
|  |  | 22106 | Normal |
|  |  | 22107 | Normal |
|  |  | 22108 | Normal |
|  |  | 22109 | Normal |
|  |  | 22110 | Normal |
|  |  | 22111 | Normal |
|  |  | 22112 | Normal |
|  |  | 22113 | Normal |
|  |  | 22114 | Normal |
|  |  | 22115 | Normal |
|  |  | 22116 | Normal |
|  |  | 22117 | Normal |
|  |  | 22118 | Normal |
|  |  | 22119 | Normal |
|  |  | 22120 | Normal |

Note: Examination items included eyelid, conjunctiva, cornea, sclera, iris, pupil, lens, vitreous bodies, and fundus.

### Ophthalmology data of individual animal

| **Dose** | **Gender** | **Animal no.** | **Ophthalmological examination result** | | | |
| --- | --- | --- | --- | --- | --- | --- |
| **g/kg** |  |  | **D-2** | **D28** | **D91** | **rD28** |
| 5 | Male | 13121 | Normal |  | Normal |  |
|  |  | 13122 | Normal |  | Normal |  |
|  |  | 13123 | Normal |  | Normal |  |
|  |  | 13124 | Normal |  | Normal |  |
|  |  | 13125 | Normal |  | Normal |  |
|  |  | 13126 | Normal |  | Normal |  |
|  |  | 13127 | Normal |  | Normal |  |
|  |  | 13128 | Normal |  | Normal |  |
|  |  | 13129 | Normal |  | Normal |  |
|  |  | 13130 | Normal |  | Normal |  |
|  |  | 13131 | Normal |  |  | Normal |
|  |  | 13132 | Normal |  |  | Normal |
|  |  | 13133 | Normal |  |  | Normal |
|  |  | 13134 | Normal |  |  | Normal |
|  |  | 13135 | Normal |  |  | Normal |
|  |  | 13136 | Normal | Normal |  |  |
|  |  | 13137 | Normal | Normal |  |  |
|  |  | 13138 | Normal | Normal |  |  |
|  |  | 13139 | Normal | Normal |  |  |
|  |  | 13140 | Normal | Normal |  |  |
|  | Female | 23141 | Normal |  | Normal |  |
|  |  | 23142 | Normal |  | Normal |  |
|  |  | 23143 | Normal |  | Normal |  |
|  |  | 23144 | Normal |  | Normal |  |
|  |  | 23145 | Normal |  | Normal |  |
|  |  | 23146 | Normal |  | Normal |  |
|  |  | 23147 | Normal |  | Normal |  |
|  |  | 23148 | Normal |  | Normal |  |
|  |  | 23149 | Normal |  | Normal |  |
|  |  | 23150 | Normal |  | Normal |  |
|  |  | 23151 | Normal |  |  | Normal |
|  |  | 23152 | Normal |  |  | Normal |
|  |  | 23153 | Normal |  |  | Normal |
|  |  | 23154 | Normal |  |  | Normal |
|  |  | 23155 | Normal |  |  | Normal |
|  |  | 23156 | Normal | Normal |  |  |
|  |  | 23157 | Normal | Normal |  |  |
|  |  | 23158 | Normal | Normal |  |  |
|  |  | 23159 | Normal | Normal |  |  |
|  |  | 23160 | Normal | Normal |  |  |

Note: Examination items included eyelid, conjunctiva, cornea, sclera, iris, pupil, lens, vitreous bodies, and fundus.

### Hematology data of individual animal

| **Hematology data on day 92** | | | | | | | |
| --- | --- | --- | --- | --- | --- | --- | --- |
| **Gender** | **Dose** | **Animal no.** | **WBC** | **NEUT#** | **LYMPH#** | **MONO#** | **EO#** |
|  | **g/kg** |  | **10^9^/L** | **10^9^/L** | **10^9^/L** | **10^9^/L** | **10^9^/L** |
| Male | 0 | 10001 | 7.37 | 0.92 | 6.29 | 0.08 | 0.08 |
|  |  | 10002 | 6.30 | 0.85 | 5.26 | 0.07 | 0.12 |
|  |  | 10003 | 6.85 | 0.52 | 6.26 | 0.03 | 0.04 |
|  |  | 10004 | 8.87 | 0.95 | 7.81 | 0.02 | 0.09 |
|  |  | 10005 | 5.12 | 0.38 | 4.59 | 0.04 | 0.11 |
|  |  | 10006 | 6.69 | 1.57 | 4.88 | 0.11 | 0.13 |
|  |  | 10007 | 6.22 | 1.21 | 4.89 | 0.05 | 0.07 |
|  |  | 10008 | 5.23 | 1.99 | 3.09 | 0.10 | 0.05 |
|  |  | 10009 | 7.22 | 1.27 | 5.76 | 0.13 | 0.06 |
|  |  | 10010 | 6.57 | 0.97 | 5.41 | 0.11 | 0.08 |
|  | 0.5 | 11041 | 5.98 | 0.89 | 5.03 | 0.03 | 0.03 |
|  |  | 11042 | 8.49 | 0.99 | 7.31 | 0.12 | 0.07 |
|  |  | 11043 | 6.61 | 0.61 | 5.91 | 0.05 | 0.04 |
|  |  | 11044 | 10.99 | 1.00 | 9.80 | 0.08 | 0.11 |
|  |  | 11045 | 4.99 | 1.12 | 3.84 | 0.00 | 0.03 |
|  |  | 11046 | 5.07 | 1.30 | 3.61 | 0.07 | 0.09 |
|  |  | 11047 | 4.98 | 1.05 | 3.76 | 0.08 | 0.09 |
|  |  | 11048 | 3.39 | 0.73 | 2.53 | 0.03 | 0.10 |
|  |  | 11049 | 9.64 | 1.60 | 7.94 | 0.06 | 0.04 |
|  |  | 11050 | 6.07 | 0.81 | 5.15 | 0.06 | 0.05 |
|  | 1.5 | 12081 | 7.51 | 0.77 | 6.61 | 0.06 | 0.07 |
|  |  | 12082 | 6.41 | 0.68 | 5.62 | 0.06 | 0.05 |
|  |  | 12083 | 5.39 | 0.73 | 4.60 | 0.01 | 0.05 |
|  |  | 12084 | 5.78 | 0.74 | 4.94 | 0.02 | 0.08 |
|  |  | 12085 | 10.72 | 0.79 | 9.74 | 0.09 | 0.10 |
|  |  | 12086 | 4.80 | 1.18 | 3.38 | 0.06 | 0.18 |
|  |  | 12087 | 5.21 | 0.78 | 4.24 | 0.11 | 0.08 |
|  |  | 12088 | 5.70 | 1.84 | 3.68 | 0.08 | 0.10 |
|  |  | 12089 | 7.57 | 1.27 | 6.14 | 0.06 | 0.09 |
|  |  | 12090 | 6.34 | 0.73 | 5.49 | 0.05 | 0.07 |
|  | 5 | 13121 | 9.90 | 1.06 | 8.69 | 0.09 | 0.06 |
|  |  | 13122 | 10.59 | 0.98 | 9.41 | 0.10 | 0.10 |
|  |  | 13123 | 8.37 | 0.97 | 7.21 | 0.09 | 0.10 |
|  |  | 13124 | 7.30 | 0.77 | 6.36 | 0.02 | 0.15 |
|  |  | 13125 | 7.44 | 0.90 | 6.38 | 0.04 | 0.12 |
|  |  | 13126 | 6.64 | 0.88 | 5.63 | 0.03 | 0.10 |
|  |  | 13127 | 5.86 | 0.84 | 4.79 | 0.11 | 0.12 |
|  |  | 13128 | 5.60 | 0.95 | 4.43 | 0.07 | 0.15 |
|  |  | 13129 | 6.53 | 1.05 | 5.37 | 0.05 | 0.06 |
|  |  | 13130 | 5.06 | 1.52 | 3.41 | 0.04 | 0.09 |

### Hematology data of individual animal

| **Hematology data on day 92** | | | | | | | |
| --- | --- | --- | --- | --- | --- | --- | --- |
| **Gender** | **Dose** | **Animal no.** | **BASO#** | **NEUT%** | **LYMPH%** | **MONO%** | **EO%** |
|  | **g/kg** |  | **10^9^/L** | **%** | **%** | **%** | **%** |
| Male | 0 | 10001 | 0.00 | 12.5 | 85.3 | 1.1 | 1.1 |
|  |  | 10002 | 0.00 | 13.5 | 83.5 | 1.1 | 1.9 |
|  |  | 10003 | 0.00 | 7.6 | 91.4 | 0.4 | 0.6 |
|  |  | 10004 | 0.00 | 10.8 | 88.0 | 0.2 | 1.0 |
|  |  | 10005 | 0.00 | 7.5 | 89.6 | 0.8 | 2.1 |
|  |  | 10006 | 0.00 | 23.6 | 72.9 | 1.6 | 1.9 |
|  |  | 10007 | 0.00 | 19.5 | 78.6 | 0.8 | 1.1 |
|  |  | 10008 | 0.00 | 38.0 | 59.1 | 1.9 | 1.0 |
|  |  | 10009 | 0.00 | 17.6 | 79.8 | 1.8 | 0.8 |
|  |  | 10010 | 0.00 | 14.8 | 82.3 | 1.7 | 1.2 |
|  | 0.5 | 11041 | 0.00 | 14.9 | 84.1 | 0.5 | 0.5 |
|  |  | 11042 | 0.00 | 11.7 | 86.1 | 1.4 | 0.8 |
|  |  | 11043 | 0.00 | 9.2 | 89.4 | 0.8 | 0.6 |
|  |  | 11044 | 0.00 | 9.1 | 89.2 | 0.7 | 1.0 |
|  |  | 11045 | 0.00 | 22.4 | 77.0 | 0.0 | 0.6 |
|  |  | 11046 | 0.00 | 25.6 | 71.2 | 1.4 | 1.8 |
|  |  | 11047 | 0.00 | 21.1 | 75.5 | 1.6 | 1.8 |
|  |  | 11048 | 0.00 | 21.6 | 74.6 | 0.9 | 2.9 |
|  |  | 11049 | 0.00 | 16.6 | 82.4 | 0.6 | 0.4 |
|  |  | 11050 | 0.00 | 13.4 | 84.8 | 1.0 | 0.8 |
|  | 1.5 | 12081 | 0.00 | 10.3 | 88.0 | 0.8 | 0.9 |
|  |  | 12082 | 0.00 | 10.6 | 87.7 | 0.9 | 0.8 |
|  |  | 12083 | 0.00 | 13.6 | 85.3 | 0.2 | 0.9 |
|  |  | 12084 | 0.00 | 12.8 | 85.5 | 0.3 | 1.4 |
|  |  | 12085 | 0.00 | 7.4 | 90.9 | 0.8 | 0.9 |
|  |  | 12086 | 0.00 | 24.5 | 70.4 | 1.3 | 3.8 |
|  |  | 12087 | 0.00 | 15.0 | 81.4 | 2.1 | 1.5 |
|  |  | 12088 | 0.00 | 32.2 | 64.6 | 1.4 | 1.8 |
|  |  | 12089 | 0.01 | 16.8 | 81.1 | 0.8 | 1.2 |
|  |  | 12090 | 0.00 | 11.5 | 86.6 | 0.8 | 1.1 |
|  | 5 | 13121 | 0.00 | 10.7 | 87.8 | 0.9 | 0.6 |
|  |  | 13122 | 0.00 | 9.3 | 88.9 | 0.9 | 0.9 |
|  |  | 13123 | 0.00 | 11.6 | 86.1 | 1.1 | 1.2 |
|  |  | 13124 | 0.00 | 10.5 | 87.1 | 0.3 | 2.1 |
|  |  | 13125 | 0.00 | 12.1 | 85.8 | 0.5 | 1.6 |
|  |  | 13126 | 0.00 | 13.2 | 84.8 | 0.5 | 1.5 |
|  |  | 13127 | 0.00 | 14.4 | 81.7 | 1.9 | 2.0 |
|  |  | 13128 | 0.00 | 16.9 | 79.1 | 1.3 | 2.7 |
|  |  | 13129 | 0.00 | 16.1 | 82.2 | 0.8 | 0.9 |
|  |  | 13130 | 0.00 | 30.0 | 67.4 | 0.8 | 1.8 |

### Hematology data of individual animal

| **Hematology data on day 92** | | | | | | | |
| --- | --- | --- | --- | --- | --- | --- | --- |
| **Gender** | **Dose** | **Animal no.** | **BASO%** | **RBC** | **HGB** | **HCT** | **MCV** |
|  | **g/kg** |  | **%** | **10^12^/L** | **g/L** | **%** | **fL** |
| Male | 0 | 10001 | 0.0 | 8.78 | 151 | 41.6 | 47.4 |
|  |  | 10002 | 0.0 | 9.39 | 164 | 45.8 | 48.8 |
|  |  | 10003 | 0.0 | 8.84 | 151 | 42.1 | 47.6 |
|  |  | 10004 | 0.0 | 8.96 | 158 | 45.2 | 50.4 |
|  |  | 10005 | 0.0 | 8.59 | 156 | 44.9 | 52.3 |
|  |  | 10006 | 0.0 | 9.04 | 159 | 44.3 | 49.0 |
|  |  | 10007 | 0.0 | 9.05 | 153 | 43.1 | 47.6 |
|  |  | 10008 | 0.0 | 8.62 | 159 | 43.8 | 50.8 |
|  |  | 10009 | 0.0 | 8.84 | 163 | 45.1 | 51.0 |
|  |  | 10010 | 0.0 | 8.88 | 165 | 46.3 | 52.1 |
|  | 0.5 | 11041 | 0.0 | 8.23 | 154 | 44.4 | 53.9 |
|  |  | 11042 | 0.0 | 8.97 | 159 | 44.6 | 49.7 |
|  |  | 11043 | 0.0 | 9.06 | 156 | 44.6 | 49.2 |
|  |  | 11044 | 0.0 | 9.66 | 170 | 46.8 | 48.4 |
|  |  | 11045 | 0.0 | 8.48 | 161 | 45.8 | 54.0 |
|  |  | 11046 | 0.0 | 8.74 | 164 | 45.9 | 52.5 |
|  |  | 11047 | 0.0 | 8.82 | 159 | 44.1 | 50.0 |
|  |  | 11048 | 0.0 | 8.85 | 158 | 43.4 | 49.0 |
|  |  | 11049 | 0.0 | 9.12 | 163 | 44.6 | 48.9 |
|  |  | 11050 | 0.0 | 9.16 | 160 | 45.1 | 49.2 |
|  | 1.5 | 12081 | 0.0 | 9.22 | 163 | 44.8 | 48.6 |
|  |  | 12082 | 0.0 | 8.63 | 152 | 44.1 | 51.1 |
|  |  | 12083 | 0.0 | 8.68 | 154 | 44.4 | 51.2 |
|  |  | 12084 | 0.0 | 8.78 | 155 | 43.8 | 49.9 |
|  |  | 12085 | 0.0 | 8.84 | 170 | 47.3 | 53.5 |
|  |  | 12086 | 0.0 | 8.80 | 159 | 44.1 | 50.1 |
|  |  | 12087 | 0.0 | 9.05 | 164 | 45.1 | 49.8 |
|  |  | 12088 | 0.0 | 8.73 | 163 | 45.4 | 52.0 |
|  |  | 12089 | 0.1 | 8.88 | 162 | 44.8 | 50.5 |
|  |  | 12090 | 0.0 | 8.74 | 157 | 44.3 | 50.7 |
|  | 5 | 13121 | 0.0 | 8.59 | 152 | 42.8 | 49.8 |
|  |  | 13122 | 0.0 | 8.99 | 163 | 44.8 | 49.8 |
|  |  | 13123 | 0.0 | 8.38 | 149 | 42.0 | 50.1 |
|  |  | 13124 | 0.0 | 9.18 | 164 | 45.8 | 49.9 |
|  |  | 13125 | 0.0 | 8.83 | 160 | 44.5 | 50.4 |
|  |  | 13126 | 0.0 | 8.90 | 164 | 45.3 | 50.9 |
|  |  | 13127 | 0.0 | 8.64 | 167 | 46.5 | 53.8 |
|  |  | 13128 | 0.0 | 9.05 | 158 | 44.2 | 48.8 |
|  |  | 13129 | 0.0 | 8.35 | 148 | 40.7 | 48.7 |
|  |  | 13130 | 0.0 | 8.57 | 161 | 44.8 | 52.3 |

### Hematology data of individual animal

| **Hematology data on day 92** | | | | | | | |
| --- | --- | --- | --- | --- | --- | --- | --- |
| **Gender** | **Dose** | **Animal no.** | **MCH** | **MCHC** | **PLT** | **RET%** | **RET#** |
|  | **g/kg** |  | **pg** | **g/L** | **10^9^/L** | **%** | **10^9^/L** |
| Male | 0 | 10001 | 17.2 | 363 | 1267 | 2.65 | 232.7 |
|  |  | 10002 | 17.5 | 358 | 1148 | 2.84 | 266.7 |
|  |  | 10003 | 17.1 | 359 | 1000 | 1.78 | 157.4 |
|  |  | 10004 | 17.6 | 350 | 1089 | 3.53 | 316.3 |
|  |  | 10005 | 18.2 | 347 | 1020 | 2.17 | 186.4 |
|  |  | 10006 | 17.6 | 359 | 1461 | 3.33 | 301.0 |
|  |  | 10007 | 16.9 | 355 | 1522 | 3.99 | 361.1 |
|  |  | 10008 | 18.4 | 363 | 1273 | 2.37 | 204.3 |
|  |  | 10009 | 18.4 | 361 | 1343 | 2.86 | 252.8 |
|  |  | 10010 | 18.6 | 356 | 1243 | 2.55 | 226.4 |
|  | 0.5 | 11041 | 18.7 | 347 | 1092 | 3.31 | 272.4 |
|  |  | 11042 | 17.7 | 357 | 1345 | 3.19 | 286.1 |
|  |  | 11043 | 17.2 | 350 | 1168 | 3.35 | 303.5 |
|  |  | 11044 | 17.6 | 363 | 1119 | 2.33 | 225.1 |
|  |  | 11045 | 19.0 | 352 | 1001 | 3.27 | 277.3 |
|  |  | 11046 | 18.8 | 357 | 1083 | 2.38 | 208.0 |
|  |  | 11047 | 18.0 | 361 | 1200 | 2.78 | 245.2 |
|  |  | 11048 | 17.9 | 364 | 1421 | 2.69 | 238.1 |
|  |  | 11049 | 17.9 | 365 | 1358 | 3.42 | 311.9 |
|  |  | 11050 | 17.5 | 355 | 1124 | 2.88 | 263.8 |
|  | 1.5 | 12081 | 17.7 | 364 | 1124 | 2.07 | 190.9 |
|  |  | 12082 | 17.6 | 345 | 1221 | 3.47 | 299.5 |
|  |  | 12083 | 17.7 | 347 | 1137 | 2.95 | 256.1 |
|  |  | 12084 | 17.7 | 354 | 1043 | 2.79 | 245.0 |
|  |  | 12085 | 19.2 | 359 | 904 | 2.35 | 207.7 |
|  |  | 12086 | 18.1 | 361 | 1124 | 3.07 | 270.2 |
|  |  | 12087 | 18.1 | 364 | 1092 | 2.01 | 181.9 |
|  |  | 12088 | 18.7 | 359 | 1068 | 2.19 | 191.2 |
|  |  | 12089 | 18.2 | 362 | 1228 | 2.18 | 193.6 |
|  |  | 12090 | 18.0 | 354 | 1060 | 3.02 | 263.9 |
|  | 5 | 13121 | 17.7 | 355 | 1173 | 2.28 | 195.9 |
|  |  | 13122 | 18.1 | 364 | 1117 | 3.02 | 271.5 |
|  |  | 13123 | 17.8 | 355 | 1202 | 3.56 | 298.3 |
|  |  | 13124 | 17.9 | 358 | 1152 | 2.21 | 202.9 |
|  |  | 13125 | 18.1 | 360 | 1293 | 2.45 | 216.3 |
|  |  | 13126 | 18.4 | 362 | 1102 | 2.74 | 243.9 |
|  |  | 13127 | 19.3 | 359 | 1017 | 2.83 | 244.5 |
|  |  | 13128 | 17.5 | 357 | 1138 | 2.93 | 265.2 |
|  |  | 13129 | 17.7 | 364 | 1146 | 2.60 | 217.1 |
|  |  | 13130 | 18.8 | 359 | 1226 | 2.81 | 240.8 |

### Hematology data of individual animal

| **Hematology data on day 92** | | | | | | | |
| --- | --- | --- | --- | --- | --- | --- | --- |
| **Gender** | **Dose** | **Animal no.** | **WBC** | **NEUT#** | **LYMPH#** | **MONO#** | **EO#** |
|  | **g/kg** |  | **10^9^/L** | **10^9^/L** | **10^9^/L** | **10^9^/L** | **10^9^/L** |
| Female | 0 | 20021 | 3.13 | 0.55 | 2.54 | 0.03 | 0.01 |
|  |  | 20022 | 3.73 | 1.32 | 2.32 | 0.07 | 0.02 |
|  |  | 20023 | 5.47 | 0.69 | 4.71 | 0.04 | 0.03 |
|  |  | 20024 | 3.72 | 0.47 | 3.19 | 0.04 | 0.02 |
|  |  | 20025 | 5.54 | 1.21 | 4.24 | 0.05 | 0.04 |
|  |  | 20026 | 3.47 | 0.48 | 2.92 | 0.05 | 0.02 |
|  |  | 20027 | 3.22 | 0.30 | 2.88 | 0.02 | 0.02 |
|  |  | 20028 | 3.29 | 0.25 | 3.01 | 0.02 | 0.01 |
|  |  | 20029 | 3.75 | 0.26 | 3.47 | 0.00 | 0.02 |
|  |  | 20030 | 6.26 | 0.74 | 5.44 | 0.05 | 0.03 |
|  | 0.5 | 21061 | 4.77 | 0.53 | 4.21 | 0.01 | 0.02 |
|  |  | 21062 | 2.29 | 0.24 | 1.99 | 0.01 | 0.05 |
|  |  | 21063 | 2.35 | 0.20 | 2.05 | 0.03 | 0.07 |
|  |  | 21064 | 4.69 | 0.51 | 4.14 | 0.01 | 0.03 |
|  |  | 21065 | 2.12 | 0.21 | 1.87 | 0.01 | 0.03 |
|  |  | 21066 | 6.48 | 0.68 | 5.67 | 0.09 | 0.04 |
|  |  | 21067 | 3.63 | 0.46 | 3.09 | 0.05 | 0.03 |
|  |  | 21068 | 4.57 | 0.44 | 4.06 | 0.05 | 0.02 |
|  |  | 21069 | 2.41 | 0.30 | 2.07 | 0.02 | 0.02 |
|  |  | 21070 | 4.35 | 0.43 | 3.82 | 0.06 | 0.04 |
|  | 1.5 | 22101 | 2.69 | 0.28 | 2.33 | 0.03 | 0.05 |
|  |  | 22102 | 2.11 | 0.23 | 1.85 | 0.00 | 0.03 |
|  |  | 22103 | 3.02 | 0.46 | 2.42 | 0.07 | 0.07 |
|  |  | 22104 | 2.55 | 0.43 | 2.08 | 0.03 | 0.01 |
|  |  | 22105 | 3.49 | 0.74 | 2.66 | 0.03 | 0.06 |
|  |  | 22106 | 4.48 | 0.48 | 3.91 | 0.03 | 0.06 |
|  |  | 22107 | 3.50 | 0.51 | 2.93 | 0.03 | 0.03 |
|  |  | 22108 | 2.32 | 0.27 | 2.01 | 0.02 | 0.02 |
|  |  | 22109 | 3.44 | 0.51 | 2.84 | 0.07 | 0.02 |
|  |  | 22110 | 2.64 | 0.46 | 2.10 | 0.04 | 0.04 |
|  | 5 | 23141 | 2.34 | 0.21 | 2.06 | 0.03 | 0.04 |
|  |  | 23142 | 1.51 | 0.26 | 1.23 | 0.00 | 0.02 |
|  |  | 23143 | 2.83 | 0.30 | 2.45 | 0.04 | 0.04 |
|  |  | 23144 | 3.16 | 0.39 | 2.72 | 0.03 | 0.02 |
|  |  | 23145 | 3.27 | 0.59 | 2.64 | 0.03 | 0.01 |
|  |  | 23146 | 4.77 | 0.86 | 3.81 | 0.03 | 0.07 |
|  |  | 23147 | 2.13 | 0.34 | 1.76 | 0.01 | 0.02 |
|  |  | 23148 | 4.11 | 0.78 | 3.18 | 0.05 | 0.10 |
|  |  | 23149 | 3.70 | 0.26 | 3.37 | 0.02 | 0.05 |
|  |  | 23150 | 4.60 | 0.61 | 3.91 | 0.04 | 0.04 |

### Hematology data of individual animal

| **Hematology data on day 92** | | | | | | | |
| --- | --- | --- | --- | --- | --- | --- | --- |
| **Gender** | **Dose** | **Animal no.** | **BASO#** | **NEUT%** | **LYMPH%** | **MONO%** | **EO%** |
|  | **g/kg** |  | **10^9^/L** | **%** | **%** | **%** | **%** |
| Female | 0 | 20021 | 0.00 | 17.5 | 81.2 | 1.0 | 0.3 |
|  |  | 20022 | 0.00 | 35.4 | 62.2 | 1.9 | 0.5 |
|  |  | 20023 | 0.00 | 12.7 | 86.1 | 0.7 | 0.5 |
|  |  | 20024 | 0.00 | 12.6 | 85.8 | 1.1 | 0.5 |
|  |  | 20025 | 0.00 | 21.9 | 76.5 | 0.9 | 0.7 |
|  |  | 20026 | 0.00 | 13.9 | 84.1 | 1.4 | 0.6 |
|  |  | 20027 | 0.00 | 9.4 | 89.4 | 0.6 | 0.6 |
|  |  | 20028 | 0.00 | 7.6 | 91.5 | 0.6 | 0.3 |
|  |  | 20029 | 0.00 | 7.0 | 92.5 | 0.0 | 0.5 |
|  |  | 20030 | 0.00 | 11.8 | 86.9 | 0.8 | 0.5 |
|  | 0.5 | 21061 | 0.00 | 11.1 | 88.3 | 0.2 | 0.4 |
|  |  | 21062 | 0.00 | 10.5 | 86.9 | 0.4 | 2.2 |
|  |  | 21063 | 0.00 | 8.5 | 87.2 | 1.3 | 3.0 |
|  |  | 21064 | 0.00 | 10.9 | 88.3 | 0.2 | 0.6 |
|  |  | 21065 | 0.00 | 9.9 | 88.2 | 0.5 | 1.4 |
|  |  | 21066 | 0.00 | 10.5 | 87.5 | 1.4 | 0.6 |
|  |  | 21067 | 0.00 | 12.7 | 85.1 | 1.4 | 0.8 |
|  |  | 21068 | 0.00 | 9.7 | 88.8 | 1.1 | 0.4 |
|  |  | 21069 | 0.00 | 12.5 | 85.9 | 0.8 | 0.8 |
|  |  | 21070 | 0.00 | 9.9 | 87.8 | 1.4 | 0.9 |
|  | 1.5 | 22101 | 0.00 | 10.4 | 86.6 | 1.1 | 1.9 |
|  |  | 22102 | 0.00 | 10.9 | 87.7 | 0.0 | 1.4 |
|  |  | 22103 | 0.00 | 15.3 | 80.1 | 2.3 | 2.3 |
|  |  | 22104 | 0.00 | 16.8 | 81.6 | 1.2 | 0.4 |
|  |  | 22105 | 0.00 | 21.2 | 76.2 | 0.9 | 1.7 |
|  |  | 22106 | 0.00 | 10.7 | 87.3 | 0.7 | 1.3 |
|  |  | 22107 | 0.00 | 14.5 | 83.7 | 0.9 | 0.9 |
|  |  | 22108 | 0.00 | 11.6 | 86.6 | 0.9 | 0.9 |
|  |  | 22109 | 0.00 | 14.8 | 82.6 | 2.0 | 0.6 |
|  |  | 22110 | 0.00 | 17.5 | 79.5 | 1.5 | 1.5 |
|  | 5 | 23141 | 0.00 | 9.0 | 88.0 | 1.3 | 1.7 |
|  |  | 23142 | 0.00 | 17.2 | 81.5 | 0.0 | 1.3 |
|  |  | 23143 | 0.00 | 10.6 | 86.6 | 1.4 | 1.4 |
|  |  | 23144 | 0.00 | 12.4 | 86.1 | 0.9 | 0.6 |
|  |  | 23145 | 0.00 | 18.1 | 80.7 | 0.9 | 0.3 |
|  |  | 23146 | 0.00 | 18.0 | 79.9 | 0.6 | 1.5 |
|  |  | 23147 | 0.00 | 16.0 | 82.6 | 0.5 | 0.9 |
|  |  | 23148 | 0.00 | 19.0 | 77.4 | 1.2 | 2.4 |
|  |  | 23149 | 0.00 | 7.0 | 91.1 | 0.5 | 1.4 |
|  |  | 23150 | 0.00 | 13.2 | 85.0 | 0.9 | 0.9 |

### Hematology data of individual animal

| **Hematology data on day 92** | | | | | | | |
| --- | --- | --- | --- | --- | --- | --- | --- |
| **Gender** | **Dose** | **Animal no.** | **BASO%** | **RBC** | **HGB** | **HCT** | **MCV** |
|  | **g/kg** |  | **%** | **10^12^/L** | **g/L** | **%** | **fL** |
| Female | 0 | 20021 | 0.0 | 7.50 | 136 | 37.7 | 50.3 |
|  |  | 20022 | 0.0 | 7.82 | 141 | 39.2 | 50.1 |
|  |  | 20023 | 0.0 | 7.66 | 153 | 42.7 | 55.7 |
|  |  | 20024 | 0.0 | 7.89 | 151 | 42.7 | 54.1 |
|  |  | 20025 | 0.0 | 8.32 | 152 | 41.8 | 50.2 |
|  |  | 20026 | 0.0 | 8.23 | 148 | 42.2 | 51.3 |
|  |  | 20027 | 0.0 | 8.21 | 154 | 42.6 | 51.9 |
|  |  | 20028 | 0.0 | 8.01 | 148 | 41.1 | 51.3 |
|  |  | 20029 | 0.0 | 7.85 | 152 | 42.3 | 53.9 |
|  |  | 20030 | 0.0 | 8.44 | 157 | 44.0 | 52.1 |
|  | 0.5 | 21061 | 0.0 | 8.27 | 154 | 42.9 | 51.9 |
|  |  | 21062 | 0.0 | 8.01 | 150 | 41.2 | 51.4 |
|  |  | 21063 | 0.0 | 8.24 | 153 | 42.5 | 51.6 |
|  |  | 21064 | 0.0 | 7.57 | 141 | 38.6 | 51.0 |
|  |  | 21065 | 0.0 | 7.01 | 131 | 37.3 | 53.2 |
|  |  | 21066 | 0.0 | 7.90 | 151 | 42.1 | 53.3 |
|  |  | 21067 | 0.0 | 7.51 | 145 | 41.2 | 54.9 |
|  |  | 21068 | 0.0 | 7.63 | 141 | 39.7 | 52.0 |
|  |  | 21069 | 0.0 | 7.98 | 145 | 40.8 | 51.1 |
|  |  | 21070 | 0.0 | 7.39 | 149 | 41.2 | 55.8 |
|  | 1.5 | 22101 | 0.0 | 7.97 | 149 | 41.2 | 51.7 |
|  |  | 22102 | 0.0 | 8.29 | 153 | 42.6 | 51.4 |
|  |  | 22103 | 0.0 | 8.55 | 158 | 42.8 | 50.1 |
|  |  | 22104 | 0.0 | 8.27 | 150 | 41.8 | 50.5 |
|  |  | 22105 | 0.0 | 7.97 | 153 | 42.5 | 53.3 |
|  |  | 22106 | 0.0 | 7.25 | 147 | 39.6 | 54.6 |
|  |  | 22107 | 0.0 | 7.52 | 147 | 40.9 | 54.4 |
|  |  | 22108 | 0.0 | 7.67 | 145 | 40.4 | 52.7 |
|  |  | 22109 | 0.0 | 7.95 | 149 | 41.6 | 52.3 |
|  |  | 22110 | 0.0 | 7.71 | 149 | 43.0 | 55.8 |
|  | 5 | 23141 | 0.0 | 7.97 | 153 | 42.7 | 53.6 |
|  |  | 23142 | 0.0 | 8.24 | 157 | 43.9 | 53.3 |
|  |  | 23143 | 0.0 | 7.54 | 151 | 42.8 | 56.8 |
|  |  | 23144 | 0.0 | 8.26 | 155 | 43.2 | 52.3 |
|  |  | 23145 | 0.0 | 7.45 | 148 | 43.0 | 57.7 |
|  |  | 23146 | 0.0 | 7.40 | 144 | 39.9 | 53.9 |
|  |  | 23147 | 0.0 | 7.98 | 151 | 42.0 | 52.6 |
|  |  | 23148 | 0.0 | 8.53 | 162 | 44.6 | 52.3 |
|  |  | 23149 | 0.0 | 8.02 | 151 | 41.9 | 52.2 |
|  |  | 23150 | 0.0 | 7.71 | 146 | 40.6 | 52.7 |

### Hematology data of individual animal

| **Hematology data on day 92** | | | | | | | |
| --- | --- | --- | --- | --- | --- | --- | --- |
| **Gender** | **Dose** | **Animal no.** | **MCH** | **MCHC** | **PLT** | **RET%** | **RET#** |
|  | **g/kg** |  | **pg** | **g/L** | **10^9^/L** | **%** | **10^9^/L** |
| Female | 0 | 20021 | 18.1 | 361 | 1012 | 2.78 | 208.5 |
|  |  | 20022 | 18.0 | 360 | 1107 | 2.94 | 229.9 |
|  |  | 20023 | 20.0 | 358 | 990 | 3.51 | 268.9 |
|  |  | 20024 | 19.1 | 354 | 1305 | 2.58 | 203.6 |
|  |  | 20025 | 18.3 | 364 | 1146 | 2.44 | 203.0 |
|  |  | 20026 | 18.0 | 351 | 1167 | 3.11 | 256.0 |
|  |  | 20027 | 18.8 | 362 | 1202 | 3.02 | 247.9 |
|  |  | 20028 | 18.5 | 360 | 780 | 2.03 | 162.6 |
|  |  | 20029 | 19.4 | 359 | 1027 | 2.43 | 190.8 |
|  |  | 20030 | 18.6 | 357 | 1133 | 2.90 | 244.8 |
|  | 0.5 | 21061 | 18.6 | 359 | 1240 | 1.91 | 158.0 |
|  |  | 21062 | 18.7 | 364 | 1518 | 2.45 | 196.2 |
|  |  | 21063 | 18.6 | 360 | 995 | 1.79 | 147.5 |
|  |  | 21064 | 18.6 | 365 | 961 | 2.74 | 207.4 |
|  |  | 21065 | 18.7 | 351 | 1042 | 2.70 | 189.3 |
|  |  | 21066 | 19.1 | 359 | 940 | 2.85 | 225.2 |
|  |  | 21067 | 19.3 | 352 | 1226 | 2.66 | 199.8 |
|  |  | 21068 | 18.5 | 355 | 1179 | 2.78 | 212.1 |
|  |  | 21069 | 18.2 | 355 | 1033 | 2.63 | 209.9 |
|  |  | 21070 | 20.2 | 362 | 989 | 2.33 | 172.2 |
|  | 1.5 | 22101 | 18.7 | 362 | 1140 | 3.05 | 243.1 |
|  |  | 22102 | 18.5 | 359 | 1294 | 2.64 | 218.9 |
|  |  | 22103 | 18.5 | 369 | 1212 | 1.98 | 169.3 |
|  |  | 22104 | 18.1 | 359 | 1257 | 2.57 | 212.5 |
|  |  | 22105 | 19.2 | 360 | 1109 | 2.74 | 218.4 |
|  |  | 22106 | 20.3 | 371 | 1037 | 3.31 | 240.0 |
|  |  | 22107 | 19.5 | 359 | 1042 | 2.74 | 206.0 |
|  |  | 22108 | 18.9 | 359 | 1056 | 2.99 | 229.3 |
|  |  | 22109 | 18.7 | 358 | 1197 | 2.31 | 183.6 |
|  |  | 22110 | 19.3 | 347 | 1088 | 3.77 | 290.7 |
|  | 5 | 23141 | 19.2 | 358 | 983 | 1.97 | 157.0 |
|  |  | 23142 | 19.1 | 358 | 1168 | 3.26 | 268.6 |
|  |  | 23143 | 20.0 | 353 | 1111 | 3.86 | 291.0 |
|  |  | 23144 | 18.8 | 359 | 1128 | 2.59 | 213.9 |
|  |  | 23145 | 19.9 | 344 | 991 | 3.54 | 263.7 |
|  |  | 23146 | 19.5 | 361 | 1049 | 3.19 | 236.1 |
|  |  | 23147 | 18.9 | 360 | 1093 | 3.10 | 247.4 |
|  |  | 23148 | 19.0 | 363 | 1247 | 2.10 | 179.1 |
|  |  | 23149 | 18.8 | 360 | 1117 | 2.24 | 179.6 |
|  |  | 23150 | 18.9 | 360 | 998 | 3.94 | 303.8 |

### Hematology data of individual animal

| **Hematology data on recovery day 29** | | | | | | | |
| --- | --- | --- | --- | --- | --- | --- | --- |
| **Gender** | **Dose** | **Animal no.** | **WBC** | **NEUT#** | **LYMPH#** | **MONO#** | **EO#** |
|  | **g/kg** |  | **10^9^/L** | **10^9^/L** | **10^9^/L** | **10^9^/L** | **10^9^/L** |
| Male | 0 | 10011 | 6.19 | 0.85 | 5.18 | 0.03 | 0.12 |
|  |  | 10012 | 3.87 | 1.39 | 2.39 | 0.03 | 0.06 |
|  |  | 10013 | 4.49 | 0.72 | 3.65 | 0.03 | 0.09 |
|  |  | 10014 | 12.63 | 3.14 | 9.11 | 0.22 | 0.16 |
|  |  | 10015 | 7.31 | 1.10 | 6.00 | 0.06 | 0.15 |
|  | 0.5 | 11051 | 4.37 | 0.71 | 3.52 | 0.08 | 0.06 |
|  |  | 11052 | 5.87 | 0.78 | 4.94 | 0.06 | 0.09 |
|  |  | 11053 | 6.35 | 1.35 | 4.84 | 0.06 | 0.10 |
|  |  | 11054 | 7.70 | 1.70 | 5.85 | 0.07 | 0.08 |
|  |  | 11055 | 5.39 | 1.43 | 3.82 | 0.05 | 0.09 |
|  | 1.5 | 12091 | 4.32 | 0.87 | 3.33 | 0.03 | 0.09 |
|  |  | 12092 | 4.69 | 1.07 | 3.38 | 0.09 | 0.15 |
|  |  | 12093 | 5.43 | 1.26 | 4.06 | 0.05 | 0.06 |
|  |  | 12094 | 6.12 | 1.03 | 4.99 | 0.02 | 0.08 |
|  |  | 12095 | 6.34 | 1.31 | 4.85 | 0.08 | 0.10 |
|  | 5 | 13131 | 3.30 | 0.69 | 2.47 | 0.03 | 0.11 |
|  |  | 13132 | 5.27 | 1.16 | 3.91 | 0.07 | 0.13 |
|  |  | 13133 | 4.76 | 1.22 | 3.39 | 0.06 | 0.09 |
|  |  | 13134 | 6.16 | 2.22 | 3.83 | 0.05 | 0.06 |
|  |  | 13135 | 7.18 | 1.31 | 5.75 | 0.04 | 0.08 |
| Female | 0 | 20031 | 4.63 | 0.90 | 3.67 | 0.03 | 0.03 |
|  |  | 20032 | 5.46 | 0.62 | 4.81 | 0.02 | 0.01 |
|  |  | 20033 | 7.21 | 0.32 | 6.84 | 0.00 | 0.05 |
|  |  | 20034 | 2.53 | 0.24 | 2.26 | 0.00 | 0.03 |
|  |  | 20035 | 2.27 | 0.32 | 1.88 | 0.03 | 0.04 |
|  | 0.5 | 21071 | 4.67 | 1.04 | 3.54 | 0.01 | 0.08 |
|  |  | 21072 | 3.97 | 1.01 | 2.80 | 0.01 | 0.15 |
|  |  | 21073 | 4.72 | 0.71 | 3.98 | 0.01 | 0.02 |
|  |  | 21074 | 3.84 | 0.92 | 2.74 | 0.09 | 0.09 |
|  |  | 21075 | 4.32 | 0.53 | 3.71 | 0.04 | 0.04 |
|  | 1.5 | 22111 | 2.22 | 0.37 | 1.78 | 0.03 | 0.04 |
|  |  | 22112 | 2.61 | 0.44 | 2.11 | 0.02 | 0.04 |
|  |  | 22113 | 3.20 | 0.52 | 2.59 | 0.05 | 0.04 |
|  |  | 22114 | 1.43 | 0.25 | 1.15 | 0.00 | 0.03 |
|  |  | 22115 | 2.22 | 0.43 | 1.72 | 0.02 | 0.05 |
|  | 5 | 23151 | 2.36 | 0.33 | 2.00 | 0.00 | 0.03 |
|  |  | 23152 | 2.75 | 0.27 | 2.45 | 0.01 | 0.02 |
|  |  | 23153 | 4.05 | 1.37 | 2.57 | 0.07 | 0.04 |
|  |  | 23154 | 4.21 | 0.84 | 3.30 | 0.03 | 0.04 |
|  |  | 23155 | 2.55 | 0.51 | 1.96 | 0.04 | 0.04 |

### Hematology data of individual animal

| **Hematology data on recovery day 29** | | | | | | | |
| --- | --- | --- | --- | --- | --- | --- | --- |
| **Gender** | **Dose** | **Animal no.** | **BASO#** | **NEUT%** | **LYMPH%** | **MONO%** | **EO%** |
|  | **g/kg** |  | **10^9^/L** | **%** | **%** | **%** | **%** |
| Male | 0 | 10011 | 0.01 | 13.7 | 83.7 | 0.5 | 1.9 |
|  |  | 10012 | 0.00 | 35.8 | 61.8 | 0.8 | 1.6 |
|  |  | 10013 | 0.00 | 16.0 | 81.3 | 0.7 | 2.0 |
|  |  | 10014 | 0.00 | 24.9 | 72.1 | 1.7 | 1.3 |
|  |  | 10015 | 0.00 | 15.0 | 82.1 | 0.8 | 2.1 |
|  | 0.5 | 11051 | 0.00 | 16.3 | 80.5 | 1.8 | 1.4 |
|  |  | 11052 | 0.00 | 13.3 | 84.2 | 1.0 | 1.5 |
|  |  | 11053 | 0.00 | 21.3 | 76.2 | 0.9 | 1.6 |
|  |  | 11054 | 0.00 | 22.1 | 76.0 | 0.9 | 1.0 |
|  |  | 11055 | 0.00 | 26.5 | 70.9 | 0.9 | 1.7 |
|  | 1.5 | 12091 | 0.00 | 20.1 | 77.1 | 0.7 | 2.1 |
|  |  | 12092 | 0.00 | 22.8 | 72.1 | 1.9 | 3.2 |
|  |  | 12093 | 0.00 | 23.2 | 74.8 | 0.9 | 1.1 |
|  |  | 12094 | 0.00 | 16.9 | 81.5 | 0.3 | 1.3 |
|  |  | 12095 | 0.00 | 20.6 | 76.5 | 1.3 | 1.6 |
|  | 5 | 13131 | 0.00 | 21.0 | 74.8 | 0.9 | 3.3 |
|  |  | 13132 | 0.00 | 22.0 | 74.2 | 1.3 | 2.5 |
|  |  | 13133 | 0.00 | 25.6 | 71.2 | 1.3 | 1.9 |
|  |  | 13134 | 0.00 | 36.0 | 62.2 | 0.8 | 1.0 |
|  |  | 13135 | 0.00 | 18.2 | 80.1 | 0.6 | 1.1 |
| Female | 0 | 20031 | 0.00 | 19.5 | 79.3 | 0.6 | 0.6 |
|  |  | 20032 | 0.00 | 11.3 | 88.1 | 0.4 | 0.2 |
|  |  | 20033 | 0.00 | 4.4 | 94.9 | 0.0 | 0.7 |
|  |  | 20034 | 0.00 | 9.5 | 89.3 | 0.0 | 1.2 |
|  |  | 20035 | 0.00 | 14.1 | 82.8 | 1.3 | 1.8 |
|  | 0.5 | 21071 | 0.00 | 22.3 | 75.8 | 0.2 | 1.7 |
|  |  | 21072 | 0.00 | 25.4 | 70.5 | 0.3 | 3.8 |
|  |  | 21073 | 0.00 | 15.1 | 84.3 | 0.2 | 0.4 |
|  |  | 21074 | 0.00 | 24.0 | 71.4 | 2.3 | 2.3 |
|  |  | 21075 | 0.00 | 12.3 | 85.9 | 0.9 | 0.9 |
|  | 1.5 | 22111 | 0.00 | 16.6 | 80.2 | 1.4 | 1.8 |
|  |  | 22112 | 0.00 | 16.9 | 80.8 | 0.8 | 1.5 |
|  |  | 22113 | 0.00 | 16.2 | 80.9 | 1.6 | 1.3 |
|  |  | 22114 | 0.00 | 17.5 | 80.4 | 0.0 | 2.1 |
|  |  | 22115 | 0.00 | 19.3 | 77.5 | 0.9 | 2.3 |
|  | 5 | 23151 | 0.00 | 14.0 | 84.7 | 0.0 | 1.3 |
|  |  | 23152 | 0.00 | 9.8 | 89.1 | 0.4 | 0.7 |
|  |  | 23153 | 0.00 | 33.8 | 63.5 | 1.7 | 1.0 |
|  |  | 23154 | 0.00 | 19.9 | 78.4 | 0.7 | 1.0 |
|  |  | 23155 | 0.00 | 19.9 | 76.9 | 1.6 | 1.6 |

### Hematology data of individual animal

| **Hematology data on recovery day 29** | | | | | | | |
| --- | --- | --- | --- | --- | --- | --- | --- |
| **Gender** | **Dose** | **Animal no.** | **BASO%** | **RBC** | **HGB** | **HCT** | **MCV** |
|  | **g/kg** |  | **%** | **10^12^/L** | **g/L** | **%** | **fL** |
| Male | 0 | 10011 | 0.2 | 9.05 | 165 | 45.8 | 50.6 |
|  |  | 10012 | 0.0 | 8.46 | 140 | 39.9 | 47.2 |
|  |  | 10013 | 0.0 | 8.46 | 149 | 41.0 | 48.5 |
|  |  | 10014 | 0.0 | 8.71 | 150 | 42.5 | 48.8 |
|  |  | 10015 | 0.0 | 8.98 | 161 | 45.0 | 50.1 |
|  | 0.5 | 11051 | 0.0 | 9.15 | 159 | 43.3 | 47.3 |
|  |  | 11052 | 0.0 | 8.40 | 162 | 44.4 | 52.9 |
|  |  | 11053 | 0.0 | 8.99 | 160 | 45.2 | 50.3 |
|  |  | 11054 | 0.0 | 9.66 | 159 | 44.4 | 46.0 |
|  |  | 11055 | 0.0 | 9.00 | 159 | 45.6 | 50.7 |
|  | 1.5 | 12091 | 0.0 | 8.56 | 151 | 42.1 | 49.2 |
|  |  | 12092 | 0.0 | 9.29 | 162 | 44.2 | 47.6 |
|  |  | 12093 | 0.0 | 8.57 | 149 | 41.4 | 48.3 |
|  |  | 12094 | 0.0 | 8.22 | 150 | 42.3 | 51.5 |
|  |  | 12095 | 0.0 | 9.28 | 164 | 45.9 | 49.5 |
|  | 5 | 13131 | 0.0 | 8.36 | 157 | 43.5 | 52.0 |
|  |  | 13132 | 0.0 | 8.70 | 164 | 46.1 | 53.0 |
|  |  | 13133 | 0.0 | 8.68 | 156 | 42.9 | 49.4 |
|  |  | 13134 | 0.0 | 8.61 | 158 | 44.0 | 51.1 |
|  |  | 13135 | 0.0 | 8.47 | 156 | 44.1 | 52.1 |
| Female | 0 | 20031 | 0.0 | 8.23 | 142 | 40.3 | 49.0 |
|  |  | 20032 | 0.0 | 7.93 | 149 | 41.9 | 52.8 |
|  |  | 20033 | 0.0 | 8.02 | 144 | 41.2 | 51.4 |
|  |  | 20034 | 0.0 | 7.86 | 144 | 41.0 | 52.2 |
|  |  | 20035 | 0.0 | 7.59 | 139 | 39.4 | 51.9 |
|  | 0.5 | 21071 | 0.0 | 8.17 | 149 | 42.4 | 51.9 |
|  |  | 21072 | 0.0 | 7.99 | 152 | 43.4 | 54.3 |
|  |  | 21073 | 0.0 | 8.01 | 148 | 41.7 | 52.1 |
|  |  | 21074 | 0.0 | 8.25 | 156 | 43.9 | 53.2 |
|  |  | 21075 | 0.0 | 7.86 | 143 | 40.5 | 51.5 |
|  | 1.5 | 22111 | 0.0 | 8.16 | 148 | 41.8 | 51.2 |
|  |  | 22112 | 0.0 | 8.13 | 148 | 41.5 | 51.0 |
|  |  | 22113 | 0.0 | 7.46 | 142 | 40.1 | 53.8 |
|  |  | 22114 | 0.0 | 7.75 | 153 | 43.2 | 55.7 |
|  |  | 22115 | 0.0 | 8.00 | 150 | 42.3 | 52.9 |
|  | 5 | 23151 | 0.0 | 7.54 | 148 | 41.9 | 55.6 |
|  |  | 23152 | 0.0 | 7.90 | 151 | 43.0 | 54.4 |
|  |  | 23153 | 0.0 | 7.83 | 146 | 41.0 | 52.4 |
|  |  | 23154 | 0.0 | 7.85 | 149 | 42.4 | 54.0 |
|  |  | 23155 | 0.0 | 8.16 | 150 | 41.5 | 50.9 |

### Hematology data of individual animal

| **Hematology data on recovery day 29** | | | | | | | |
| --- | --- | --- | --- | --- | --- | --- | --- |
| **Gender** | **Dose** | **Animal no.** | **MCH** | **MCHC** | **PLT** | **RET%** | **RET#** |
|  | **g/kg** |  | **pg** | **g/L** | **10^9^/L** | **%** | **10^9^/L** |
| Male | 0 | 10011 | 18.2 | 360 | 1089 | 2.64 | 238.9 |
|  |  | 10012 | 16.5 | 351 | 1273 | 4.07 | 344.3 |
|  |  | 10013 | 17.6 | 363 | 1256 | 2.50 | 211.5 |
|  |  | 10014 | 17.2 | 353 | 1300 | 2.91 | 253.5 |
|  |  | 10015 | 17.9 | 358 | 1384 | 2.52 | 226.3 |
|  | 0.5 | 11051 | 17.4 | 367 | 1196 | 2.46 | 225.1 |
|  |  | 11052 | 19.3 | 365 | 1041 | 2.91 | 244.4 |
|  |  | 11053 | 17.8 | 354 | 1036 | 2.79 | 250.8 |
|  |  | 11054 | 16.5 | 358 | 1467 | 3.44 | 332.3 |
|  |  | 11055 | 17.7 | 349 | 1421 | 3.71 | 333.9 |
|  | 1.5 | 12091 | 17.6 | 359 | 1038 | 2.64 | 226.0 |
|  |  | 12092 | 17.4 | 367 | 1079 | 2.24 | 208.1 |
|  |  | 12093 | 17.4 | 360 | 1089 | 2.67 | 228.8 |
|  |  | 12094 | 18.2 | 355 | 1201 | 2.88 | 236.7 |
|  |  | 12095 | 17.7 | 357 | 1224 | 2.32 | 215.3 |
|  | 5 | 13131 | 18.8 | 361 | 1271 | 3.28 | 274.2 |
|  |  | 13132 | 18.9 | 356 | 1132 | 2.72 | 236.6 |
|  |  | 13133 | 18.0 | 364 | 1174 | 3.15 | 273.4 |
|  |  | 13134 | 18.4 | 359 | 1259 | 3.31 | 285.0 |
|  |  | 13135 | 18.4 | 354 | 1255 | 2.54 | 215.1 |
| Female | 0 | 20031 | 17.3 | 352 | 1208 | 2.38 | 195.9 |
|  |  | 20032 | 18.8 | 356 | 1160 | 2.40 | 190.3 |
|  |  | 20033 | 18.0 | 350 | 1362 | 2.22 | 178.0 |
|  |  | 20034 | 18.3 | 351 | 1086 | 2.17 | 170.6 |
|  |  | 20035 | 18.3 | 353 | 1113 | 2.70 | 204.9 |
|  | 0.5 | 21071 | 18.2 | 351 | 1133 | 2.59 | 211.6 |
|  |  | 21072 | 19.0 | 350 | 1024 | 2.66 | 212.5 |
|  |  | 21073 | 18.5 | 355 | 1289 | 2.60 | 208.3 |
|  |  | 21074 | 18.9 | 355 | 1214 | 1.87 | 154.3 |
|  |  | 21075 | 18.2 | 353 | 1548 | 2.16 | 169.8 |
|  | 1.5 | 22111 | 18.1 | 354 | 1092 | 2.12 | 173.0 |
|  |  | 22112 | 18.2 | 357 | 1158 | 2.30 | 187.0 |
|  |  | 22113 | 19.0 | 354 | 1005 | 2.62 | 195.5 |
|  |  | 22114 | 19.7 | 354 | 1106 | 3.09 | 239.5 |
|  |  | 22115 | 18.8 | 355 | 1217 | 2.43 | 194.4 |
|  | 5 | 23151 | 19.6 | 353 | 1003 | 2.03 | 153.1 |
|  |  | 23152 | 19.1 | 351 | 1102 | 3.53 | 278.9 |
|  |  | 23153 | 18.6 | 356 | 916 | 2.16 | 169.1 |
|  |  | 23154 | 19.0 | 351 | 972 | 1.83 | 143.7 |
|  |  | 23155 | 18.4 | 361 | 1129 | 1.80 | 146.9 |

### Coagulation data of individual animal

| **Coagulation data on day 92** | | | | | |
| --- | --- | --- | --- | --- | --- |
| **Gender** | **Dose** | **Animal no.** | **PT** | **APTT** | **FIB** |
|  | **g/kg** |  | **Sec.** | **Sec.** | **g/L** |
| Male | 0 | 10001 | 15.3 | 20.1 | 3.21 |
|  |  | 10002 | 16.7 | 18.1 | 3.40 |
|  |  | 10003 | 16.5 | 17.8 | 2.65 |
|  |  | 10004 | 15.5 | 19.2 | 3.17 |
|  |  | 10005 | 16.6 | 25.5 | 3.13 |
|  |  | 10006 | 15.8 | 18.8 | 3.57 |
|  |  | 10007 | 16.4 | 18.3 | 3.47 |
|  |  | 10008 | 16.4 | 20.0 | 3.42 |
|  |  | 10009 | 15.7 | 19.0 | 3.45 |
|  |  | 10010 | 16.6 | 25.5 | 2.99 |
|  | 0.5 | 11041 | 16.7 | 19.4 | 3.21 |
|  |  | 11042 | 16.3 | 18.4 | 3.23 |
|  |  | 11043 | 15.7 | 20.5 | 3.04 |
|  |  | 11044 | 16.4 | 20.0 | 2.61 |
|  |  | 11045 | 16.6 | 21.3 | 3.08 |
|  |  | 11046 | 16.0 | 18.5 | 3.04 |
|  |  | 11047 | 16.3 | 18.6 | 2.78 |
|  |  | 11048 | 16.7 | 18.6 | 3.21 |
|  |  | 11049 | 15.1 | 20.4 | 3.19 |
|  |  | 11050 | 15.6 | 18.0 | 4.11 |
|  | 1.5 | 12081 | 17.1 | 20.6 | 2.78 |
|  |  | 12082 | 15.5 | 20.8 | 2.95 |
|  |  | 12083 | 17.4 | 19.4 | 2.95 |
|  |  | 12084 | 15.7 | 18.5 | 2.95 |
|  |  | 12085 | 16.3 | 22.2 | 2.66 |
|  |  | 12086 | 16.5 | 21.6 | 2.89 |
|  |  | 12087 | 16.3 | 18.9 | 3.02 |
|  |  | 12088 | 16.5 | 20.4 | 3.09 |
|  |  | 12089 | 16.7 | 20.6 | 2.99 |
|  |  | 12090 | 16.0 | 18.6 | 3.21 |
|  | 5 | 13121 | 16.9 | 21.0 | 2.97 |
|  |  | 13122 | 15.4 | 18.0 | 2.90 |
|  |  | 13123 | 16.3 | 17.5 | 3.23 |
|  |  | 13124 | 18.2 | 19.6 | 2.79 |
|  |  | 13125 | 16.9 | 21.3 | 3.00 |
|  |  | 13126 | 17.0 | 18.0 | 3.29 |
|  |  | 13127 | 17.1 | 19.8 | 2.56 |
|  |  | 13128 | 16.3 | 17.1 | 2.89 |
|  |  | 13129 | 16.9 | 16.8 | 2.99 |
|  |  | 13130 | 16.7 | 19.6 | 2.89 |

### Coagulation data of individual animal

| **Coagulation data on day 92** | | | | | |
| --- | --- | --- | --- | --- | --- |
| **Gender** | **Dose** | **Animal no.** | **PT** | **APTT** | **FIB** |
|  | **g/kg** |  | **Sec.** | **Sec.** | **g/L** |
| Female | 0 | 20021 | 15.9 | 18.1 | 2.22 |
|  |  | 20022 | 16.6 | 20.3 | 2.20 |
|  |  | 20023 | 15.6 | 18.6 | 2.20 |
|  |  | 20024 | 15.9 | 17.8 | 1.99 |
|  |  | 20025 | 15.9 | 19.2 | 2.24 |
|  |  | 20026 | 14.9 | 16.1 | 2.26 |
|  |  | 20027 | 15.6 | 21.2 | 2.27 |
|  |  | 20028 | 15.4 | 19.6 | 2.16 |
|  |  | 20029 | 15.3 | 18.9 | 1.90 |
|  |  | 20030 | 16.1 | 18.5 | 2.44 |
|  | 0.5 | 21061 | 17.3 | 19.1 | 2.30 |
|  |  | 21062 | 16.7 | 15.4 | 1.99 |
|  |  | 21063 | 16.3 | 18.4 | 2.64 |
|  |  | 21064 | 15.3 | 21.9 | 2.17 |
|  |  | 21065 | 16.0 | 19.7 | 2.06 |
|  |  | 21066 | 15.5 | 17.2 | 2.31 |
|  |  | 21067 | 14.6 | 17.8 | 2.25 |
|  |  | 21068 | 16.2 | 18.3 | 2.34 |
|  |  | 21069 | 15.8 | 17.2 | 2.51 |
|  |  | 21070 | 17.0 | 19.6 | 2.01 |
|  | 1.5 | 22101 | 16.1 | 17.2 | 2.05 |
|  |  | 22102 | 16.5 | 19.7 | 2.31 |
|  |  | 22103 | 15.3 | 15.6 | 2.32 |
|  |  | 22104 | 16.5 | 18.1 | 2.36 |
|  |  | 22105 | 15.5 | 20.8 | 1.93 |
|  |  | 22106 | 15.1 | 17.4 | 2.02 |
|  |  | 22107 | 15.1 | 17.2 | 2.29 |
|  |  | 22108 | 15.3 | 16.4 | 2.48 |
|  |  | 22109 | 16.4 | 16.0 | 2.37 |
|  |  | 22110 | 15.9 | 15.9 | 1.98 |
|  | 5 | 23141 | 15.8 | 17.4 | 1.99 |
|  |  | 23142 | 15.2 | 18.6 | 2.36 |
|  |  | 23143 | 15.3 | 20.2 | 1.95 |
|  |  | 23144 | 16.2 | 17.0 | 2.44 |
|  |  | 23145 | 16.3 | 16.4 | 2.75 |
|  |  | 23146 | 15.3 | 18.5 | 2.10 |
|  |  | 23147 | 17.0 | 14.5 | 2.46 |
|  |  | 23148 | 16.3 | 18.9 | 2.24 |
|  |  | 23149 | 16.9 | 18.8 | 2.24 |
|  |  | 23150 | 16.9 | 19.9 | 2.44 |

### Blood biochemistry data of individual animal

| **Blood biochemistry data on day 92** | | | | | | | | |
| --- | --- | --- | --- | --- | --- | --- | --- | --- |
| **Gender** | **Dose** | **Animal no.** | **TBIL** | **TP** | **ALB** | **GLOB** | **A/G** | **ALT** |
|  | **g/kg** |  | **µmol/L** | **g/L** | **g/L** | **g/L** |  | **U/L** |
| Male | 0 | 10001 | 1.5 | 66.5 | 35.0 | 31.5 | 1.1 | 29 |
|  |  | 10002 | 1.7 | 71.2 | 37.9 | 33.3 | 1.1 | 42 |
|  |  | 10003 | 1.4 | 62.8 | 34.1 | 28.7 | 1.2 | 47 |
|  |  | 10004 | 1.6 | 73.2 | 39.1 | 34.1 | 1.1 | 33 |
|  |  | 10005 | 1.9 | 69.4 | 36.7 | 32.7 | 1.1 | 37 |
|  |  | 10006 | 1.4 | 74.1 | 39.2 | 34.9 | 1.1 | 36 |
|  |  | 10007 | 1.6 | 68.7 | 37.4 | 31.3 | 1.2 | 42 |
|  |  | 10008 | 1.2 | 64.9 | 34.6 | 30.3 | 1.1 | 43 |
|  |  | 10009 | 2.2 | 63.3 | 33.6 | 29.7 | 1.1 | 41 |
|  |  | 10010 | 2.0 | 69.2 | 37.2 | 32.0 | 1.2 | 43 |
|  | 0.5 | 11041 | 1.5 | 61.3 | 32.2 | 29.1 | 1.1 | 47 |
|  |  | 11042 | 2.0 | 71.8 | 38.5 | 33.3 | 1.2 | 36 |
|  |  | 11043 | 1.8 | 68.8 | 36.5 | 32.3 | 1.1 | 36 |
|  |  | 11044 | 1.4 | 62.3 | 33.8 | 28.5 | 1.2 | 37 |
|  |  | 11045 | 1.4 | 70.6 | 37.7 | 32.9 | 1.1 | 38 |
|  |  | 11046 | 1.5 | 60.3 | 32.8 | 27.5 | 1.2 | 34 |
|  |  | 11047 | 1.2 | 63.6 | 34.3 | 29.3 | 1.2 | 31 |
|  |  | 11048 | 1.1 | 63.4 | 35.9 | 27.5 | 1.3 | 28 |
|  |  | 11049 | 1.6 | 68.8 | 37.3 | 31.5 | 1.2 | 39 |
|  |  | 11050 | 2.1 | 72.4 | 37.8 | 34.6 | 1.1 | 34 |
|  | 1.5 | 12081 | 1.9 | 64.3 | 34.8 | 29.5 | 1.2 | 35 |
|  |  | 12082 | 1.5 | 68.3 | 35.9 | 32.4 | 1.1 | 28 |
|  |  | 12083 | 2.0 | 60.1 | 33.5 | 26.6 | 1.3 | 28 |
|  |  | 12084 | 1.5 | 64.0 | 34.4 | 29.6 | 1.2 | 36 |
|  |  | 12085 | 1.7 | 64.2 | 34.4 | 29.8 | 1.2 | 42 |
|  |  | 12086 | 1.7 | 63.3 | 34.0 | 29.3 | 1.2 | 45 |
|  |  | 12087 | 2.1 | 66.0 | 35.4 | 30.6 | 1.2 | 28 |
|  |  | 12088 | 1.6 | 63.6 | 34.7 | 28.9 | 1.2 | 29 |
|  |  | 12089 | 1.8 | 64.1 | 34.3 | 29.8 | 1.2 | 66 |
|  |  | 12090 | 1.8 | 68.5 | 36.4 | 32.1 | 1.1 | 23 |
|  | 5 | 13121 | 1.7 | 61.7 | 33.4 | 28.3 | 1.2 | 58 |
|  |  | 13122 | 1.9 | 66.3 | 35.1 | 31.2 | 1.1 | 26 |
|  |  | 13123 | 2.3 | 61.0 | 33.7 | 27.3 | 1.2 | 21 |
|  |  | 13124 | 1.8 | 64.5 | 34.5 | 30.0 | 1.1 | 33 |
|  |  | 13125 | 1.7 | 65.5 | 35.2 | 30.3 | 1.2 | 27 |
|  |  | 13126 | 1.7 | 65.2 | 35.8 | 29.4 | 1.2 | 26 |
|  |  | 13127 | 2.0 | 61.5 | 34.1 | 27.4 | 1.2 | 57 |
|  |  | 13128 | 2.1 | 66.2 | 36.3 | 29.9 | 1.2 | 36 |
|  |  | 13129 | 1.2 | 63.8 | 35.5 | 28.3 | 1.3 | 49 |
|  |  | 13130 | 1.5 | 61.0 | 33.2 | 27.8 | 1.2 | 38 |

### Blood biochemistry data of individual animal

| **Blood biochemistry data on day 92** | | | | | | | | |
| --- | --- | --- | --- | --- | --- | --- | --- | --- |
| **Gender** | **Dose** | **Animal no.** | **AST** | **ALP** | **GGT** | **CK** | **UREA** | **CREA** |
|  | **g/kg** |  | **U/L** | **U/L** | **U/L** | **U/L** | **mmol/L** | **µmol/L** |
| Male | 0 | 10001 | 102 | 69 | 1 | 397 | 5.65 | 25.2 |
|  |  | 10002 | 136 | 64 | 0 | 523 | 5.84 | 23.7 |
|  |  | 10003 | 157 | 77 | 0 | 536 | 5.41 | 28.3 |
|  |  | 10004 | 115 | 78 | 0 | 439 | 5.14 | 21.7 |
|  |  | 10005 | 94 | 76 | 2 | 350 | 5.60 | 25.8 |
|  |  | 10006 | 129 | 108 | 1 | 440 | 5.96 | 24.2 |
|  |  | 10007 | 115 | 82 | 0 | 302 | 6.55 | 24.0 |
|  |  | 10008 | 116 | 84 | 0 | 501 | 6.48 | 23.9 |
|  |  | 10009 | 118 | 102 | 1 | 482 | 8.39 | 28.2 |
|  |  | 10010 | 118 | 71 | 0 | 324 | 6.79 | 25.4 |
|  | 0.5 | 11041 | 167 | 75 | 1 | 465 | 6.05 | 29.1 |
|  |  | 11042 | 132 | 85 | 0 | 517 | 5.70 | 26.2 |
|  |  | 11043 | 114 | 96 | 0 | 361 | 5.34 | 29.2 |
|  |  | 11044 | 187 | 89 | 1 | 750 | 5.35 | 22.5 |
|  |  | 11045 | 100 | 60 | 0 | 318 | 6.20 | 22.9 |
|  |  | 11046 | 96 | 88 | 0 | 275 | 7.32 | 26.8 |
|  |  | 11047 | 125 | 76 | 1 | 375 | 6.30 | 25.0 |
|  |  | 11048 | 101 | 79 | 1 | 321 | 6.77 | 28.6 |
|  |  | 11049 | 162 | 60 | 1 | 596 | 6.89 | 23.1 |
|  |  | 11050 | 116 | 62 | 1 | 518 | 5.34 | 19.1 |
|  | 1.5 | 12081 | 170 | 83 | 0 | 764 | 5.62 | 27.4 |
|  |  | 12082 | 131 | 79 | 0 | 574 | 5.81 | 28.2 |
|  |  | 12083 | 128 | 81 | 0 | 592 | 7.07 | 29.4 |
|  |  | 12084 | 138 | 90 | 1 | 566 | 6.85 | 24.2 |
|  |  | 12085 | 141 | 98 | 1 | 447 | 6.90 | 28.9 |
|  |  | 12086 | 103 | 80 | 0 | 225 | 5.81 | 27.0 |
|  |  | 12087 | 78 | 82 | 1 | 178 | 5.43 | 24.4 |
|  |  | 12088 | 87 | 95 | 0 | 235 | 6.59 | 29.8 |
|  |  | 12089 | 179 | 98 | 2 | 536 | 7.68 | 28.1 |
|  |  | 12090 | 121 | 62 | 1 | 417 | 5.83 | 21.1 |
|  | 5 | 13121 | 147 | 134 | 0 | 455 | 5.56 | 27.7 |
|  |  | 13122 | 124 | 104 | 2 | 425 | 7.36 | 30.4 |
|  |  | 13123 | 114 | 63 | 0 | 451 | 5.22 | 30.6 |
|  |  | 13124 | 144 | 53 | 1 | 572 | 5.95 | 27.1 |
|  |  | 13125 | 110 | 119 | 2 | 514 | 5.66 | 27.0 |
|  |  | 13126 | 102 | 84 | 0 | 364 | 5.89 | 24.3 |
|  |  | 13127 | 162 | 88 | 1 | 411 | 5.15 | 21.6 |
|  |  | 13128 | 108 | 75 | 1 | 364 | 4.91 | 19.1 |
|  |  | 13129 | 141 | 56 | 1 | 392 | 5.62 | 24.8 |
|  |  | 13130 | 157 | 97 | 1 | 486 | 7.16 | 30.5 |

### Blood biochemistry data of individual animal

| **Blood biochemistry data on day 92** | | | | | | | | |
| --- | --- | --- | --- | --- | --- | --- | --- | --- |
| **Gender** | **Dose** | **Animal no.** | **GLU** | **TG** | **CHOL** | **K^+^** | **Na^+^** | **Cl^-^** |
|  | **g/kg** |  | **mmol/L** | **mmol/L** | **mmol/L** | **mmol/L** | **mmol/L** | **mmol/L** |
| Male | 0 | 10001 | 7.50 | 0.45 | 1.53 | 4.7 | 141 | 102 |
|  |  | 10002 | 7.66 | 1.61 | 2.65 | 4.7 | 143 | 103 |
|  |  | 10003 | 7.23 | 0.24 | 1.21 | 4.3 | 143 | 106 |
|  |  | 10004 | 9.25 | 1.72 | 2.23 | 5.0 | 143 | 102 |
|  |  | 10005 | 9.58 | 0.57 | 2.48 | 4.8 | 142 | 103 |
|  |  | 10006 | 7.74 | 1.42 | 1.74 | 5.0 | 143 | 103 |
|  |  | 10007 | 8.25 | 1.36 | 2.05 | 4.8 | 142 | 105 |
|  |  | 10008 | 7.83 | 0.72 | 1.39 | 4.9 | 140 | 106 |
|  |  | 10009 | 8.80 | 0.50 | 1.54 | 4.6 | 141 | 106 |
|  |  | 10010 | 7.51 | 0.56 | 1.59 | 4.6 | 143 | 107 |
|  | 0.5 | 11041 | 7.28 | 0.51 | 1.42 | 4.4 | 142 | 108 |
|  |  | 11042 | 9.01 | 1.06 | 1.63 | 5.0 | 141 | 101 |
|  |  | 11043 | 9.34 | 1.05 | 2.24 | 4.9 | 141 | 104 |
|  |  | 11044 | 9.48 | 0.67 | 1.47 | 4.9 | 141 | 104 |
|  |  | 11045 | 10.85 | 1.53 | 1.81 | 4.6 | 143 | 103 |
|  |  | 11046 | 9.56 | 0.23 | 1.41 | 5.0 | 142 | 107 |
|  |  | 11047 | 9.76 | 0.54 | 1.42 | 5.1 | 142 | 105 |
|  |  | 11048 | 6.91 | 0.31 | 1.52 | 5.1 | 143 | 107 |
|  |  | 11049 | 11.13 | 1.09 | 2.33 | 4.7 | 142 | 104 |
|  |  | 11050 | 10.36 | 0.75 | 3.50 | 5.0 | 141 | 100 |
|  | 1.5 | 12081 | 8.71 | 0.63 | 1.40 | 4.9 | 141 | 103 |
|  |  | 12082 | 10.16 | 2.44 | 1.85 | 4.9 | 140 | 103 |
|  |  | 12083 | 8.14 | 0.56 | 0.98 | 5.0 | 140 | 105 |
|  |  | 12084 | 9.94 | 0.54 | 2.29 | 4.8 | 142 | 104 |
|  |  | 12085 | 8.76 | 0.53 | 1.53 | 4.8 | 142 | 103 |
|  |  | 12086 | 7.20 | 0.39 | 1.33 | 4.8 | 144 | 105 |
|  |  | 12087 | 11.00 | 0.80 | 1.80 | 5.1 | 141 | 102 |
|  |  | 12088 | 9.38 | 0.26 | 1.43 | 5.0 | 142 | 106 |
|  |  | 12089 | 8.95 | 0.49 | 1.05 | 4.8 | 142 | 107 |
|  |  | 12090 | 9.38 | 0.77 | 1.75 | 4.8 | 143 | 103 |
|  | 5 | 13121 | 9.12 | 0.61 | 1.55 | 5.3 | 139 | 101 |
|  |  | 13122 | 10.67 | 0.75 | 1.54 | 4.8 | 139 | 99 |
|  |  | 13123 | 9.44 | 0.30 | 1.37 | 4.7 | 139 | 103 |
|  |  | 13124 | 9.40 | 0.33 | 1.87 | 4.9 | 140 | 105 |
|  |  | 13125 | 8.33 | 0.30 | 1.08 | 5.1 | 141 | 104 |
|  |  | 13126 | 12.02 | 0.54 | 1.21 | 5.3 | 141 | 102 |
|  |  | 13127 | 9.38 | 0.46 | 1.57 | 4.8 | 140 | 102 |
|  |  | 13128 | 11.43 | 0.64 | 1.78 | 5.3 | 141 | 104 |
|  |  | 13129 | 10.29 | 1.17 | 1.28 | 5.1 | 141 | 102 |
|  |  | 13130 | 7.71 | 0.34 | 1.28 | 4.8 | 142 | 106 |

### Blood biochemistry data of individual animal

| **Blood biochemistry data on day 92** | | | | | | | | |
| --- | --- | --- | --- | --- | --- | --- | --- | --- |
| **Gender** | **Dose** | **Animal no.** | **TBIL** | **TP** | **ALB** | **GLOB** | **A/G** | **ALT** |
|  | **g/kg** |  | **µmol/L** | **g/L** | **g/L** | **g/L** |  | **U/L** |
| Female | 0 | 20021 | 2.0 | 69.3 | 38.2 | 31.1 | 1.2 | 31 |
|  |  | 20022 | 1.9 | 67.7 | 37.8 | 29.9 | 1.3 | 58 |
|  |  | 20023 | 2.3 | 69.7 | 39.8 | 29.9 | 1.3 | 33 |
|  |  | 20024 | 1.8 | 70.3 | 39.4 | 30.9 | 1.3 | 31 |
|  |  | 20025 | 2.6 | 71.2 | 41.2 | 30.0 | 1.4 | 43 |
|  |  | 20026 | 2.4 | 72.1 | 41.3 | 30.8 | 1.3 | 89 |
|  |  | 20027 | 2.2 | 70.9 | 39.9 | 31.0 | 1.3 | 51 |
|  |  | 20028 | 2.2 | 74.0 | 41.8 | 32.2 | 1.3 | 35 |
|  |  | 20029 | 2.0 | 74.0 | 40.4 | 33.6 | 1.2 | 32 |
|  |  | 20030 | 3.3 | 65.9 | 38.8 | 27.1 | 1.4 | 31 |
|  | 0.5 | 21061 | 2.1 | 62.1 | 35.1 | 27.0 | 1.3 | 50 |
|  |  | 21062 | 1.9 | 68.0 | 38.8 | 29.2 | 1.3 | 126 |
|  |  | 21063 | 1.9 | 66.2 | 36.6 | 29.6 | 1.2 | 30 |
|  |  | 21064 | 2.0 | 76.6 | 43.2 | 33.4 | 1.3 | 24 |
|  |  | 21065 | 1.8 | 70.0 | 40.5 | 29.5 | 1.4 | 36 |
|  |  | 21066 | 3.3 | 70.8 | 39.9 | 30.9 | 1.3 | 37 |
|  |  | 21067 | 2.0 | 75.6 | 42.3 | 33.3 | 1.3 | 31 |
|  |  | 21068 | 1.9 | 67.0 | 36.7 | 30.3 | 1.2 | 23 |
|  |  | 21069 | 2.0 | 73.9 | 41.0 | 32.9 | 1.2 | 54 |
|  |  | 21070 | 2.4 | 67.5 | 39.1 | 28.4 | 1.4 | 38 |
|  | 1.5 | 22101 | 2.2 | 71.5 | 40.8 | 30.7 | 1.3 | 79 |
|  |  | 22102 | 2.3 | 73.9 | 41.6 | 32.3 | 1.3 | 33 |
|  |  | 22103 | 2.4 | 71.8 | 41.6 | 30.2 | 1.4 | 37 |
|  |  | 22104 | 1.9 | 66.0 | 35.4 | 30.6 | 1.2 | 31 |
|  |  | 22105 | 1.9 | 72.5 | 42.1 | 30.4 | 1.4 | 27 |
|  |  | 22106 | 2.6 | 73.4 | 41.5 | 31.9 | 1.3 | 29 |
|  |  | 22107 | 2.0 | 72.9 | 41.0 | 31.9 | 1.3 | 34 |
|  |  | 22108 | 2.0 | 71.4 | 39.0 | 32.4 | 1.2 | 30 |
|  |  | 22109 | 2.2 | 70.1 | 39.1 | 31.0 | 1.3 | 35 |
|  |  | 22110 | 2.4 | 70.3 | 40.5 | 29.8 | 1.4 | 31 |
|  | 5 | 23141 | 2.1 | 65.0 | 36.4 | 28.6 | 1.3 | 25 |
|  |  | 23142 | 1.5 | 68.4 | 38.1 | 30.3 | 1.3 | 34 |
|  |  | 23143 | 3.0 | 80.8 | 45.2 | 35.6 | 1.3 | 25 |
|  |  | 23144 | 2.2 | 69.1 | 38.4 | 30.7 | 1.3 | 23 |
|  |  | 23145 | 2.1 | 66.4 | 38.0 | 28.4 | 1.3 | 22 |
|  |  | 23146 | 2.3 | 77.0 | 44.2 | 32.8 | 1.3 | 31 |
|  |  | 23147 | 1.7 | 67.6 | 37.4 | 30.2 | 1.2 | 23 |
|  |  | 23148 | 2.2 | 68.1 | 38.2 | 29.9 | 1.3 | 28 |
|  |  | 23149 | 2.1 | 68.9 | 38.2 | 30.7 | 1.2 | 33 |
|  |  | 23150 | 2.1 | 67.2 | 37.8 | 29.4 | 1.3 | 22 |

### Blood biochemistry data of individual animal

| **Blood biochemistry data on day 92** | | | | | | | | |
| --- | --- | --- | --- | --- | --- | --- | --- | --- |
| **Gender** | **Dose** | **Animal no.** | **AST** | **ALP** | **GGT** | **CK** | **UREA** | **CREA** |
|  | **g/kg** |  | **U/L** | **U/L** | **U/L** | **U/L** | **mmol/L** | **µmol/L** |
| Female | 0 | 20021 | 142 | 39 | 1 | 545 | 7.62 | 32.3 |
|  |  | 20022 | 191 | 32 | 1 | 541 | 8.26 | 35.0 |
|  |  | 20023 | 93 | 31 | 1 | 212 | 7.94 | 31.5 |
|  |  | 20024 | 108 | 40 | 2 | 340 | 10.73 | 42.6 |
|  |  | 20025 | 98 | 34 | 1 | 207 | 5.25 | 32.1 |
|  |  | 20026 | 241 | 45 | 0 | 294 | 6.33 | 30.8 |
|  |  | 20027 | 146 | 46 | 1 | 378 | 8.67 | 38.1 |
|  |  | 20028 | 111 | 29 | 1 | 299 | 6.44 | 35.2 |
|  |  | 20029 | 137 | 26 | 1 | 438 | 14.47 | 54.3 |
|  |  | 20030 | 96 | 53 | 1 | 203 | 9.12 | 30.6 |
|  | 0.5 | 21061 | 143 | 42 | 1 | 430 | 13.49 | 52.9 |
|  |  | 21062 | 268 | 45 | 1 | 709 | 8.78 | 35.6 |
|  |  | 21063 | 122 | 45 | 1 | 368 | 8.84 | 41.5 |
|  |  | 21064 | 82 | 29 | 2 | 122 | 7.42 | 31.6 |
|  |  | 21065 | 123 | 30 | 0 | 412 | 8.39 | 41.4 |
|  |  | 21066 | 134 | 31 | 0 | 389 | 9.52 | 35.3 |
|  |  | 21067 | 114 | 37 | 1 | 351 | 11.01 | 37.2 |
|  |  | 21068 | 114 | 45 | 0 | 347 | 8.28 | 32.2 |
|  |  | 21069 | 151 | 38 | 2 | 315 | 5.14 | 30.5 |
|  |  | 21070 | 92 | 36 | 0 | 195 | 7.72 | 35.2 |
|  | 1.5 | 22101 | 190 | 52 | 0 | 292 | 7.34 | 29.1 |
|  |  | 22102 | 113 | 33 | 1 | 315 | 7.71 | 46.8 |
|  |  | 22103 | 96 | 28 | 1 | 230 | 7.98 | 26.3 |
|  |  | 22104 | 99 | 40 | 0 | 279 | 7.81 | 34.1 |
|  |  | 22105 | 85 | 30 | 1 | 235 | 8.97 | 26.7 |
|  |  | 22106 | 127 | 37 | 2 | 633 | 8.55 | 50.2 |
|  |  | 22107 | 114 | 37 | 1 | 515 | 10.50 | 36.7 |
|  |  | 22108 | 90 | 32 | 1 | 215 | 7.99 | 30.9 |
|  |  | 22109 | 113 | 59 | 2 | 317 | 9.94 | 37.9 |
|  |  | 22110 | 87 | 38 | 1 | 226 | 6.55 | 28.8 |
|  | 5 | 23141 | 104 | 39 | 0 | 282 | 5.44 | 25.1 |
|  |  | 23142 | 115 | 35 | 1 | 323 | 6.22 | 33.0 |
|  |  | 23143 | 100 | 27 | 1 | 295 | 5.85 | 26.3 |
|  |  | 23144 | 132 | 58 | 1 | 522 | 6.95 | 46.0 |
|  |  | 23145 | 122 | 43 | 2 | 474 | 6.96 | 39.3 |
|  |  | 23146 | 118 | 27 | 1 | 466 | 4.92 | 29.2 |
|  |  | 23147 | 123 | 33 | 2 | 389 | 5.36 | 28.0 |
|  |  | 23148 | 131 | 40 | 2 | 440 | 6.64 | 33.7 |
|  |  | 23149 | 132 | 27 | 0 | 491 | 6.56 | 38.1 |
|  |  | 23150 | 95 | 28 | 1 | 228 | 6.83 | 32.0 |

### Blood biochemistry data of individual animal

| **Blood biochemistry data on day 92** | | | | | | | | |
| --- | --- | --- | --- | --- | --- | --- | --- | --- |
| **Gender** | **Dose** | **Animal no.** | **GLU** | **TG** | **CHOL** | **K^+^** | **Na^+^** | **Cl^-^** |
|  | **g/kg** |  | **mmol/L** | **mmol/L** | **mmol/L** | **mmol/L** | **mmol/L** | **mmol/L** |
| Female | 0 | 20021 | 7.55 | 0.23 | 0.96 | 3.9 | 142 | 105 |
|  |  | 20022 | 6.66 | 0.20 | 0.95 | 4.2 | 141 | 107 |
|  |  | 20023 | 7.53 | 0.46 | 1.38 | 4.0 | 143 | 108 |
|  |  | 20024 | 6.39 | 0.38 | 1.05 | 4.4 | 142 | 111 |
|  |  | 20025 | 8.46 | 0.39 | 1.34 | 4.0 | 142 | 106 |
|  |  | 20026 | 7.30 | 0.36 | 1.27 | 3.6 | 141 | 108 |
|  |  | 20027 | 6.73 | 0.23 | 1.20 | 3.7 | 143 | 108 |
|  |  | 20028 | 6.79 | 0.23 | 1.89 | 3.6 | 142 | 108 |
|  |  | 20029 | 6.11 | 0.23 | 1.88 | 4.4 | 142 | 108 |
|  |  | 20030 | 7.84 | 0.33 | 0.90 | 3.8 | 143 | 109 |
|  | 0.5 | 21061 | 6.58 | 0.27 | 1.21 | 4.2 | 143 | 109 |
|  |  | 21062 | 6.92 | 0.24 | 2.00 | 4.3 | 141 | 107 |
|  |  | 21063 | 6.59 | 0.14 | 1.31 | 4.3 | 143 | 109 |
|  |  | 21064 | 8.21 | 0.24 | 1.84 | 3.9 | 142 | 107 |
|  |  | 21065 | 8.74 | 0.34 | 1.74 | 4.2 | 141 | 108 |
|  |  | 21066 | 6.12 | 0.33 | 1.47 | 4.1 | 141 | 107 |
|  |  | 21067 | 7.26 | 0.41 | 1.77 | 4.4 | 142 | 107 |
|  |  | 21068 | 7.39 | 0.48 | 2.20 | 4.1 | 142 | 108 |
|  |  | 21069 | 7.95 | 0.20 | 1.89 | 4.3 | 141 | 108 |
|  |  | 21070 | 6.75 | 0.22 | 1.22 | 4.2 | 142 | 109 |
|  | 1.5 | 22101 | 8.10 | 0.19 | 1.95 | 4.3 | 142 | 107 |
|  |  | 22102 | 9.80 | 0.20 | 1.29 | 4.7 | 144 | 104 |
|  |  | 22103 | 8.35 | 0.32 | 1.54 | 4.5 | 140 | 107 |
|  |  | 22104 | 6.38 | 0.14 | 1.36 | 4.3 | 142 | 106 |
|  |  | 22105 | 6.16 | 0.23 | 1.15 | 4.1 | 142 | 107 |
|  |  | 22106 | 6.89 | 0.42 | 1.63 | 4.3 | 140 | 106 |
|  |  | 22107 | 7.18 | 0.25 | 2.30 | 4.1 | 141 | 107 |
|  |  | 22108 | 6.93 | 0.19 | 1.64 | 4.2 | 142 | 107 |
|  |  | 22109 | 6.01 | 0.32 | 1.31 | 4.3 | 143 | 106 |
|  |  | 22110 | 8.13 | 0.58 | 1.69 | 4.3 | 143 | 107 |
|  | 5 | 23141 | 7.70 | 0.17 | 1.19 | 4.5 | 141 | 105 |
|  |  | 23142 | 5.23 | 0.16 | 1.46 | 4.3 | 144 | 109 |
|  |  | 23143 | 7.73 | 0.24 | 2.75 | 4.6 | 140 | 106 |
|  |  | 23144 | 6.46 | 0.25 | 2.28 | 4.5 | 140 | 106 |
|  |  | 23145 | 7.48 | 0.19 | 2.24 | 4.5 | 142 | 109 |
|  |  | 23146 | 7.74 | 0.18 | 1.65 | 4.1 | 141 | 104 |
|  |  | 23147 | 7.20 | 0.19 | 1.84 | 4.1 | 141 | 107 |
|  |  | 23148 | 6.14 | 0.24 | 1.91 | 4.1 | 141 | 104 |
|  |  | 23149 | 7.27 | 0.27 | 1.84 | 4.6 | 141 | 106 |
|  |  | 23150 | 7.35 | 0.36 | 1.38 | 4.7 | 141 | 106 |

### Blood biochemistry data of individual animal

| **Blood biochemistry data on recovery day 29** | | | | | | | | |
| --- | --- | --- | --- | --- | --- | --- | --- | --- |
| **Gender** | **Dose** | **Animal no.** | **TBIL** | **TP** | **ALB** | **GLOB** | **A/G** | **ALT** |
|  | **g/kg** |  | **µmol/L** | **g/L** | **g/L** | **g/L** |  | **U/L** |
| Male | 0 | 10011 | 2.2 | 65.4 | 34.6 | 30.8 | 1.1 | 54 |
|  |  | 10012 | 2.7 | 68.0 | 35.6 | 32.4 | 1.1 | 33 |
|  |  | 10013 | 1.9 | 67.5 | 35.5 | 32.0 | 1.1 | 39 |
|  |  | 10014 | 2.4 | 68.7 | 34.4 | 34.3 | 1.0 | 39 |
|  |  | 10015 | 2.9 | 69.2 | 36.6 | 32.6 | 1.1 | 62 |
|  | 0.5 | 11051 | 2.9 | 62.9 | 34.1 | 28.8 | 1.2 | 39 |
|  |  | 11052 | 2.2 | 62.8 | 33.9 | 28.9 | 1.2 | 31 |
|  |  | 11053 | 2.2 | 61.4 | 32.4 | 29.0 | 1.1 | 36 |
|  |  | 11054 | 2.3 | 68.7 | 37.3 | 31.4 | 1.2 | 46 |
|  |  | 11055 | 2.6 | 69.1 | 35.5 | 33.6 | 1.1 | 37 |
|  | 1.5 | 12091 | 2.2 | 66.4 | 34.5 | 31.9 | 1.1 | 74 |
|  |  | 12092 | 2.4 | 66.3 | 35.3 | 31.0 | 1.1 | 25 |
|  |  | 12093 | 2.5 | 62.4 | 33.6 | 28.8 | 1.2 | 49 |
|  |  | 12094 | 2.6 | 66.9 | 34.4 | 32.5 | 1.1 | 36 |
|  |  | 12095 | 2.4 | 63.8 | 34.5 | 29.3 | 1.2 | 41 |
|  | 5 | 13131 | 2.3 | 69.8 | 36.6 | 33.2 | 1.1 | 39 |
|  |  | 13132 | 2.1 | 68.0 | 36.7 | 31.3 | 1.2 | 33 |
|  |  | 13133 | 2.1 | 59.9 | 32.9 | 27.0 | 1.2 | 42 |
|  |  | 13134 | 2.5 | 62.1 | 34.0 | 28.1 | 1.2 | 28 |
|  |  | 13135 | 2.8 | 68.3 | 35.0 | 33.3 | 1.1 | 37 |
| Female | 0 | 20031 | 3.5 | 78.3 | 43.1 | 35.2 | 1.2 | 28 |
|  |  | 20032 | 2.9 | 71.9 | 38.2 | 33.7 | 1.1 | 35 |
|  |  | 20033 | 2.4 | 78.5 | 42.9 | 35.6 | 1.2 | 30 |
|  |  | 20034 | 4.4 | 83.9 | 47.2 | 36.7 | 1.3 | 35 |
|  |  | 20035 | 2.6 | 74.4 | 41.6 | 32.8 | 1.3 | 45 |
|  | 0.5 | 21071 | 3.1 | 67.9 | 39.5 | 28.4 | 1.4 | 27 |
|  |  | 21072 | 2.7 | 70.4 | 39.3 | 31.1 | 1.3 | 55 |
|  |  | 21073 | 3.0 | 66.2 | 35.7 | 30.5 | 1.2 | 26 |
|  |  | 21074 | 3.2 | 67.3 | 36.7 | 30.6 | 1.2 | 29 |
|  |  | 21075 | 3.9 | 76.9 | 42.5 | 34.4 | 1.2 | 72 |
|  | 1.5 | 22111 | 3.8 | 70.3 | 38.8 | 31.5 | 1.2 | 31 |
|  |  | 22112 | 2.9 | 66.8 | 36.3 | 30.5 | 1.2 | 47 |
|  |  | 22113 | 3.0 | 76.9 | 42.1 | 34.8 | 1.2 | 28 |
|  |  | 22114 | 2.5 | 70.9 | 39.0 | 31.9 | 1.2 | 165 |
|  |  | 22115 | 3.0 | 74.8 | 42.0 | 32.8 | 1.3 | 71 |
|  | 5 | 23151 | 3.2 | 78.6 | 44.2 | 34.4 | 1.3 | 46 |
|  |  | 23152 | 4.1 | 75.1 | 42.0 | 33.1 | 1.3 | 28 |
|  |  | 23153 | 3.7 | 73.5 | 40.9 | 32.6 | 1.3 | 33 |
|  |  | 23154 | 3.6 | 70.9 | 39.8 | 31.1 | 1.3 | 45 |
|  |  | 23155 | 2.8 | 73.0 | 42.2 | 30.8 | 1.4 | 36 |

### Blood biochemistry data of individual animal

| **Blood biochemistry data on recovery day 29** | | | | | | | | |
| --- | --- | --- | --- | --- | --- | --- | --- | --- |
| **Gender** | **Dose** | **Animal no.** | **AST** | **ALP** | **GGT** | **CK** | **UREA** | **CREA** |
|  | **g/kg** |  | **U/L** | **U/L** | **U/L** | **U/L** | **mmol/L** | **µmol/L** |
| Male | 0 | 10011 | 168 | 65 | 2 | 498 | 7.11 | 36.4 |
|  |  | 10012 | 139 | 57 | 0 | 354 | 5.84 | 38.0 |
|  |  | 10013 | 93 | 80 | 0 | 190 | 7.22 | 34.7 |
|  |  | 10014 | 141 | 74 | 0 | 475 | 7.64 | 23.6 |
|  |  | 10015 | 178 | 70 | 1 | 445 | 8.28 | 29.3 |
|  | 0.5 | 11051 | 162 | 96 | 0 | 483 | 7.71 | 40.1 |
|  |  | 11052 | 105 | 58 | 0 | 300 | 6.27 | 30.0 |
|  |  | 11053 | 119 | 118 | 0 | 250 | 7.69 | 32.3 |
|  |  | 11054 | 198 | 88 | 1 | 640 | 7.00 | 20.8 |
|  |  | 11055 | 166 | 83 | 0 | 428 | 6.23 | 22.4 |
|  | 1.5 | 12091 | 127 | 73 | 1 | 277 | 7.44 | 26.6 |
|  |  | 12092 | 83 | 75 | 1 | 204 | 5.90 | 30.0 |
|  |  | 12093 | 154 | 72 | 0 | 366 | 5.67 | 32.3 |
|  |  | 12094 | 161 | 84 | 1 | 463 | 7.37 | 26.2 |
|  |  | 12095 | 143 | 56 | 1 | 476 | 7.60 | 24.0 |
|  | 5 | 13131 | 99 | 88 | 0 | 209 | 8.56 | 34.6 |
|  |  | 13132 | 82 | 63 | 0 | 106 | 6.07 | 27.3 |
|  |  | 13133 | 129 | 60 | 2 | 401 | 7.00 | 27.4 |
|  |  | 13134 | 122 | 68 | 0 | 417 | 7.22 | 25.9 |
|  |  | 13135 | 118 | 92 | 0 | 321 | 5.91 | 21.4 |
| Female | 0 | 20031 | 108 | 46 | 0 | 337 | 7.45 | 28.3 |
|  |  | 20032 | 136 | 46 | 0 | 463 | 7.89 | 27.6 |
|  |  | 20033 | 120 | 31 | 0 | 590 | 5.00 | 26.7 |
|  |  | 20034 | 82 | 35 | 0 | 169 | 7.14 | 31.8 |
|  |  | 20035 | 103 | 34 | 0 | 234 | 8.04 | 36.8 |
|  | 0.5 | 21071 | 104 | 32 | 1 | 270 | 7.99 | 39.0 |
|  |  | 21072 | 153 | 42 | 1 | 444 | 7.90 | 37.7 |
|  |  | 21073 | 135 | 35 | 1 | 456 | 8.32 | 34.2 |
|  |  | 21074 | 102 | 37 | 1 | 218 | 7.43 | 33.0 |
|  |  | 21075 | 190 | 23 | 1 | 245 | 8.38 | 34.2 |
|  | 1.5 | 22111 | 92 | 35 | 1 | 278 | 6.78 | 32.5 |
|  |  | 22112 | 122 | 43 | 0 | 188 | 6.41 | 31.5 |
|  |  | 22113 | 74 | 32 | 0 | 204 | 9.53 | 32.7 |
|  |  | 22114 | 256 | 41 | 1 | 290 | 9.35 | 34.1 |
|  |  | 22115 | 158 | 46 | 1 | 215 | 7.20 | 31.5 |
|  | 5 | 23151 | 101 | 33 | 0 | 136 | 6.02 | 32.4 |
|  |  | 23152 | 91 | 42 | 1 | 225 | 6.90 | 28.4 |
|  |  | 23153 | 77 | 23 | 2 | 154 | 7.14 | 28.2 |
|  |  | 23154 | 93 | 37 | 0 | 195 | 8.61 | 35.4 |
|  |  | 23155 | 71 | 42 | 1 | 119 | 9.79 | 41.3 |

### Blood biochemistry data of individual animal

| **Blood biochemistry data on recovery day 29** | | | | | | | | |
| --- | --- | --- | --- | --- | --- | --- | --- | --- |
| **Gender** | **Dose** | **Animal no.** | **GLU** | **TG** | **CHOL** | **K^+^** | **Na^+^** | **Cl^-^** |
|  | **g/kg** |  | **mmol/L** | **mmol/L** | **mmol/L** | **mmol/L** | **mmol/L** | **mmol/L** |
| Male | 0 | 10011 | 11.25 | 0.66 | 1.20 | 4.4 | 146 | 107 |
|  |  | 10012 | 10.06 | 0.65 | 1.41 | 5.0 | 145 | 108 |
|  |  | 10013 | 11.27 | 0.60 | 1.62 | 4.5 | 146 | 106 |
|  |  | 10014 | 7.87 | 0.81 | 1.72 | 4.9 | 145 | 107 |
|  |  | 10015 | 7.75 | 0.81 | 2.36 | 4.9 | 146 | 107 |
|  | 0.5 | 11051 | 9.72 | 0.44 | 1.35 | 4.9 | 144 | 105 |
|  |  | 11052 | 10.68 | 0.74 | 1.73 | 4.4 | 145 | 109 |
|  |  | 11053 | 9.83 | 0.39 | 1.44 | 4.8 | 145 | 109 |
|  |  | 11054 | 7.69 | 2.05 | 2.14 | 4.8 | 146 | 106 |
|  |  | 11055 | 7.93 | 0.53 | 1.71 | 4.2 | 148 | 108 |
|  | 1.5 | 12091 | 11.47 | 0.55 | 1.57 | 4.6 | 145 | 106 |
|  |  | 12092 | 12.10 | 0.43 | 1.25 | 4.8 | 145 | 106 |
|  |  | 12093 | 10.05 | 0.32 | 1.27 | 4.3 | 146 | 108 |
|  |  | 12094 | 8.28 | 1.24 | 1.59 | 4.4 | 148 | 107 |
|  |  | 12095 | 7.36 | 0.46 | 1.43 | 4.8 | 147 | 107 |
|  | 5 | 13131 | 14.61 | 0.68 | 2.24 | 4.7 | 144 | 104 |
|  |  | 13132 | 10.57 | 0.31 | 1.45 | 4.5 | 145 | 108 |
|  |  | 13133 | 8.73 | 0.29 | 1.34 | 4.9 | 146 | 109 |
|  |  | 13134 | 7.25 | 0.50 | 1.46 | 4.3 | 147 | 111 |
|  |  | 13135 | 9.14 | 1.25 | 1.83 | 4.6 | 146 | 107 |
| Female | 0 | 20031 | 7.57 | 0.30 | 1.87 | 3.8 | 145 | 109 |
|  |  | 20032 | 7.59 | 0.28 | 1.20 | 4.0 | 145 | 111 |
|  |  | 20033 | 9.18 | 0.36 | 3.14 | 4.2 | 143 | 108 |
|  |  | 20034 | 8.51 | 0.61 | 2.40 | 4.5 | 144 | 108 |
|  |  | 20035 | 7.79 | 0.25 | 1.60 | 4.2 | 146 | 110 |
|  | 0.5 | 21071 | 8.68 | 0.82 | 1.08 | 4.0 | 147 | 108 |
|  |  | 21072 | 7.60 | 0.23 | 1.76 | 4.1 | 147 | 111 |
|  |  | 21073 | 6.07 | 0.18 | 0.58 | 4.3 | 146 | 111 |
|  |  | 21074 | 7.43 | 0.23 | 1.41 | 4.1 | 145 | 110 |
|  |  | 21075 | 9.10 | 0.41 | 2.01 | 4.4 | 145 | 108 |
|  | 1.5 | 22111 | 8.13 | 0.37 | 1.31 | 4.2 | 145 | 109 |
|  |  | 22112 | 7.76 | 0.16 | 1.78 | 4.0 | 146 | 109 |
|  |  | 22113 | 8.21 | 0.46 | 1.72 | 4.2 | 143 | 109 |
|  |  | 22114 | 9.21 | 0.24 | 1.48 | 4.3 | 145 | 110 |
|  |  | 22115 | 9.19 | 0.22 | 2.03 | 4.6 | 144 | 109 |
|  | 5 | 23151 | 7.74 | 0.29 | 1.68 | 4.4 | 145 | 109 |
|  |  | 23152 | 7.84 | 0.37 | 1.50 | 4.1 | 146 | 110 |
|  |  | 23153 | 8.09 | 0.25 | 1.75 | 4.1 | 145 | 109 |
|  |  | 23154 | 8.08 | 0.31 | 1.84 | 4.7 | 145 | 111 |
|  |  | 23155 | 7.92 | 0.18 | 1.15 | 4.1 | 146 | 109 |

### Urine data of individual animal

| **Urine data on day 27** | | | | | | | |
| --- | --- | --- | --- | --- | --- | --- | --- |
| **Gender** | **Dose** | **Animal no.** | **COL** | **TURB** | **pH** | **NIT** | **GLU** |
|  | **g/kg** |  |  |  |  |  |  |
| Male | 0 | 10016 | COLORLESS | - | 8.5 | - | NORMAL |
|  |  | 10017 | COLORLESS | +1 | 8.5 | - | NORMAL |
|  |  | 10018 | COLORLESS | - | 8.5 | - | NORMAL |
|  |  | 10019 | COLORLESS | +1 | 8.5 | +1 | NORMAL |
|  |  | 10020 | COLORLESS | - | 8.5 | - | NORMAL |
|  | 0.5 | 11056 | COLORLESS | +1 | 8.5 | +1 | NORMAL |
|  |  | 11057 | COLORLESS | +1 | 8.5 | +1 | NORMAL |
|  |  | 11058 | COLORLESS | +1 | 8.5 | +1 | NORMAL |
|  |  | 11059 | COLORLESS | +1 | 8.5 | +1 | NORMAL |
|  |  | 11060 | COLORLESS | +1 | 8.5 | +1 | NORMAL |
|  | 1.5 | 12096 | LIGHT RED | +1 | 8.5 | - | NORMAL |
|  |  | 12097 | COLORLESS | +1 | 8.5 | - | NORMAL |
|  |  | 12098 | COLORLESS | +1 | 8.5 | - | NORMAL |
|  |  | 12099 | COLORLESS | - | 8.0 | - | NORMAL |
|  |  | 12100 | COLORLESS | +1 | 8.5 | - | NORMAL |
|  | 5 | 13136 | COLORLESS | +1 | 8.5 | - | NORMAL |
|  |  | 13137 | YELLOW | +1 | 8.0 | +1 | NORMAL |
|  |  | 13138 | LIGHT YELLOW | +1 | 8.0 | +1 | NORMAL |
|  |  | 13139 | YELLOW | +1 | 8.5 | +1 | NORMAL |
|  |  | 13140 | YELLOW | +1 | 8.0 | +1 | NORMAL |
| Female | 0 | 20036 | COLORLESS | - | 8.5 | +1 | NORMAL |
|  |  | 20037 | LIGHT YELLOW | - | 8.5 | - | NORMAL |
|  |  | 20038 | COLORLESS | +1 | 8.0 | +1 | NORMAL |
|  |  | 20039 | COLORLESS | +1 | 8.5 | +1 | NORMAL |
|  |  | 20040 | YELLOW | +1 | 9.0 | - | NORMAL |
|  | 0.5 | 21076 | COLORLESS | - | 8.0 | - | NORMAL |
|  |  | 21077 | COLORLESS | +1 | 8.5 | +1 | NORMAL |
|  |  | 21078 | COLORLESS | +1 | 8.5 | +1 | NORMAL |
|  |  | 21079 | LIGHT RED | - | 8.0 | - | NORMAL |
|  |  | 21080 | COLORLESS | +1 | 8.5 | +1 | NORMAL |
|  | 1.5 | 22116 | LIGHT RED | +1 | 8.0 | - | NORMAL |
|  |  | 22117 | COLORLESS | - | 8.5 | - | NORMAL |
|  |  | 22118 | COLORLESS | - | 8.5 | - | NORMAL |
|  |  | 22119 | COLORLESS | +1 | 8.5 | - | NORMAL |
|  |  | 22120 | LIGHT YELLOW | +1 | 8.5 | +2 | NORMAL |
|  | 5 | 23156 | YELLOW | +1 | 8.5 | +2 | NORMAL |
|  |  | 23157 | LIGHT YELLOW | - | 8.0 | +1 | NORMAL |
|  |  | 23158 | YELLOW | +1 | 8.0 | +2 | NORMAL |
|  |  | 23159 | LIGHT YELLOW | +1 | 7.5 | - | NORMAL |
|  |  | 23160 | COLORLESS | - | 7.5 | - | NORMAL |

### Urine data of individual animal

| **Urine data on day 27** | | | | | | | | | |
| --- | --- | --- | --- | --- | --- | --- | --- | --- | --- |
| **Gender** | **Dose** | **Animal no.** | **SG** | **BLD** | **PRO** | **BIL** | **URO** | **KET** | **LEU** |
|  | **g/kg** |  |  |  |  |  |  |  | **Leu/uL** |
| Male | 0 | 10016 | 1.017 | - | +1 | - | NORMAL | - | 250 |
|  |  | 10017 | 1.009 | - | +- | - | NORMAL | - | 75 |
|  |  | 10018 | 1.019 | - | +1 | - | NORMAL | - | 75 |
|  |  | 10019 | 1.007 | - | +- | - | NORMAL | - | 25 |
|  |  | 10020 | 1.016 | - | +1 | - | NORMAL | - | 25 |
|  | 0.5 | 11056 | 1.009 | - | +- | - | NORMAL | - | NEG. |
|  |  | 11057 | 1.009 | - | +1 | - | NORMAL | - | NEG. |
|  |  | 11058 | 1.014 | - | +- | - | NORMAL | - | 25 |
|  |  | 11059 | 1.010 | - | +1 | - | NORMAL | - | 25 |
|  |  | 11060 | 1.007 | - | +- | - | NORMAL | - | NEG. |
|  | 1.5 | 12096 | 1.017 | - | +- | - | NORMAL | - | 25 |
|  |  | 12097 | 1.011 | - | +- | - | NORMAL | - | 25 |
|  |  | 12098 | 1.021 | +- | +1 | - | NORMAL | - | 75 |
|  |  | 12099 | 1.019 | - | +- | - | NORMAL | - | 75 |
|  |  | 12100 | 1.015 | +- | +1 | - | NORMAL | - | 25 |
|  | 5 | 13136 | 1.027 | - | +1 | - | NORMAL | - | 250 |
|  |  | 13137 | 1.040 | - | +1 | - | NORMAL | - | 75 |
|  |  | 13138 | 1.030 | - | +1 | - | NORMAL | - | 75 |
|  |  | 13139 | 1.034 | - | +1 | - | NORMAL | - | 75 |
|  |  | 13140 | 1.033 | - | +1 | - | NORMAL | - | 75 |
| Female | 0 | 20036 | 1.013 | - | - | - | NORMAL | - | NEG. |
|  |  | 20037 | 1.019 | - | +- | - | NORMAL | - | NEG. |
|  |  | 20038 | 1.016 | - | - | - | NORMAL | - | NEG. |
|  |  | 20039 | 1.010 | - | - | - | NORMAL | - | NEG. |
|  |  | 20040 | 1.032 | - | +- | - | +1 | - | NEG. |
|  | 0.5 | 21076 | 1.008 | - | - | - | NORMAL | - | NEG. |
|  |  | 21077 | 1.011 | - | - | - | NORMAL | - | NEG. |
|  |  | 21078 | 1.009 | - | - | - | NORMAL | - | NEG. |
|  |  | 21079 | 1.011 | - | - | - | NORMAL | - | NEG. |
|  |  | 21080 | 1.017 | - | - | - | NORMAL | - | NEG. |
|  | 1.5 | 22116 | 1.036 | - | +1 | - | NORMAL | - | NEG. |
|  |  | 22117 | 1.015 | - | - | - | NORMAL | - | 25 |
|  |  | 22118 | 1.020 | - | +- | - | NORMAL | - | 75 |
|  |  | 22119 | 1.017 | - | - | - | NORMAL | - | NEG. |
|  |  | 22120 | 1.019 | - | +- | - | NORMAL | - | NEG. |
|  | 5 | 23156 | 1.033 | - | +- | - | NORMAL | - | 25 |
|  |  | 23157 | 1.030 | - | +- | - | NORMAL | - | NEG. |
|  |  | 23158 | 1.044 | - | +1 | - | NORMAL | - | NEG. |
|  |  | 23159 | 1.034 | - | +- | - | NORMAL | - | 25 |
|  |  | 23160 | 1.031 | - | +- | - | NORMAL | - | 25 |

### Urine data of individual animal

| **Urine data on 90** | | | | | | | |
| --- | --- | --- | --- | --- | --- | --- | --- |
| **Gender** | **Dose** | **Animal no.** | **COL** | **TURB** | **pH** | **NIT** | **GLU** |
|  | **g/kg** |  |  |  |  |  |  |
| Male | 0 | 10001 | COLORLESS | - | 8.5 | - | NORMAL |
|  |  | 10002 | COLORLESS | - | 8.5 | - | NORMAL |
|  |  | 10003 | COLORLESS | +1 | 8.5 | +1 | NORMAL |
|  |  | 10004 | RED | +1 | 8.5 | +2 | NORMAL |
|  |  | 10005 | COLORLESS | - | 8.0 | - | NORMAL |
|  |  | 10006 | COLORLESS | +1 | 8.5 | +1 | NORMAL |
|  |  | 10007 | COLORLESS | +1 | 8.5 | - | NORMAL |
|  |  | 10008 | COLORLESS | +1 | 8.5 | - | NORMAL |
|  |  | 10009 | YELLOW | +1 | 8.5 | - | NORMAL |
|  |  | 10010 | COLORLESS | +1 | 8.5 | - | NORMAL |
|  | 0.5 | 11041 | YELLOW | +1 | 8.5 | - | NORMAL |
|  |  | 11042 | COLORLESS | +1 | 8.5 | - | NORMAL |
|  |  | 11043 | YELLOW | +1 | 8.5 | - | NORMAL |
|  |  | 11044 | COLORLESS | +1 | 8.5 | +1 | NORMAL |
|  |  | 11045 | COLORLESS | - | 8.5 | - | NORMAL |
|  |  | 11046 | COLORLESS | - | 8.5 | - | NORMAL |
|  |  | 11047 | YELLOW | +1 | 8.5 | - | NORMAL |
|  |  | 11048 | COLORLESS | +1 | 8.5 | +1 | NORMAL |
|  |  | 11049 | LIGHT YELLOW | +1 | 8.5 | - | NORMAL |
|  |  | 11050 | COLORLESS | +1 | 8.5 | - | NORMAL |
|  | 1.5 | 12081 | YELLOW | +2 | 8.5 | - | NORMAL |
|  |  | 12082 | YELLOW | +2 | 8.5 | - | NORMAL |
|  |  | 12083 | LIGHT YELLOW | +1 | 6.5 | - | NORMAL |
|  |  | 12084 | YELLOW | +1 | 8.5 | +1 | NORMAL |
|  |  | 12085 | LIGHT YELLOW | +1 | 8.0 | +1 | NORMAL |
|  |  | 12086 | LIGHT YELLOW | +1 | 7.5 | - | NORMAL |
|  |  | 12087 | YELLOW | - | 8.5 | +1 | NORMAL |
|  |  | 12088 | COLORLESS | - | 8.0 | - | NORMAL |
|  |  | 12089 | RED | +2 | 8.0 | - | NORMAL |
|  |  | 12090 | COLORLESS | +1 | 8.0 | - | NORMAL |
|  | 5 | 13121 | LIGHT YELLOW | - | 8.0 | +1 | NORMAL |
|  |  | 13122 | YELLOW | +1 | 8.5 | - | NORMAL |
|  |  | 13123 | LIGHT YELLOW | +1 | 7.5 | +1 | NORMAL |
|  |  | 13124 | DARK BROWN | +1 | 7.0 | +2 | NORMAL |
|  |  | 13125 | COLORLESS | - | 6.0 | - | NORMAL |
|  |  | 13126 | COLORLESS | - | 8.0 | - | NORMAL |
|  |  | 13127 | RED | +1 | 8.5 | - | NORMAL |
|  |  | 13128 | COLORLESS | +1 | 6.5 | - | NORMAL |
|  |  | 13129 | YELLOW | +1 | 8.0 | - | NORMAL |
|  |  | 13130 | YELLOW | +1 | 8.5 | - | NORMAL |

### Urine data of individual animal

| **Urine data on 90** | | | | | | | | | |
| --- | --- | --- | --- | --- | --- | --- | --- | --- | --- |
| **Gender** | **Dose** | **Animal no.** | **SG** | **BLD** | **PRO** | **BIL** | **URO** | **KET** | **LEU** |
|  | **g/kg** |  |  |  |  |  |  |  | **Leu/uL** |
| Male | 0 | 10001 | 1.007 | +- | +1 | - | NORMAL | - | 25 |
|  |  | 10002 | 1.010 | - | +1 | - | NORMAL | - | 250 |
|  |  | 10003 | 1.018 | - | +1 | - | NORMAL | - | 25 |
|  |  | 10004 | 1.013 | - | +1 | - | NORMAL | - | 250 |
|  |  | 10005 | 1.003 | +- | - | - | NORMAL | - | 25 |
|  |  | 10006 | 1.010 | - | +1 | - | NORMAL | - | 250 |
|  |  | 10007 | 1.009 | - | +- | - | NORMAL | - | 25 |
|  |  | 10008 | 1.010 | - | +1 | - | NORMAL | - | 75 |
|  |  | 10009 | 1.015 | +- | +2 | - | NORMAL | - | 500 |
|  |  | 10010 | 1.008 | - | +- | - | NORMAL | - | 25 |
|  | 0.5 | 11041 | 1.039 | +1 | +1 | - | +1 | +- | 250 |
|  |  | 11042 | 1.009 | - | +1 | - | NORMAL | - | 75 |
|  |  | 11043 | 1.005 | +- | +- | - | NORMAL | - | NEG. |
|  |  | 11044 | 1.021 | - | +1 | - | NORMAL | - | 75 |
|  |  | 11045 | 1.019 | - | +1 | - | NORMAL | - | 500 |
|  |  | 11046 | 1.019 | - | +1 | - | NORMAL | - | 250 |
|  |  | 11047 | 1.017 | - | +1 | - | NORMAL | - | 250 |
|  |  | 11048 | 1.012 | +- | +1 | - | NORMAL | - | 25 |
|  |  | 11049 | 1.024 | - | +1 | - | NORMAL | +- | 75 |
|  |  | 11050 | 1.016 | - | +2 | - | NORMAL | - | 500 |
|  | 1.5 | 12081 | 1.025 | +1 | +1 | - | NORMAL | - | 75 |
|  |  | 12082 | 1.033 | +- | +1 | - | NORMAL | - | 75 |
|  |  | 12083 | 1.027 | - | +1 | - | NORMAL | - | 75 |
|  |  | 12084 | 1.032 | - | +1 | - | NORMAL | - | 75 |
|  |  | 12085 | 1.013 | - | +1 | - | NORMAL | - | NEG. |
|  |  | 12086 | 1.024 | - | +1 | - | NORMAL | - | 75 |
|  |  | 12087 | 1.030 | - | +1 | - | NORMAL | - | 25 |
|  |  | 12088 | 1.011 | - | - | - | NORMAL | - | NEG. |
|  |  | 12089 | 1.024 | +1 | +1 | - | NORMAL | - | 75 |
|  |  | 12090 | 1.011 | +- | +- | - | NORMAL | - | 25 |
|  | 5 | 13121 | 1.026 | - | +1 | - | NORMAL | - | 75 |
|  |  | 13122 | 1.030 | - | +- | - | NORMAL | - | 75 |
|  |  | 13123 | 1.026 | +1 | +- | - | NORMAL | - | NEG. |
|  |  | 13124 | 1.036 | +1 | +1 | - | NORMAL | - | 75 |
|  |  | 13125 | 1.018 | - | +- | - | NORMAL | - | 75 |
|  |  | 13126 | 1.017 | +2 | +- | - | NORMAL | - | 25 |
|  |  | 13127 | 1.042 | - | +1 | - | NORMAL | - | 25 |
|  |  | 13128 | 1.012 | +- | - | - | NORMAL | - | 25 |
|  |  | 13129 | 1.033 | - | +1 | - | NORMAL | - | 250 |
|  |  | 13130 | 1.043 | - | +1 | - | +1 | +- | 250 |

### Urine data of individual animal

| **Urine data on 90** | | | | | | | |
| --- | --- | --- | --- | --- | --- | --- | --- |
| **Gender** | **Dose** | **Animal no.** | **COL** | **TURB** | **pH** | **NIT** | **GLU** |
|  | **g/kg** |  |  |  |  |  |  |
| Female | 0 | 20021 | COLORLESS | - | 8.5 | +1 | NORMAL |
|  |  | 20022 | LIGHT YELLOW | - | 8.5 | +1 | NORMAL |
|  |  | 20023 | COLORLESS | - | 8.5 | - | NORMAL |
|  |  | 20024 | COLORLESS | - | 8.5 | +1 | NORMAL |
|  |  | 20025 | COLORLESS | - | 8.5 | +1 | NORMAL |
|  |  | 20026 | LIGHT RED | +1 | 8.5 | +1 | NORMAL |
|  |  | 20027 | COLORLESS | +1 | 8.5 | +2 | NORMAL |
|  |  | 20028 | COLORLESS | - | 8.5 | - | NORMAL |
|  |  | 20029 | COLORLESS | - | 8.5 | +1 | NORMAL |
|  |  | 20030 | COLORLESS | - | 8.5 | +1 | NORMAL |
|  | 0.5 | 21061 | LIGHT YELLOW | - | 8.0 | +1 | NORMAL |
|  |  | 21062 | COLORLESS | - | 8.5 | - | NORMAL |
|  |  | 21063 | COLORLESS | - | 8.0 | +1 | NORMAL |
|  |  | 21064 | COLORLESS | - | 8.0 | - | NORMAL |
|  |  | 21065 | COLORLESS | +1 | 7.5 | +1 | NORMAL |
|  |  | 21066 | COLORLESS | +1 | 7.5 | +1 | NORMAL |
|  |  | 21067 | COLORLESS | +1 | 8.0 | - | NORMAL |
|  |  | 21068 | COLORLESS | +1 | 8.5 | - | NORMAL |
|  |  | 21069 | COLORLESS | - | 8.0 | - | NORMAL |
|  |  | 21070 | COLORLESS | - | 8.5 | +1 | NORMAL |
|  | 1.5 | 22101 | COLORLESS | +1 | 7.5 | - | NORMAL |
|  |  | 22102 | COLORLESS | - | 7.5 | - | NORMAL |
|  |  | 22103 | LIGHT YELLOW | +1 | 6.5 | - | NORMAL |
|  |  | 22104 | COLORLESS | +1 | 7.0 | - | NORMAL |
|  |  | 22105 | COLORLESS | - | 7.5 | - | NORMAL |
|  |  | 22106 | COLORLESS | - | 8.0 | +1 | NORMAL |
|  |  | 22107 | COLORLESS | - | 8.0 | +1 | NORMAL |
|  |  | 22108 | COLORLESS | +1 | 8.0 | +1 | NORMAL |
|  |  | 22109 | COLORLESS | - | 6.5 | - | NORMAL |
|  |  | 22110 | COLORLESS | +1 | 7.5 | +1 | NORMAL |
|  | 5 | 23141 | YELLOW | +1 | 8.0 | +2 | NORMAL |
|  |  | 23142 | LIGHT YELLOW | - | 8.5 | +2 | NORMAL |
|  |  | 23143 | LIGHT YELLOW | - | 8.0 | +1 | NORMAL |
|  |  | 23144 | LIGHT YELLOW | - | 7.5 | +1 | NORMAL |
|  |  | 23145 | COLORLESS | - | 7.5 | - | NORMAL |
|  |  | 23146 | LIGHT YELLOW | +1 | 8.0 | +2 | NORMAL |
|  |  | 23147 | LIGHT YELLOW | +2 | 7.5 | +1 | NORMAL |
|  |  | 23148 | LIGHT YELLOW | +1 | 8.5 | +1 | NORMAL |
|  |  | 23149 | YELLOW | - | 8.0 | +1 | NORMAL |
|  |  | 23150 | YELLOW | +2 | 8.5 | +2 | NORMAL |

### Urine data of individual animal

| **Urine data on 90** | | | | | | | | | |
| --- | --- | --- | --- | --- | --- | --- | --- | --- | --- |
| **Gender** | **Dose** | **Animal no.** | **SG** | **BLD** | **PRO** | **BIL** | **URO** | **KET** | **LEU** |
|  | **g/kg** |  |  |  |  |  |  |  | **Leu/uL** |
| Female | 0 | 20021 | 1.004 | +2 | - | - | NORMAL | - | NEG. |
|  |  | 20022 | 1.021 | - | +- | - | NORMAL | - | NEG. |
|  |  | 20023 | 1.007 | - | - | - | NORMAL | - | NEG. |
|  |  | 20024 | 1.014 | - | - | - | NORMAL | - | NEG. |
|  |  | 20025 | 1.016 | - | - | - | NORMAL | - | NEG. |
|  |  | 20026 | 1.009 | - | - | - | NORMAL | - | NEG. |
|  |  | 20027 | 1.007 | - | - | - | NORMAL | - | NEG. |
|  |  | 20028 | 1.007 | - | - | - | NORMAL | - | NEG. |
|  |  | 20029 | 1.015 | +2 | +- | - | NORMAL | - | NEG. |
|  |  | 20030 | 1.011 | - | - | - | NORMAL | - | NEG. |
|  | 0.5 | 21061 | 1.007 | +3 | +1 | - | NORMAL | - | NEG. |
|  |  | 21062 | 1.015 | +- | - | - | NORMAL | - | NEG. |
|  |  | 21063 | 1.003 | +3 | +- | - | NORMAL | - | 25 |
|  |  | 21064 | 1.010 | - | - | - | NORMAL | - | NEG. |
|  |  | 21065 | 1.015 | - | +- | - | NORMAL | - | NEG. |
|  |  | 21066 | 1.006 | - | - | - | NORMAL | - | NEG. |
|  |  | 21067 | 1.016 | - | +- | - | NORMAL | - | 25 |
|  |  | 21068 | 1.006 | - | - | - | NORMAL | - | NEG. |
|  |  | 21069 | 1.006 | - | - | - | NORMAL | - | NEG. |
|  |  | 21070 | 1.007 | - | - | - | NORMAL | - | NEG. |
|  | 1.5 | 22101 | 1.013 | - | +- | - | NORMAL | - | NEG. |
|  |  | 22102 | 1.004 | - | - | - | NORMAL | - | NEG. |
|  |  | 22103 | 1.011 | +3 | +1 | - | NORMAL | - | 25 |
|  |  | 22104 | 1.009 | - | - | - | NORMAL | - | NEG. |
|  |  | 22105 | 1.006 | - | - | - | NORMAL | - | NEG. |
|  |  | 22106 | 1.005 | +3 | - | - | NORMAL | - | NEG. |
|  |  | 22107 | 1.011 | - | +- | - | NORMAL | - | NEG. |
|  |  | 22108 | 1.005 | - | - | - | NORMAL | - | NEG. |
|  |  | 22109 | 1.015 | - | - | - | NORMAL | - | NEG. |
|  |  | 22110 | 1.017 | +2 | +- | - | NORMAL | - | NEG. |
|  | 5 | 23141 | 1.028 | +- | +- | - | NORMAL | - | NEG. |
|  |  | 23142 | 1.027 | - | +- | - | NORMAL | - | NEG. |
|  |  | 23143 | 1.031 | - | +- | - | NORMAL | - | NEG. |
|  |  | 23144 | 1.036 | - | +- | - | NORMAL | - | 25 |
|  |  | 23145 | 1.024 | - | - | - | NORMAL | - | NEG. |
|  |  | 23146 | 1.012 | - | - | - | NORMAL | - | NEG. |
|  |  | 23147 | 1.017 | +1 | +- | - | NORMAL | - | NEG. |
|  |  | 23148 | 1.030 | - | +1 | - | NORMAL | - | NEG. |
|  |  | 23149 | 1.042 | - | +1 | - | NORMAL | - | NEG. |
|  |  | 23150 | 1.031 | - | +- | - | NORMAL | - | NEG. |

### Urine data of individual animal

| **Urine data on recovery day 27** | | | | | | | |
| --- | --- | --- | --- | --- | --- | --- | --- |
| **Gender** | **Dose** | **Animal no.** | **COL** | **TURB** | **pH** | **NIT** | **GLU** |
|  | **g/kg** |  |  |  |  |  |  |
| Male | 0 | 10011 | COLORLESS | +1 | 8.0 | - | NORMAL |
|  |  | 10012 | COLORLESS | +1 | 8.0 | - | NORMAL |
|  |  | 10013 | COLORLESS | +1 | 8.5 | - | NORMAL |
|  |  | 10014 | COLORLESS | - | 8.0 | - | NORMAL |
|  |  | 10015 | LIGHT RED | +1 | 8.0 | - | NORMAL |
|  | 0.5 | 11051 | COLORLESS | +1 | 8.5 | - | NORMAL |
|  |  | 11052 | LIGHT RED | +1 | 8.0 | - | NORMAL |
|  |  | 11053 | COLORLESS | - | 8.0 | - | NORMAL |
|  |  | 11054 | COLORLESS | +1 | 8.0 | - | NORMAL |
|  |  | 11055 | COLORLESS | +1 | 8.0 | - | NORMAL |
|  | 1.5 | 12091 | COLORLESS | - | 8.0 | - | NORMAL |
|  |  | 12092 | COLORLESS | - | 8.5 | - | NORMAL |
|  |  | 12093 | COLORLESS | +1 | 7.0 | - | NORMAL |
|  |  | 12094 | COLORLESS | +1 | 8.5 | - | NORMAL |
|  |  | 12095 | COLORLESS | +1 | 8.5 | +1 | NORMAL |
|  | 5 | 13131 | COLORLESS | +1 | 8.5 | - | NORMAL |
|  |  | 13132 | COLORLESS | +1 | 8.0 | - | NORMAL |
|  |  | 13133 | COLORLESS | - | 8.0 | - | NORMAL |
|  |  | 13134 | COLORLESS | - | 8.0 | - | NORMAL |
|  |  | 13135 | COLORLESS | - | 7.5 | - | NORMAL |
| Female | 0 | 20031 | COLORLESS | +1 | 8.0 | +1 | NORMAL |
|  |  | 20032 | COLORLESS | - | 8.0 | - | NORMAL |
|  |  | 20033 | COLORLESS | +1 | 7.0 | +1 | NORMAL |
|  |  | 20034 | LIGHT YELLOW | +1 | 8.0 | - | NORMAL |
|  |  | 20035 | COLORLESS | - | 7.0 | - | NORMAL |
|  | 0.5 | 21071 | COLORLESS | - | 8.0 | +1 | NORMAL |
|  |  | 21072 | COLORLESS | - | 8.0 | +1 | NORMAL |
|  |  | 21073 | COLORLESS | - | 8.0 | +1 | NORMAL |
|  |  | 21074 | COLORLESS | - | 7.5 | - | NORMAL |
|  |  | 21075 | COLORLESS | - | 7.5 | +1 | NORMAL |
|  | 1.5 | 22111 | COLORLESS | - | 8.5 | +2 | NORMAL |
|  |  | 22112 | COLORLESS | +1 | 8.5 | +1 | NORMAL |
|  |  | 22113 | COLORLESS | +1 | 8.5 | +1 | NORMAL |
|  |  | 22114 | COLORLESS | - | 7.0 | - | NORMAL |
|  |  | 22115 | COLORLESS | +1 | 8.0 | - | NORMAL |
|  | 5 | 23151 | COLORLESS | - | 8.0 | - | NORMAL |
|  |  | 23152 | COLORLESS | +1 | 8.5 | - | NORMAL |
|  |  | 23153 | COLORLESS | - | 8.5 | +1 | NORMAL |
|  |  | 23154 | COLORLESS | +1 | 8.0 | - | NORMAL |
|  |  | 23155 | LIGHT YELLOW | +1 | 8.5 | +1 | NORMAL |

### Urine data of individual animal

| **Urine data on recovery day 27** | | | | | | | | | |
| --- | --- | --- | --- | --- | --- | --- | --- | --- | --- |
| **Gender** | **Dose** | **Animal no.** | **SG** | **BLD** | **PRO** | **BIL** | **URO** | **KET** | **LEU** |
|  | **g/kg** |  |  |  |  |  |  |  | **Leu/uL** |
| Male | 0 | 10011 | 1.011 | - | +- | - | NORMAL | - | 75 |
|  |  | 10012 | 1.014 | - | +1 | - | NORMAL | - | 250 |
|  |  | 10013 | 1.025 | - | +1 | - | NORMAL | - | 25 |
|  |  | 10014 | 1.013 | +2 | +1 | - | NORMAL | - | 500 |
|  |  | 10015 | 1.010 | - | +1 | - | NORMAL | - | 250 |
|  | 0.5 | 11051 | 1.011 | - | +- | - | NORMAL | - | NEG. |
|  |  | 11052 | 1.016 | - | +1 | - | NORMAL | - | 25 |
|  |  | 11053 | 1.008 | - | - | - | NORMAL | - | NEG. |
|  |  | 11054 | 1.010 | - | +1 | - | NORMAL | - | 250 |
|  |  | 11055 | 1.023 | - | +1 | - | NORMAL | - | 75 |
|  | 1.5 | 12091 | 1.014 | - | +- | - | NORMAL | - | NEG. |
|  |  | 12092 | 1.020 | - | +1 | - | NORMAL | - | 75 |
|  |  | 12093 | 1.018 | +1 | +- | - | NORMAL | - | 25 |
|  |  | 12094 | 1.020 | - | +1 | - | NORMAL | - | 75 |
|  |  | 12095 | 1.007 | - | - | - | NORMAL | - | NEG. |
|  | 5 | 13131 | 1.021 | - | +2 | - | NORMAL | - | 500 |
|  |  | 13132 | 1.011 | +1 | +1 | - | NORMAL | - | 75 |
|  |  | 13133 | 1.013 | - | +- | - | NORMAL | - | 25 |
|  |  | 13134 | 1.010 | - | +- | - | NORMAL | - | NEG. |
|  |  | 13135 | 1.005 | - | +- | - | NORMAL | - | NEG. |
| Female | 0 | 20031 | 1.008 | +- | +- | - | NORMAL | - | NEG. |
|  |  | 20032 | 1.013 | - | - | - | NORMAL | - | NEG. |
|  |  | 20033 | 1.005 | - | - | - | NORMAL | - | NEG. |
|  |  | 20034 | 1.013 | - | - | - | NORMAL | - | NEG. |
|  |  | 20035 | 1.003 | +- | - | - | NORMAL | - | NEG. |
|  | 0.5 | 21071 | 1.004 | - | - | - | NORMAL | - | NEG. |
|  |  | 21072 | 1.003 | - | - | - | NORMAL | - | NEG. |
|  |  | 21073 | 1.005 | - | - | - | NORMAL | - | NEG. |
|  |  | 21074 | 1.004 | - | - | - | NORMAL | - | NEG. |
|  |  | 21075 | 1.003 | +2 | - | - | NORMAL | - | NEG. |
|  | 1.5 | 22111 | 1.008 | - | - | - | NORMAL | - | NEG. |
|  |  | 22112 | 1.007 | - | - | - | NORMAL | - | NEG. |
|  |  | 22113 | 1.007 | - | - | - | NORMAL | - | NEG. |
|  |  | 22114 | 1.004 | +1 | - | - | NORMAL | - | NEG. |
|  |  | 22115 | 1.006 | - | - | - | NORMAL | - | NEG. |
|  | 5 | 23151 | 1.008 | - | - | - | NORMAL | - | NEG. |
|  |  | 23152 | 1.007 | - | - | - | NORMAL | - | NEG. |
|  |  | 23153 | 1.009 | - | - | - | NORMAL | - | NEG. |
|  |  | 23154 | 1.006 | - | - | - | NORMAL | - | NEG. |
|  |  | 23155 | 1.006 | - | - | - | NORMAL | - | NEG. |

### Organ data of individual animal

| **Organ data of animals dissected on day 92** | | | | | | | | |
| --- | --- | --- | --- | --- | --- | --- | --- | --- |
| **Gender** | **Dose** | **Animal no.** | **Heart** | | | **Liver** | | |
|  | **g/kg** |  | **Weight** | **Organ-to-body ratio** | **Organ-to-brain ratio** | **Weight** | **Organ-to-body ratio** | **Organ-to-brain ratio** |
| Male | 0 | 10001 | 1.760 | 0.276 | 0.693 | 16.133 | 2.533 | 6.349 |
|  |  | 10002 | 1.797 | 0.296 | 0.793 | 17.445 | 2.873 | 7.699 |
|  |  | 10003 | 1.412 | 0.279 | 0.652 | 11.438 | 2.261 | 5.283 |
|  |  | 10004 | 1.937 | 0.297 | 0.887 | 20.419 | 3.131 | 9.349 |
|  |  | 10005 | 1.624 | 0.314 | 0.749 | 14.472 | 2.794 | 6.672 |
|  |  | 10006 | 1.798 | 0.273 | 0.790 | 20.280 | 3.079 | 8.906 |
|  |  | 10007 | 1.802 | 0.285 | 0.831 | 19.488 | 3.083 | 8.985 |
|  |  | 10008 | 1.609 | 0.270 | 0.788 | 16.366 | 2.743 | 8.019 |
|  |  | 10009 | 1.714 | 0.293 | 0.820 | 15.225 | 2.602 | 7.285 |
|  |  | 10010 | 1.500 | 0.283 | 0.701 | 12.685 | 2.397 | 5.930 |
|  | 0.5 | 11041 | 1.733 | 0.322 | 0.783 | 14.228 | 2.640 | 6.432 |
|  |  | 11042 | 1.919 | 0.317 | 0.885 | 17.930 | 2.962 | 8.266 |
|  |  | 11043 | 1.551 | 0.282 | 0.723 | 14.660 | 2.664 | 6.834 |
|  |  | 11044 | 1.661 | 0.320 | 0.741 | 14.633 | 2.818 | 6.524 |
|  |  | 11045 | 1.923 | 0.300 | 0.840 | 21.766 | 3.397 | 9.513 |
|  |  | 11046 | 1.830 | 0.321 | 0.825 | 14.460 | 2.533 | 6.516 |
|  |  | 11047 | 1.655 | 0.296 | 0.717 | 14.773 | 2.639 | 6.401 |
|  |  | 11048 | 1.550 | 0.316 | 0.723 | 12.599 | 2.569 | 5.879 |
|  |  | 11049 | 1.597 | 0.261 | 0.757 | 18.428 | 3.006 | 8.738 |
|  |  | 11050 | 1.980 | 0.296 | 0.924 | 20.657 | 3.086 | 9.644 |
|  | 1.5 | 12081 | 1.764 | 0.297 | 0.844 | 15.606 | 2.626 | 7.471 |
|  |  | 12082 | 1.754 | 0.291 | 0.831 | 17.272 | 2.863 | 8.186 |
|  |  | 12083 | 1.638 | 0.295 | 0.727 | 13.906 | 2.508 | 6.169 |
|  |  | 12084 | 1.722 | 0.299 | 0.763 | 15.929 | 2.766 | 7.061 |
|  |  | 12085 | 1.446 | 0.252 | 0.652 | 13.728 | 2.397 | 6.192 |
|  |  | 12086 | 1.396 | 0.290 | 0.649 | 12.226 | 2.540 | 5.681 |
|  |  | 12087 | 1.596 | 0.256 | 0.709 | 16.143 | 2.593 | 7.171 |
|  |  | 12088 | 1.487 | 0.311 | 0.681 | 12.131 | 2.537 | 5.557 |
|  |  | 12089 | 1.538 | 0.295 | 0.715 | 13.361 | 2.561 | 6.209 |
|  |  | 12090 | 1.837 | 0.284 | 0.839 | 18.732 | 2.894 | 8.553 |
|  | 5 | 13121 | 1.472 | 0.281 | 0.693 | 15.346 | 2.925 | 7.225 |
|  |  | 13122 | 1.609 | 0.295 | 0.724 | 17.925 | 3.289 | 8.071 |
|  |  | 13123 | 1.564 | 0.271 | 0.733 | 14.930 | 2.585 | 6.993 |
|  |  | 13124 | 1.685 | 0.312 | 0.743 | 15.635 | 2.896 | 6.894 |
|  |  | 13125 | 1.676 | 0.321 | 0.711 | 15.036 | 2.876 | 6.382 |
|  |  | 13126 | 1.876 | 0.327 | 0.835 | 16.962 | 2.956 | 7.549 |
|  |  | 13127 | 1.626 | 0.288 | 0.743 | 15.558 | 2.754 | 7.114 |
|  |  | 13128 | 1.763 | 0.292 | 0.792 | 16.720 | 2.774 | 7.515 |
|  |  | 13129 | 1.931 | 0.284 | 0.917 | 18.937 | 2.787 | 8.992 |
|  |  | 13130 | 1.580 | 0.284 | 0.704 | 14.811 | 2.664 | 6.603 |

Note: Weight (Unit:g); organ to body ratio (g / 100 g body weight); organ to brain ratio (g / g brain weight)

### Organ data of individual animal

| **Organ data of animals dissected on day 92** | | | | | | | | |
| --- | --- | --- | --- | --- | --- | --- | --- | --- |
| **Gender** | **Dose** | **Animal no.** | **Spleen** | | | **Kidneys** | | |
|  | **g/kg** |  | **Weight** | **Organ-to-body ratio** | **Organ-to-brain ratio** | **Weight** | **Organ-to-body ratio** | **Organ-to-brain ratio** |
| Male | 0 | 10001 | 0.855 | 0.134 | 0.336 | 3.864 | 0.607 | 1.521 |
|  |  | 10002 | 0.958 | 0.158 | 0.423 | 3.957 | 0.652 | 1.746 |
|  |  | 10003 | 0.816 | 0.161 | 0.377 | 2.916 | 0.576 | 1.347 |
|  |  | 10004 | 0.986 | 0.151 | 0.451 | 4.334 | 0.665 | 1.984 |
|  |  | 10005 | 0.721 | 0.139 | 0.332 | 3.316 | 0.640 | 1.529 |
|  |  | 10006 | 1.078 | 0.164 | 0.473 | 4.140 | 0.629 | 1.818 |
|  |  | 10007 | 1.047 | 0.166 | 0.483 | 3.805 | 0.602 | 1.754 |
|  |  | 10008 | 0.942 | 0.158 | 0.462 | 3.839 | 0.643 | 1.881 |
|  |  | 10009 | 0.945 | 0.162 | 0.452 | 3.669 | 0.627 | 1.756 |
|  |  | 10010 | 0.760 | 0.144 | 0.355 | 2.977 | 0.562 | 1.392 |
|  | 0.5 | 11041 | 1.067 | 0.198 | 0.482 | 3.529 | 0.655 | 1.595 |
|  |  | 11042 | 0.969 | 0.160 | 0.447 | 4.269 | 0.705 | 1.968 |
|  |  | 11043 | 0.844 | 0.153 | 0.393 | 3.033 | 0.551 | 1.414 |
|  |  | 11044 | 0.803 | 0.155 | 0.358 | 3.820 | 0.736 | 1.703 |
|  |  | 11045 | 1.104 | 0.172 | 0.483 | 3.824 | 0.597 | 1.671 |
|  |  | 11046 | 1.018 | 0.178 | 0.459 | 3.907 | 0.684 | 1.761 |
|  |  | 11047 | 0.913 | 0.163 | 0.396 | 3.492 | 0.624 | 1.513 |
|  |  | 11048 | 0.787 | 0.160 | 0.367 | 3.040 | 0.620 | 1.419 |
|  |  | 11049 | 0.971 | 0.158 | 0.460 | 3.614 | 0.590 | 1.714 |
|  |  | 11050 | 1.083 | 0.162 | 0.506 | 4.430 | 0.662 | 2.068 |
|  | 1.5 | 12081 | 0.748 | 0.126 | 0.358 | 3.868 | 0.651 | 1.852 |
|  |  | 12082 | 0.849 | 0.141 | 0.402 | 3.567 | 0.591 | 1.691 |
|  |  | 12083 | 0.828 | 0.149 | 0.367 | 3.535 | 0.638 | 1.568 |
|  |  | 12084 | 1.216 | 0.211 | 0.539 | 3.689 | 0.641 | 1.635 |
|  |  | 12085 | 0.868 | 0.152 | 0.392 | 3.233 | 0.565 | 1.458 |
|  |  | 12086 | 0.937 | 0.195 | 0.435 | 2.771 | 0.576 | 1.288 |
|  |  | 12087 | 0.731 | 0.117 | 0.325 | 3.930 | 0.631 | 1.746 |
|  |  | 12088 | 0.618 | 0.129 | 0.283 | 3.106 | 0.650 | 1.423 |
|  |  | 12089 | 0.715 | 0.137 | 0.332 | 3.068 | 0.588 | 1.426 |
|  |  | 12090 | 1.149 | 0.178 | 0.525 | 4.286 | 0.662 | 1.957 |
|  | 5 | 13121 | 0.721 | 0.137 | 0.339 | 3.766 | 0.718 | 1.773 |
|  |  | 13122 | 0.840 | 0.154 | 0.378 | 4.090 | 0.750 | 1.842 |
|  |  | 13123 | 1.045 | 0.181 | 0.489 | 3.850 | 0.667 | 1.803 |
|  |  | 13124 | 0.785 | 0.145 | 0.346 | 4.207 | 0.779 | 1.855 |
|  |  | 13125 | 0.751 | 0.144 | 0.319 | 3.620 | 0.692 | 1.537 |
|  |  | 13126 | 1.064 | 0.185 | 0.474 | 4.197 | 0.731 | 1.868 |
|  |  | 13127 | 0.881 | 0.156 | 0.403 | 3.721 | 0.659 | 1.701 |
|  |  | 13128 | 1.029 | 0.171 | 0.462 | 4.272 | 0.709 | 1.920 |
|  |  | 13129 | 0.864 | 0.127 | 0.410 | 4.382 | 0.645 | 2.081 |
|  |  | 13130 | 0.885 | 0.159 | 0.395 | 3.730 | 0.671 | 1.663 |

Note: Weight (Unit:g); organ to body ratio (g / 100 g body weight); organ to brain ratio (g / g brain weight)

### Organ data of individual animal

| **Organ data of animals dissected on day 92** | | | | | | | | |
| --- | --- | --- | --- | --- | --- | --- | --- | --- |
| **Gender** | **Dose** | **Animal no.** | **Thymus** | | | **Adrenal glands** | | |
|  | **g/kg** |  | **Weight** | **Organ-to-body ratio** | **Organ-to-brain ratio** | **Weight** | **Organ-to-body ratio** | **Organ-to-brain ratio** |
| Male | 0 | 10001 | 0.318 | 0.050 | 0.125 | 0.068 | 0.011 | 0.027 |
|  |  | 10002 | 0.293 | 0.048 | 0.129 | 0.073 | 0.012 | 0.032 |
|  |  | 10003 | 0.194 | 0.038 | 0.090 | 0.055 | 0.011 | 0.025 |
|  |  | 10004 | 0.250 | 0.038 | 0.114 | 0.082 | 0.013 | 0.038 |
|  |  | 10005 | 0.362 | 0.070 | 0.167 | 0.070 | 0.014 | 0.032 |
|  |  | 10006 | 0.198 | 0.030 | 0.087 | 0.076 | 0.012 | 0.033 |
|  |  | 10007 | 0.250 | 0.040 | 0.115 | 0.080 | 0.013 | 0.037 |
|  |  | 10008 | 0.269 | 0.045 | 0.132 | 0.067 | 0.011 | 0.033 |
|  |  | 10009 | 0.244 | 0.042 | 0.117 | 0.076 | 0.013 | 0.036 |
|  |  | 10010 | 0.250 | 0.047 | 0.117 | 0.068 | 0.013 | 0.032 |
|  | 0.5 | 11041 | 0.289 | 0.054 | 0.131 | 0.082 | 0.015 | 0.037 |
|  |  | 11042 | 0.321 | 0.053 | 0.148 | 0.077 | 0.013 | 0.036 |
|  |  | 11043 | 0.210 | 0.038 | 0.098 | 0.060 | 0.011 | 0.028 |
|  |  | 11044 | 0.292 | 0.056 | 0.130 | 0.061 | 0.012 | 0.027 |
|  |  | 11045 | 0.379 | 0.059 | 0.166 | 0.067 | 0.010 | 0.029 |
|  |  | 11046 | 0.243 | 0.043 | 0.110 | 0.080 | 0.014 | 0.036 |
|  |  | 11047 | 0.265 | 0.047 | 0.115 | 0.059 | 0.011 | 0.026 |
|  |  | 11048 | 0.238 | 0.049 | 0.111 | 0.073 | 0.015 | 0.034 |
|  |  | 11049 | 0.267 | 0.044 | 0.127 | 0.091 | 0.015 | 0.043 |
|  |  | 11050 | 0.338 | 0.050 | 0.158 | 0.093 | 0.014 | 0.043 |
|  | 1.5 | 12081 | 0.313 | 0.053 | 0.150 | 0.060 | 0.010 | 0.029 |
|  |  | 12082 | 0.164 | 0.027 | 0.078 | 0.083 | 0.014 | 0.039 |
|  |  | 12083 | 0.247 | 0.045 | 0.110 | 0.073 | 0.013 | 0.032 |
|  |  | 12084 | 0.294 | 0.051 | 0.130 | 0.069 | 0.012 | 0.031 |
|  |  | 12085 | 0.206 | 0.036 | 0.093 | 0.078 | 0.014 | 0.035 |
|  |  | 12086 | 0.263 | 0.055 | 0.122 | 0.048 | 0.010 | 0.022 |
|  |  | 12087 | 0.274 | 0.044 | 0.122 | 0.073 | 0.012 | 0.032 |
|  |  | 12088 | 0.208 | 0.044 | 0.095 | 0.080 | 0.017 | 0.037 |
|  |  | 12089 | 0.208 | 0.040 | 0.097 | 0.082 | 0.016 | 0.038 |
|  |  | 12090 | 0.326 | 0.050 | 0.149 | 0.093 | 0.014 | 0.042 |
|  | 5 | 13121 | 0.257 | 0.049 | 0.121 | 0.086 | 0.016 | 0.040 |
|  |  | 13122 | 0.334 | 0.061 | 0.150 | 0.067 | 0.012 | 0.030 |
|  |  | 13123 | 0.248 | 0.043 | 0.116 | 0.063 | 0.011 | 0.030 |
|  |  | 13124 | 0.301 | 0.056 | 0.133 | 0.094 | 0.017 | 0.041 |
|  |  | 13125 | 0.311 | 0.059 | 0.132 | 0.076 | 0.015 | 0.032 |
|  |  | 13126 | 0.249 | 0.043 | 0.111 | 0.071 | 0.012 | 0.032 |
|  |  | 13127 | 0.311 | 0.055 | 0.142 | 0.063 | 0.011 | 0.029 |
|  |  | 13128 | 0.297 | 0.049 | 0.133 | 0.063 | 0.010 | 0.028 |
|  |  | 13129 | 0.296 | 0.044 | 0.141 | 0.072 | 0.011 | 0.034 |
|  |  | 13130 | 0.328 | 0.059 | 0.146 | 0.067 | 0.012 | 0.030 |

Note: Weight (Unit:g); organ to body ratio (g / 100 g body weight); organ to brain ratio (g / g brain weight)

### Organ data of individual animal

| **Organ data of animals dissected on day 92** | | | | | | | | |
| --- | --- | --- | --- | --- | --- | --- | --- | --- |
| **Gender** | **Dose** | **Animal no.** | **Testes** | | | **Epididymides** | | |
|  | **g/kg** |  | **Weight** | **Organ-to-body ratio** | **Organ-to-brain ratio** | **Weight** | **Organ-to-body ratio** | **Organ-to-brain ratio** |
| Male | 0 | 10001 | 4.308 | 0.676 | 1.695 | 1.764 | 0.277 | 0.694 |
|  |  | 10002 | 3.671 | 0.605 | 1.620 | 1.419 | 0.234 | 0.626 |
|  |  | 10003 | 3.266 | 0.646 | 1.509 | 1.334 | 0.264 | 0.616 |
|  |  | 10004 | 3.762 | 0.577 | 1.723 | 1.632 | 0.250 | 0.747 |
|  |  | 10005 | 3.651 | 0.705 | 1.683 | 1.298 | 0.251 | 0.598 |
|  |  | 10006 | 4.414 | 0.670 | 1.939 | 1.800 | 0.273 | 0.791 |
|  |  | 10007 | 3.291 | 0.521 | 1.517 | 1.332 | 0.211 | 0.614 |
|  |  | 10008 | 3.273 | 0.549 | 1.604 | 1.273 | 0.213 | 0.624 |
|  |  | 10009 | 3.289 | 0.562 | 1.574 | 1.215 | 0.208 | 0.581 |
|  |  | 10010 | 3.509 | 0.663 | 1.640 | 1.413 | 0.267 | 0.661 |
|  | 0.5 | 11041 | 3.290 | 0.611 | 1.487 | 1.436 | 0.266 | 0.649 |
|  |  | 11042 | 4.165 | 0.688 | 1.920 | 1.679 | 0.277 | 0.774 |
|  |  | 11043 | 3.647 | 0.663 | 1.700 | 1.349 | 0.245 | 0.629 |
|  |  | 11044 | 3.846 | 0.741 | 1.715 | 1.485 | 0.286 | 0.662 |
|  |  | 11045 | 3.286 | 0.513 | 1.436 | 1.451 | 0.226 | 0.634 |
|  |  | 11046 | 3.868 | 0.678 | 1.743 | 1.470 | 0.258 | 0.662 |
|  |  | 11047 | 3.345 | 0.598 | 1.449 | 1.600 | 0.286 | 0.693 |
|  |  | 11048 | 3.384 | 0.690 | 1.579 | 1.508 | 0.307 | 0.704 |
|  |  | 11049 | 3.344 | 0.546 | 1.586 | 1.282 | 0.209 | 0.608 |
|  |  | 11050 | 3.903 | 0.583 | 1.822 | 1.564 | 0.234 | 0.730 |
|  | 1.5 | 12081 | 3.169 | 0.533 | 1.517 | 1.557 | 0.262 | 0.745 |
|  |  | 12082 | 3.332 | 0.552 | 1.579 | 1.248 | 0.207 | 0.591 |
|  |  | 12083 | 3.349 | 0.604 | 1.486 | 1.440 | 0.260 | 0.639 |
|  |  | 12084 | 3.618 | 0.628 | 1.604 | 1.458 | 0.253 | 0.646 |
|  |  | 12085 | 3.907 | 0.682 | 1.762 | 1.509 | 0.263 | 0.681 |
|  |  | 12086 | 3.241 | 0.673 | 1.506 | 1.425 | 0.296 | 0.662 |
|  |  | 12087 | 3.676 | 0.591 | 1.633 | 1.596 | 0.256 | 0.709 |
|  |  | 12088 | 3.207 | 0.671 | 1.469 | 1.226 | 0.256 | 0.562 |
|  |  | 12089 | 3.370 | 0.646 | 1.566 | 1.448 | 0.278 | 0.673 |
|  |  | 12090 | 3.981 | 0.615 | 1.818 | 1.603 | 0.248 | 0.732 |
|  | 5 | 13121 | 3.320 | 0.633 | 1.563 | 1.534 | 0.292 | 0.722 |
|  |  | 13122 | 3.054 | 0.560 | 1.375 | 1.447 | 0.266 | 0.652 |
|  |  | 13123 | 3.132 | 0.542 | 1.467 | 1.267 | 0.219 | 0.593 |
|  |  | 13124 | 3.752 | 0.695 | 1.654 | 1.464 | 0.271 | 0.646 |
|  |  | 13125 | 3.184 | 0.609 | 1.351 | 1.359 | 0.260 | 0.577 |
|  |  | 13126 | 3.520 | 0.613 | 1.567 | 1.378 | 0.240 | 0.613 |
|  |  | 13127 | 3.865 | 0.684 | 1.767 | 1.463 | 0.259 | 0.669 |
|  |  | 13128 | 3.757 | 0.623 | 1.689 | 1.533 | 0.254 | 0.689 |
|  |  | 13129 | 3.879 | 0.571 | 1.842 | 1.552 | 0.228 | 0.737 |
|  |  | 13130 | 3.581 | 0.644 | 1.597 | 1.481 | 0.266 | 0.660 |

Note: Weight (Unit:g); organ to body ratio (g / 100 g body weight); organ to brain ratio (g / g brain weight)

### Organ data of individual animal

| **Organ data of animals dissected on day 92** | | | | | |
| --- | --- | --- | --- | --- | --- |
| **Gender** | **Dose** | **Animal no.** | **Brain** | | **Body weight** |
|  | **g/kg** |  | **Weight** | **Organ-to-body ratio** | **g** |
| Male | 0 | 10001 | 2.541 | 0.399 | 637.0 |
|  |  | 10002 | 2.266 | 0.373 | 607.2 |
|  |  | 10003 | 2.165 | 0.428 | 505.9 |
|  |  | 10004 | 2.184 | 0.335 | 652.1 |
|  |  | 10005 | 2.169 | 0.419 | 518.0 |
|  |  | 10006 | 2.277 | 0.346 | 658.6 |
|  |  | 10007 | 2.169 | 0.343 | 632.2 |
|  |  | 10008 | 2.041 | 0.342 | 596.6 |
|  |  | 10009 | 2.090 | 0.357 | 585.1 |
|  |  | 10010 | 2.139 | 0.404 | 529.3 |
|  | 0.5 | 11041 | 2.212 | 0.410 | 538.9 |
|  |  | 11042 | 2.169 | 0.358 | 605.3 |
|  |  | 11043 | 2.145 | 0.390 | 550.4 |
|  |  | 11044 | 2.243 | 0.432 | 519.3 |
|  |  | 11045 | 2.288 | 0.357 | 640.8 |
|  |  | 11046 | 2.219 | 0.389 | 570.8 |
|  |  | 11047 | 2.308 | 0.412 | 559.8 |
|  |  | 11048 | 2.143 | 0.437 | 490.5 |
|  |  | 11049 | 2.109 | 0.344 | 613.0 |
|  |  | 11050 | 2.142 | 0.320 | 669.4 |
|  | 1.5 | 12081 | 2.089 | 0.351 | 594.4 |
|  |  | 12082 | 2.110 | 0.350 | 603.2 |
|  |  | 12083 | 2.254 | 0.407 | 554.4 |
|  |  | 12084 | 2.256 | 0.392 | 575.8 |
|  |  | 12085 | 2.217 | 0.387 | 572.7 |
|  |  | 12086 | 2.152 | 0.447 | 481.4 |
|  |  | 12087 | 2.251 | 0.362 | 622.5 |
|  |  | 12088 | 2.183 | 0.457 | 478.1 |
|  |  | 12089 | 2.152 | 0.412 | 521.8 |
|  |  | 12090 | 2.190 | 0.338 | 647.2 |
|  | 5 | 13121 | 2.124 | 0.405 | 524.7 |
|  |  | 13122 | 2.221 | 0.408 | 545.0 |
|  |  | 13123 | 2.135 | 0.370 | 577.6 |
|  |  | 13124 | 2.268 | 0.420 | 539.9 |
|  |  | 13125 | 2.356 | 0.451 | 522.9 |
|  |  | 13126 | 2.247 | 0.392 | 573.8 |
|  |  | 13127 | 2.187 | 0.387 | 565.0 |
|  |  | 13128 | 2.225 | 0.369 | 602.8 |
|  |  | 13129 | 2.106 | 0.310 | 679.5 |
|  |  | 13130 | 2.243 | 0.403 | 556.0 |

Note: Weight (Unit:g); organ to body ratio (g / 100 g body weight); organ to brain ratio (g / g brain weight)

### Organ data of individual animal

| **Organ data of animals dissected on day 92** | | | | | | | | |
| --- | --- | --- | --- | --- | --- | --- | --- | --- |
| **Gender** | **Dose** | **Animal no.** | **Heart** | | | **Liver** | | |
|  | **g/kg** |  | **Weight** | **Organ-to-body ratio** | **Organ-to-brain ratio** | **Weight** | **Organ-to-body ratio** | **Organ-to-brain ratio** |
| Female | 0 | 20021 | 0.889 | 0.335 | 0.443 | 6.877 | 2.591 | 3.423 |
|  |  | 20022 | 0.914 | 0.302 | 0.450 | 7.586 | 2.510 | 3.737 |
|  |  | 20023 | 1.057 | 0.319 | 0.529 | 7.831 | 2.366 | 3.916 |
|  |  | 20024 | 1.087 | 0.349 | 0.569 | 8.281 | 2.661 | 4.331 |
|  |  | 20025 | 1.020 | 0.348 | 0.495 | 7.867 | 2.687 | 3.815 |
|  |  | 20026 | 1.026 | 0.365 | 0.494 | 8.011 | 2.848 | 3.861 |
|  |  | 20027 | 0.993 | 0.313 | 0.490 | 7.841 | 2.475 | 3.866 |
|  |  | 20028 | 0.908 | 0.333 | 0.437 | 7.434 | 2.725 | 3.574 |
|  |  | 20029 | 0.985 | 0.347 | 0.514 | 8.106 | 2.858 | 4.228 |
|  |  | 20030 | 1.051 | 0.314 | 0.589 | 8.104 | 2.419 | 4.543 |
|  | 0.5 | 21061 | 0.900 | 0.352 | 0.489 | 6.390 | 2.498 | 3.469 |
|  |  | 21062 | 0.908 | 0.336 | 0.459 | 7.197 | 2.660 | 3.635 |
|  |  | 21063 | 0.876 | 0.335 | 0.405 | 6.278 | 2.404 | 2.905 |
|  |  | 21064 | 0.933 | 0.331 | 0.478 | 8.107 | 2.875 | 4.155 |
|  |  | 21065 | 0.967 | 0.310 | 0.511 | 8.338 | 2.671 | 4.407 |
|  |  | 21066 | 0.990 | 0.351 | 0.537 | 7.217 | 2.558 | 3.918 |
|  |  | 21067 | 0.960 | 0.296 | 0.461 | 9.515 | 2.935 | 4.566 |
|  |  | 21068 | 0.939 | 0.341 | 0.460 | 7.438 | 2.702 | 3.641 |
|  |  | 21069 | 1.151 | 0.389 | 0.522 | 9.100 | 3.077 | 4.125 |
|  |  | 21070 | 0.828 | 0.313 | 0.406 | 6.659 | 2.517 | 3.267 |
|  | 1.5 | 22101 | 0.917 | 0.352 | 0.471 | 6.749 | 2.589 | 3.468 |
|  |  | 22102 | 0.965 | 0.351 | 0.490 | 7.181 | 2.614 | 3.643 |
|  |  | 22103 | 1.029 | 0.358 | 0.534 | 7.457 | 2.597 | 3.868 |
|  |  | 22104 | 0.879 | 0.341 | 0.397 | 6.439 | 2.501 | 2.906 |
|  |  | 22105 | 0.872 | 0.346 | 0.442 | 7.268 | 2.888 | 3.680 |
|  |  | 22106 | 0.998 | 0.315 | 0.493 | 8.697 | 2.744 | 4.299 |
|  |  | 22107 | 0.870 | 0.290 | 0.425 | 7.852 | 2.614 | 3.834 |
|  |  | 22108 | 1.094 | 0.371 | 0.540 | 7.814 | 2.649 | 3.859 |
|  |  | 22109 | 1.119 | 0.370 | 0.553 | 8.271 | 2.738 | 4.086 |
|  |  | 22110 | 1.006 | 0.328 | 0.512 | 8.869 | 2.889 | 4.511 |
|  | 5 | 23141 | 0.917 | 0.333 | 0.456 | 7.388 | 2.684 | 3.676 |
|  |  | 23142 | 0.800 | 0.314 | 0.430 | 6.480 | 2.546 | 3.486 |
|  |  | 23143 | 0.931 | 0.332 | 0.483 | 8.050 | 2.873 | 4.177 |
|  |  | 23144 | 1.036 | 0.310 | 0.509 | 8.518 | 2.549 | 4.182 |
|  |  | 23145 | 1.052 | 0.335 | 0.554 | 9.797 | 3.118 | 5.162 |
|  |  | 23146 | 0.960 | 0.318 | 0.492 | 8.544 | 2.833 | 4.377 |
|  |  | 23147 | 0.881 | 0.328 | 0.418 | 7.534 | 2.807 | 3.576 |
|  |  | 23148 | 0.906 | 0.297 | 0.440 | 8.849 | 2.905 | 4.298 |
|  |  | 23149 | 1.026 | 0.342 | 0.490 | 9.698 | 3.235 | 4.627 |
|  |  | 23150 | 1.040 | 0.357 | 0.537 | 8.239 | 2.831 | 4.251 |

Note: Weight (Unit:g); organ to body ratio (g / 100 g body weight); organ to brain ratio (g / g brain weight)

### Organ data of individual animal

| **Organ data of animals dissected on day 92** | | | | | | | | |
| --- | --- | --- | --- | --- | --- | --- | --- | --- |
| **Gender** | **Dose** | **Animal no.** | **Spleen** | | | **Kidneys** | | |
|  | **g/kg** |  | **Weight** | **Organ-to-body ratio** | **Organ-to-brain ratio** | **Weight** | **Organ-to-body ratio** | **Organ-to-brain ratio** |
| Female | 0 | 20021 | 0.467 | 0.176 | 0.232 | 1.889 | 0.712 | 0.940 |
|  |  | 20022 | 0.659 | 0.218 | 0.325 | 2.065 | 0.683 | 1.017 |
|  |  | 20023 | 0.681 | 0.206 | 0.341 | 2.012 | 0.608 | 1.006 |
|  |  | 20024 | 0.733 | 0.236 | 0.383 | 2.020 | 0.649 | 1.056 |
|  |  | 20025 | 0.537 | 0.183 | 0.260 | 2.075 | 0.709 | 1.006 |
|  |  | 20026 | 0.520 | 0.185 | 0.251 | 2.067 | 0.735 | 0.996 |
|  |  | 20027 | 0.511 | 0.161 | 0.252 | 1.963 | 0.620 | 0.968 |
|  |  | 20028 | 0.578 | 0.212 | 0.278 | 1.921 | 0.704 | 0.924 |
|  |  | 20029 | 0.524 | 0.185 | 0.273 | 2.082 | 0.734 | 1.086 |
|  |  | 20030 | 0.485 | 0.145 | 0.272 | 2.047 | 0.611 | 1.147 |
|  | 0.5 | 21061 | 0.482 | 0.188 | 0.262 | 1.816 | 0.710 | 0.986 |
|  |  | 21062 | 0.417 | 0.154 | 0.211 | 1.926 | 0.712 | 0.973 |
|  |  | 21063 | 0.532 | 0.204 | 0.246 | 1.728 | 0.662 | 0.800 |
|  |  | 21064 | 0.554 | 0.196 | 0.284 | 1.781 | 0.632 | 0.913 |
|  |  | 21065 | 0.453 | 0.145 | 0.239 | 1.866 | 0.598 | 0.986 |
|  |  | 21066 | 0.507 | 0.180 | 0.275 | 1.904 | 0.675 | 1.034 |
|  |  | 21067 | 0.634 | 0.196 | 0.304 | 2.267 | 0.699 | 1.088 |
|  |  | 21068 | 0.545 | 0.198 | 0.267 | 1.837 | 0.667 | 0.899 |
|  |  | 21069 | 0.577 | 0.195 | 0.262 | 2.117 | 0.716 | 0.960 |
|  |  | 21070 | 0.565 | 0.214 | 0.277 | 1.689 | 0.638 | 0.829 |
|  | 1.5 | 22101 | 0.514 | 0.197 | 0.264 | 1.852 | 0.710 | 0.952 |
|  |  | 22102 | 0.459 | 0.167 | 0.233 | 1.955 | 0.712 | 0.992 |
|  |  | 22103 | 0.497 | 0.173 | 0.258 | 1.657 | 0.577 | 0.859 |
|  |  | 22104 | 0.541 | 0.210 | 0.244 | 1.783 | 0.692 | 0.805 |
|  |  | 22105 | 0.587 | 0.233 | 0.297 | 1.692 | 0.672 | 0.857 |
|  |  | 22106 | 0.548 | 0.173 | 0.271 | 1.941 | 0.612 | 0.959 |
|  |  | 22107 | 0.540 | 0.180 | 0.264 | 1.982 | 0.660 | 0.968 |
|  |  | 22108 | 0.570 | 0.193 | 0.281 | 2.141 | 0.726 | 1.057 |
|  |  | 22109 | 0.608 | 0.201 | 0.300 | 2.040 | 0.675 | 1.008 |
|  |  | 22110 | 0.583 | 0.190 | 0.297 | 2.381 | 0.776 | 1.211 |
|  | 5 | 23141 | 0.502 | 0.182 | 0.250 | 2.088 | 0.758 | 1.039 |
|  |  | 23142 | 0.425 | 0.167 | 0.229 | 1.947 | 0.765 | 1.047 |
|  |  | 23143 | 0.487 | 0.174 | 0.253 | 1.981 | 0.707 | 1.028 |
|  |  | 23144 | 0.627 | 0.188 | 0.308 | 2.148 | 0.643 | 1.054 |
|  |  | 23145 | 0.681 | 0.217 | 0.359 | 2.151 | 0.685 | 1.133 |
|  |  | 23146 | 0.545 | 0.181 | 0.279 | 2.169 | 0.719 | 1.111 |
|  |  | 23147 | 0.504 | 0.188 | 0.239 | 2.185 | 0.814 | 1.037 |
|  |  | 23148 | 0.601 | 0.197 | 0.292 | 2.219 | 0.728 | 1.078 |
|  |  | 23149 | 0.650 | 0.217 | 0.310 | 2.174 | 0.725 | 1.037 |
|  |  | 23150 | 0.686 | 0.236 | 0.354 | 2.012 | 0.691 | 1.038 |

Note: Weight (Unit:g); organ to body ratio (g / 100 g body weight); organ to brain ratio (g / g brain weight)

### Organ data of individual animal

| **Organ data of animals dissected on day 92** | | | | | | | | |
| --- | --- | --- | --- | --- | --- | --- | --- | --- |
| **Gender** | **Dose** | **Animal no.** | **Thymus** | | | **Adrenal glands** | | |
|  | **g/kg** |  | **Weight** | **Organ-to-body ratio** | **Organ-to-brain ratio** | **Weight** | **Organ-to-body ratio** | **Organ-to-brain ratio** |
| Female | 0 | 20021 | 0.154 | 0.058 | 0.077 | 0.093 | 0.035 | 0.046 |
|  |  | 20022 | 0.195 | 0.065 | 0.096 | 0.086 | 0.028 | 0.042 |
|  |  | 20023 | 0.200 | 0.060 | 0.100 | 0.079 | 0.024 | 0.040 |
|  |  | 20024 | 0.337 | 0.108 | 0.176 | 0.085 | 0.027 | 0.044 |
|  |  | 20025 | 0.200 | 0.068 | 0.097 | 0.098 | 0.033 | 0.048 |
|  |  | 20026 | 0.171 | 0.061 | 0.082 | 0.089 | 0.032 | 0.043 |
|  |  | 20027 | 0.140 | 0.044 | 0.069 | 0.086 | 0.027 | 0.042 |
|  |  | 20028 | 0.186 | 0.068 | 0.089 | 0.090 | 0.033 | 0.043 |
|  |  | 20029 | 0.115 | 0.041 | 0.060 | 0.080 | 0.028 | 0.042 |
|  |  | 20030 | 0.314 | 0.094 | 0.176 | 0.068 | 0.020 | 0.038 |
|  | 0.5 | 21061 | 0.258 | 0.101 | 0.140 | 0.065 | 0.025 | 0.035 |
|  |  | 21062 | 0.182 | 0.067 | 0.092 | 0.065 | 0.024 | 0.033 |
|  |  | 21063 | 0.133 | 0.051 | 0.062 | 0.075 | 0.029 | 0.035 |
|  |  | 21064 | 0.209 | 0.074 | 0.107 | 0.061 | 0.022 | 0.031 |
|  |  | 21065 | 0.202 | 0.065 | 0.107 | 0.087 | 0.028 | 0.046 |
|  |  | 21066 | 0.189 | 0.067 | 0.103 | 0.064 | 0.023 | 0.035 |
|  |  | 21067 | 0.295 | 0.091 | 0.142 | 0.076 | 0.023 | 0.036 |
|  |  | 21068 | 0.224 | 0.081 | 0.110 | 0.065 | 0.024 | 0.032 |
|  |  | 21069 | 0.207 | 0.070 | 0.094 | 0.089 | 0.030 | 0.040 |
|  |  | 21070 | 0.163 | 0.062 | 0.080 | 0.071 | 0.027 | 0.035 |
|  | 1.5 | 22101 | 0.192 | 0.074 | 0.099 | 0.074 | 0.028 | 0.038 |
|  |  | 22102 | 0.162 | 0.059 | 0.082 | 0.069 | 0.025 | 0.035 |
|  |  | 22103 | 0.220 | 0.077 | 0.114 | 0.082 | 0.029 | 0.043 |
|  |  | 22104 | 0.146 | 0.057 | 0.066 | 0.082 | 0.032 | 0.037 |
|  |  | 22105 | 0.300 | 0.119 | 0.152 | 0.078 | 0.031 | 0.039 |
|  |  | 22106 | 0.229 | 0.072 | 0.113 | 0.085 | 0.027 | 0.042 |
|  |  | 22107 | 0.247 | 0.082 | 0.121 | 0.078 | 0.026 | 0.038 |
|  |  | 22108 | 0.236 | 0.080 | 0.117 | 0.078 | 0.026 | 0.039 |
|  |  | 22109 | 0.243 | 0.080 | 0.120 | 0.084 | 0.028 | 0.042 |
|  |  | 22110 | 0.261 | 0.085 | 0.133 | 0.091 | 0.030 | 0.046 |
|  | 5 | 23141 | 0.234 | 0.085 | 0.116 | 0.083 | 0.030 | 0.041 |
|  |  | 23142 | 0.181 | 0.071 | 0.097 | 0.066 | 0.026 | 0.036 |
|  |  | 23143 | 0.226 | 0.081 | 0.117 | 0.091 | 0.032 | 0.047 |
|  |  | 23144 | 0.320 | 0.096 | 0.157 | 0.082 | 0.025 | 0.040 |
|  |  | 23145 | 0.203 | 0.065 | 0.107 | 0.070 | 0.022 | 0.037 |
|  |  | 23146 | 0.281 | 0.093 | 0.144 | 0.092 | 0.031 | 0.047 |
|  |  | 23147 | 0.211 | 0.079 | 0.100 | 0.084 | 0.031 | 0.040 |
|  |  | 23148 | 0.227 | 0.075 | 0.110 | 0.083 | 0.027 | 0.040 |
|  |  | 23149 | 0.196 | 0.065 | 0.094 | 0.089 | 0.030 | 0.042 |
|  |  | 23150 | 0.249 | 0.086 | 0.128 | 0.087 | 0.030 | 0.045 |

Note: Weight (Unit:g); organ to body ratio (g / 100 g body weight); organ to brain ratio (g / g brain weight)

### Organ data of individual animal

| **Organ data of animals dissected on day 92** | | | | | | | | |
| --- | --- | --- | --- | --- | --- | --- | --- | --- |
| **Gender** | **Dose** | **Animal no.** | **Uterus** | | | **Ovaries** | | |
|  | **g/kg** |  | **Weight** | **Organ-to-body ratio** | **Organ-to-brain ratio** | **Weight** | **Organ-to-body ratio** | **Organ-to-brain ratio** |
| Female | 0 | 20021 | 0.596 | 0.225 | 0.297 | 0.131 | 0.049 | 0.065 |
|  |  | 20022 | 0.587 | 0.194 | 0.289 | 0.100 | 0.033 | 0.049 |
|  |  | 20023 | 0.709 | 0.214 | 0.355 | 0.097 | 0.029 | 0.049 |
|  |  | 20024 | 0.715 | 0.230 | 0.374 | 0.109 | 0.035 | 0.057 |
|  |  | 20025 | 0.602 | 0.206 | 0.292 | 0.079 | 0.027 | 0.038 |
|  |  | 20026 | 0.841 | 0.299 | 0.405 | 0.100 | 0.036 | 0.048 |
|  |  | 20027 | 0.876 | 0.277 | 0.432 | 0.076 | 0.024 | 0.037 |
|  |  | 20028 | 0.638 | 0.234 | 0.307 | 0.073 | 0.027 | 0.035 |
|  |  | 20029 | 0.493 | 0.174 | 0.257 | 0.101 | 0.036 | 0.053 |
|  |  | 20030 | 0.702 | 0.210 | 0.393 | 0.076 | 0.023 | 0.043 |
|  | 0.5 | 21061 | 0.589 | 0.230 | 0.320 | 0.074 | 0.029 | 0.040 |
|  |  | 21062 | 0.571 | 0.211 | 0.288 | 0.070 | 0.026 | 0.035 |
|  |  | 21063 | 0.619 | 0.237 | 0.286 | 0.119 | 0.046 | 0.055 |
|  |  | 21064 | 0.483 | 0.171 | 0.248 | 0.107 | 0.038 | 0.055 |
|  |  | 21065 | 0.594 | 0.190 | 0.314 | 0.077 | 0.025 | 0.041 |
|  |  | 21066 | 0.416 | 0.147 | 0.226 | 0.058 | 0.021 | 0.031 |
|  |  | 21067 | 0.480 | 0.148 | 0.230 | 0.082 | 0.025 | 0.039 |
|  |  | 21068 | 0.635 | 0.231 | 0.311 | 0.098 | 0.036 | 0.048 |
|  |  | 21069 | 0.708 | 0.239 | 0.321 | 0.090 | 0.030 | 0.041 |
|  |  | 21070 | 0.590 | 0.223 | 0.289 | 0.076 | 0.029 | 0.037 |
|  | 1.5 | 22101 | 0.802 | 0.308 | 0.412 | 0.066 | 0.025 | 0.034 |
|  |  | 22102 | 0.833 | 0.303 | 0.423 | 0.076 | 0.028 | 0.039 |
|  |  | 22103 | 0.597 | 0.208 | 0.310 | 0.108 | 0.038 | 0.056 |
|  |  | 22104 | 0.607 | 0.236 | 0.274 | 0.085 | 0.033 | 0.038 |
|  |  | 22105 | 0.664 | 0.264 | 0.336 | 0.092 | 0.037 | 0.047 |
|  |  | 22106 | 0.556 | 0.175 | 0.275 | 0.105 | 0.033 | 0.052 |
|  |  | 22107 | 0.474 | 0.158 | 0.231 | 0.101 | 0.034 | 0.049 |
|  |  | 22108 | 0.582 | 0.197 | 0.287 | 0.118 | 0.040 | 0.058 |
|  |  | 22109 | 0.648 | 0.214 | 0.320 | 0.110 | 0.036 | 0.054 |
|  |  | 22110 | 0.800 | 0.261 | 0.407 | 0.088 | 0.029 | 0.045 |
|  | 5 | 23141 | 0.562 | 0.204 | 0.280 | 0.092 | 0.033 | 0.046 |
|  |  | 23142 | 0.764 | 0.300 | 0.411 | 0.086 | 0.034 | 0.046 |
|  |  | 23143 | 0.711 | 0.254 | 0.369 | 0.059 | 0.021 | 0.031 |
|  |  | 23144 | 0.481 | 0.144 | 0.236 | 0.076 | 0.023 | 0.037 |
|  |  | 23145 | 0.850 | 0.271 | 0.448 | 0.105 | 0.033 | 0.055 |
|  |  | 23146 | 0.623 | 0.207 | 0.319 | 0.125 | 0.041 | 0.064 |
|  |  | 23147 | 0.713 | 0.266 | 0.338 | 0.099 | 0.037 | 0.047 |
|  |  | 23148 | 0.657 | 0.216 | 0.319 | 0.095 | 0.031 | 0.046 |
|  |  | 23149 | 0.474 | 0.158 | 0.226 | 0.106 | 0.035 | 0.051 |
|  |  | 23150 | 0.572 | 0.197 | 0.295 | 0.106 | 0.036 | 0.055 |

Note: Weight (Unit:g); organ to body ratio (g / 100 g body weight); organ to brain ratio (g / g brain weight)

### Organ data of individual animal

| **Organ data of animals dissected on day 92** | | | | | |
| --- | --- | --- | --- | --- | --- |
| **Gender** | **Dose** | **Animal no.** | **Brain** | | **Body weight** |
|  | **g/kg** |  | **Weight** | **Organ-to-body ratio** | **g** |
| Female | 0 | 20021 | 2.009 | 0.757 | 265.4 |
|  |  | 20022 | 2.030 | 0.672 | 302.2 |
|  |  | 20023 | 2.000 | 0.604 | 331.0 |
|  |  | 20024 | 1.912 | 0.614 | 311.2 |
|  |  | 20025 | 2.062 | 0.704 | 292.8 |
|  |  | 20026 | 2.075 | 0.738 | 281.3 |
|  |  | 20027 | 2.028 | 0.640 | 316.8 |
|  |  | 20028 | 2.080 | 0.762 | 272.8 |
|  |  | 20029 | 1.917 | 0.676 | 283.6 |
|  |  | 20030 | 1.784 | 0.533 | 335.0 |
|  | 0.5 | 21061 | 1.842 | 0.720 | 255.8 |
|  |  | 21062 | 1.980 | 0.732 | 270.6 |
|  |  | 21063 | 2.161 | 0.827 | 261.2 |
|  |  | 21064 | 1.951 | 0.692 | 282.0 |
|  |  | 21065 | 1.892 | 0.606 | 312.2 |
|  |  | 21066 | 1.842 | 0.653 | 282.1 |
|  |  | 21067 | 2.084 | 0.643 | 324.2 |
|  |  | 21068 | 2.043 | 0.742 | 275.3 |
|  |  | 21069 | 2.206 | 0.746 | 295.7 |
|  |  | 21070 | 2.038 | 0.770 | 264.6 |
|  | 1.5 | 22101 | 1.946 | 0.746 | 260.7 |
|  |  | 22102 | 1.971 | 0.718 | 274.7 |
|  |  | 22103 | 1.928 | 0.672 | 287.1 |
|  |  | 22104 | 2.216 | 0.861 | 257.5 |
|  |  | 22105 | 1.975 | 0.785 | 251.7 |
|  |  | 22106 | 2.023 | 0.638 | 317.0 |
|  |  | 22107 | 2.048 | 0.682 | 300.4 |
|  |  | 22108 | 2.025 | 0.686 | 295.0 |
|  |  | 22109 | 2.024 | 0.670 | 302.1 |
|  |  | 22110 | 1.966 | 0.640 | 307.0 |
|  | 5 | 23141 | 2.010 | 0.730 | 275.3 |
|  |  | 23142 | 1.859 | 0.730 | 254.5 |
|  |  | 23143 | 1.927 | 0.688 | 280.2 |
|  |  | 23144 | 2.037 | 0.610 | 334.2 |
|  |  | 23145 | 1.898 | 0.604 | 314.2 |
|  |  | 23146 | 1.952 | 0.647 | 301.6 |
|  |  | 23147 | 2.107 | 0.785 | 268.4 |
|  |  | 23148 | 2.059 | 0.676 | 304.6 |
|  |  | 23149 | 2.096 | 0.699 | 299.8 |
|  |  | 23150 | 1.938 | 0.666 | 291.0 |

Note: Weight (Unit:g); organ to body ratio (g / 100 g body weight); organ to brain ratio (g / g brain weight)

### Organ data of individual animal

| **Organ data of animals dissected on recovery day 29** | | | | | | | | |
| --- | --- | --- | --- | --- | --- | --- | --- | --- |
| **Gender** | **Dose** | **Animal no.** | **Heart** | | | **Liver** | | |
|  | **g/kg** |  | **Weight** | **Organ-to-body ratio** | **Organ-to-brain ratio** | **Weight** | **Organ-to-body ratio** | **Organ-to-brain ratio** |
| Male | 0 | 10011 | 1.840 | 0.283 | 0.820 | 15.586 | 2.396 | 6.949 |
|  |  | 10012 | 1.870 | 0.268 | 0.862 | 19.531 | 2.797 | 9.005 |
|  |  | 10013 | 1.557 | 0.290 | 0.736 | 16.033 | 2.988 | 7.581 |
|  |  | 10014 | 2.132 | 0.304 | 0.993 | 18.166 | 2.592 | 8.457 |
|  |  | 10015 | 2.129 | 0.315 | 0.982 | 17.762 | 2.625 | 8.197 |
|  | 0.5 | 11051 | 1.788 | 0.278 | 0.765 | 15.324 | 2.380 | 6.557 |
|  |  | 11052 | 1.685 | 0.281 | 0.795 | 15.321 | 2.551 | 7.230 |
|  |  | 11053 | 1.953 | 0.318 | 0.846 | 14.955 | 2.438 | 6.477 |
|  |  | 11054 | 2.176 | 0.292 | 1.018 | 23.767 | 3.194 | 11.116 |
|  |  | 11055 | 1.899 | 0.316 | 0.824 | 15.593 | 2.595 | 6.762 |
|  | 1.5 | 12091 | 1.845 | 0.280 | 0.796 | 17.494 | 2.659 | 7.550 |
|  |  | 12092 | 1.720 | 0.285 | 0.745 | 15.699 | 2.600 | 6.802 |
|  |  | 12093 | 1.684 | 0.266 | 0.738 | 14.800 | 2.341 | 6.488 |
|  |  | 12094 | 1.637 | 0.258 | 0.762 | 17.591 | 2.767 | 8.189 |
|  |  | 12095 | 1.773 | 0.277 | 0.804 | 16.056 | 2.511 | 7.278 |
|  | 5 | 13131 | 2.022 | 0.304 | 0.921 | 20.579 | 3.096 | 9.375 |
|  |  | 13132 | 1.815 | 0.295 | 0.776 | 15.810 | 2.569 | 6.762 |
|  |  | 13133 | 1.726 | 0.269 | 0.758 | 16.199 | 2.529 | 7.117 |
|  |  | 13134 | 1.708 | 0.302 | 0.849 | 13.459 | 2.381 | 6.689 |
|  |  | 13135 | 1.836 | 0.326 | 0.909 | 15.582 | 2.764 | 7.718 |
| Female | 0 | 20031 | 1.063 | 0.334 | 0.501 | 7.574 | 2.380 | 3.569 |
|  |  | 20032 | 0.986 | 0.332 | 0.536 | 7.012 | 2.363 | 3.813 |
|  |  | 20033 | 1.067 | 0.348 | 0.546 | 7.962 | 2.595 | 4.071 |
|  |  | 20034 | 1.202 | 0.331 | 0.560 | 10.309 | 2.841 | 4.806 |
|  |  | 20035 | 1.009 | 0.368 | 0.485 | 7.377 | 2.689 | 3.548 |
|  | 0.5 | 21071 | 1.102 | 0.346 | 0.518 | 8.052 | 2.530 | 3.786 |
|  |  | 21072 | 1.123 | 0.349 | 0.550 | 7.958 | 2.471 | 3.897 |
|  |  | 21073 | 1.086 | 0.352 | 0.503 | 6.906 | 2.239 | 3.199 |
|  |  | 21074 | 1.056 | 0.335 | 0.502 | 8.066 | 2.561 | 3.832 |
|  |  | 21075 | 1.050 | 0.353 | 0.551 | 8.342 | 2.803 | 4.379 |
|  | 1.5 | 22111 | 0.909 | 0.294 | 0.453 | 8.185 | 2.649 | 4.082 |
|  |  | 22112 | 1.125 | 0.345 | 0.568 | 7.817 | 2.399 | 3.950 |
|  |  | 22113 | 1.185 | 0.357 | 0.568 | 9.726 | 2.930 | 4.663 |
|  |  | 22114 | 1.050 | 0.306 | 0.493 | 7.582 | 2.208 | 3.563 |
|  |  | 22115 | 1.048 | 0.327 | 0.483 | 8.497 | 2.648 | 3.912 |
|  | 5 | 23151 | 1.041 | 0.320 | 0.491 | 8.856 | 2.723 | 4.177 |
|  |  | 23152 | 1.235 | 0.369 | 0.595 | 8.717 | 2.601 | 4.203 |
|  |  | 23153 | 0.994 | 0.339 | 0.494 | 7.712 | 2.634 | 3.829 |
|  |  | 23154 | 1.208 | 0.347 | 0.617 | 9.337 | 2.685 | 4.769 |
|  |  | 23155 | 1.047 | 0.385 | 0.484 | 7.527 | 2.770 | 3.483 |

Note: Weight (Unit:g); organ to body ratio (g / 100 g body weight); organ to brain ratio (g / g brain weight)

### Organ data of individual animal

| **Organ data of animals dissected on recovery day 29** | | | | | | | | |
| --- | --- | --- | --- | --- | --- | --- | --- | --- |
| **Gender** | **Dose** | **Animal no.** | **Spleen** | | | **Kidneys** | | |
|  | **g/kg** |  | **Weight** | **Organ-to-body ratio** | **Organ-to-brain ratio** | **Weight** | **Organ-to-body ratio** | **Organ-to-brain ratio** |
| Male | 0 | 10011 | 0.725 | 0.111 | 0.323 | 3.654 | 0.562 | 1.629 |
|  |  | 10012 | 0.953 | 0.136 | 0.439 | 3.922 | 0.562 | 1.808 |
|  |  | 10013 | 0.744 | 0.139 | 0.352 | 2.840 | 0.529 | 1.343 |
|  |  | 10014 | 0.915 | 0.131 | 0.426 | 4.876 | 0.696 | 2.270 |
|  |  | 10015 | 0.928 | 0.137 | 0.428 | 3.405 | 0.503 | 1.571 |
|  | 0.5 | 11051 | 0.821 | 0.127 | 0.351 | 4.449 | 0.691 | 1.904 |
|  |  | 11052 | 0.754 | 0.126 | 0.356 | 3.547 | 0.590 | 1.674 |
|  |  | 11053 | 0.939 | 0.153 | 0.407 | 3.799 | 0.619 | 1.645 |
|  |  | 11054 | 1.083 | 0.146 | 0.507 | 4.075 | 0.548 | 1.906 |
|  |  | 11055 | 1.108 | 0.184 | 0.480 | 3.726 | 0.620 | 1.616 |
|  | 1.5 | 12091 | 0.995 | 0.151 | 0.429 | 4.109 | 0.624 | 1.773 |
|  |  | 12092 | 0.767 | 0.127 | 0.332 | 3.699 | 0.613 | 1.603 |
|  |  | 12093 | 0.813 | 0.129 | 0.356 | 3.609 | 0.571 | 1.582 |
|  |  | 12094 | 0.873 | 0.137 | 0.406 | 3.794 | 0.597 | 1.766 |
|  |  | 12095 | 0.796 | 0.125 | 0.361 | 4.095 | 0.641 | 1.856 |
|  | 5 | 13131 | 1.006 | 0.151 | 0.458 | 4.013 | 0.604 | 1.828 |
|  |  | 13132 | 0.819 | 0.133 | 0.350 | 3.843 | 0.624 | 1.644 |
|  |  | 13133 | 1.035 | 0.162 | 0.455 | 4.170 | 0.651 | 1.832 |
|  |  | 13134 | 0.819 | 0.145 | 0.407 | 3.179 | 0.562 | 1.580 |
|  |  | 13135 | 0.960 | 0.170 | 0.475 | 3.425 | 0.607 | 1.696 |
| Female | 0 | 20031 | 0.600 | 0.189 | 0.283 | 2.519 | 0.792 | 1.187 |
|  |  | 20032 | 0.553 | 0.186 | 0.301 | 1.840 | 0.620 | 1.001 |
|  |  | 20033 | 0.580 | 0.189 | 0.297 | 1.932 | 0.630 | 0.988 |
|  |  | 20034 | 0.551 | 0.152 | 0.257 | 2.419 | 0.667 | 1.128 |
|  |  | 20035 | 0.556 | 0.203 | 0.267 | 1.859 | 0.678 | 0.894 |
|  | 0.5 | 21071 | 0.576 | 0.181 | 0.271 | 2.216 | 0.696 | 1.042 |
|  |  | 21072 | 0.665 | 0.207 | 0.326 | 2.059 | 0.639 | 1.008 |
|  |  | 21073 | 0.598 | 0.194 | 0.277 | 2.167 | 0.703 | 1.004 |
|  |  | 21074 | 0.514 | 0.163 | 0.244 | 2.059 | 0.654 | 0.978 |
|  |  | 21075 | 0.537 | 0.180 | 0.282 | 2.035 | 0.684 | 1.068 |
|  | 1.5 | 22111 | 0.515 | 0.167 | 0.257 | 1.723 | 0.558 | 0.859 |
|  |  | 22112 | 0.602 | 0.185 | 0.304 | 1.926 | 0.591 | 0.973 |
|  |  | 22113 | 0.473 | 0.142 | 0.227 | 2.364 | 0.712 | 1.133 |
|  |  | 22114 | 0.525 | 0.153 | 0.247 | 1.965 | 0.572 | 0.923 |
|  |  | 22115 | 0.582 | 0.181 | 0.268 | 1.871 | 0.583 | 0.861 |
|  | 5 | 23151 | 0.506 | 0.156 | 0.239 | 2.119 | 0.652 | 1.000 |
|  |  | 23152 | 0.676 | 0.202 | 0.326 | 2.040 | 0.609 | 0.984 |
|  |  | 23153 | 0.515 | 0.176 | 0.256 | 1.934 | 0.661 | 0.960 |
|  |  | 23154 | 0.747 | 0.215 | 0.382 | 2.387 | 0.686 | 1.219 |
|  |  | 23155 | 0.474 | 0.174 | 0.219 | 1.943 | 0.715 | 0.899 |

Note: Weight (Unit:g); organ to body ratio (g / 100 g body weight); organ to brain ratio (g / g brain weight)

### Organ data of individual animal

| **Organ data of animals dissected on recovery day 29** | | | | | | | | |
| --- | --- | --- | --- | --- | --- | --- | --- | --- |
| **Gender** | **Dose** | **Animal no.** | **Thymus** | | | **Adrenal glands** | | |
|  | **g/kg** |  | **Weight** | **Organ-to-body ratio** | **Organ-to-brain ratio** | **Weight** | **Organ-to-body ratio** | **Organ-to-brain ratio** |
| Male | 0 | 10011 | 0.342 | 0.053 | 0.152 | 0.057 | 0.009 | 0.025 |
|  |  | 10012 | 0.177 | 0.025 | 0.082 | 0.079 | 0.011 | 0.036 |
|  |  | 10013 | 0.282 | 0.053 | 0.133 | 0.063 | 0.012 | 0.030 |
|  |  | 10014 | 0.182 | 0.026 | 0.085 | 0.087 | 0.012 | 0.041 |
|  |  | 10015 | 0.382 | 0.056 | 0.176 | 0.072 | 0.011 | 0.033 |
|  | 0.5 | 11051 | 0.248 | 0.039 | 0.106 | 0.064 | 0.010 | 0.027 |
|  |  | 11052 | 0.272 | 0.045 | 0.128 | 0.079 | 0.013 | 0.037 |
|  |  | 11053 | 0.235 | 0.038 | 0.102 | 0.061 | 0.010 | 0.026 |
|  |  | 11054 | 0.182 | 0.024 | 0.085 | 0.060 | 0.008 | 0.028 |
|  |  | 11055 | 0.310 | 0.052 | 0.134 | 0.082 | 0.014 | 0.036 |
|  | 1.5 | 12091 | 0.303 | 0.046 | 0.131 | 0.064 | 0.010 | 0.028 |
|  |  | 12092 | 0.241 | 0.040 | 0.104 | 0.069 | 0.011 | 0.030 |
|  |  | 12093 | 0.265 | 0.042 | 0.116 | 0.070 | 0.011 | 0.031 |
|  |  | 12094 | 0.281 | 0.044 | 0.131 | 0.067 | 0.011 | 0.031 |
|  |  | 12095 | 0.318 | 0.050 | 0.144 | 0.074 | 0.012 | 0.034 |
|  | 5 | 13131 | 0.340 | 0.051 | 0.155 | 0.069 | 0.010 | 0.031 |
|  |  | 13132 | 0.294 | 0.048 | 0.126 | 0.062 | 0.010 | 0.027 |
|  |  | 13133 | 0.170 | 0.027 | 0.075 | 0.070 | 0.011 | 0.031 |
|  |  | 13134 | 0.293 | 0.052 | 0.146 | 0.060 | 0.011 | 0.030 |
|  |  | 13135 | 0.298 | 0.053 | 0.148 | 0.058 | 0.010 | 0.029 |
| Female | 0 | 20031 | 0.151 | 0.047 | 0.071 | 0.078 | 0.025 | 0.037 |
|  |  | 20032 | 0.253 | 0.085 | 0.138 | 0.070 | 0.024 | 0.038 |
|  |  | 20033 | 0.203 | 0.066 | 0.104 | 0.073 | 0.024 | 0.037 |
|  |  | 20034 | 0.261 | 0.072 | 0.122 | 0.087 | 0.024 | 0.041 |
|  |  | 20035 | 0.178 | 0.065 | 0.086 | 0.064 | 0.023 | 0.031 |
|  | 0.5 | 21071 | 0.254 | 0.080 | 0.119 | 0.095 | 0.030 | 0.045 |
|  |  | 21072 | 0.244 | 0.076 | 0.119 | 0.078 | 0.024 | 0.038 |
|  |  | 21073 | 0.269 | 0.087 | 0.125 | 0.082 | 0.027 | 0.038 |
|  |  | 21074 | 0.176 | 0.056 | 0.084 | 0.096 | 0.030 | 0.046 |
|  |  | 21075 | 0.228 | 0.077 | 0.120 | 0.088 | 0.030 | 0.046 |
|  | 1.5 | 22111 | 0.171 | 0.055 | 0.085 | 0.065 | 0.021 | 0.032 |
|  |  | 22112 | 0.345 | 0.106 | 0.174 | 0.082 | 0.025 | 0.041 |
|  |  | 22113 | 0.157 | 0.047 | 0.075 | 0.069 | 0.021 | 0.033 |
|  |  | 22114 | 0.283 | 0.082 | 0.133 | 0.074 | 0.022 | 0.035 |
|  |  | 22115 | 0.205 | 0.064 | 0.094 | 0.065 | 0.020 | 0.030 |
|  | 5 | 23151 | 0.377 | 0.116 | 0.178 | 0.094 | 0.029 | 0.044 |
|  |  | 23152 | 0.168 | 0.050 | 0.081 | 0.049 | 0.015 | 0.024 |
|  |  | 23153 | 0.208 | 0.071 | 0.103 | 0.056 | 0.019 | 0.028 |
|  |  | 23154 | 0.199 | 0.057 | 0.102 | 0.084 | 0.024 | 0.043 |
|  |  | 23155 | 0.158 | 0.058 | 0.073 | 0.065 | 0.024 | 0.030 |

Note: Weight (Unit:g); organ to body ratio (g / 100 g body weight); organ to brain ratio (g / g brain weight)

### Organ data of individual animal

| **Organ data of animals dissected on recovery day 29** | | | | | | | | |
| --- | --- | --- | --- | --- | --- | --- | --- | --- |
| **Gender** | **Dose** | **Animal no.** | **Testes** | | | **Epididymides** | | |
|  | **g/kg** |  | **Weight** | **Organ-to-body ratio** | **Organ-to-brain ratio** | **Weight** | **Organ-to-body ratio** | **Organ-to-brain ratio** |
| Male | 0 | 10011 | 3.198 | 0.492 | 1.426 | 1.518 | 0.233 | 0.677 |
|  |  | 10012 | 3.625 | 0.519 | 1.671 | 1.531 | 0.219 | 0.706 |
|  |  | 10013 | 3.564 | 0.664 | 1.685 | 1.437 | 0.268 | 0.679 |
|  |  | 10014 | 4.005 | 0.571 | 1.865 | 1.560 | 0.223 | 0.726 |
|  |  | 10015 | 3.481 | 0.514 | 1.606 | 1.575 | 0.233 | 0.727 |
|  | 0.5 | 11051 | 3.811 | 0.592 | 1.631 | 1.612 | 0.250 | 0.690 |
|  |  | 11052 | 3.735 | 0.622 | 1.763 | 1.491 | 0.248 | 0.704 |
|  |  | 11053 | 3.628 | 0.591 | 1.571 | 1.723 | 0.281 | 0.746 |
|  |  | 11054 | 3.672 | 0.493 | 1.717 | 1.491 | 0.200 | 0.697 |
|  |  | 11055 | 3.183 | 0.530 | 1.380 | 1.408 | 0.234 | 0.611 |
|  | 1.5 | 12091 | 3.667 | 0.557 | 1.583 | 1.502 | 0.228 | 0.648 |
|  |  | 12092 | 3.656 | 0.606 | 1.584 | 1.620 | 0.268 | 0.702 |
|  |  | 12093 | 3.966 | 0.627 | 1.739 | 1.520 | 0.240 | 0.666 |
|  |  | 12094 | 4.067 | 0.640 | 1.893 | 1.591 | 0.250 | 0.741 |
|  |  | 12095 | 4.026 | 0.630 | 1.825 | 1.601 | 0.250 | 0.726 |
|  | 5 | 13131 | 3.448 | 0.519 | 1.571 | 1.533 | 0.231 | 0.698 |
|  |  | 13132 | 3.183 | 0.517 | 1.361 | 1.683 | 0.273 | 0.720 |
|  |  | 13133 | 3.494 | 0.546 | 1.535 | 1.508 | 0.235 | 0.663 |
|  |  | 13134 | 2.907 | 0.514 | 1.445 | 1.544 | 0.273 | 0.767 |
|  |  | 13135 | 3.922 | 0.696 | 1.943 | 1.339 | 0.237 | 0.663 |

Note: Weight (Unit:g); organ to body ratio (g / 100 g body weight); organ to brain ratio (g / g brain weight)

### Organ data of individual animal

| **Organ data of animals dissected on recovery day 29** | | | | | | | | |
| --- | --- | --- | --- | --- | --- | --- | --- | --- |
| **Gender** | **Dose** | **Animal no.** | **Uterus** | | | **Ovaries** | | |
|  | **g/kg** |  | **Weight** | **Organ-to-body ratio** | **Organ-to-brain ratio** | **Weight** | **Organ-to-body ratio** | **Organ-to-brain ratio** |
| Female | 0 | 20031 | 0.833 | 0.262 | 0.393 | 0.063 | 0.020 | 0.030 |
|  |  | 20032 | 0.653 | 0.220 | 0.355 | 0.080 | 0.027 | 0.044 |
|  |  | 20033 | 0.702 | 0.229 | 0.359 | 0.068 | 0.022 | 0.035 |
|  |  | 20034 | 0.651 | 0.179 | 0.303 | 0.080 | 0.022 | 0.037 |
|  |  | 20035 | 0.713 | 0.260 | 0.343 | 0.072 | 0.026 | 0.035 |
|  | 0.5 | 21071 | 0.879 | 0.276 | 0.413 | 0.087 | 0.027 | 0.041 |
|  |  | 21072 | 0.715 | 0.222 | 0.350 | 0.081 | 0.025 | 0.040 |
|  |  | 21073 | 0.570 | 0.185 | 0.264 | 0.121 | 0.039 | 0.056 |
|  |  | 21074 | 0.775 | 0.246 | 0.368 | 0.088 | 0.028 | 0.042 |
|  |  | 21075 | 0.568 | 0.191 | 0.298 | 0.080 | 0.027 | 0.042 |
|  | 1.5 | 22111 | 0.612 | 0.198 | 0.305 | 0.082 | 0.027 | 0.041 |
|  |  | 22112 | 0.597 | 0.183 | 0.302 | 0.105 | 0.032 | 0.053 |
|  |  | 22113 | 0.546 | 0.164 | 0.262 | 0.098 | 0.030 | 0.047 |
|  |  | 22114 | 0.601 | 0.175 | 0.282 | 0.083 | 0.024 | 0.039 |
|  |  | 22115 | 0.598 | 0.186 | 0.275 | 0.077 | 0.024 | 0.035 |
|  | 5 | 23151 | 0.686 | 0.211 | 0.324 | 0.077 | 0.024 | 0.036 |
|  |  | 23152 | 0.726 | 0.217 | 0.350 | 0.096 | 0.029 | 0.046 |
|  |  | 23153 | 0.453 | 0.155 | 0.225 | 0.070 | 0.024 | 0.035 |
|  |  | 23154 | 0.572 | 0.164 | 0.292 | 0.098 | 0.028 | 0.050 |
|  |  | 23155 | 0.567 | 0.209 | 0.262 | 0.102 | 0.038 | 0.047 |

Note: Weight (Unit:g); organ to body ratio (g / 100 g body weight); organ to brain ratio (g / g brain weight)

### Organ data of individual animal

| **Organ data of animals dissected on recovery day 29** | | | | | |
| --- | --- | --- | --- | --- | --- |
| **Gender** | **Dose** | **Animal no.** | **Brain** | | **Body weight** |
|  | **g/kg** |  | **Weight** | **Organ-to-body ratio** | **g** |
| Male | 0 | 10011 | 2.243 | 0.345 | 650.4 |
|  |  | 10012 | 2.169 | 0.311 | 698.3 |
|  |  | 10013 | 2.115 | 0.394 | 536.5 |
|  |  | 10014 | 2.148 | 0.307 | 700.8 |
|  |  | 10015 | 2.167 | 0.320 | 676.6 |
|  | 0.5 | 11051 | 2.337 | 0.363 | 644.0 |
|  |  | 11052 | 2.119 | 0.353 | 600.7 |
|  |  | 11053 | 2.309 | 0.376 | 613.4 |
|  |  | 11054 | 2.138 | 0.287 | 744.1 |
|  |  | 11055 | 2.306 | 0.384 | 600.8 |
|  | 1.5 | 12091 | 2.317 | 0.352 | 658.0 |
|  |  | 12092 | 2.308 | 0.382 | 603.7 |
|  |  | 12093 | 2.281 | 0.361 | 632.3 |
|  |  | 12094 | 2.148 | 0.338 | 635.7 |
|  |  | 12095 | 2.206 | 0.345 | 639.3 |
|  | 5 | 13131 | 2.195 | 0.330 | 664.7 |
|  |  | 13132 | 2.338 | 0.380 | 615.5 |
|  |  | 13133 | 2.276 | 0.355 | 640.5 |
|  |  | 13134 | 2.012 | 0.356 | 565.2 |
|  |  | 13135 | 2.019 | 0.358 | 563.8 |
| Female | 0 | 20031 | 2.122 | 0.667 | 318.2 |
|  |  | 20032 | 1.839 | 0.620 | 296.7 |
|  |  | 20033 | 1.956 | 0.638 | 306.8 |
|  |  | 20034 | 2.145 | 0.591 | 362.9 |
|  |  | 20035 | 2.079 | 0.758 | 274.3 |
|  | 0.5 | 21071 | 2.127 | 0.668 | 318.3 |
|  |  | 21072 | 2.042 | 0.634 | 322.0 |
|  |  | 21073 | 2.159 | 0.700 | 308.4 |
|  |  | 21074 | 2.105 | 0.668 | 315.0 |
|  |  | 21075 | 1.905 | 0.640 | 297.6 |
|  | 1.5 | 22111 | 2.005 | 0.649 | 309.0 |
|  |  | 22112 | 1.979 | 0.607 | 325.8 |
|  |  | 22113 | 2.086 | 0.628 | 332.0 |
|  |  | 22114 | 2.128 | 0.620 | 343.4 |
|  |  | 22115 | 2.172 | 0.677 | 320.9 |
|  | 5 | 23151 | 2.120 | 0.652 | 325.2 |
|  |  | 23152 | 2.074 | 0.619 | 335.1 |
|  |  | 23153 | 2.014 | 0.688 | 292.8 |
|  |  | 23154 | 1.958 | 0.563 | 347.8 |
|  |  | 23155 | 2.161 | 0.795 | 271.7 |

Note: Weight (Unit:g); organ to body ratio (g / 100 g body weight); organ to brain ratio (g / g brain weight)

### Bone marrow cell test data of individual animal

| **Bone marrow cell test data of individual animal** | | | | | | | | | |
| --- | --- | --- | --- | --- | --- | --- | --- | --- | --- |
| **Dose**  **(g/kg)** | **Gender** | **Animal no.** | **Myeloblast (%)** | **Promyelocyte (%)** | **Neutrophilic myelocyte (%)** | **Neutrophilic metamyelocyte (%)** | **Banded neutrophils (%)** | **Segmented neutrophils (%)** | **Segmented eosinophils (%)** |
| 0 | Male | 10001 | 0.5 | 5.5 | 9.0 | 12.0 | 17.0 | 14.0 | 3.0 |
|  |  | 10002 | 0.5 | 1.5 | 6.5 | 11.0 | 19.5 | 16.0 | 8.5 |
|  |  | 10003 |  | 3.5 | 3.0 | 14.0 | 18.0 | 12.0 | 6.0 |
|  |  | 10004 |  | 3.5 | 5.5 | 16.5 | 12.5 | 11.0 | 6.0 |
|  |  | 10005 | 0.5 | 3.5 | 6.0 | 11.5 | 16.5 | 12.0 | 3.5 |
|  |  | 10006 |  | 2.5 | 8.5 | 12.0 | 11.5 | 14.0 | 5.0 |
|  |  | 10007 | 0.5 | 2.5 | 5.5 | 15.5 | 13.5 | 11.5 | 7.5 |
|  |  | 10008 |  | 4.0 | 6.0 | 17.0 | 19.5 | 17.5 | 4.5 |
|  |  | 10009 |  | 4.5 | 4.0 | 17.0 | 10.5 | 15.5 | 6.0 |
|  |  | 10010 |  | 5.0 | 5.0 | 12.5 | 12.5 | 13.5 | 5.5 |
|  | Female | 20021 | 0.5 | 4.0 | 6.0 | 10.0 | 15.5 | 7.5 | 4.5 |
|  |  | 20022 |  | 2.5 | 5.0 | 12.0 | 16.5 | 9.5 | 3.5 |
|  |  | 20023 |  | 2.5 | 4.0 | 13.5 | 15.0 | 9.0 | 5.0 |
|  |  | 20024 | 1.0 | 5.0 | 2.0 | 9.5 | 13.5 | 3.5 | 5.0 |
|  |  | 20025 | 0.5 | 5.0 | 4.0 | 5.0 | 18.5 | 13.5 | 4.0 |
|  |  | 20026 | 1.0 | 2.0 | 3.0 | 12.5 | 16.5 | 10.0 | 1.5 |
|  |  | 20027 |  | 1.0 | 3.5 | 11.0 | 19.5 | 10.5 | 4.5 |
|  |  | 20028 |  | 3.5 | 4.5 | 10.0 | 21.0 | 14.0 | 4.5 |
|  |  | 20029 |  | 0.5 | 6.0 | 13.0 | 20.0 | 15.5 | 5.0 |
|  |  | 20030 |  | 2.5 | 4.0 | 10.0 | 21.5 | 10.5 | 6.5 |
| 5 | Male | 13121 |  | 2.0 | 5.5 | 11.5 | 17.5 | 11.5 | 2.5 |
|  |  | 13122 |  | 4.5 | 5.5 | 14.5 | 16.5 | 10.0 | 2.5 |
|  |  | 13123 | 0.5 | 2.0 | 3.0 | 11.5 | 12.5 | 10.0 | 5.0 |
|  |  | 13124 |  | 3.5 | 4.0 | 17.5 | 19.5 | 5.5 | 4.0 |
|  |  | 13125 |  | 3.0 | 2.5 | 14.0 | 17.0 | 14.0 | 4.5 |
|  |  | 13126 | 0.5 | 1.5 | 6.0 | 13.5 | 18.0 | 9.5 | 3.0 |
|  |  | 13127 |  | 3.5 | 2.5 | 8.5 | 13.5 | 13.5 | 6.0 |
|  |  | 13128 |  | 3.5 | 4.5 | 15.5 | 17.5 | 12.0 | 5.0 |
|  |  | 13129 |  | 4.5 | 6.0 | 15.5 | 14.5 | 9.5 | 5.0 |
|  |  | 13130 |  | 5.0 | 5.5 | 13.0 | 15.0 | 11.0 | 7.0 |
|  | Female | 23141 | 0.5 | 1.5 | 3.0 | 9.5 | 15.5 | 16.0 | 4.0 |
|  |  | 23142 |  | 7.0 | 2.5 | 11.0 | 14.0 | 11.5 | 4.5 |
|  |  | 23143 |  | 4.0 | 3.0 | 14.0 | 12.5 | 12.0 | 6.5 |
|  |  | 23144 |  | 2.5 | 4.5 | 12.0 | 17.0 | 16.0 | 6.0 |
|  |  | 23145 | 0.5 | 2.0 | 4.5 | 15.5 | 12.5 | 18.0 | 3.0 |
|  |  | 23146 |  | 5.0 | 4.0 | 13.0 | 14.5 | 18.5 | 4.0 |
|  |  | 23147 | 0.5 | 3.0 | 5.0 | 13.0 | 5.5 | 8.0 | 3.0 |
|  |  | 23148 |  | 2.5 | 5.5 | 11.5 | 8.5 | 14.0 | 5.5 |
|  |  | 23149 | 0.5 | 3.0 | 3.0 | 11.0 | 12.0 | 18.0 | 6.5 |
|  |  | 23150 | 0.5 | 2.0 | 6.0 | 14.0 | 15.0 | 17.0 | 5.0 |

### Bone marrow cell test data of individual animal

| **Bone marrow cell test data of individual animal** | | | | | | | | | | |
| --- | --- | --- | --- | --- | --- | --- | --- | --- | --- | --- |
| **Dose**  **(g/kg)** | **Gender** | **Animal no.** | **Segmented eosinophils (%)** | **Early normoblast (%)** | **Intermediate normoblast (%)** | **Late normoblast (%)** | **Myeloids/**  **erythroblasts** | **Mature lymphocyte (%)** | **Mature monocyte (%)** | **Mature plasma cell (%)** |
| 0 | Male | 10001 |  | 1.0 | 11.5 | 17.0 | 2.07:1 | 9.5 |  |  |
|  |  | 10002 |  |  | 8.0 | 15.5 | 2.70:1 | 12.0 | 1.0 |  |
|  |  | 10003 |  |  | 11.5 | 15.5 | 2.09:1 | 16.5 |  |  |
|  |  | 10004 |  |  | 11.0 | 21.0 | 1.72:1 | 12.5 |  | 0.5 |
|  |  | 10005 |  |  | 10.0 | 23.5 | 1.60:1 | 12.0 | 0.5 | 0.5 |
|  |  | 10006 |  |  | 9.0 | 24.0 | 1.62:1 | 12.0 | 0.5 | 1.0 |
|  |  | 10007 |  |  | 10.0 | 22.5 | 1.74:1 | 10.5 | 0.5 |  |
|  |  | 10008 |  |  | 8.5 | 15.0 | 2.91:1 | 7.5 |  | 0.5 |
|  |  | 10009 |  |  | 9.0 | 18.0 | 2.13:1 | 13.0 | 1.5 | 1.0 |
|  |  | 10010 |  |  | 6.5 | 19.0 | 2.12:1 | 17.5 | 2.0 | 1.0 |
|  | Female | 20016 |  | 0.5 | 12.0 | 21.0 | 1.43:1 | 16.5 | 0.5 | 1.5 |
|  |  | 20017 |  |  | 13.5 | 17.0 | 1.61:1 | 19.0 | 0.5 | 1.0 |
|  |  | 20018 |  | 0.5 | 11.0 | 19.5 | 1.58:1 | 18.0 | 0.5 | 1.5 |
|  |  | 20019 |  | 0.5 | 18.0 | 26.0 | 0.89:1 | 14.0 | 0.5 | 1.5 |
|  |  | 20020 |  | 1.0 | 6.5 | 23.5 | 1.63:1 | 16.0 | 1.0 | 1.5 |
|  |  | 20021 |  | 0.5 | 13.5 | 23.0 | 1.26:1 | 15.5 | 0.5 | 0.5 |
|  |  | 20022 |  |  | 12.5 | 20.5 | 1.52:1 | 15.5 |  | 1.5 |
|  |  | 20023 |  | 1.0 | 15.0 | 20.0 | 1.60:1 | 5.0 |  | 1.5 |
|  |  | 20024 |  | 0.5 | 11.5 | 16.5 | 2.11:1 | 10.0 | 1.0 | 0.5 |
|  |  | 20025 |  | 1.0 | 6.0 | 19.5 | 2.08:1 | 15.5 | 2.0 | 1.0 |
| 5 | Male | 13091 |  | 0.5 | 12.5 | 19.0 | 1.58:1 | 16.5 |  | 1.0 |
|  |  | 13092 |  |  | 13.0 | 17.0 | 1.78:1 | 15.5 |  | 1.0 |
|  |  | 13093 |  | 0.5 | 12.5 | 24.0 | 1.20:1 | 17.5 | 1.0 |  |
|  |  | 13094 |  | 0.5 | 8.0 | 16.5 | 2.16:1 | 20.5 | 0.5 |  |
|  |  | 13095 |  |  | 11.0 | 14.5 | 2.16:1 | 18.5 |  | 1.0 |
|  |  | 13096 |  |  | 11.5 | 17.5 | 1.79:1 | 17.0 | 0.5 | 1.5 |
|  |  | 13097 |  | 0.5 | 13.0 | 19.5 | 1.44:1 | 19.0 |  | 0.5 |
|  |  | 13098 |  | 0.5 | 9.5 | 16.0 | 2.23:1 | 15.0 | 0.5 | 0.5 |
|  |  | 13099 |  | 0.5 | 9.5 | 20.5 | 1.80:1 | 13.0 | 1.0 | 0.5 |
|  |  | 13100 |  |  | 12.5 | 18.0 | 1.85:1 | 12.0 | 0.5 | 0.5 |
|  | Female | 23106 |  | 1.0 | 7.5 | 17.0 | 1.96:1 | 21.5 | 1.0 | 2.0 |
|  |  | 23107 |  | 1.0 | 14.0 | 20.0 | 1.44:1 | 14.0 | 0.5 |  |
|  |  | 23108 |  | 0.5 | 10.5 | 20.0 | 1.68:1 | 16.0 | 0.5 | 0.5 |
|  |  | 23109 |  |  | 8.5 | 19.5 | 2.07:1 | 14.0 |  |  |
|  |  | 23110 |  | 0.5 | 12.0 | 19.0 | 1.78:1 | 11.5 |  | 1.0 |
|  |  | 23111 |  |  | 10.5 | 20.0 | 1.93:1 | 8.5 | 1.0 | 1.0 |
|  |  | 23112 |  | 1.5 | 16.5 | 25.5 | 0.87:1 | 16.5 | 1.0 | 1.0 |
|  |  | 23113 |  |  | 15.0 | 24.0 | 1.22:1 | 12.0 | 0.5 | 1.0 |
|  |  | 23114 | 0.5 |  | 9.5 | 19.5 | 1.88:1 | 16.5 |  |  |
|  |  | 23115 |  | 0.5 | 9.0 | 18.0 | 2.16:1 | 12.0 |  | 1.0 |

# Attached Pathology Figures

| 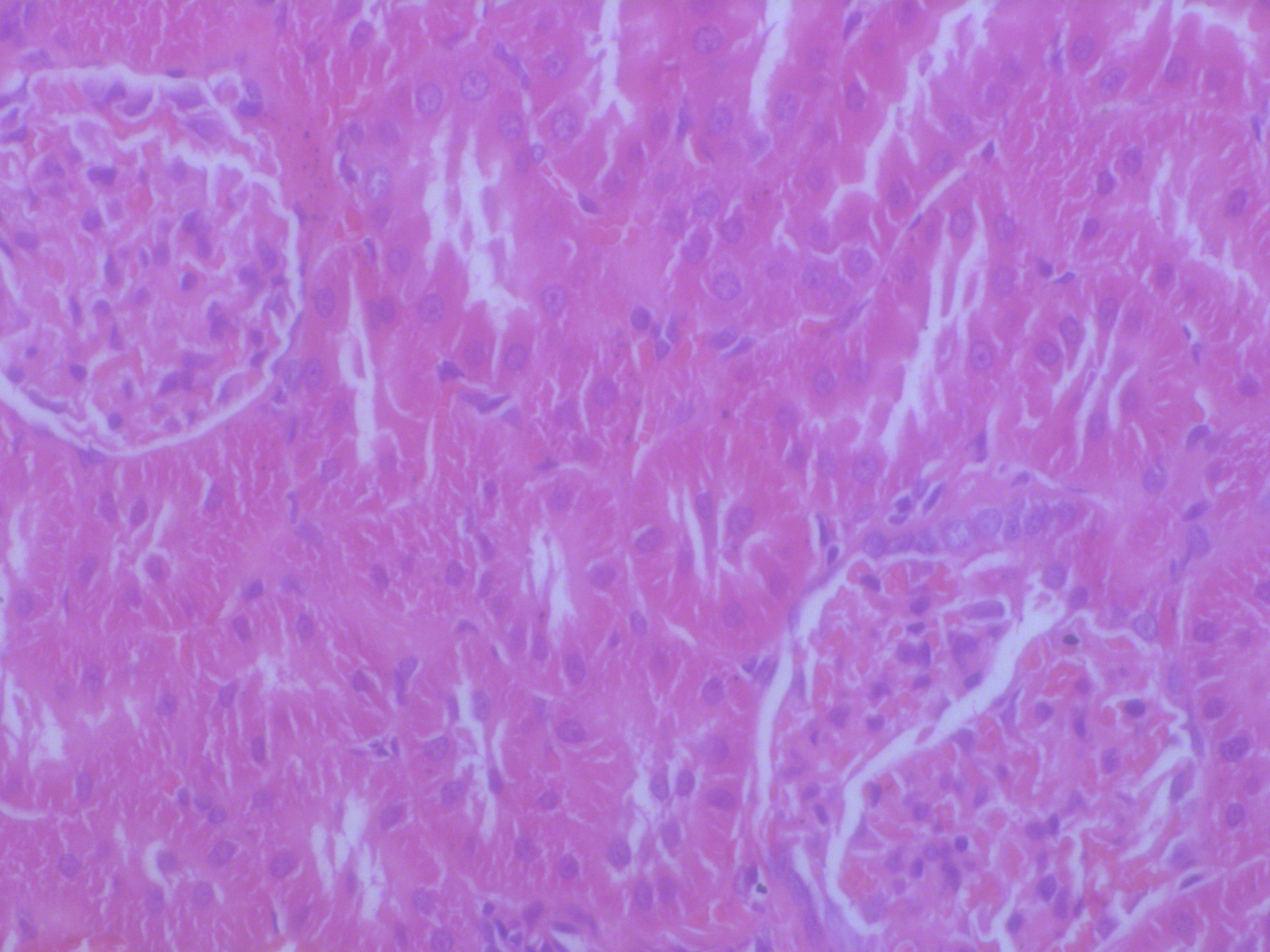 | 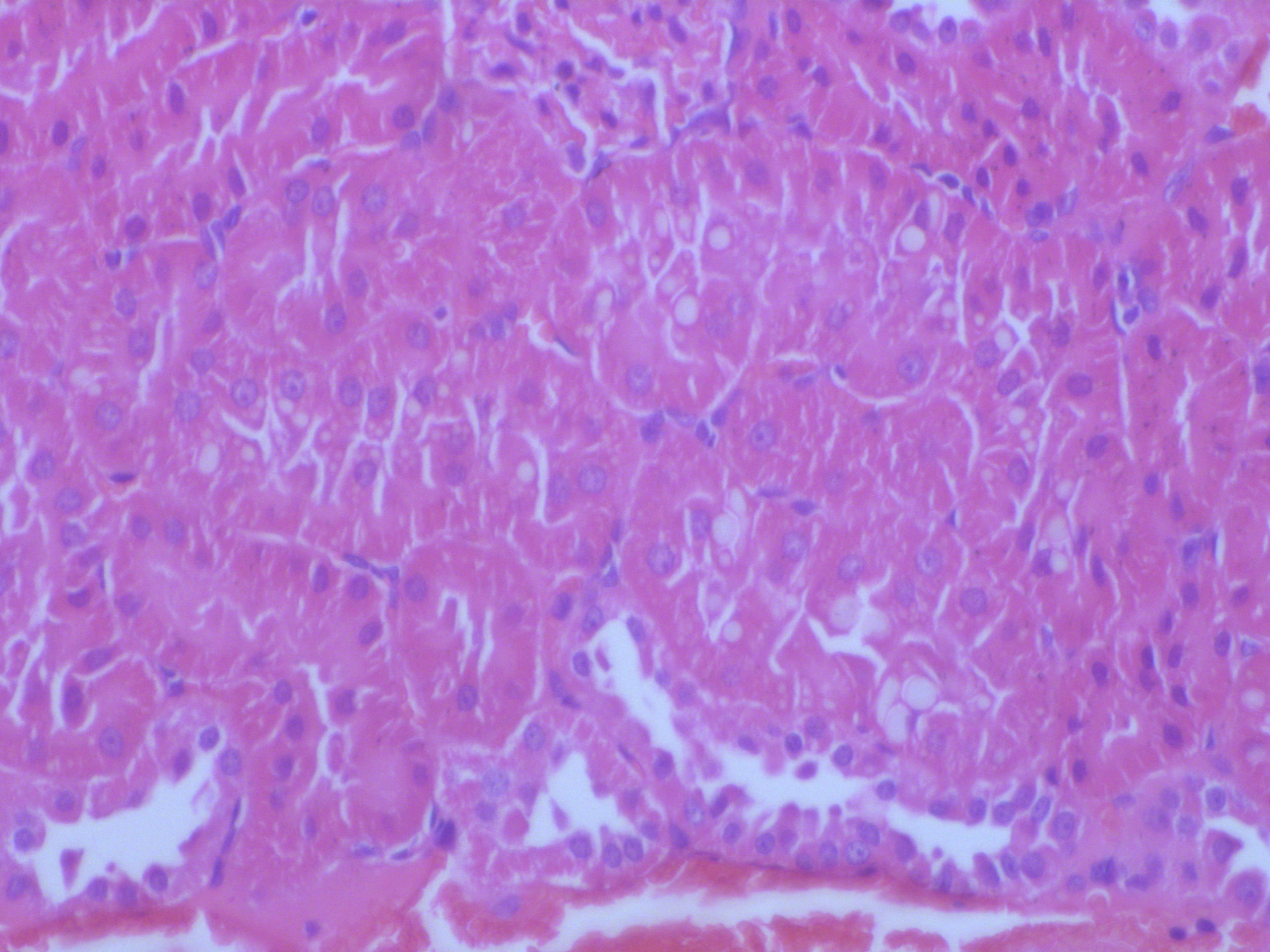 |
| --- | --- |
| Figure 1. #10001 in negative control group, normal kidney in the field of view, HE stained, ×400 | Figure 2. #13121 in high-dose group, kidney, multifocal hyaline droplet accumulation in renal tubules, HE stained, ×400 |
| 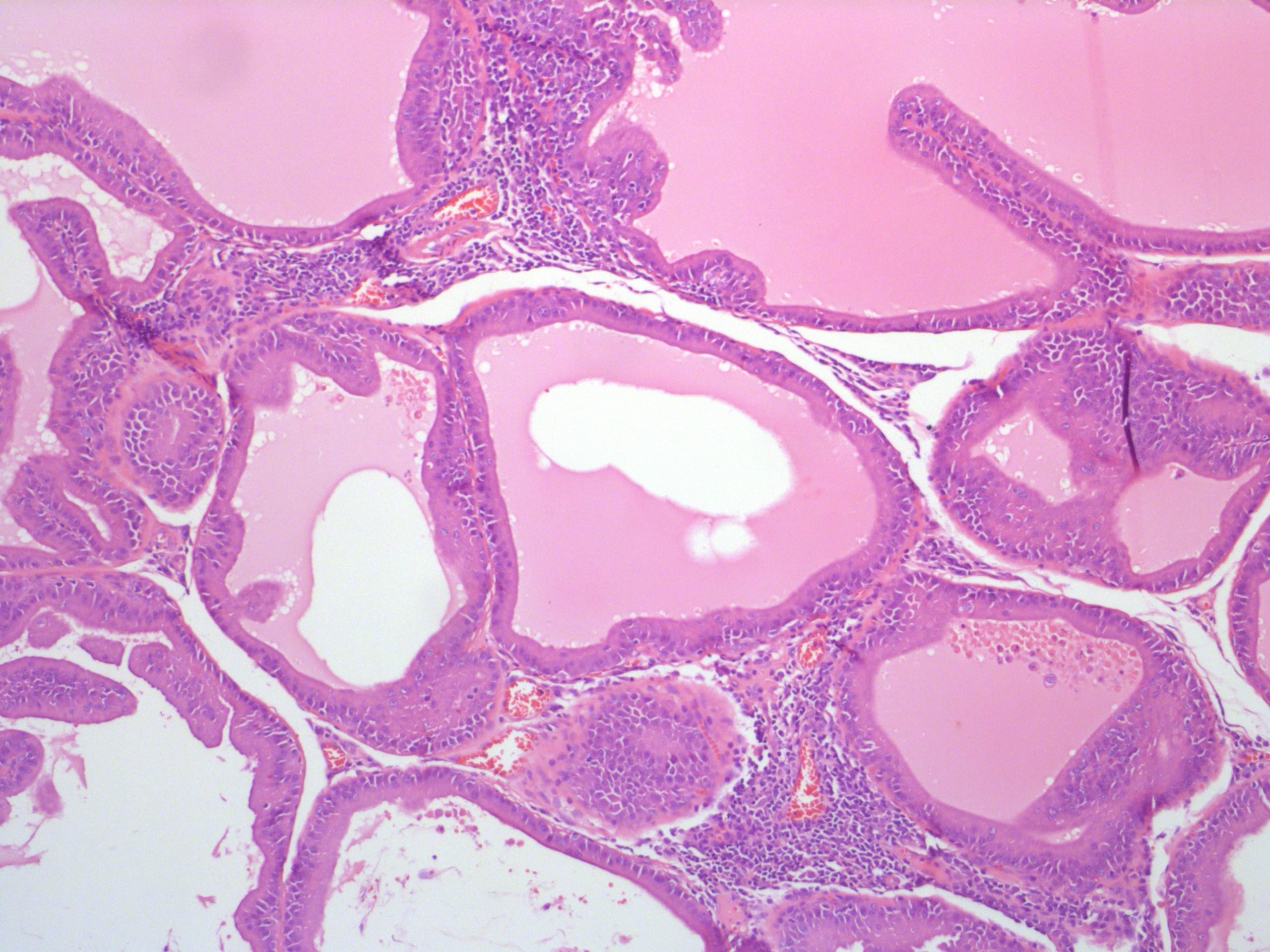 | 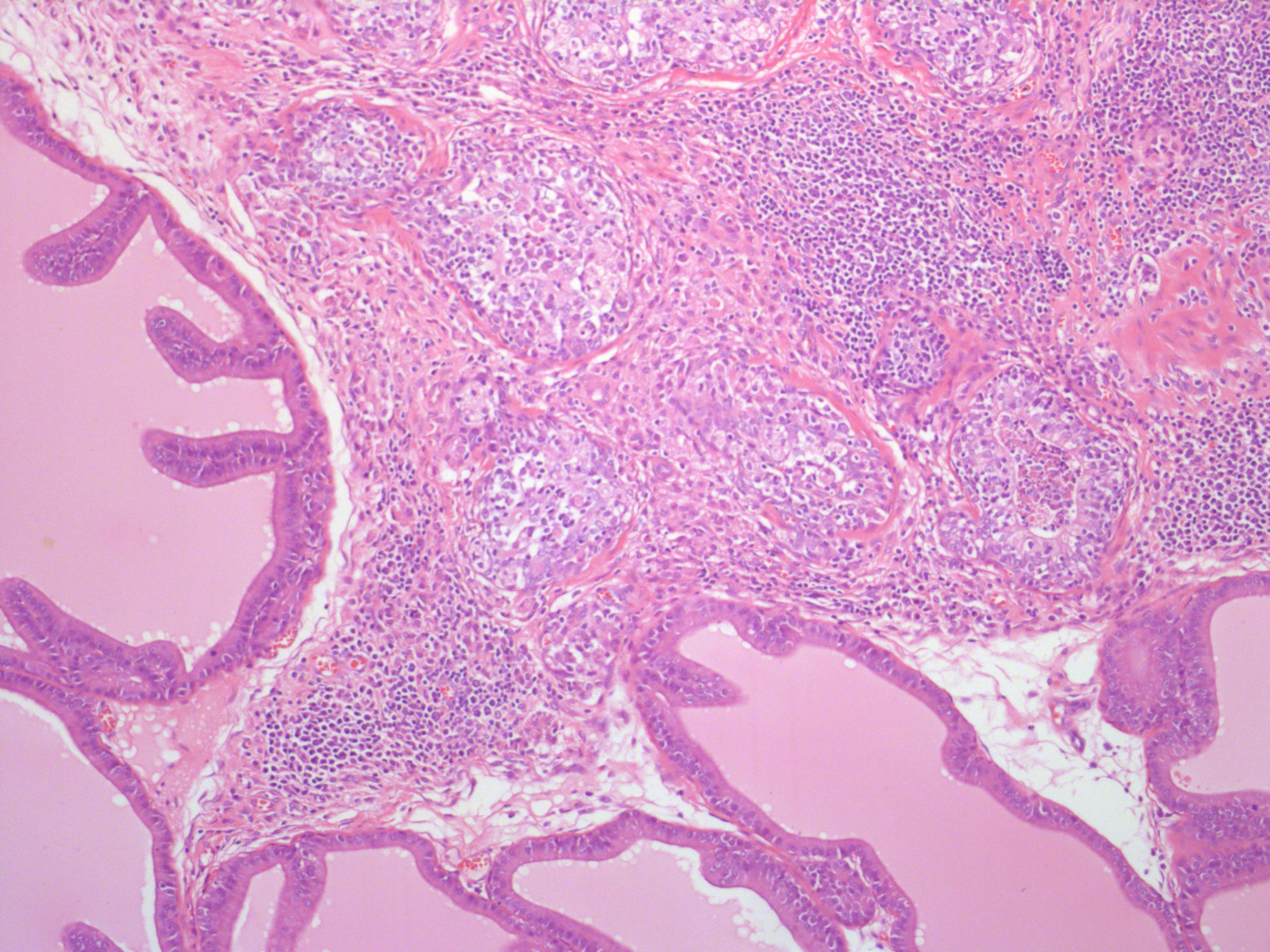 |
| Figure 3. #10003 in negative control group, prostate, infiltration of focal inflammatory cells, HE stained, ×100 | Figure 4. #13122 in high-dose group, prostate, chronic inflammation, HE stained, ×100 |

| Attached Pathology Table 1. Macroscopic observation in dissection — at the intermediate stage of administration (day 29) | | | | |
| --- | --- | --- | --- | --- |
| **Group** | **Negative control group** | **Low-dose group** | **Moderate-dose group** | **High-dose group** |
| **Dose (g/kg)** | 0 | 0.5 | 1.5 | 5 |
| **Number of animals** | **10** | **10** | **10** | **10** |
| **No obvious macroscopic abnormality in all the organs** | 10 | 10 | 10 | 10 |

| Attached Pathology Table 2. Macroscopic observation in dissection — at the end of administration (day 92) | | | | |
| --- | --- | --- | --- | --- |
| **Group** | **Negative control group** | **Low-dose group** | **Moderate-dose group** | **High-dose group** |
| **Dose (g/kg)** | 0 | 0.5 | 1.5 | 5 |
| **Number of animals** | **20** | **20** | **20** | **20** |
| **Esophagus** |  |  |  |  |
| Mung bean-sized yellowish white solid nodules | 0 | 0 | 0 | 1^a^ |

^a^: number of animals with lesions.

| Attached Pathology Table 3. Macroscopic observation in dissection—at the end of recovery (recovery day 29) | | | | |
| --- | --- | --- | --- | --- |
| **Group** | **Negative control group** | **Low-dose group** | **Moderate-dose group** | **High-dose group** |
| **Dose (g/kg)** | 0 | 0.5 | 1.5 | 5 |
| **Number of animals** | **10** | **10** | **10** | **10** |
| **Subcutaneous mass in the chest on the left side** | 0 | 0 | 1^a^ | 0 |

^a^: number of animals with lesions.

| Attached Pathology Table 4. Histopathological examination — at the intermediate stage of administration (day 29) | | | | | | | | | | | | | | | | | | | | |
| --- | --- | --- | --- | --- | --- | --- | --- | --- | --- | --- | --- | --- | --- | --- | --- | --- | --- | --- | --- | --- |
| **Group** | **Negative control group** | | | | | **High-dose group** | | | | | **Negative control group** | | | | | **High-dose group** | | | | |
| **Gender** | **Male** | | | | | | | | | | **Female** | | | | | | | | | |
| **Stage of administration** | **Intermediate stage of administration** | | | | | | | | | | | | | | | | | | | |
| **Dose/g/kg** | **0** | | | | | **5** | | | | | **0** | | | | | **5** | | | | |
| **Animal no.** | **10016** | **10017** | **10018** | **10019** | **10020** | **13136** | **13137** | **13138** | **13139** | **13140** | **20036** | **20037** | **20038** | **20039** | **20040** | **23156** | **23157** | **23158** | **23159** | **23160** |
| **Brain** | N | N | N | N | N | N | N | N | N | N | N | N | N | N | N | N | N | N | N | N |
| **Spinal cord (cervical, thoracic, and lumbar segments)** | N | N | N | N | N | N | N | N | N | N | N | N | N | N | N | N | N | N | N | N |
| **Sciatic nerve** | N | N | N | N | N | N | N | N | N | N | N | N | N | N | N | N | N | N | N | N |
| **Heart** | N | N | N | N | N | N | N | N |  | N | N | N | N | N | N | N | N | N | N | N |
| Focal myocardial degeneration with monocyte infiltration |  |  |  |  |  |  |  |  |  |  |  |  |  |  |  |  |  |  |  |  |
| 1+ |  |  |  |  |  |  |  |  | P |  |  |  |  |  |  |  |  |  |  |  |
| **Aorta** | N | N | N | N | N | N | N | N | N | N | N | N | N | N | N | N | N | N | N | N |
| **Trachea** | N | N | N | N | N | N | N | N | N | N | N | N | N | N | N | N | N | N | N | N |
| **Esophagus** | N | N | N | N | N | N | N | N | N | N | N | N | N | N | N | N | N | N | N | N |
| **Thyroid** | N | N | N | N | N | N | N | N | N | N | N |  | N | N | N | N | N | N | N | N |
| Ectopic thymus |  |  |  |  |  |  |  |  |  |  |  | P |  |  |  |  |  |  |  |  |
| **Parathyroid glands** | N | N | N | N | N | N | N | N | N | N | N | N | N | N | N | N | N | N | N | N |
| **Lungs** | N | N | N | N | N | N | N | N | N | N | N | N | N | N | N | N | N | N | N | N |
| **Main bronchi** | N | N | N | N | N | N | N | N | N | N | N | N | N | N | N | N | N | N | N | N |
| **Pancreas** | N | N | N | N | N | N | N | N | N | N | N | N | N | N | N | N | N | N | N | N |
| **Salivary glands (submandibular glands)** | N | N | N | N | N | N | N | N | N | N | N | N | N | N | N | N | N | N | N | N |
| **Submandibular lymph node** | N | N | N | N | N | N | N | N | N | N | N | N | N | N | N | N | N | N | N | N |
| **Stomach** | N | N | N | N | N | N | N | N | N | N | N | N | N | N | N | N | N | N | N | N |
| **Duodenum** | N | N | N | N | N | N | N | N | N | N | N | N | N | N | N | N | N | N | N | N |
| **Jejunum** | N | N | N | N | N | N | N | N | N | N | N | N | N | N | N | N | N | N | N | N |
| **Ileum** | N | N | N | N | N | N | N | N | N | N | N | N | N | N | N | N | N | N | N | N |
| **Cecum** | N | N | N | N | N | N | N | N | N | N | N | N | N | N | N | N | N | N | N | N |
| **Colon** | N | N | N | N | N | N | N | N | N | N | N | N | N | N | N | N | N | N | N | N |
| **Rectum** | N | N | N | N | N | N | N | N | N | N | N | N | N | N | N | N | N | N | N | N |
| **Skeletal muscle** | N | N | N | N | N | N | N | N | N | N | N | N | N | N | N | N | N | N | N | N |
| **Liver** |  |  | N | N |  |  |  | N | N |  |  |  | N | N | N |  | N | N | N |  |
| Focal monocyte infiltration |  |  |  |  |  |  |  |  |  |  |  |  |  |  |  |  |  |  |  |  |
| ± |  | P |  |  | P | P | P |  |  | P |  |  |  |  |  |  |  |  |  | P |
| Multifocal monocyte infiltration |  |  |  |  |  |  |  |  |  |  |  |  |  |  |  |  |  |  |  |  |
| ± | P |  |  |  |  |  |  |  |  |  | P | P |  |  |  | P |  |  |  |  |
| **Kidneys** | N |  | N | N |  |  |  |  | N | N | N |  | N | N | N |  | N | N |  |  |
| Multifocal hyaline droplet accumulation in renal tubules |  |  |  |  |  |  |  |  |  |  |  |  |  |  |  |  |  |  |  |  |
| ± |  |  |  |  |  |  |  | P |  |  |  |  |  |  |  |  |  |  |  |  |
| Focal interstitial monocyte infiltration |  |  |  |  |  |  |  |  |  |  |  |  |  |  |  |  |  |  |  |  |
| ± |  |  |  |  |  |  |  |  |  |  |  |  |  |  |  |  |  |  | P | P |
| Focal renal tubular basophil degeneration |  |  |  |  |  |  |  |  |  |  |  |  |  |  |  |  |  |  |  |  |
| ± |  | P |  |  | P | P | P |  |  |  |  |  |  |  |  |  |  |  |  | P |
| Cyst |  |  |  |  |  |  |  |  |  |  |  | P |  |  |  |  |  |  |  |  |
| Focal renal tubular hyaline cast |  |  |  |  |  |  |  |  |  |  |  |  |  |  |  |  |  |  |  |  |
| ± |  |  |  |  |  |  |  |  |  |  |  |  |  |  |  | P |  |  |  |  |
| Pyelectasia |  |  |  |  |  |  |  |  |  |  |  |  |  |  |  |  |  |  |  |  |
| 1+ |  |  |  |  |  |  | P |  |  |  |  |  |  |  |  |  |  |  |  |  |
| **Testes** | N | N | N | N | N | N | N | N | N | N | NA | NA | NA | NA | NA | NA | NA | NA | NA | NA |
| **Epididymides** | N | N | N | N | N | N | N | N | N | N | NA | NA | NA | NA | NA | NA | NA | NA | NA | NA |
| **Prostate** | N | N | N | N | N | N |  | N | N | N | NA | NA | NA | NA | NA | NA | NA | NA | NA | NA |
| Focal chronic inflammation |  |  |  |  |  |  |  |  |  |  |  |  |  |  |  |  |  |  |  |  |
| 1+ |  |  |  |  |  |  | P |  |  |  |  |  |  |  |  |  |  |  |  |  |
| **Seminal vesicle** | N | N | N | N | N | N | N | N | N | N | NA | NA | NA | NA | NA | NA | NA | NA | NA | NA |
| **Ovaries** | NA | NA | NA | NA | NA | NA | NA | NA | NA | NA | N | N | N | N | N | N | N | N | N | N |
| **Oviduct** | NA | NA | NA | NA | NA | NA | NA | NA | NA | NA | N | N | N | N | N | N | N | N | N | N |
| **Uterus** | NA | NA | NA | NA | NA | NA | NA | NA | NA | NA | N | N | N | N | N | N | N | N | N | N |
| **Cervix** | NA | NA | NA | NA | NA | NA | NA | NA | NA | NA | N | N | N | N | N | N | N | N | N | N |
| **Vagina** | NA | NA | NA | NA | NA | NA | NA | NA | NA | NA | N | N | N | N | N | N | N | N | N | N |
| **Skin** | N | N | N | N | N | N | N | N | N | N | N | N | N | N | N | N | N | N | N | N |
| **Mammary glands** | N | N | N | N | N |  | NE |  | N | N | N | N | N | N | N | N | N | N | N | N |
| Atrophy |  |  |  |  |  |  |  |  |  |  |  |  |  |  |  |  |  |  |  |  |
| 1+ |  |  |  |  |  |  |  | P |  |  |  |  |  |  |  |  |  |  |  |  |
| 2+ |  |  |  |  |  | P |  |  |  |  |  |  |  |  |  |  |  |  |  |  |
| **Adrenal glands** | N | N |  | N | N | N | N | N | N | N | N | N | N | N | N | N | N | N | N | N |
| Diffuse vacuolation of cortical fascicular zone |  |  |  |  |  |  |  |  |  |  |  |  |  |  |  |  |  |  |  |  |
| ± |  |  | P |  |  |  |  |  |  |  |  |  |  |  |  |  |  |  |  |  |
| **Pituitary gland** | N | N | N | N | N |  | N | N | N | N | N | N | N | N | N | N | N | N | N | N |
| Cyst |  |  |  |  |  | P |  |  |  |  |  |  |  |  |  |  |  |  |  |  |
| **Thymus** | N | N | N | N | N | N | N | N | N | N | N | N | N | N | N | N | N | N | N | N |
| **Spleen** | N | N | N |  |  |  | N |  |  |  | N | N | N | N | N | N | N | N | N | N |
| Extramedullary hemopoiesis hyperfunction |  |  |  |  |  |  |  |  |  |  |  |  |  |  |  |  |  |  |  |  |
| ± |  |  |  | P | P |  |  |  | P |  |  |  |  |  |  |  |  |  |  |  |
| 1+ |  |  |  |  |  | P |  | P |  | P |  |  |  |  |  |  |  |  |  |  |
| Marginal zone reduced |  |  |  |  |  |  |  |  |  |  |  |  |  |  |  |  |  |  |  |  |
| ± |  |  |  | P |  |  |  |  |  |  |  |  |  |  |  |  |  |  |  |  |
| **Mesenteric lymph node** | N | N | N | N | N | N | N | N | N | N | N | N | N | N | N | N | N | N | N | N |
| **Bladder** | N | N | N | N | N | N | N | N | N | N | N | N | N | N | N | N | N | N | N | N |
| **Eyes** | N | N | N | N | N | N | N | N | N | N | N | N | N | N | N | N | N | N | N | N |
| **Optic nerve** | N | N | N | N | N | NE | N | N | NE | N | N | N | N | N | N | N | N | N | N | N |
| **Harderian gland** | N | N | N | N | N | N | N | N | N | N | N | N | N | N | N | N | N | N | N | N |
| **Bone marrow (sternum)** | N | N | N | N | N | N | N | N | N | N | N | N | N | N | N | N | N | N | N | N |
| **Bone (femur)** | N | N | N | N | N | N | N | N | N | N | N | N | N | N | N | N | N | N | N | N |

Note: N = no obvious abnormality; AU = autolysed; NA = not applicable; NE = not examined; OE = only one side examined; P = presence of the lesion; minimal = ±; slight = 1+; moderate = 2+; marked = 3+.

## Attached Pathology Table 5. Histopathological examination — at the intermediate stage of administration (day 29) (with additional organ examination for low-dose group and moderate-dose group)

| **Group** | **Low-dose group** | | | | | **Moderate-dose group** | | | | |
| --- | --- | --- | --- | --- | --- | --- | --- | --- | --- | --- |
| **Gender** | **Male** | | | | | | | | | |
| **Stage of administration** | **Intermediate stage of administration (day 29)** | | | | | | | | | |
| **Dose/g/kg** | **0.5** | | | | | **1.5** | | | | |
| **Animal no.** | **11056** | **11057** | **11058** | **11059** | **11060** | **12096** | **12097** | **12098** | **12099** | **12100** |
| **Mammary glands** | N | N | N | N | N | N | N | N |  | N |
| Atrophy |  |  |  |  |  |  |  |  |  |  |
| 1+ |  |  |  |  |  |  |  |  | P |  |
| **Spleen** |  |  | N |  |  |  | N |  | N | N |
| Extramedullary hemopoiesis hyperfunction |  |  |  |  |  |  |  |  |  |  |
| ± |  |  |  |  |  |  |  | P |  |  |
| 1+ | P | P |  | P | P | P |  |  |  |  |
| **Kidneys** |  |  | N |  |  |  |  | N |  | N |
| Focal renal tubular basophil degeneration |  |  |  |  |  |  |  |  |  |  |
| ± | P | P |  |  | P | P | P |  | P |  |
| 1+ |  |  |  | P |  |  |  |  |  |  |
| Pyelectasia |  |  |  |  |  |  |  |  |  |  |
| ± |  |  |  |  | P |  |  |  |  |  |
| 1+ | P |  |  |  |  |  |  |  |  |  |
| 2+ |  |  |  |  |  | P |  |  |  |  |

Note: N = no obvious abnormality; AU = autolysed; NA = not applicable; NE = not observed in examination; OE = only one side examined; P = presence of the lesion; minimal = ±; slight = 1+; moderate = 2+; marked = 3+.

| Attached Pathology Table 6. Histopathological examination — at the end of administration (day 92) (male animals) | | | | | | | | | | | | | | | | | | | | |
| --- | --- | --- | --- | --- | --- | --- | --- | --- | --- | --- | --- | --- | --- | --- | --- | --- | --- | --- | --- | --- |
| **Group** | **Negative control group** | | | | | | | | | | **High-dose group** | | | | | | | | | |
| **Gender** | **Male** | | | | | | | | | | | | | | | | | | | |
| **Stage of administration** | **End of administration** | | | | | | | | | | | | | | | | | | | |
| **Dose/g/kg** | **0** | | | | | | | | | | **5** | | | | | | | | | |
| **Animal no.** | **10001** | **10002** | **10003** | **10004** | **10005** | **10006** | **10007** | **10008** | **10009** | **10010** | **13121** | **13122** | **13123** | **13124** | **13125** | **13126** | **13127** | **13128** | **13129** | **13130** |
| **Brain** | N | N | N | N | N | N | N | N | N | N | N | N | N | N | N | N | N | N | N | N |
| **Spinal cord (cervical, thoracic, and lumbar segments)** | N | N | N | N | N | N | N | N | N | N | N | N | N | N | N | N | N | N | N | N |
| **Sciatic nerve** | N | N | N | N | N | N | N | N | N | N | N | N | N | N | N | N | N | N | N | N |
| **Heart** | N | N |  | N | N | N | N | N | N | N | N | N | N | N | N | N | N | N | N | N |
| Focal myocardial degeneration with monocyte infiltration |  |  |  |  |  |  |  |  |  |  |  |  |  |  |  |  |  |  |  |  |
| 1+ |  |  | P |  |  |  |  |  |  |  |  |  |  |  |  |  |  |  |  |  |
| **Aorta** | N | N | N | N | N | N | N | N | N | N | N | N | N | N | N | N | N | N | N | N |
| **Trachea** | N | N | N | N | N | N | N | N | N | N | N | N | N | N | N | N | N | N | N | N |
| **Esophagus** | N | N | N | N | N | N | N | N | N | N | N | N | N | N |  | N | N | N | N | N |
| Pyogenic granuloma |  |  |  |  |  |  |  |  |  |  |  |  |  |  |  |  |  |  |  |  |
| 3+ |  |  |  |  |  |  |  |  |  |  |  |  |  |  | P |  |  |  |  |  |
| **Thyroid** | N | N | N | N | N | N | N |  | N | N | N | N | N | N | N | N | N |  | N | N |
| Follicular epithelial cell hypertrophy |  |  |  |  |  |  |  |  |  |  |  |  |  |  |  |  |  |  |  |  |
| 1+ |  |  |  |  |  |  |  | P |  |  |  |  |  |  |  |  |  | P |  |  |
| **Parathyroid glands** | N | N | N | N | N | N | N | N | N | N | N | N | N | N | N | N | N | N | N | N |
| **Lungs** | N | N | N | N | N | N | N |  | N | N |  | N |  | N | N |  | N | N | N | N |
| Focal osseous metaplasia |  |  |  |  |  |  |  |  |  |  |  |  |  |  |  |  |  |  |  |  |
| ± |  |  |  |  |  |  |  | P |  |  |  |  |  |  |  |  |  |  |  |  |
| Perivascular monocyte aggregation |  |  |  |  |  |  |  |  |  |  |  |  |  |  |  |  |  |  |  |  |
| ± |  |  |  |  |  |  |  |  |  |  |  |  | P |  |  |  |  |  |  |  |
| 1+ |  |  |  |  |  |  |  |  |  |  | P |  |  |  |  |  |  |  |  |  |
| Focal chronic inflammation |  |  |  |  |  |  |  |  |  |  |  |  |  |  |  |  |  |  |  |  |
| 1+ |  |  |  |  |  |  |  |  |  |  |  |  |  |  |  | P |  |  |  |  |
| **Main bronchi** | N | N | N | N | N | N | N | N | N | N | N | N | N | N | N | N | N | N | N | N |
| **Pancreas** | N | N | N | N | N |  | N | N | N |  | N | N |  | N | N | N | N | N | N |  |
| Focal pancreatic islet fibrosis |  |  |  |  |  |  |  |  |  |  |  |  |  |  |  |  |  |  |  |  |
| ± |  |  |  |  |  | P |  |  |  |  |  |  |  |  |  |  |  |  |  |  |
| Focal acinar atrophy |  |  |  |  |  |  |  |  |  |  |  |  |  |  |  |  |  |  |  |  |
| ± |  |  |  |  |  |  |  |  |  | P |  |  |  |  |  |  |  |  |  |  |
| Focal interstitial monocyte infiltration |  |  |  |  |  |  |  |  |  |  |  |  |  |  |  |  |  |  |  |  |
| ± |  |  |  |  |  |  |  |  |  |  |  |  | P |  |  |  |  |  |  | P |
| **Salivary glands (submandibular glands)** | N | N | N | N | N | N | N | N | N | N | N | N | N | N | N | N | N | N | N | N |
| **Submandibular lymph node** | N | N | N | N | N | N | N | N | N | N | N | N | N | N | N | N | N | N | N | N |
| **Stomach** | N | N | N | N | N | N | N | N | N | N | N | N | N | N | N | N | N | N | N | N |
| **Duodenum** | N | N | N | N | N | N | N | N | N | N | N | N | N | N | N | N | N | N | N | N |
| **Jejunum** | N | N | N | N | N | N | N | N | N | N | N | N | N | N | N | N | N | N | N | N |
| **Ileum** | N | N | N | N | N | N | N | N | N | N | N | N | N | N | N | N | N | N | N | N |
| **Cecum** | N | N | N | N | N | N | N | N | N | N | N | N | N | N | N | N | N | N | N | N |
| **Colon** | N | N | N | N | N | N | N | N | N | N | N | N | N | N | N | N | N | N | N | N |
| **Rectum** | N | N | N | N | N | N | N | N | N | N | N | N | N | N | N | N | N | N | N | N |
| **Skeletal muscle** | N | N | N | N | N | N | N | N | N | N | N | N | N | N | N | N | N | N | N | N |
| **Liver** | N | N | N |  | N |  | N |  | N |  | N | N |  |  |  | N |  | N |  | N |
| Focal monocyte infiltration |  |  |  |  |  |  |  |  |  |  |  |  |  |  |  |  |  |  |  |  |
| ± |  |  |  | P |  |  |  | P |  | P |  |  | P | P | P |  |  |  | P |  |
| Periportal area hepatocyte vacuolation |  |  |  |  |  |  |  |  |  |  |  |  |  |  |  |  |  |  |  |  |
| ± |  |  |  |  |  | P |  |  |  |  |  |  |  |  |  |  |  |  |  |  |
| 1+ |  |  |  |  |  |  |  |  |  |  |  |  |  |  |  |  | P |  |  |  |
| **Kidneys** |  |  |  |  |  |  | N | N |  |  |  |  |  |  |  |  | N |  |  |  |
| Multifocal hyaline droplet accumulation in renal tubules |  |  |  |  |  |  |  |  |  |  |  |  |  |  |  |  |  |  |  |  |
| ± |  |  |  |  |  |  |  |  |  |  |  | P |  | P |  |  |  |  |  |  |
| 1+ |  |  |  |  |  |  |  |  |  |  |  |  |  |  |  | P |  | P |  |  |
| 2+ |  |  |  |  |  |  |  |  |  |  | P |  |  |  |  |  |  |  |  |  |
| Focal interstitial monocyte infiltration |  |  |  |  |  |  |  |  |  |  |  |  |  |  |  |  |  |  |  |  |
| ± | P |  |  |  |  |  |  |  |  |  |  |  |  |  |  |  |  |  |  |  |
| Focal renal tubular basophil degeneration |  |  |  |  |  |  |  |  |  |  |  |  |  |  |  |  |  |  |  |  |
| ± | P |  |  |  | P | P |  |  |  | P |  |  |  |  | P |  |  | P | P |  |
| 1+ |  | P |  | P |  |  |  |  | P |  |  |  |  |  |  | P |  |  |  |  |
| Focal renal tubular hyaline cast |  |  |  |  |  |  |  |  |  |  |  |  |  |  |  |  |  |  |  |  |
| ± |  |  |  |  | P |  |  |  | P |  | P | P |  |  |  |  |  |  | P |  |
| Focal renal tubular erythrocyte cast |  |  |  |  |  |  |  |  |  |  |  |  |  |  |  |  |  |  |  |  |
| ± |  |  |  | P | P |  |  |  | P |  |  |  |  |  |  |  |  | P |  |  |
| Focal renal tubular ectasia |  |  |  |  |  |  |  |  |  |  |  |  |  |  |  |  |  |  |  |  |
| 1+ |  |  |  |  |  |  |  |  |  |  |  |  |  |  |  |  |  |  |  | P |
| Pyelectasia |  |  |  |  |  |  |  |  |  |  |  |  |  |  |  |  |  |  |  |  |
| ± |  |  |  |  |  | P |  |  |  |  |  |  |  |  |  |  |  |  |  |  |
| 1+ |  |  |  |  |  |  |  |  |  |  |  |  | P |  |  |  |  |  |  |  |
| **Testes** | N | N | N | N | N | N |  | N | N | N | N |  | N |  | N |  | N | N | N | N |
| Unilateral testicular interstitium edema |  |  |  |  |  |  |  |  |  |  |  |  |  |  |  |  |  |  |  |  |
| ± |  |  |  |  |  |  |  |  |  |  |  |  |  | P |  |  |  |  |  |  |
| 1+ |  |  |  |  |  |  | P |  |  |  |  |  |  |  |  | P |  |  |  |  |
| Multifocal atrophy of seminiferous tubules in unilateral testis |  |  |  |  |  |  |  |  |  |  |  |  |  |  |  |  |  |  |  |  |
| ± |  |  |  |  |  |  |  |  |  |  |  | P |  |  |  |  |  |  |  |  |
| **Epididymides** | N | N | N | N | N | N | N | N | N |  | N | N | N | N | N | N | N | N | N | N |
| Focal sperm granuloma in unilateral epididymal lumen |  |  |  |  |  |  |  |  |  |  |  |  |  |  |  |  |  |  |  |  |
| 1+ |  |  |  |  |  |  |  |  |  | P |  |  |  |  |  |  |  |  |  |  |
| **Prostate** | N | N |  |  |  |  | N | N |  | N | N |  | N |  |  |  |  |  |  |  |
| Focal chronic inflammation |  |  |  |  |  |  |  |  |  |  |  |  |  |  |  |  |  |  |  |  |
| 3+ |  |  |  |  |  |  |  |  |  |  |  | P |  |  |  |  |  |  |  |  |
| Multifocal chronic inflammation |  |  |  |  |  |  |  |  |  |  |  |  |  |  |  |  |  |  |  |  |
| ± |  |  |  |  |  |  |  |  |  |  |  |  |  |  |  | P | P |  |  |  |
| 1+ |  |  |  | P |  |  |  |  | P |  |  |  |  |  | P |  |  | P |  | P |
| 2+ |  |  |  |  |  |  |  |  |  |  |  |  |  | P |  |  |  |  |  |  |
| Focal interstitial monocyte infiltration |  |  |  |  |  |  |  |  |  |  |  |  |  |  |  |  |  |  |  |  |
| ± |  |  |  |  | P |  |  |  |  |  |  |  |  |  |  |  |  |  | P |  |
| 1+ |  |  | P |  |  |  |  |  |  |  |  |  |  |  |  |  |  |  |  |  |
| Focal glandular neutrophil infiltration |  |  |  |  |  |  |  |  |  |  |  |  |  |  |  |  |  |  |  |  |
| ± |  |  | P |  |  |  |  |  |  |  |  |  |  |  |  |  |  |  |  |  |
| 1+ |  |  |  |  |  | P |  |  |  |  |  |  |  |  |  |  |  |  |  |  |
| **Seminal vesicle** | N | N | N | N | N | N | N | N | N | N | N | N | N | N | N | N | N | N | N | N |
| **Skin** | N | N | N | N | N | N | N | N | N | N | N | N | N | N | N | N | N | N | N | N |
| **Mammary glands** | N | N | N | N | N | N | N | N | N | N | N | N | N | N | N | N | N | N | N | N |
| **Adrenal glands** | N |  |  | N |  | N |  |  | N |  |  | N | N | N |  | N | N | N |  | N |
| Diffuse vacuolation of cortical fascicular zone |  |  |  |  |  |  |  |  |  |  |  |  |  |  |  |  |  |  |  |  |
| ± |  | P |  |  |  |  | P |  |  |  | P |  |  |  | P |  |  |  | P |  |
| 1+ |  |  |  |  | P |  |  | P |  | P |  |  |  |  |  |  |  |  |  |  |
| Focal cortical cell hypertrophy |  |  |  |  |  |  |  |  |  |  |  |  |  |  |  |  |  |  |  |  |
| 1+ |  |  | P |  |  |  |  |  |  |  |  |  |  |  |  |  |  |  |  |  |
| **Pituitary gland** | N | N | N | N | N | N | N | N | N | N | N | N | N | N | N | N | N | N | N | N |
| **Thymus** | N | N | N | N | N | N | N | N | N | N | N | N | N | N | N | N | N | N | N | N |
| **Spleen** | N | N | N | N | N | N |  | N | N | N | N | N |  | N | N | N | N | N | N | N |
| Extramedullary hemopoiesis hyperfunction |  |  |  |  |  |  |  |  |  |  |  |  |  |  |  |  |  |  |  |  |
| 1+ |  |  |  |  |  |  | P |  |  |  |  |  | P |  |  |  |  |  |  |  |
| **Mesenteric lymph node** | N | N | N | N | N | N | N | N | N | N | N | N | N | N | N | N | N | N | N | N |
| **Bladder** | N | N | N | N | N | N | N | N | N | N | N | N | N | N | N | N | N | N | N | N |
| **Eyes** | N | N | N | N | N | N | N | N | N | N | N | N | N | N | N | N | N | N | N | N |
| **Optic nerve** | N | N | N | N | N | N | N | N | N | N | N | N | N | N | N | N | N | N | N | N |
| **Harderian gland** | N | N | N | N | N | N | N | N |  | N | N | N | N | N | N | N | N | N | N | N |
| Focal interstitial monocyte infiltration |  |  |  |  |  |  |  |  |  |  |  |  |  |  |  |  |  |  |  |  |
| ± |  |  |  |  |  |  |  |  | P |  |  |  |  |  |  |  |  |  |  |  |
| **Bone marrow (sternum)** | N | N | N | N | N | N | N | N | N | N | N | N | N | N | N | N | N | N | N | N |
| **Bone (femur)** | N | N | N | N | N | N | N | N | N | N | N | N | N | N | N | N | N | N | N | N |

Note: N = no obvious abnormality; AU = autolysed; NA = not applicable; NE = not observed in examination; OE = only one side examined; P = presence of the lesion; minimal = ±; slight = 1+; moderate = 2+; marked = 3+.

| Attached Pathology Table 7. Histopathological examination — at the end of administration (day 92) (female animals) | | | | | | | | | | | | | | | | | | | | |
| --- | --- | --- | --- | --- | --- | --- | --- | --- | --- | --- | --- | --- | --- | --- | --- | --- | --- | --- | --- | --- |
| **Group** | **Negative control group** | | | | | | | | | | **High-dose group** | | | | | | | | | |
| **Gender** | **Female** | | | | | | | | | | | | | | | | | | | |
| **Stage of administration** | **End of administration** | | | | | | | | | | | | | | | | | | | |
| **Dose/g/kg** | **0** | | | | | | | | | | **5** | | | | | | | | | |
| **Animal no.** | **20021** | **20022** | **20023** | **20024** | **20025** | **20026** | **20027** | **20028** | **20029** | **20030** | **23141** | **23142** | **23143** | **23144** | **23145** | **23146** | **23147** | **23148** | **23149** | **23150** |
| **Brain** | N | N | N | N | N | N | N | N | N | N | N | N | N | N | N | N | N | N | N | N |
| **Spinal cord (cervical, thoracic, and lumbar segments)** | N | N | N | N | N | N | N | N | N | N | N | N | N | N | N | N | N | N | N | N |
| **Sciatic nerve** | N | N | N | N | N | N | N | N | N | N | N | N | N | N | N | N | N | N | N | N |
| **Heart** | N | N | N | N | N | N | N | N | N | N | N | N | N | N | N | N | N | N | N | N |
| **Aorta** | N | N | N | N | N | N | N | N | N | N | N | N | N | N | N | N | N | N | N | N |
| **Trachea** | N | N | N | N | N | N | N | N | N | N | N | N | N | N | N | N | N | N | N | N |
| **Esophagus** | N | N | N | N | N | N | N | N | N | N | N | N | N | N | N | N | N | N | N | N |
| **Thyroid** | N | N | N | N | N | N | N | N | N | N | N | N | N | N | N | N | N | N | N | N |
| **Parathyroid glands** | N | NE | N | N | N | NE | N | N | N | N | N | N | N | N | N | N | N | N | N | N |
| **Lungs** |  | N | N | N | N | N | N | N |  | N | N | N | N |  | N | N | N | N | N | N |
| Perivascular monocyte aggregation |  |  |  |  |  |  |  |  |  |  |  |  |  |  |  |  |  |  |  |  |
| ± |  |  |  |  |  |  |  |  | P |  |  |  |  |  |  |  |  |  |  |  |
| 1+ | P |  |  |  |  |  |  |  |  |  |  |  |  | P |  |  |  |  |  |  |
| Focal chronic inflammation |  |  |  |  |  |  |  |  |  |  |  |  |  |  |  |  |  |  |  |  |
| 1+ | P |  |  |  |  |  |  |  |  |  |  |  |  |  |  |  |  |  |  |  |
| **Main bronchi** | N | N | N | N | N | N | N | N | N | N | N | N | N | N | N | N | N | N | N | N |
| **Pancreas** | N | N | N |  | N | N | N |  | N | N | N | N | N | N | N | N | N | N | N |  |
| Focal interstitial monocyte infiltration |  |  |  |  |  |  |  |  |  |  |  |  |  |  |  |  |  |  |  |  |
| ± |  |  |  | P |  |  |  |  |  |  |  |  |  |  |  |  |  |  |  | P |
| Focal acinar atrophy |  |  |  |  |  |  |  |  |  |  |  |  |  |  |  |  |  |  |  |  |
| 2+ |  |  |  |  |  |  |  | P |  |  |  |  |  |  |  |  |  |  |  |  |
| **Salivary glands (submandibular glands)** | N | N |  | N | N | N | N | N | N | N | N | N | N | N | N | N | N | N | N | N |
| Interstitial hemorrhage with inflammation |  |  |  |  |  |  |  |  |  |  |  |  |  |  |  |  |  |  |  |  |
| 1+ |  |  | P |  |  |  |  |  |  |  |  |  |  |  |  |  |  |  |  |  |
| **Submandibular lymph node** | N | N | N | N | N | N | N | N | N | N | N | N | N | N | N | N | N | N | N | N |
| **Stomach** | N | N | N | N |  |  | N | N | N | N | N | N | N | N | N | N | N | N | N | N |
| Submucosal layer edema |  |  |  |  |  |  |  |  |  |  |  |  |  |  |  |  |  |  |  |  |
| 1+ |  |  |  |  |  | P |  |  |  |  |  |  |  |  |  |  |  |  |  |  |
| 2+ |  |  |  |  | P |  |  |  |  |  |  |  |  |  |  |  |  |  |  |  |
| **Duodenum** | N | N | N | N | N | N | N | N | N | N | N | N | N | N | N | N | N | N | N | N |
| **Jejunum** | N | N | N | N | N | N | N | N | N | N | N | N | N | N | N | N | N | N | N | N |
| **Ileum** | N | N | N | N | N | N | N | N | N | N | N | N | N | N | N | N | N | N | N | N |
| **Cecum** | N | N | N | N | N | N | N | N | N | N | N | N | N | N |  |  | N | N | N | N |
| Submucosal layer hemorrhage |  |  |  |  |  |  |  |  |  |  |  |  |  |  |  |  |  |  |  |  |
| 2+ |  |  |  |  |  |  |  |  |  |  |  |  |  |  | P |  |  |  |  |  |
| Mucosal atrophy |  |  |  |  |  |  |  |  |  |  |  |  |  |  |  |  |  |  |  |  |
| 2+ |  |  |  |  |  |  |  |  |  |  |  |  |  |  |  | P |  |  |  |  |
| **Colon** | N | N | N | N | N | N | N | N | N | N | N | N | N | N | N | N | N | N | N | N |
| **Rectum** | N | N | N | N | N | N | N | N | N | N | N | N | N | N | N | N | N | N | N | N |
| **Skeletal muscle** | N | N | N | N | N | N | N | N | N | N | N | N | N | N | N | N | N | N | N | N |
| **Liver** | N |  | N | N |  | N | N |  | N | N | N | N | N | N | N | N |  | N | N |  |
| Focal monocyte infiltration |  |  |  |  |  |  |  |  |  |  |  |  |  |  |  |  |  |  |  |  |
| ± |  |  |  |  | P |  |  | P |  |  |  |  |  |  |  |  | P |  |  | P |
| Focal hepatocyte necrosis with inflammation |  |  |  |  |  |  |  |  |  |  |  |  |  |  |  |  |  |  |  |  |
| 1+ |  | P |  |  |  |  |  |  |  |  |  |  |  |  |  |  |  |  |  |  |
| Focal hemorrhage |  |  |  |  |  |  |  |  |  |  |  |  |  |  |  |  |  |  |  |  |
| 1+ |  | P |  |  |  |  |  |  |  |  |  |  |  |  |  |  |  |  |  |  |
| **Kidneys** | N |  |  | N | N | N | N | N | N | N |  | N | N | N | N | N | N | N | N | N |
| Focal renal tubular hyaline cast |  |  |  |  |  |  |  |  |  |  |  |  |  |  |  |  |  |  |  |  |
| ± |  | P | P |  |  |  |  |  |  |  |  |  |  |  |  |  |  |  |  |  |
| Focal interstitial monocyte infiltration |  |  |  |  |  |  |  |  |  |  |  |  |  |  |  |  |  |  |  |  |
| ± |  | P |  |  |  |  |  |  |  |  |  |  |  |  |  |  |  |  |  |  |
| Focal infarction |  |  |  |  |  |  |  |  |  |  |  |  |  |  |  |  |  |  |  |  |
| 1+ |  |  |  |  |  |  |  |  |  |  | P |  |  |  |  |  |  |  |  |  |
| **Ovaries** | N | N | N | N | N | N | N | N | N | N | N | N | N | N | N | N | N | N | N | N |
| **Oviduct** | N | N | N | N | N | N | N | N | N | N | N | N | N | N | N | N | N | N | N | N |
| **Uterus** | N | N | N | N | N | N | N | N | N | N | N | N | N | N | N | N | N | N | N | N |
| **Cervix** | N | N | N | N | N | N | N | N | N | N | N | N | N | N | N | N | N | N | N | N |
| **Vagina** | N | N | N | N | N | N | N | N | N | N | N | N | N | N | N | N | N | N | N | N |
| **Skin** | N | N | N | N | N | N | N | N | N | N | N | N | N | N | N | N | N | N | N | N |
| **Mammary glands** | N | N | N | N | N | N | N | N | N | N | N | N | N | N | N | N | N | N | N | N |
| **Adrenal glands** | N | N | N | N | N | N | N | N | N | N | N | N | N | N | N | N | N | N | N | N |
| **Pituitary gland** | N | N | N | N | N | N | N | N | N | N | N | N | N | N | N | N | N | N | N | N |
| **Thymus** | N | N | N | N |  | N | N | N | N | N | N | N | N | N | N | N | N | N | N | N |
| Epithelioid cell proliferation |  |  |  |  |  |  |  |  |  |  |  |  |  |  |  |  |  |  |  |  |
| ± |  |  |  |  | P |  |  |  |  |  |  |  |  |  |  |  |  |  |  |  |
| **Spleen** | N | N |  |  | N |  | N | N |  | N | N |  |  | N |  | N |  | N | N |  |
| Pigmentation of yellowish brown color phagocytosed by macrophages |  |  |  |  |  |  |  |  |  |  |  |  |  |  |  |  |  |  |  |  |
| 1+ |  |  |  |  |  | P |  |  | P |  |  | P | P |  | P |  | P |  |  |  |
| Extramedullary hemopoiesis hyperfunction |  |  |  |  |  |  |  |  |  |  |  |  |  |  |  |  |  |  |  |  |
| ± |  |  |  | P |  |  |  |  |  |  |  |  | P |  |  |  |  |  |  | P |
| 1+ |  |  | P |  |  |  |  |  |  |  |  |  |  |  |  |  |  |  |  |  |
| **Mesenteric lymph node** | N | N | N | N | N | N | N | N | N | N | N | N | N | N | N | N | N | N | N | N |
| **Bladder** | N | N | N | N | N | N | N | N | N | N | N | N | N | N | N | N | N | N | N | N |
| **Eyes** | N | N | N | N | N | N | N | N | N | N | N | N | N | N | N | N | N | N | N | N |
| **Optic nerve** | N | N | N | N | N | N | N | N | N | N | N | N | N | N | N | N | N | N | N | N |
| **Harderian gland** | N | N | N | N | N | N | N | N | N | N | N | N | N | N | N | N | N | N | N | N |
| **Bone marrow (sternum)** | N | N | N | N | N | N | N | N | N | N | N | N | N | N | N | N | N | N | N | N |
| **Bone (femur)** | N | N | N | N | N | N | N | N | N | N | N | N | N | N | N | N | N | N | N | N |

Note: N = no obvious abnormality; AU = autolysed; NA = not applicable; NE = not observed in examination; OE = only one side examined; P = presence of the lesion; minimal = ±; slight = 1+; moderate = 2+; marked = 3+.

| Attached Pathology Table 8. Histopathological examination — at the end of administration (day 92) (with additional organ examination for low-dose group and moderate-dose group) | | | | | | | | | | | | | | | | | | | | |
| --- | --- | --- | --- | --- | --- | --- | --- | --- | --- | --- | --- | --- | --- | --- | --- | --- | --- | --- | --- | --- |
| **Group** | **Low-dose group** | | | | | | | | | | **Moderate-dose group** | | | | | | | | | |
| **Gender** | **Male** | | | | | | | | | | | | | | | | | | | |
| **Stage of administration** | **End of administration** | | | | | | | | | | | | | | | | | | | |
| **Dose/g/kg** | **0.5** | | | | | | | | | | **1.5** | | | | | | | | | |
| **Animal no.** | **11041** | **11042** | **11043** | **11044** | **11045** | **11046** | **11047** | **11048** | **11049** | **11050** | **12081** | **12082** | **12083** | **12084** | **12085** | **12086** | **12087** | **12088** | **12089** | **12090** |
| **Prostate** |  |  |  | N |  | N |  | N |  |  |  | N |  | N |  | N |  |  |  |  |
| Multifocal chronic inflammation |  |  |  |  |  |  |  |  |  |  |  |  |  |  |  |  |  |  |  |  |
| 2+ |  |  |  |  |  |  |  |  |  |  |  |  |  |  | P |  |  |  |  | P |
| Focal interstitial monocyte infiltration |  |  |  |  |  |  |  |  |  |  |  |  |  |  |  |  |  |  |  |  |
| ± | P | P |  |  |  |  | P |  |  |  | P |  | P |  |  |  |  | P |  |  |
| 1+ |  |  |  |  |  |  |  |  |  | P |  |  |  |  |  |  | P |  | P |  |
| Focal glandular neutrophil infiltration |  |  |  |  |  |  |  |  |  |  |  |  |  |  |  |  |  |  |  |  |
| ± |  | P |  |  |  |  |  |  | P |  | P |  |  |  |  |  |  |  | P |  |
| 1+ |  |  | P |  | P |  |  |  |  |  |  |  |  |  |  |  |  |  |  |  |
| **Kidneys** |  |  | N |  |  |  | N |  | N |  |  | N |  |  | N | N | N |  | N | N |
| Multifocal interstitial monocyte infiltration |  |  |  |  |  |  |  |  |  |  |  |  |  |  |  |  |  |  |  |  |
| 1+ |  |  |  |  |  |  |  |  |  |  |  |  | P |  |  |  |  |  |  |  |
| Focal renal tubular basophil degeneration |  |  |  |  |  |  |  |  |  |  |  |  |  |  |  |  |  |  |  |  |
| ± |  |  |  |  |  | P |  |  |  |  | P |  |  |  |  |  |  |  |  |  |
| 1+ | P | P |  |  | P |  |  |  |  | P |  |  |  | P |  |  |  |  |  |  |
| Multifocal renal tubular basophil degeneration |  |  |  |  |  |  |  |  |  |  |  |  |  |  |  |  |  |  |  |  |
| 2+ |  |  |  |  |  |  |  |  |  |  |  |  | P |  |  |  |  |  |  |  |
| Focal renal tubular hyaline cast |  |  |  |  |  |  |  |  |  |  |  |  |  |  |  |  |  |  |  |  |
| ± |  |  |  |  |  |  |  |  |  |  |  |  |  |  |  |  |  | P |  |  |
| 1+ | P |  |  |  |  |  |  |  |  | P |  |  |  |  |  |  |  |  |  |  |
| Pyelectasia |  |  |  |  |  |  |  |  |  |  |  |  |  |  |  |  |  |  |  |  |
| ± |  | P |  | P |  |  |  |  |  |  | P |  |  | P |  |  |  |  |  |  |
| 2+ |  |  |  |  |  |  |  |  |  |  |  |  | P |  |  |  |  |  |  |  |
| Focal renal tubular erythrocyte cast |  |  |  |  |  |  |  |  |  |  |  |  |  |  |  |  |  |  |  |  |
| ± |  |  |  |  |  |  |  | P |  |  |  |  |  |  |  |  |  |  |  |  |

Note: N = no obvious abnormality; AU = autolysed; NA = not applicable; NE = not observed in examination; OE = only one side examined; P = presence of the lesion; minimal = ±; slight = 1+; moderate = 2+; marked = 3+.

| Attached Pathology Table 9. Histopathological examination — at the end of recovery (recovery day 29) | | | | | | | | | | | | | | | | | | | | |
| --- | --- | --- | --- | --- | --- | --- | --- | --- | --- | --- | --- | --- | --- | --- | --- | --- | --- | --- | --- | --- |
| **Group** | **Negative control group** | | | | | **High-dose group** | | | | | **Negative control group** | | | | | **High-dose group** | | | | |
| **Gender** | **Male** | | | | | | | | | | **Female** | | | | | | | | | |
| **Stage of administration** | **End of recovery** | | | | | | | | | | | | | | | | | | | |
| **Dose/g/kg** | **0** | | | | | **5** | | | | | **0** | | | | | **5** | | | | |
| **Animal no.** | **10011** | **10012** | **10013** | **10014** | **10015** | **13131** | **13132** | **13133** | **13134** | **13135** | **20031** | **20032** | **20033** | **20034** | **20035** | **23151** | **23152** | **23153** | **23154** | **23155** |
| **Brain** | N | N | N | N | N | N | N | N | N | N | N | N | N | N | N | N | N | N | N | N |
| **Spinal cord (cervical cord, thoracic cord, and lumbar cord)** | N | N | N | N | N | N | N | N | N | N | N | N | N | N | N | N | N | N | N | N |
| **Sciatic nerve** | N | N | N | N | N | N | N | N | N | N | N | N | N | N | N | N | N | N | N | N |
| **Heart** | N | N |  |  | N | N |  | N | N | N | N | N | N | N | N | N | N | N | N | N |
| Focal monocyte infiltration |  |  |  |  |  |  |  |  |  |  |  |  |  |  |  |  |  |  |  |  |
| ± |  |  | P | P |  |  | P |  |  |  |  |  |  |  |  |  |  |  |  |  |
| **Aorta** | N | N | N | N | N | N | N | N | N | N | N | N | N | N | N | N | N | N | N | N |
| **Trachea** | N | N | N | N | N | N | N | N | N | N | N | N | N | N | N | N | N | N | N | N |
| **Esophagus** | N | N | N | N | N | N | N | N | N | N | N | N | N | N | N | N | N | N | N | N |
| **Thyroid** | N | N | N | N | N | N | N | N | N | N | N | N | N | N | N | N | N | N | N | N |
| **Parathyroid glands** | N | NE | N | N | NE | NE | N | N | N | N | N | N | N | N | N | N | N | N | N | N |
| **Lungs** |  | N | N |  |  |  |  | N | N |  | N | N |  | N | N |  |  |  | N | N |
| Perivascular monocyte aggregation |  |  |  |  |  |  |  |  |  |  |  |  |  |  |  |  |  |  |  |  |
| ± |  |  |  | P |  | P | P |  |  | P |  |  |  |  |  |  | P | P |  |  |
| 1+ | P |  |  |  |  |  |  |  |  |  |  |  | P |  |  | P |  |  |  |  |
| Focal macrophage aggregation in alveolar space |  |  |  |  |  |  |  |  |  |  |  |  |  |  |  |  |  |  |  |  |
| ± |  |  |  | P | P | P | P |  |  |  |  |  |  |  |  |  |  |  |  |  |
| 1+ | P |  |  |  |  |  |  |  |  |  |  |  |  |  |  |  |  |  |  |  |
| Focal chronic inflammation |  |  |  |  |  |  |  |  |  |  |  |  |  |  |  |  |  |  |  |  |
| 1+ |  |  |  |  |  |  |  |  |  |  |  |  |  |  |  |  |  | P |  |  |
| **Main bronchi** | N | N | N | N | N | N | N | N | N | N | N | N | N | N | N | N | N | N | N | N |
| **Pancreas** | N | N | N | N | N | N | N | N | N | N | N | N | N | N | N | N |  | N | N | N |
| Focal interstitial monocyte infiltration |  |  |  |  |  |  |  |  |  |  |  |  |  |  |  |  |  |  |  |  |
| ± |  |  |  |  |  |  |  |  |  |  |  |  |  |  |  |  | P |  |  |  |
| **Salivary glands (submandibular glands)** | N | N | N | N | N | N | N | N | N | N | N | N | N | N | N | N | N | N | N | N |
| **Submandibular lymph node** | N | N | N | N | N | N | N | N | N | N | N | N | N | N | N | N | N | N | N | N |
| **Stomach** | N | N | N | N | N | N | N | N | N | N | N | N | N | N | N | N | N | N | N | N |
| **Duodenum** | N | N | N | N | N | N | N | N | N | N | N | N | N | N | N | N | N | N | N | N |
| **Jejunum** | N | N | N | N | N | N | N | N | N | N | N | N | N | N | N | N | N | N | N | N |
| **Ileum** | N | N | N | N | N | N | N | N | N | N | N | N | N | N | N | N | N | N | N | N |
| **Cecum** | N | N | N | N | N | N | N | N | N | N | N | N | N | N | N | N | N | N | N | N |
| **Colon** | N | N | N | N | N | N | N | N | N | N | N | N | N | N | N | N | N | N | N | N |
| **Rectum** | N | N | N | N | N | N | N | N | N | N | N | N | N | N | N | N | N | N | N | N |
| **Skeletal muscle** | N | N | N | N | N | N | N | N | N | N | N | N | N | N | N | N | N | N | N | N |
| **Liver** | N |  | N |  |  | N | N | N | N | N |  | N |  |  | N | N | N |  | N |  |
| Focal monocyte infiltration |  |  |  |  |  |  |  |  |  |  |  |  |  |  |  |  |  |  |  |  |
| ± |  |  |  | P | P |  |  |  |  |  |  |  |  |  |  |  |  |  |  |  |
| Ligament lipidosis |  |  |  |  |  |  |  |  |  |  |  |  |  |  |  |  |  |  |  |  |
| 1+ |  |  |  |  |  |  |  |  |  |  | P |  | P |  |  |  |  |  |  |  |
| Focal hepatocyte necrosis with inflammation |  |  |  |  |  |  |  |  |  |  |  |  |  |  |  |  |  |  |  |  |
| ± |  | P |  |  |  |  |  |  |  |  | P |  |  |  |  |  |  | P |  |  |
| Periportal area hepatocyte hypertrophy |  |  |  |  |  |  |  |  |  |  |  |  |  |  |  |  |  |  |  |  |
| ± |  |  |  |  |  |  |  |  |  |  |  |  |  |  |  |  |  |  |  | P |
| 1+ |  |  |  |  |  |  |  |  |  |  |  |  |  | P |  |  |  |  |  |  |
| **Kidneys** |  | N | N |  |  |  |  |  | N | N |  | N | N | N | N | N | N | N |  | N |
| Multifocal hyaline droplet accumulation in renal tubules |  |  |  |  |  |  |  |  |  |  |  |  |  |  |  |  |  |  |  |  |
| ± |  |  |  |  |  |  | P |  |  |  |  |  |  |  |  |  |  |  |  |  |
| Focal renal tubular basophil degeneration |  |  |  |  |  |  |  |  |  |  |  |  |  |  |  |  |  |  |  |  |
| ± |  |  |  |  |  |  |  |  |  |  |  |  |  |  |  |  |  |  | P |  |
| 1+ | P |  |  | P | P | P |  |  |  |  |  |  |  |  |  |  |  |  |  |  |
| Focal renal tubular hyaline cast |  |  |  |  |  |  |  |  |  |  |  |  |  |  |  |  |  |  |  |  |
| ± |  |  |  | P |  |  |  | P |  |  | P |  |  |  |  |  |  |  |  |  |
| 1+ |  |  |  |  |  | P |  |  |  |  |  |  |  |  |  |  |  |  |  |  |
| Pyelectasia |  |  |  |  |  |  |  |  |  |  |  |  |  |  |  |  |  |  |  |  |
| ± |  |  |  | P |  |  |  |  |  |  |  |  |  |  |  |  |  |  |  |  |
| **Testes** | N |  | N | N | N | N | N | N | N |  | NA | NA | NA | NA | NA | NA | NA | NA | NA | NA |
| Unilateral testicular interstitium edema |  |  |  |  |  |  |  |  |  |  |  |  |  |  |  |  |  |  |  |  |
| 1+ |  | P |  |  |  |  |  |  |  | P |  |  |  |  |  |  |  |  |  |  |
| **Epididymides** | N | N | N | N | N | N | N | N | N | N | NA | NA | NA | NA | NA | NA | NA | NA | NA | NA |
| **Prostate** | N | N |  | N |  |  |  |  | N | N | NA | NA | NA | NA | NA | NA | NA | NA | NA | NA |
| Multifocal chronic inflammation |  |  |  |  |  |  |  |  |  |  |  |  |  |  |  |  |  |  |  |  |
| ± |  |  | P |  |  |  |  |  |  |  |  |  |  |  |  |  |  |  |  |  |
| 1+ |  |  |  |  | P |  | P |  |  |  |  |  |  |  |  |  |  |  |  |  |
| Focal interstitial monocyte infiltration |  |  |  |  |  |  |  |  |  |  |  |  |  |  |  |  |  |  |  |  |
| ± |  |  |  |  |  | P |  | P |  |  |  |  |  |  |  |  |  |  |  |  |
| **Seminal vesicle** | N | N | N | N | N | N | N | N | N | N | NA | NA | NA | NA | NA | NA | NA | NA | NA | NA |
| **Ovaries** | NA | NA | NA | NA | NA | NA | NA | NA | NA | NA | N | N | N | N | N | N | N | N | N | N |
| **Oviduct** | NA | NA | NA | NA | NA | NA | NA | NA | NA | NA | N | N | N | N | N | N | N | N | N | N |
| **Uterus** | NA | NA | NA | NA | NA | NA | NA | NA | NA | NA | N | N | N | N | N | N | N | N | N | N |
| **Cervix** | NA | NA | NA | NA | NA | NA | NA | NA | NA | NA | N | N | N | N | N | N | N | N | N | N |
| **Vagina** | NA | NA | NA | NA | NA | NA | NA | NA | NA | NA | N | N | N | N | N | N | N | N | N | N |
| **Skin** | N | N | N | N | N | N | N | N | N | N | N | N | N | N | N | N | N | N | N | N |
| **Mammary glands** | N | N | N | N | N | N | N | N | N | N | N | N | N | N | N | N | N | N | N | N |
| **Adrenal glands** | N | N | N | N | N | N | N | N | N | N | N | N | N | N | N | N | N | N | N | N |
| **Pituitary gland** | N | N | N | N | N | N |  | N | N | N | N | N | N | N | N | N | N | N | N | N |
| Congestion |  |  |  |  |  |  |  |  |  |  |  |  |  |  |  |  |  |  |  |  |
| 2+ |  |  |  |  |  |  | P |  |  |  |  |  |  |  |  |  |  |  |  |  |
| **Thymus** | N | N | N | N | N | N | N | N | N | N | N | N | N | N | N | N |  | N | N | N |
| Epithelioid cell proliferation |  |  |  |  |  |  |  |  |  |  |  |  |  |  |  |  |  |  |  |  |
| 1+ |  |  |  |  |  |  |  |  |  |  |  |  |  |  |  |  | P |  |  |  |
| **Spleen** |  |  | N | N | N |  |  | N | N | N |  |  |  | N |  |  |  |  | N | N |
| Pigmentation of yellowish brown color phagocytosed by macrophages |  |  |  |  |  |  |  |  |  |  |  |  |  |  |  |  |  |  |  |  |
| ± |  |  |  |  |  |  | P |  |  |  |  |  |  |  |  |  |  |  |  |  |
| 1+ | P |  |  |  |  | P |  |  |  |  | P | P | P |  |  | P |  | P |  |  |
| 2+ |  |  |  |  |  |  |  |  |  |  |  |  |  |  |  |  | P |  |  |  |
| Extramedullary hemopoiesis hyperfunction |  |  |  |  |  |  |  |  |  |  |  |  |  |  |  |  |  |  |  |  |
| 1+ |  | P |  |  |  | P |  |  |  |  |  |  |  |  |  |  |  |  |  |  |
| Congestion |  |  |  |  |  |  |  |  |  |  |  |  |  |  |  |  |  |  |  |  |
| 2+ |  |  |  |  |  |  |  |  |  |  |  |  |  |  | P |  |  |  |  |  |
| **Mesenteric lymph node** | N | N | N | N | N | N | N | N | N | N | N | N | N | N | N | N | N | N | N | N |
| **Bladder** | N | N | N | N | N | N | N | N | N | N | N | N | N | N | N | N | N | N | N | N |
| **Eyes** | N | N | N | N | N | N | N | N | N | N | N | N | N | N | N | N | N | N | N | N |
| **Optic nerve** | N | N | N | N | N | N | N | N | NE | N | N | N | N | N | N | N | N | N | N | NE |
| **Harderian gland** | N | N | N | N | N |  | N | N | N | N | N | N | N | N | N | N | N | N | N | N |
| Focal interstitial monocyte infiltration |  |  |  |  |  |  |  |  |  |  |  |  |  |  |  |  |  |  |  |  |
| ± |  |  |  |  |  | P |  |  |  |  |  |  |  |  |  |  |  |  |  |  |
| **Bone marrow (sternum)** | N | N | N | N | N | N | N | N | N | N | N | N | N | N | N | N | N | N | N | N |
| **Bone (femur)** | N | N | N | N | N | N | N | N | N | N | N | N | N | N | N | N | N | N | N | N |

Note: N = no obvious abnormality; AU = autolysed; NA = not applicable; NE = not examined; OE = only one side examined; P = presence of the lesion; minimal = ±; slight = 1+; moderate = 2+; marked = 3+.

| Attached Pathology Table 9. Histopathological examination — at the end of recovery (recovery day 29) | |
| --- | --- |
| **Group** | **Moderate-dose group** |
| **Gender** | **Female** |
| **Stage of administration** | **End of recovery** |
| **Dose/g/kg** | **1.5** |
| **Animal no.** | **22113** |
| **Subcutaneous mass in the chest on the left side** |  |
| Breast adenocarcinoma | P |

Note: N = no obvious abnormality; AU = autolysed; NA = not applicable; NE = not examined; OE = only one side examined; P = presence of the lesion; minimal = ±; slight = 1+; moderate = 2+; marked = 3+.

## Attached Pathology Table 10. Histopathological examination — at the end of recovery (recovery day 29) (with additional organ examination for low-dose group and moderate-dose group)

| **Group** | **Low-dose group** | | | | | **Moderate-dose group** | | | | |
| --- | --- | --- | --- | --- | --- | --- | --- | --- | --- | --- |
| **Gender** | **Male** | | | | | | | | | |
| **Stage of administration** | **End of recovery** | | | | | | | | | |
| **Dose/g/kg** | **0.5** | | | | | **1.5** | | | | |
| **Animal no.** | **11051** | **11052** | **11053** | **11054** | **11055** | **12091** | **12092** | **12093** | **12094** | **12095** |
| **Kidneys** | N |  |  |  | N | N |  | N |  |  |
| Focal renal tubular basophil degeneration |  |  |  |  |  |  |  |  |  |  |
| ± |  | P |  |  |  |  |  |  | P | P |
| 1+ |  |  |  | P |  |  |  |  |  |  |
| Pyelectasia |  |  |  |  |  |  |  |  |  |  |
| ± |  |  | P |  |  |  |  |  |  |  |
| Focal renal tubular hyaline cast |  |  |  |  |  |  |  |  |  |  |
| ± |  |  |  | P |  |  | P |  |  |  |

Note: N = no obvious abnormality; AU = autolysed; NA = not applicable; NE = not examined; OE = only one side examined; P = presence of the lesion; minimal = ±; slight = 1+; moderate = 2+; marked = 3+.
